# Snapshots of Cooperative Trimetallic Alkene Hydrogenation

**DOI:** 10.1021/jacs.5c15492

**Published:** 2025-12-05

**Authors:** Till L. Kalkuhl, Israel Fernández, Terrance J. Hadlington

**Affiliations:** † Fakultät für Chemie, School of Natural Sciences, TU München, Lichtenberg Strasse 4, 85749 Garching, Germany; ‡ Departamento de Química Orgánica I, Facultad de Ciencias Químicas and Centro de Innovación en Química Avanzada, 16734 Universidad Complutense de Madrid, 28040 Madrid, Spain

## Abstract

Bimetallic cooperativity and synergistic effects have emerged as powerful tools that can enhance the reactivity and selectivity of catalytic systems, offering a pathway toward the development of catalysts based on Earth-abundant elements. Although not uncommon in nature, discrete molecular systems featuring more than two cooperative metal sites are virtually unknown. Herein we report the design of a well-defined molecular heterotrimetallic [Ga_2_Ni] system, which is effective in alkene hydrogenation catalysis. Species representing ‘snapshots’ of the catalytic cycle are isolated through consecutive addition of ethylene and dihydrogen, allowing for in-depth experimental mechanistic elucidation of two sequential 2-electron oxidative processes. Alongside combined computational, kinetic, and isotopic labeling studies, these findings reveal a fully mapped hydrogenation mechanism in which all three metal sites partake in the alkene hydrogenation process. This system thus provides unique insights into cooperative molecular multimetallic catalysis and establishes a blueprint for rationally designed multicentered cooperativity beyond established diatomic concepts.

## Graphical abstract

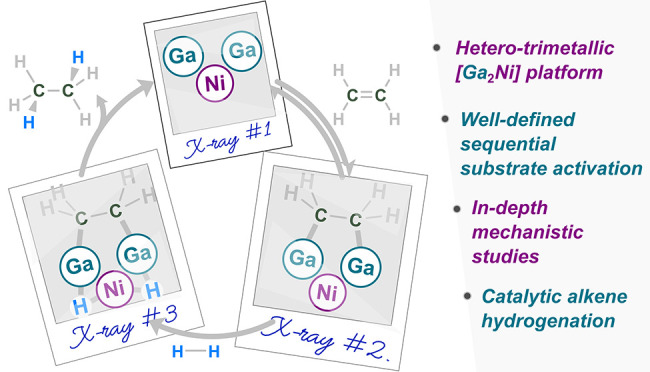

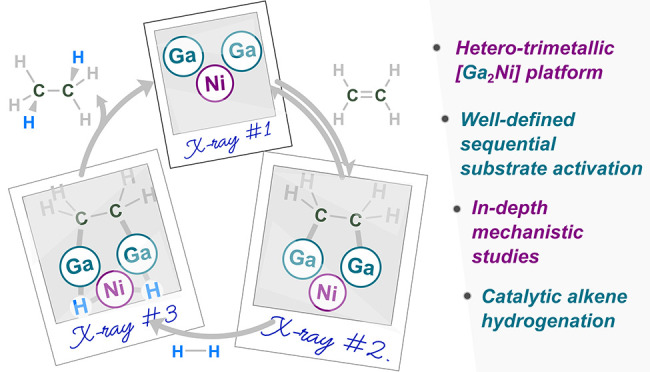

## Introduction

Catalysis is an integral part of chemical synthesis today.^1^ In homogeneous catalysis, the vast majority of known systems rely on a single metal center for bond activation and formation (Figure 1(a)).^1–3^ In contrast to single-site catalysts, certain metalloenzymes operate utilizing complex, multimetallic active sites, where cooperativity among metal centers and cofactors enables exceptional activity and selectivity.^4–9^ For example, bimetallic [FeFe]/[FeNi] hydrogenases reversibly cleave H_2_ between the two metal centers in a bridging fashion.^10,11^ Nature also goes beyond bimetallic systems: nitrogenases and multicopper oxidases illustrate how multiple redox-active centers can orchestrate complex multielectron transformations,^12,13^ e.g., where nitrogenases utilize a Fe_7_S_9_C–Mo site for binding and reduction of N_2_, while an adjacent Fe_8_–S_7_ cluster shuttles electrons for ammonia formation.^13^ While studies of metalloenzymes have highlighted the importance of cooperativity between distinct sites in driving catalytic performance, replicating this level of complexity in artificial and molecular systems remains a major challenge.^14–16^ Nevertheless, significant breakthroughs have been made in this field, often centered on unique ligand design concepts.^17–19^

**Figure 1:**
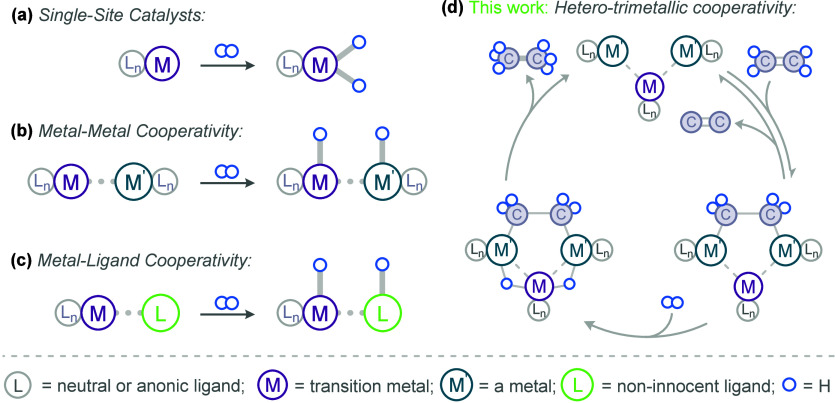
Bond scission by (a) a “classic” single-site system, (b) metal–metal cooperativity (MMC), (c) metal–ligand cooperativity (MLC), (d) cooperative trimetallic cooperativity, demonstrated in this work. We note that hydride ligands in (b) and (c) are also known to be bridging.

Drawing inspiration from these highly complex natural systems, cooperative catalysis, particularly involving metal–metal cooperativity (MMC, Figure 1(b)),^20–30^ and metal–ligand cooperativity (MLC, Figure 1(c)),^31–35^ has emerged as a central strategy for developing efficient and sustainable systems for synthetic chemistry. MLC includes strategies such as ligand aromatization/dearomatization,^36,37^ Lewis acidic Z-type ligands,^38–41^ Lewis basic hydrogen-acceptor ligands,^42–44^ and redox noninnocent ligands.^45,46^ Known examples of MMC leverage scaffold-supported metal–metal proximity,^41,47,48^ and, less frequently, direct metal–metal bonds,^49–51^ where bond activation across the metal centers can lead to the formation of the monometallic counterparts if no tethering backbone is present.^51,52^ Main-group metallo-ligands have come to play a pivotal role in MLC and especially MMC,^41,53–55^ leading to numerous bifunctional systems demonstrating cooperative small-molecule activation modes beyond the reach of single-site complexes. Nevertheless, the utility of such systems in catalysis, and in particular systems for which a definitive mechanism is explored, remains very rare indeed. This is in part due to the synthetic challenges in accessing well-defined bifunctional systems, and the transient nature of key intermediates.

Building on the potential of well-defined bimetallics for cooperative bond activation, moving toward higher-order cooperativity represents a promising frontier. Such systems may offer the possibility of greater tuneability, and facilitate multielectron redox processes with multiple redox-active metal sites in close spatial proximity, moving conceptually closer to the complex systems found in nature. Concepts in ligand design which allow for the formation of such complexes (i.e., with >2 metal centers) have been forthcoming from e.g., the groups of Betley,^19,56,57^ Agapie,^17,58^ and Murray.^18,59,60^ Indeed, the benefits of multimetallic species are echoed in transition-metal cluster and nanoparticle chemistry, where enhanced catalytic performance is frequently observed.^61–64^ Yet, mechanistic insights into such systems remain scarce, owing to inherent challenges regarding *in-operando* characterization and intermediate isolation.^65–67^ These issues can be overcome in discrete molecular systems, which benefit from the suite of characterization methods available under homogeneous solution-state conditions.^68^ Indeed, though well-defined higher-order molecular systems are still widely seen as curiosities, recent examples demonstrate their remarkable proficiency in small-molecule and bond activation.^69–73^ For instance, Hou and co-workers reported a trinuclear titanium hepta-hydride complex, capable of carbon–carbon bond cleavage in benzene,^74^ and even promoting stoichiometric hydroamination of ethylene starting from dinitrogen.^70,75^ With these exciting advances, the further development of rational design principles which give access to well-defined multimetallic systems seems a highly promising pathway to unlocking and understanding higher-order cooperative catalysis.

Herein, we report the rational synthesis of a heterotrimetallic molecular system, and its utility in alkene hydrogenation catalysis. The well-defined bisgallylene ligated nickel(0) complex (**2**) serves as a platform for the isolation of stable species pertaining to distinct stages of the catalytic cycle: consecutive addition of ethylene and dihydrogen allows for the isolation and full characterization of ethylene-bridged bis­(gallyl)-nickel complex **3**, and the corresponding ethylene-bridged bis­(gallyl)-nickeldihydride complex **4** (see Figure 1(d)). Complex **2** thus operates as a single platform capable of two distinct oxidative transformations. Combined with density functional theory (DFT), kinetic and isotope labeling studies, full mechanistic elucidation unveils cooperation between all three metal sites in each step of the cycle, underpinning the synergistic nature of this trimetallic system. The catalytic scope is demonstrated by the hydrogenation of a series of over 31 alkenes under 5 bar H_2_ pressure, between 25 and 100 °C. These findings offer an in-depth view of unprecedented bond activation and formation pathways inaccessible to mono- or bifunctional systems. More broadly, they highlight the potential of higher-order cooperativity in confined spaces, paving a way toward the guided design of sustainable cooperative catalysts.

## Results and Discussion

### Synthesis of a Molecular Trimetallic [Ga_2_Ni] System

Our previous work has demonstrated that the functionalization of heavier *p*-block systems with a chelating phosphine arm is an effective strategy for accessing robust transition metal complexes featuring low-valent *p*-block (metallo)-ligands. Such species, for example, show a significant thermal stability, remaining intact under reductive catalytic conditions (e.g., hydrogenation, hydrosilylation).^76–78^ In addition, the phosphine chelate constrains the binding angle of the ligand, creating a strongly electrophilic binding center. We aimed to utilize this strategy in accessing transition metal complexes bearing multiple *p*-block metallo-ligands, with a well-defined spatially constrained binding environment. In addition to our earlier Ga^I^–Ni^II^ work, Lu and Peters have demonstrated the noninnocence of gallane and boryl ligands, respectively, in e.g., H_2_ scission in Ni complexes.^79,80^ To this end, we employed a gallium­(III) hydride embedded in a phosphine-appended amido ligand framework, *viz*. ^Cy^LGaH_2_ (**1**; ^Cy^L = {[Cy_2_PCH_2_Si­(iPr)_2_ (Dipp)­N}; Dipp = 2,6-^i^Pr_2_C_6_H_3_), as a masked LGa^I^ species. This synthetic approach was disclosed by Aldridge and co-workers in 2017 in Rh complexation,^81^ and more recently by our group.^82^ The reaction of two equiv of **1** with Ni­(cod)_2_ (cod = 1,5-cyclooctadiene), from −78 °C to ambient temperature, leads to the formation of deep red reaction mixtures. Isolation of crystalline material from these reactions revealed the formation of trimetallic bis­(gallylene) nickel(0) complex **2** (Figure 2), through complete elimination of hydrogen, as confirmed by the absence of Ga-*H* and Ni-*H* signals in both the ^1^H NMR and ATR-IR spectra of the isolated solid. Analysis of ^1^H NMR spectra of the crude reaction mixtures indicates the formation of cyclooctene and cyclooctane – this would suggest that gaseous H_2_ is not eliminated from the mixture, but rather that the 1,5-cyclooctadiene ligand behaves as a hydrogen ‘sponge’. In these mixtures, the sole product is **2**, formed quantitatively.

**Figure 2:**
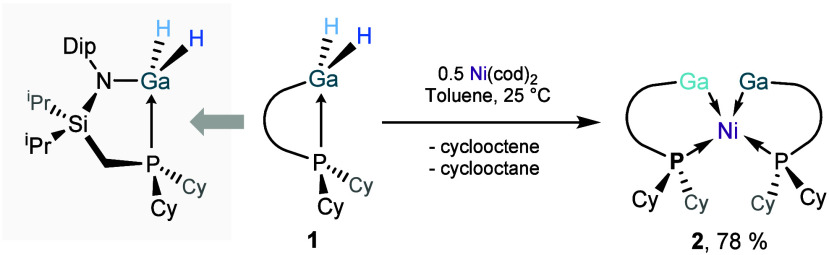
Synthesis of the trimetallic [Ga_2_Ni] complex **2**.

The molecular structure of **2** reveals two key observations: *(i)* the three metal centers (e.g., Ga–Ni–Ga) are in close spatial proximity (*d*_Ga1Ni1_ = 2.248(1) Å; *d*_Ga2Ni1_ = 2.249(1) Å), confirming the formation of a distinct multimetallic molecular species, and *(ii)* the coordination environment around nickel is highly distorted from an ideal tetrahedral geometry. The observed P–Ni–P angle of 146.44(8)° and Ga–Ni–Ga angle of 87.54(4)° deviate significantly from the expected tetrahedral angle of 109.5°, which we attribute to a combination of the steric bulk from the cyclohexyl-substituted phosphine groups, and the geometric constraints imposed by the chelating ligand framework. The geometry around Ni is best described using the *τ*_*4*_ *’* parameter, yielding a value of 0.59. This is indicative of a constrained intermediate between seesaw (*τ*_*4*_ *’* = 0.24) and tetrahedral (*τ*_*4*_ *’* = 1.0) geometries.^83^ This leads to a short Ga···Ga distance of 3.111(2) Å, which is within the sum of the van der Waals radii (3.74 Å), but longer than known Ga–Ga single bonds (e.g., 2.3320–2.551 Å) ([Fn fn3]).^84,85^

Density Functional Theory (DFT) calculations at the dispersion-corrected (RI)-BP86-D3BJ/def2-SVP level were carried out to gain further insights into the bonding situation in complex **2** (Figure 3). The optimized geometry concurs rather well with the experimental (i.e., X-ray derived) data, in particular, the Ni–Ga bond lengths are nearly identical (2.244 Å), although the computed Ga···Ga bond distance is somewhat shorter (2.933 Å). As expected, the Quantum Theory of Atoms in Molecules analysis (QTAIM, Figure 3(a)) locates a bond critical point (BCP) and a bond path (BP) running between each gallium center and the transition metal, confirming the occurrence of two Ga–Ni bonds (computed Mayer bond orders of 1.02). The positive value of the electron density Laplacian at the BCP (∇^2^ρ = +0.091) suggests that both interactions are dative bonds. In contrast, no BCP or BP were found to represent a Ga···Ga bond, indicating a much weaker interaction between these centers. This is confirmed by the computed much lower Mayer bond order of 0.34. Looking deeper into this interaction using the Natural Bond Orbital (NBO) method, a weak contact between the two gallium centers is found, derived from a degree of delocalization of the lone-pair at each metal into the p_*z*_ atomic orbital of the other (associated stabilization energy, Δ*E* ^*(2)*^ = −30.1 kcal·mol^–1^). We hypothesize that this weak interaction is partially responsible for the distorted geometry observed for **2** in the solid state. More quantitative insight into the nature of the Ga–Ni bonds can be gained with the Energy Decomposition Analysis-Natural Orbital for Chemical Valence (EDA-NOCV, Figure 3(b)) method. By using neutral [^Cy^LGa·Ni] and [^Cy^LGa] fragments, we found that these bonding interactions result from two donor–acceptor (dative) interactions, namely the donation from the lone-pair at each gallium atom to a vacant, largely s-character atomic orbital at nickel, coupled with a weaker backdonation from a doubly occupied d-atomic orbital of the nickel center to a vacant p-atomic orbital of the gallium center.

**Figure 3:**
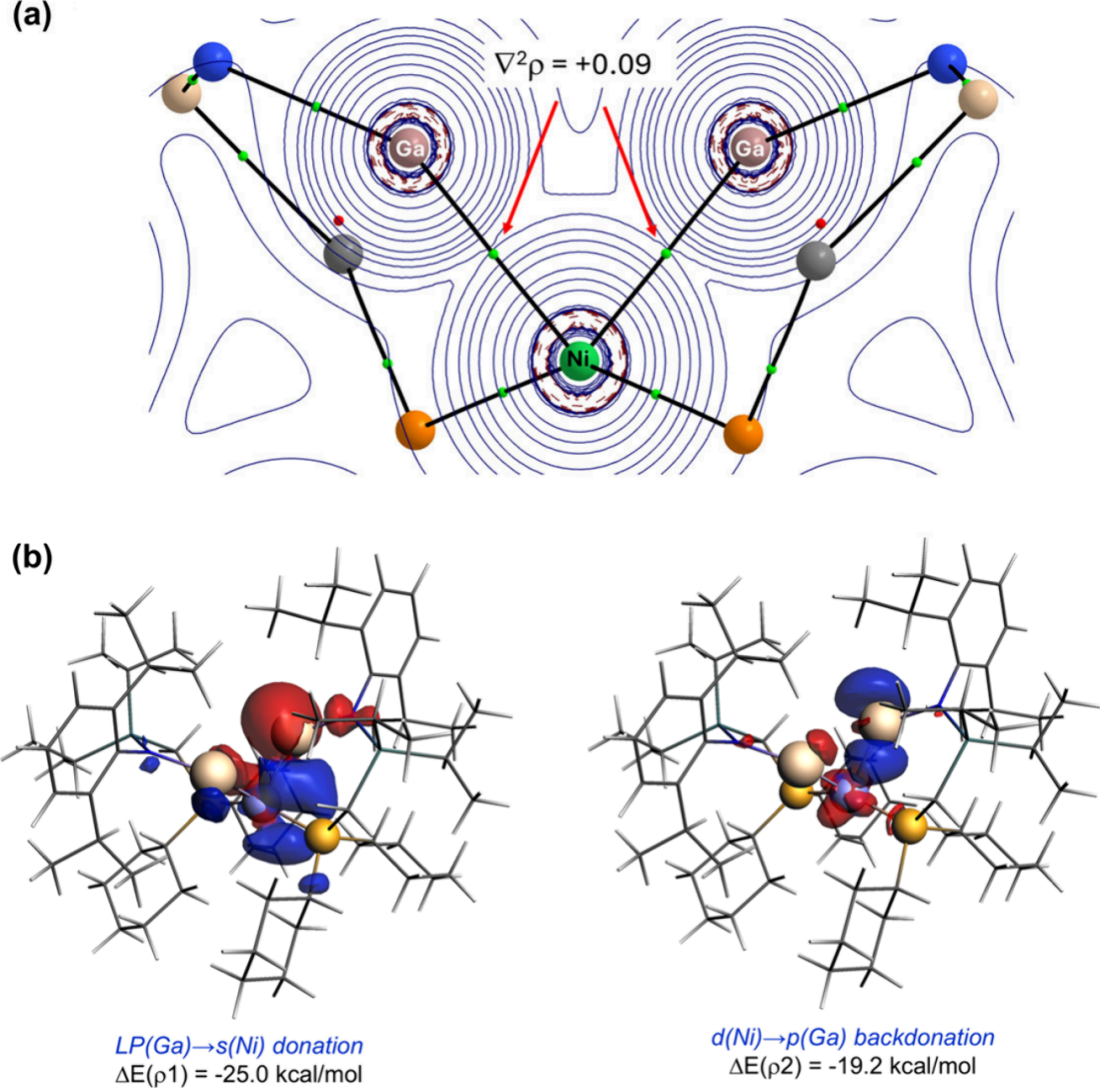
(a) Contour map of the Laplacian function of complex **2** in the Ga–Ni–Ga molecular plane (green spheres represent BCPs); (b) contour plots of the main NOCV deformation densities ρ (isosurface value of 0.001 au) and associated energies ΔE­(ρ) in complex **2**. The electronic charge flows from red to blue. Data computed at the ZORA-BP86-D3BJ/DZP//(RI)-BP86-D3BJ/def2-SVP level.

Having established the formation of a complex bearing a spatially well-defined trimetallic core in **2**, we sought to explore potential cooperative bond activation pathways. We were surprised to find that this species does not activate dihydrogen, even after heating at 60 °C for 18 h, though this does emphasize the efficacy of the developed synthetic route for this species. In contrast, solutions of **2** rapidly react with ethylene to form a single new species, **3** (Figure 4(a)). Analysis of reaction mixtures indicates a single new ^31^P­{^1^H} NMR resonance (δ = 18.1 ppm), and a shifted ^1^H NMR spectrum featuring a broad 4H singlet at δ = 0.33 ppm, attributable to a single C_2_H_4_ unit. Structural analysis of **3** reveals that addition of ethylene occurs at the [Ga_2_] interface, bridging the two Ga centers; as such, **3** is best described as an ethylene-bridged bis­(gallyl) nickel complex. The molecular structure of **3** indicates that the Ga–Ni distances are only slightly increased on moving from gallylene to gallyl ligands (e.g., **2**: 2.248(1) Å; **3**: 2.3284(1) Å), thereby indicating only a small change in bond order - this is confirmed by the computed slightly lower Mayer bond order of 0.95 in **3**, vs 1.02 in **2**. We attribute this to the phosphine arm in the ligand backbone, which prevents linearization and thwarts multiple bond formation in **2**. The bridging C_2_H_4_ moiety leads to a highly constrained tetradentate ligand with a slightly shorter Ga···Ga distance of 2.959(1) Å, and a C–C distance of 1.519(7) Å indicative of a single C–C σ-bond in this bridging unit.^86^

**Figure 4:**
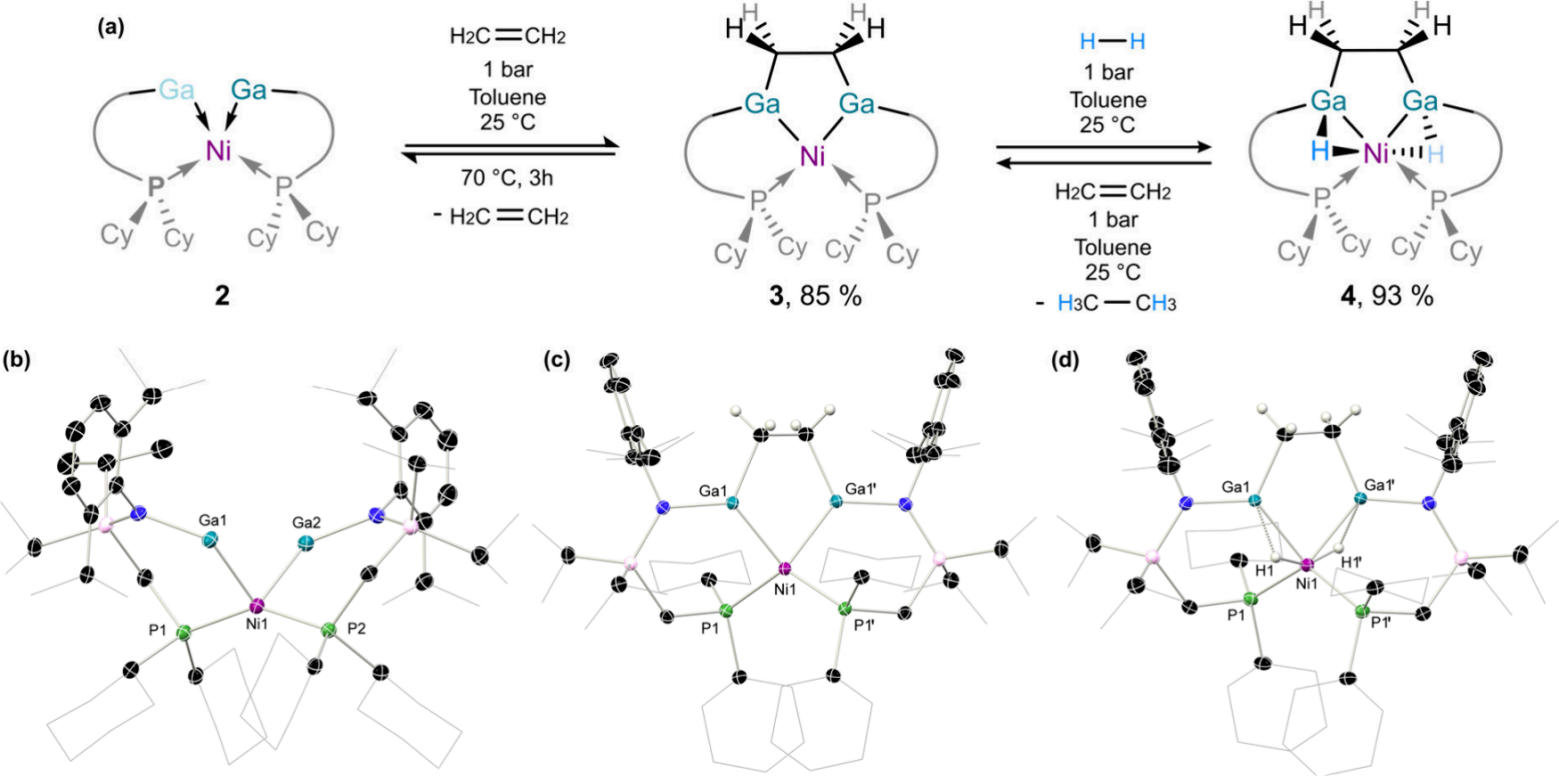
(a) Sequential activation of ethylene and dihydrogen by complex **2**, in forming complexes **3** and **4**, and the conditions for interconversion of these species. Crystal structures of complexes (b) **2**, (c) **3**, and (d) **4**, with hydrogens omitted for clarity aside from those on the ethylene bridge in **3** and **4**, and bridging hydride ligands in **4**. Thermal ellipsoids are shown as 30% probability. Selected bond lengths (Å) and angles (°) for **2**: Ga1–Ni1 2.248(1) Å, Ga2–Ni1 2.249(1) Å,, P1–Ni1 2.200(2) Å, Ga1–N1 1.926(7) Å, Ga1–Ni1–Ga2 87.54(4)°, P1–Ni1–P2 146.44(8)°, Ni1–Ga1–N1 133.4(2)°. For **3**: Ga1–Ni1 2.2284(1) Å, Ga1–C32 1.999(9) Å, C32–C32 1.519(7) Å, P1–Ni1 2.23(0) Å, Ga1–N1 1.876(6) Å, Ga1–Ni1–Ga1 79.9(8)°, P1–Ni1–P1 118.7(1)°, Ni1–Ga1–N1 125.9(3)°. For **4**: Ga1–Ni1 2.33(7) Å, Ga1–C32 1.995(4) Å, C32–C32 1.506(6) Å, P1–Ni1 2.23(5) Å, Ga1–N1 1.880(3) Å, Ga1–H1 1.75(4) Å, Ni1–H1 1.45(0) Å, Ga1–Ni1–Ga1 79.0(8)°, P1–Ni1–P1 120.6(5)°, Ni1–Ga1–N1 124.7(5)°.

Despite the facile nature of this ethylene addition reaction, dissolution of crystalline samples of **3** with mild heating leads to complete liberation of the ethylene fragment, and regeneration of **2**. Under more controlled conditions, a C_6_D_6_ solution of complex **3** was heated at 70 °C for 3 h, resulting in complete formation of **2**, as confirmed by ^1^H and ^31^P­{^1^H} NMR spectroscopic analyses (Figures S35 and S36 in Supporting Information). To gain insight into the thermodynamics of the ethylene liberation process an Eyring analysis was performed, giving values of Δ*H* ^*‡*^ = 19.36 kcal·mol^–1^, Δ*S* ^*‡*^ = −32.99 cal·mol^–1^·K^–1^, and Δ*G*_*298*_^*‡*^ = 29.20 kcal·mol^–1^; the moderate Gibbs free energy of activation is consistent with a reaction that is thermally accessible but proceeding slowly at ambient temperature. This aligns with the observed negative activation entropy, indicating an ordered transition state in the rate-determining step, and is likely driven by the elimination of gaseous ethylene.

Given that Ni is slightly more electronegative than Ga,^87^ one would formally describe **3** as bearing a Ni^2–^ center, if employing the standard IUPAC definition of oxidation states.^88^ In that case, although the observed reactivity is Ga-centered, the Ni atom effectively behaves as an electron shuttle, and thus can be seen as a redox noninnocent ‘metallo-ligand’. Calculated NPA charges rather suggest minimal change in the oxidation state at Ni on ethylene addition (**2**: 0.35; **3**: 0.48), with modest oxidation at the Ga centers (**2**: Ga1 = Ga2 = 0.392; **3:** Ga1:0.841 Ga2:0.861), in agreement with an oxidative cycloaddition at the [GaGa] unit. This reversible activation can thus be considered as a cooperative trimetallic process, wherein a redox noninnocent transition metal acts as a concealed electron reservoir in bond activation localized at main-group centers (e.g., Ga). To shed further light on the involvement of Ni in this process, additional DFT calculations were carried out to understand the mechanism involved in the ethylene activation promoted by **2** (Figure 5). It is reasonable to assume that the reaction begins with the coordination of ethylene to the transition metal. However, our calculations indicate that the formation of the corresponding complex **INT1’** is highly endergonic (Δ*G* = 24.8 kcal·mol^–1^), which is likely due to the steric hindrance in the initial species **2** which hampers the coordination of ethylene. Instead, we found that **2** can be transformed into the more stable intermediate **INT1** through transition state **TS1**, a saddle point associated with the cooperative activation of ethylene across one of the Ni–Ga bonds of **2**, with a feasible barrier of 28.5 kcal·mol^–1^. Intermediate **INT1** then evolves into the observed complex **3** in a highly exergonic reaction Δ*G* = −19.7 kcal·mol^–1^) via **TS2**, a transition state associated with the migration of the CH_2_ moiety from nickel to the adjacent gallium atom, with a low barrier of 8.3 kcal·mol^–1^. Therefore, our calculations support the cooperative action of the [Ga_2_Ni] core in **2** in the activation of ethylene. Moreover, the reversible nature of the process is confirmed by its slight endergonicity (Δ*G* = 2.2 kcal·mol^–1^) and the computed reverse barrier (Δ*G* ^*‡*^ = 28.0 kcal·mol^–1^), which nicely matches the experimentally determined value of 29.2 kcal·mol^–1^.

**Figure 5:**
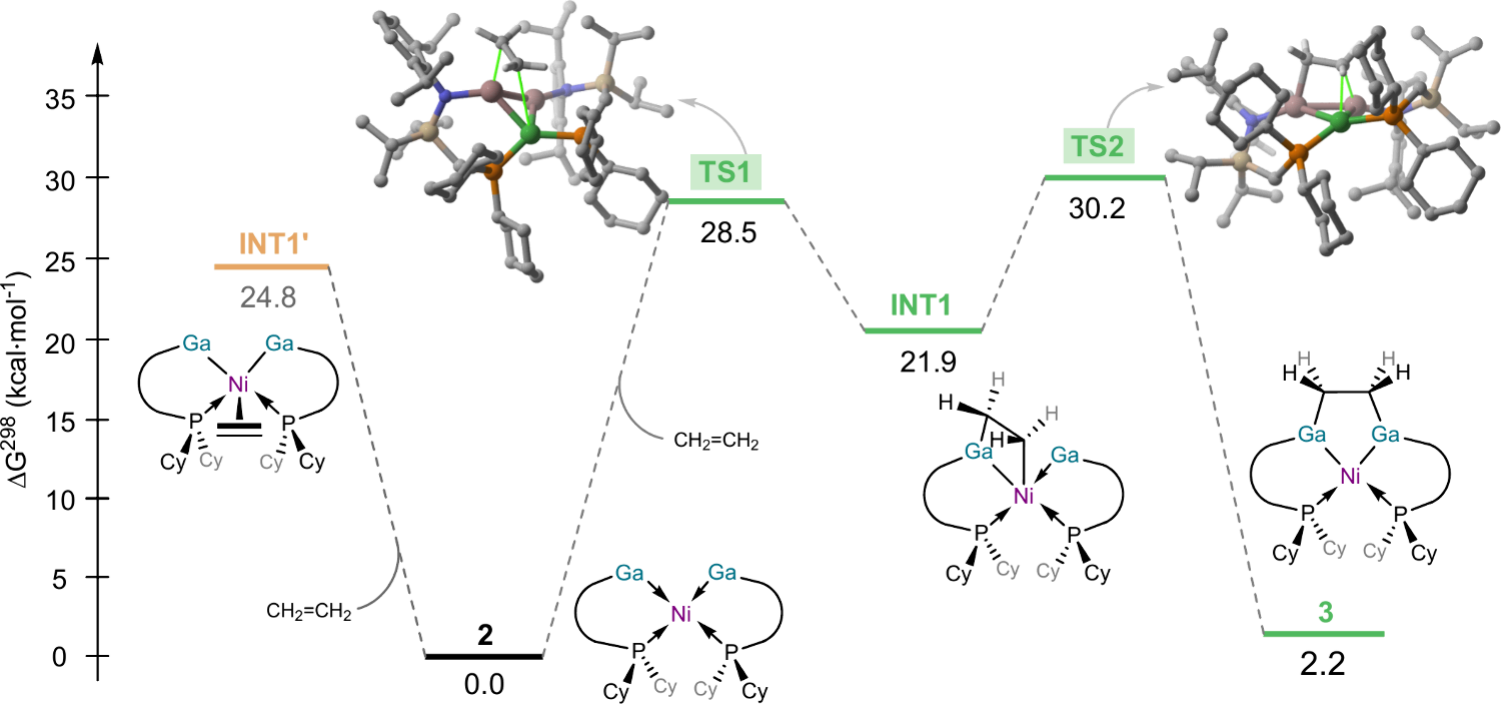
Computed reaction profile for the ethylene activation promoted by **2** and leading to complex **3**. Relative free energies (Δ*G*, at 298 K) are given in kcal·mol^–1^. All data have been computed at the (RI)-BP86-D3BJ/def2-SVP level (C–H hydrogen atoms in the transition states were omitted for clarity).

While compound **2** can reversibly activate ethylene under nonforcing conditions, we also sought to uncover further transformations of the bridging ethylene group in complex **3**, which can be seen as a highly activated alkene. Quite remarkably, exposure of a *d*_8_-THF solution of ethyl complex **3** to 1 atm of dihydrogen pressure led to a color change from red-orange to yellow-orange, concomitant with the clean formation of a single new species, **4**, within minutes. In the ^1^H NMR spectrum a new high-field shifted doublet is found at δ = −8.13 ppm (^3^ *J*_HP_ = 7.4 Hz), integrating to 2H, which is consistent with the formation of a nickel dihydride complex. Additionally, the resonance associated with the C_2_H_4_ ligand in **3** is now split into two distinct multiplets (δ = 0.10 and −0.57), indicating a break in symmetry upon H_2_ activation (*viz*. Figure 4(a)); the former resonance overlaps with one aliphatic ligand signal, lending this resonance a total integration of 3H. X-ray structural analysis, together with the described NMR data, confirmed the identity of **4** as a trimetallic nickel dihydride complex, in which each hydride ligand bridges one Ga–Ni bond, and the 1,2-bis­(gallyl)­ethane unit is retained (Figure 4(d)) ([Fn fn1]). Structurally, complex **4** is remarkably similar to **3**, as evidenced by nearly identical bond lengths and angles around the core metal centers. One would thus expect a small energetic barrier toward H_2_ activation here, given that essentially no structural rearrangement is required. To confirm this hypothesis, the dihydrogen activation reaction mediated by **3** was computationally explored (Figure 6). Our calculations suggest that the process begins with the coordination of H_2_ to the transition metal in forming intermediate **3-H**_**2**_. This step is slightly endergonic (Δ*G* = 4.9 kcal·mol^–1^) mainly due to entropic reasons (in fact, the reaction becomes exothermic, Δ*H* = – 3.8 kcal·mol^–1^ when entropy is not considered). From **3-H**_**2**_, species **4** can be formed directly through transition state **TS3** in a highly exergonic reaction (Δ*G* = – 12.8 kcal·mol^–1^), involving a low barrier of only 7.7 kcal·mol^–1^. Alternatively, we found that **3-H**_**2**_ can also be transformed into **4’**, the symmetric isomer of **4**, with a comparable activation barrier (via **TS3′**) but in a much less exergonic transformation (Δ*G* = – 2.5 kcal·mol^–1^). This species can then be transformed into the observed isomer **4** via **TS4’**, again with a relatively low barrier (Δ*G* ^*‡*^ = 7.8 kcal·mol^–1^). In any case, either directly or stepwise, our calculations support the observed ease of H_2_ activation by **3**.

**Figure 6:**
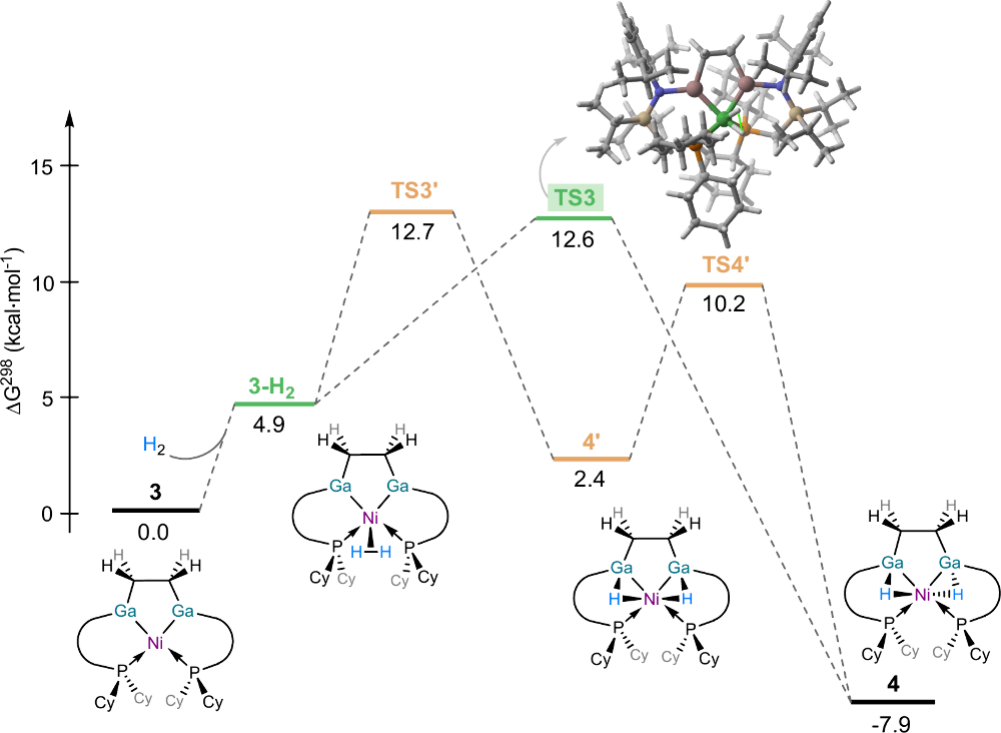
Computed reaction profiles for the dihydrogen activation mediated by **3** and leading to complex **4**. Relative free energies (Δ*G*, at 298 K) are given in kcal·mol^–1^. All data have been computed at the (RI)-BP86-D3BJ/def2-SVP level.

To further elucidate the described dihydrogen activation process in forming **4**, isotopic labeling experiments utilizing D_2_ were conducted. One would expect that the doublet corresponding to the bridging hydrides arising from H_2_ activation (i.e., δ = −8.13 ppm) would be absent in the ^1^H NMR spectrum of **4**-**D**. However, a resonance is maintained at this position, integrating to 0.5H, while one resonance assigned to the C_2_H_4_ moiety (and an overlapping ligand signal) now integrates to only 2.5H (i.e., as opposed to 3H in the ^1^H NMR spectrum of **4** (Figure 7)). This is indicative of facile H/D exchange between the bridging hydride ligands and the C_2_H_4_ moiety, and thus a low energetic barrier to both C–H formation and C–H scission, giving spectroscopic evidence for the reversible reductive elimination of one Ga-CH_2_–*CH*_3_ unit. The rapid nature of this process, which equilibrates in seconds, further supports a negligible barrier to this C–H activation. Further, addition of D_2_ to the hydride complex **4** leads to formation of a similar ratio of **4** and **4-D**, indicative of a dynamic H_2_ (and D_2_) actvation process in this complex.

**Figure 7:**
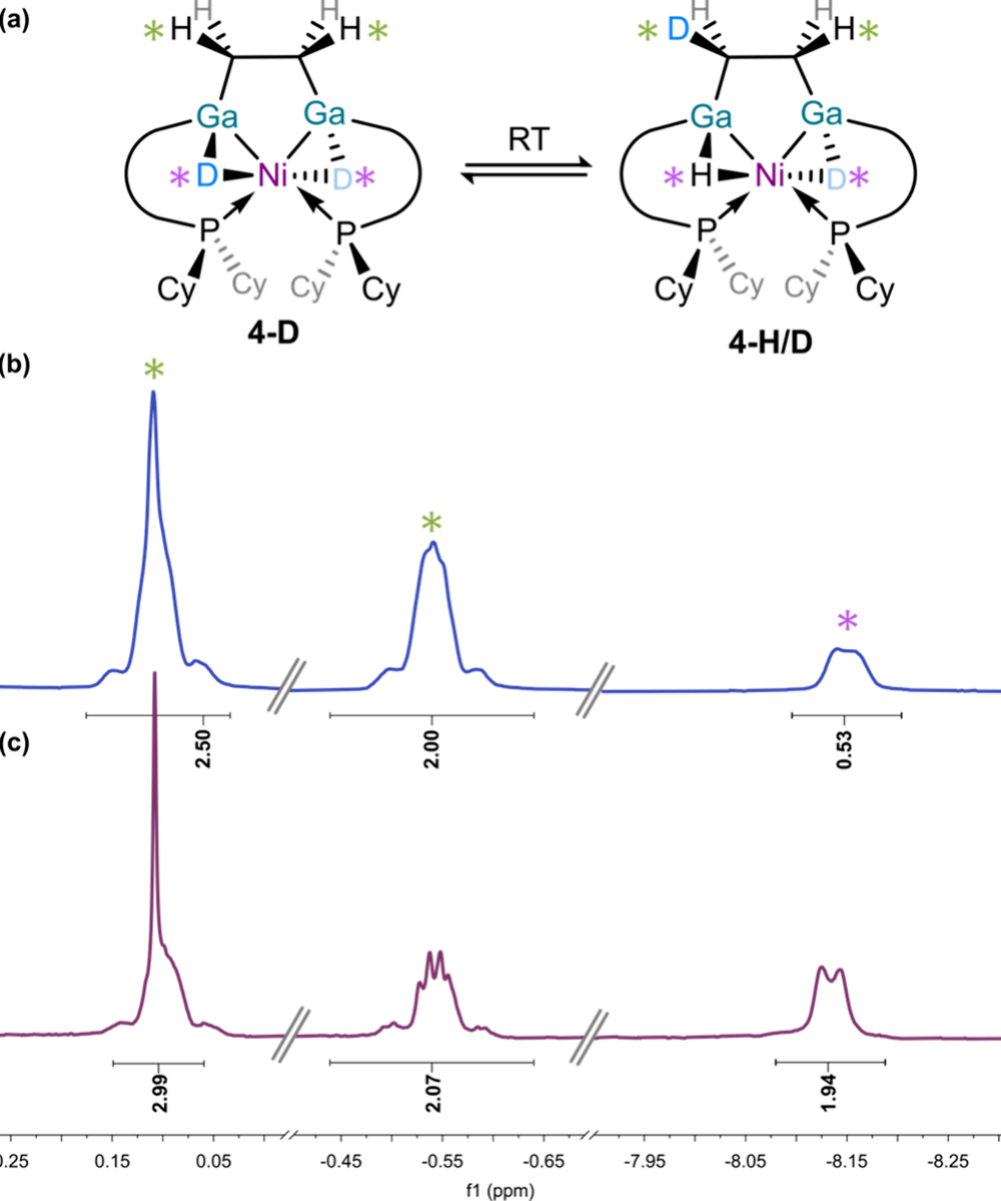
(a) Scheme demonstrating the proposed H/D scrambling in **4-D**; (b) selected regions of the ^1^H NMR spectrum of freshly prepared **4-D**, showing the resonances associated with the C_2_H_4_ (*) moiety, and the bridging hydrides/deuterides (*) in **4-D**; (c) comparative regions of the ^1^H NMR spectrum of **4**, indicating the expected integration of those peaks.

Despite this low barrier to an initial C–H bond elimination, we are surprised to find that complete elimination of the combined C_2_H_6_ fragment does not rapidly proceed at ambient temperature, suggesting that the final Ga–C bond scission by hydrogen transfer is kinetically disfavored under these conditions. Still, after 7 days, a *d*_8_-THF solution of **4** left at room temperature results in full conversion to **2**, under liberation of ethane. This process is accelerated by heating a solution of **4** to 50 °C for 6 h (Figures S37–S39 in Supporting Information) - this therefore allows for more detailed insights into the thermodynamics of this process via an Eyring analysis, which was performed utilizing both **4** and **4**-**D**. For **4**, a large negative Δ*S* ^*‡*^ value of −58.59 calmol^–1^K^–1^ is found, with a Δ*G*_*298*_^*‡*^ value of 28.43 kcal·mol^–1^ in keeping with a low reaction rate at ambient temperatures. Labeling with deuterium, i.e., in **4-D**, gives a primary inverse kinetic isotope effect of 0.35, which aligns with C–H/D bond formation, i.e., reductive elimination, being key in the rate-determining step. In addition, the highly disfavored entropic nature of the reaction aligns with a highly ordered transition state in leading to this rate-determining elimination; this is perhaps not surprising given the multicentered nature of trimetallic **4**, and indeed the requirement of forming two new C–H bonds. These kinetic data combined with the above-described H/D scrambling process would indicate that the final C–H bond forming process is rate-limiting. This is supported when observing the calculated mechanism for the final C–H elimination reaction: formal reductive elimination from intermediary [HGa-Ni-GaEt] (*viz*. INT2, Figure S125) is exergonic (Δ*G* = −11.2 kcal·mol^–1^), with a calculated barrier of 29.7 kcal·mol^–1^, matching the experimental value of 28.43 kcal·mol^–1^. As a whole, this well-defined ethane elimination process, in combination with the isolation of compounds **3** and **4**, represents a rare, if not unique example of a single system achieving two distinct oxidative chemical processes, i.e., alkene addition and H–H bond scission. The remarkably selective nature of these processes is also demonstrated by achieveing the stepwise formation of **4** in a single NMR, through sequential addition of C_2_H_4_ and H_2_ to a solution of **2** (Figures S41 and S42 in Supporting Information); observation of distinct oxidative processes is only feasible due to the presence of three redox-active metal centers. As a whole, this demonstrates a full alkene hydrogenation cycle with well-defined snapshots of each intermediate species arising from consecutive substrate activation and ultimately alkane elimination (Figure 8), aided by the confined spatial arrangement of the three metal centers involved.

**Figure 8:**
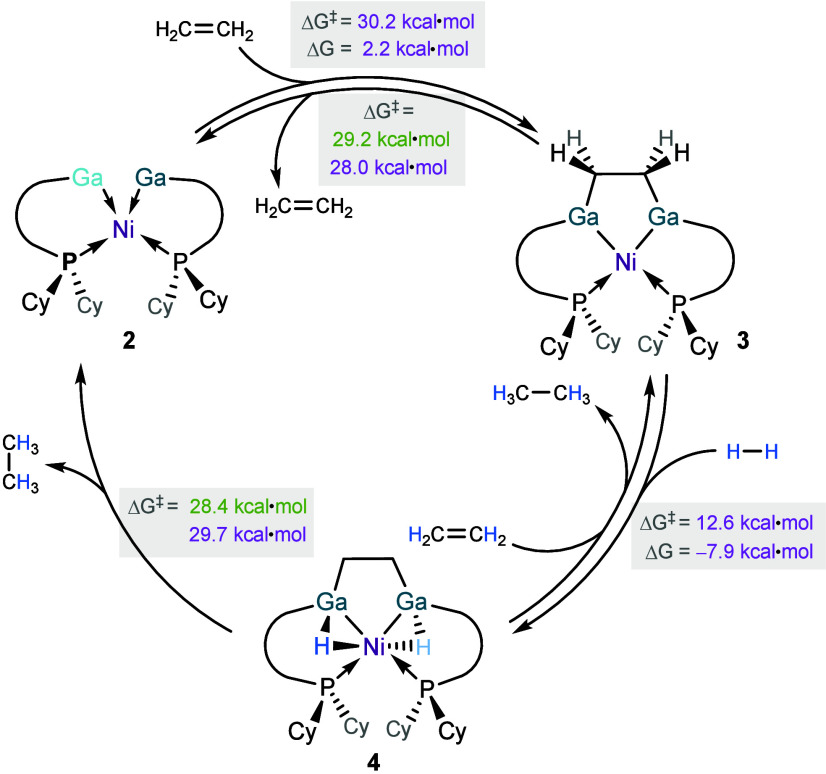
Experimentally elucidated cycle for sequential alkene addition, dihydrogen addition, and alkane elimination in complex **2**. Experimentally derived Δ*G* ^*‡*^ values are shown in green; computationally derived Δ*G* and Δ*G* ^*‡*^ (at 298 K) were computed at the (RI)-BP86-D3BJ/def2-SVP level, and are shown in purple.

### Alkene Hydrogenation Catalyzed by Trimetallic **2**

Building on the above established trimetallic hydrogenation cycle for ethylene, we next investigated the broader catalytic utility of **2** in the hydrogenation of unactivated alkenes, under mild conditions. Cyclopentene was chosen as a model substrate owing to its moderate steric demand and lessened volatility, relative to ethylene. Although no stoichiometric reaction is observed between **2** and cyclopentene, pressurizing this mixture with 1.5 atm of dihydrogen led to clean conversion to cyclopentane ([Fn fn2]). To monitor the reaction in ‘real time’, and to optimize conditions, hydrogen uptake was measured using a gas buret,^89^ allowing one to monitor pressure decrease over time. From these experiments, we find that 2 mol % of **2** in toluene can fully hydrogenate cyclopentene at room temperature within 40 min at 3 atm of dihydrogen pressure. Moving to catalyst loadings below 0.01 mol % yielded significant turnover numbers of 10,000 under optimized conditions, albeit with reaction times reaching 50 h (i.e., turnover frequency = 200 h^–1^). Notably, the parent synthons, ^Cy^LGaH_2_ and Ni­(cod)_2_,^82^ are not effective catalysts when employed alone, and mercury addition does not affect catalysis, confirming trimetallic species **2** as the active catalytic system. To probe the kinetics of the hydrogenation process, an Eyring analysis was conducted using cyclopentene as the substrate, for both H_2_ and D_2_ (Table S8 in Supporting Information). These data confirm a process which is favorable at room temperature, with a feasible reaction barrier (Δ*G*_*298*_^*‡*^ = 20.8 kcal·mol^–1^), but a highly disfavored entropic factor (Δ*S* ^*‡*^ = −52.9 cal·mol^–1^·K^–1^), which we believe arises from the multicenterd nature of the catalyst and the constraining nature imposed by the ligand environment. Isotopic labeling yields a primary inverse KIE of 0.80, in keeping with values attained for stoichiometric ethane elimination from **4**, and suggesting that C–H bond elimination is rate-determining.

Encouraged by these results, we next examined the substrate scope of **2** in alkene hydrogenation (Figure 9), utilizing a multiwell high-pressure reactor (see Figure S75 in Supporting Information). Sterically unhindered mono- and disubstituted aliphatic alkenes, e.g., 1-hexene, vinyl-trimethylsilane, and *cis*-3-octene, are quantitatively hydrogenated within 5 h by 1 mol % of **2** at 5 atm of dihydrogen pressure. These conditions proved effective for cyclic substrates, including cyclopentene and cycloheptene. Steric hindrance was found to significantly affect catalytic efficiency, e.g., with allyl-cyclohexane showing only 15% conversion to propyl-cyclohexane under the same conditions. This was further confirmed by styrene derivatives, e.g., with a significant decrease in turnover on moving from α-methylstyrene (78%) to 1,1-diphenylethylene (<5%), despite the latter being electronically more susceptible to hydrogenation. This was further demonstrated when screening a range of aryl-substituted styrene derivatives: e.g., *para*-trifluoromethyl-styrene, though more electronically activated than *p*-fluoro-styrene, demonstrates lessened rates of hydrogenation (i.e., 59% vs. 78% @ 2h, 60 °C, respectively). Notably, rates for this collection of substrates do not follow the respective Hammett parameters – this suggests that sterics play a strong role in turnover rate. We attribute this steric sensitivity to the sterically encumbered ligand environment around the trimetallic core of **2**, leading to a small binding pocket which likely limits substrate accessibility. Nevetheless, that catalysis efficiently proceeds for the above-described styrene derivatives, featuring C–Cl, C–F, C–O, and C=O bonds, is indicative of good functional group tolerance for the developed trimetallic catalyst, though this apparently does not tolerate the nitril functional group.

**Figure 9:**
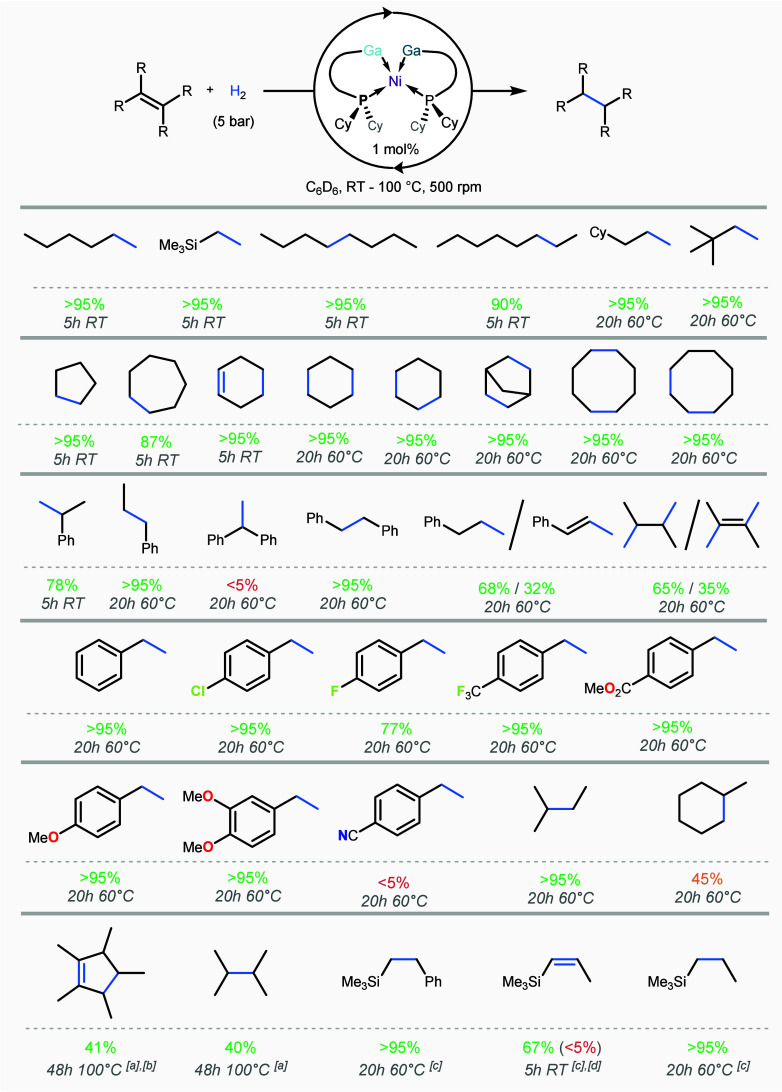
Scope of catalytic alkene hydrogenation using trimetallic complex **2**. ^[a]^Catalysis was performed with 1.5 mol % of **2** as catalyst. ^[b]^14% full hydrogenation is observed, forming pentamethylcyclohexane. ^[c]^For these examples, the alkyne was used. ^[d]^The value in parentheses indicates the percentage formation of the *E*-isomer.

Given the above observations, for more sterically demanding alkenes, including di-, tri-, and tetra-substituted derivatives, hydrogenation was also carried out at 60 °C. Under these conditions a variety of mono- and disubstituted alkenes could be hydrogenated in near quantitative yield, including styrene derivatives such as stilbene, *trans*-β-methyl-styrene, and conjugated systems, in addition to substrates that were not fully hydrogenated at ambient temperature (e.g., allyl cyclohexene). We find that even 3-methylbut-2-ene, despite being a trisubstituted alkene, can be hydrogenated to 3-methylbutane in >95% yield at 60 °C within 20h, demonstrating a powerful hydrogenation capacity for less sterically prolific alkenes. To probe the limits of the hydrogenation capability of **2**, tetrasubstituted alkenes were utilized. Here more forcing conditions were used (i.e., 100 °C, 1.5 mol % catalyst). Under these conditions, Cp*H could be converted to the cyclopentene derivative, pentamethylcyclopentene, in 41% yield, and the challenging 2,3-dimethylbut-2-ene is converted to the corresponding alkane in 40% yield. Further, under these conditions no metal deposition is observed, demonstrating the stability of the catalyst, which maintains hydrogenation performance at such elevated temperatures.

We note that, while **2** compares favorably to earlier reported homogeneous 3*d* metal hydrogenation catalysts,^90–95^ there is some way to go in reaching the efficiency and substrate scope of state-of-the-art systems. Still, trimetallic **2** displays a higher proficiency when compared to our reported bimetallic E-Ni^0^ systems (E = Ge^II^, Sn^II^, Ga^I^),^82,76^ a promising observation highlighting the amplified reactivity of the described cooperative trimetallic platform.

## Conclusion

This work introduces the concept of spatially confined trimetallic cooperativity, through the synthesis of a bis­(gallylene)-Ni^0^ complex (**2**), which features a spatially constrained [Ga_2_Ni] core. The cooperative interplay between all three metals is evidenced by the isolation of species arising from sequential ethylene (**3**) and dihydrogen (**4**) activation, both of which represent well-defined snapshots of intermediates on the catalytic alkene hydrogenation cycle. Together with DFT calculations, kinetic analyses, and isotopic labeling experiments, these findings provide unprecedented mechanistic insights into the efficacy of multimetallic cooperativity in promoting small-molecule activation and ultimately catalysis. This also establishes a guiding design principle in accessing well-defined multimetallic systems, in utilizing chelating, redox noninnocent metallo-ligands which can achieve reactivity modes inaccessible to monometallic systems. Thus, these insights may enable the rational design of a broader family of cooperative multimetallic systems, transitioning from serendipitous discovery to predictive higher-order catalyst development.

## Supplementary Material



## Footnotes

^a^
We note that the ATR-IR spectrum of **4** does not contain any significant peaks relating to the Ni–H–Ga stretching frequency. This aligns with a very low, almost neglible intensity of the predicted stretching frequency of 2 kMmol^–1^. See Figures S26, S33, and S34 in the Supporting Information.

^b^
The observed lack of stoichiometric reactivity between **2** and cyclopentene on the NMR time scale suggests that a possible cycloaddition product akin to **3** is higher in energy than **2** for this alkene, presumably due to steric pressure. This infers that, under an H_2_ atmosphere, low concentrations of the presumed cycloadduct of **2** and cyclopentene are turned over. That is, complex **2** is the catalyst resting state.

^c^
Distances taken from a survey of the CCDC, using dimeric Ga–Ga bonded species with 3-coordinate Ga centers.

## Notes

Synthetic and characterization data for all new compounds, including spectra; details of kinetic and thermodynamic studies; and full details and references for the crystallographic and computational data (PDF)
