# Supplementary material for: Snapshots of Cooperative Trimetallic Alkene Hydrogenation
Source: J Am Chem Soc. 2025 Dec 5;147(50):46373–83. doi: 10.1021/jacs.5c15492 (PMC12715791; doi:10.1021/jacs.5c15492)
Supplement: Supplementary file 1 [file ja5c15492_si_001.pdf]

Supplementary Information

Snapshots of Cooperative Trimetallic Alkene Hydrogenation

Till L. Kalkuhl,[a] Israel Fernández,[b] and Terrance J. Hadlington*,[a]

[a] Fakultät für Chemie, School of Natural Sciences, TU München, Lichtenberg Strasse 4, 85749 Garching, Germany

Email: terrance.hadlington@tum.de

[b]Departamento de Química Orgánica I, Facultad de Ciencias Químicas and Centro de Innovación en Química Avanzada, Universidad Complutense de Madrid, 28040-Madrid, Spain

Table of Contents

1. General Considerations 1
2. Experimental Procedures 3
3. Printed spectra for novel compounds 8
4. Reversibility of Ethylene activation by 2 25
5. Ethane liberation from 4, regenerating 2 27
6. Transformation of 4 regenerating 3 29
7. Consecutive transformation from 2 to 3 to 4 30
8. Eyring-Polanyi Analysis of Ethylene Elimination from 3 32
9. Eyring-Polanyi Analysis of Ethane Elimination of 4/4-D 38
10. Eyring-Polanyi Analysis of Hydrogenation catalysis utilising 2 47
11. Determination of the maximum turnover number 56
12. Catalytic hydrogenation of alkenes and alkynes 57
13. Substrate Scope of alkene/alkyne hydrogenation catalysis 58
14. Catalyst poisoning tests 60
15. Analytical data for alkene/ alkyne hydrogenation catalysis 62
16. Spectroscopic data for alkene/alkyne hydrogenation catalysis 73
17. X-ray crystallographic details 97
18. Computational Details. 98
19. References 164

1. General Considerations

All analysis and manipulations were carried out under a dry oxygen free argon atmosphere using standard Schlenk techniques or in an MBraun inert atmosphere glovebox containing an atmosphere of dry high purity argon, using flame dried glassware. All syringes, magnetic stirring bars and needles were rigorously dried. THF, hexane and diethyl ether were dried by distillation over a sodium/benzophenone mixture, degassed by standard procedures and stored over activated 4Å mol sieves. C_6D_6 and THF-d_8 were dried, degassed by standard procedures, and stored over a potassium mirror, molecular sieve prior to use, respectively. All other solvents were dried over activated 4Å mol sieves and degassed by standard procedures, prior to use. GaI_3,[1] $Ni(cod)_2$,[2] ^{CyiPr}DippNK (*i.e.* ^{Cy}LK),[3] ^{CyiPr}DippNGaI_2 (*i.e.* ^{Cy}LGaI_2),[3] and ^{CyiPr}DippNGaH_2 (*i.e.* ^{Cy}LGaH_2)[3] were synthesized according to known literature procedures. All other reagents were used as received. For all experiments utilizing dihydrogen or deuterium, grade 5.0 H_2 or D_2 was used. For all experiments utilizing ethylene, grade 2.5 C_2H_4 was used. If compounds were sensitive to oxygen and/or moisture, they were stored in a glovebox (MBraun Labmaster dp) under a dry, oxygen-free high purity argon atmosphere.

NMR Spectroscopy

NMR spectra were recorded on a Bruker AV 400 Spectrometer. The spectra were processed using the MestReNova 14.3.3 software suite. All chemical shifts δ are given in ppm. The ^{1}H, and $^{13}C\{^{1}H\}$ NMR spectra were referenced to the residual solvent signals as internal standards. $^{29}Si\{^{1}H\}$ NMR spectra were externally calibrated with $SiMe_4$. $^{31}P\{^{1}H\}$ NMR spectra were externally calibrated with H_3PO_4. The coupling constants J are given in Hz. For variable temperature NMR experiments with a targeted temperature of over 298 K, the temperature inside the probe head was externally referenced *via* monitoring the OH/CH_2 peak separation in an 80 wt% glycol/DMSO-d_6 solution. For variable temperature NMR experiments with a targeted temperature of under 298 K, the temperature inside the probe head was externally referenced *via* monitoring the OH/CH_3 peak separation in a 4 wt% methanol/methanol-d_4 solution.[4] For signal multiplicities, the following abbreviations were used: s = singlet, d = doublet, t = triplet, q = quartet, p = pentet, sept= septet, m = multiplet, br = broad, and combinations thereof.

LIFDI-MS

Liquid Injection Field Desorption Ionization Mass Spectrometry (LIFDI-MS) was measured directly from an inert atmosphere glovebox with a Thermo Fisher Scientific Exactive Plus Orbitrap equipped with an ion-source from Linden CMS.[5]

IR Spectroscopy

Infrared spectra were measured with the Alpha FT-IR from Bruker containing a platinum diamond ATR device. The compounds were measured as solids in an MBraun Labmaster dp inert atmosphere glovebox containing a dry oxygen free atmosphere of high purity argon.

UV-Vis-Spectroscopy

Absorption spectra (UV-Vis) were recorded on an Agilent Cary 60 UV/Vis spectrophotometer. Samples were prepared and transferred to a Teflon-valved cuvette with in an MBraun Labmaster dp inert atmosphere glovebox containing a dry oxygen free atmosphere of high purity argon.

Elemental Analysis

Elemental analyses (C, H, N) were performed with a combustion analyzer (elementar vario EL, Bruker).

2. Experimental Procedures

$[(^{Cy}LGa)_2\cdot Ni]$, 2.

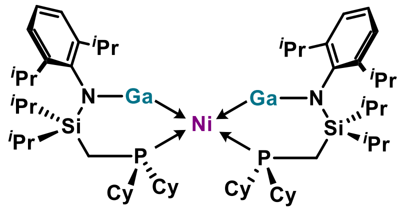

A Schlenk flask was loaded with $^{Cy}LGaH_2$ (1.000 g, 1.750 mmol) and $Ni(cod)_2$ (0.240 g, 0.873 mmol). The flask was cooled to -80 °C, and toluene (20 mL) was added with rapid stirring. The reaction mixture was stirred for 30 minutes at -80 °C, and subsequently allowed to warm to ambient temperature over the course of 16h. The reaction mixture was then filtered, and all volatiles were removed from the filtrate *in vacuo*, yielding a dark red solid. In order to completely remove all high boiling side-products (cyclooctene, cyclooctane), the reaction flask is heated gently to ~50 °C at a pressure of ~10^{-2} mbar for 1 hr. The solid residue was then washed with pentane (10 mL) and dried *in vacuo*, yielding analytically pure **2** (0.502 g, 0.418 mmol) as a dark red powder. Storage of the pentane washing solution, which had been concentrated to 5 mL, led to a crop of dark red crystals suitable for single crystal X-Ray diffraction analysis after storage overnight at ambient temperature. These were isolated by filtration and dried *in vacuo*, yielding **2** (0.317 g, 0.264 mmol). Combined yield of **2**: 0.819 g (0.682 mmol, 78%).

^{1}H NMR (C_6D_6, 400 MHz, 298 K): δ = 7.23 (m, 4H, $Dipp^{m}$-C*H*), 7.11 (t, $^3J_{HH}$ = 7.6 Hz, 2H, $Dipp^{P}$-C*H*), 4.02 (p, $^3J_{HH}$ = 6.7 Hz, 2H, N-Dipp-iPr-C*H*), 3.86 (p, $^3J_{HH}$ = 6.9 Hz, 2H, N-Dipp-iPr-C*H*), 2.50 (m, 2H, Cy-C*H*), 2.29 – 2.21 (m, 2H, Cy-C*H*), 2.14 – 2.07 (m, 2H), 2.06 – 1.95 (m, 3H, Ali-C*H*), 1.95 – 1.84 (m, 8H, Ali-C*H*), 1.78 (m, 7H, Ali-C*H*), 1.70 (s, 3H, Ali-C*H*), 1.65 – 1.50 (m, 9H, Ali-C*H*), 1.47 (m, 11H, Si-iPr-CH_3/ N-Dipp-iPr-CH_3), 1.43 (m, 9H, N-Dipp-iPr-CH_3), 1.40 (d, *J* = 7.0 Hz, 6H, N-Dipp-iPr-CH_3), 1.38 – 1.33 (m, 3H, Ali-C*H*), 1.32 – 1.23 (m, 11H, Ali-C*H*), 1.19 (s, 12H, Si-iPr-CH_3), 1.16 (d, $^3J_{HH}$ = 7.1 Hz, 2H, Ali-C*H*), 1.11 (d, $^3J_{HH}$ = 7.2 Hz, 7H, Ali-C*H*), 0.96 (d, $^3J_{HH}$ = 6.6 Hz, 8H, Ali-C*H*), 0.67 (d, $^3J_{HP}$ = 7.1 Hz, 4H, Si-CH_2-P).

^{13}C NMR (C_6D_6, 101 MHz, 298 K): δ = 145.94 (N-Dipp-$^{ipso/O}$CH), 145.43 (N-Dipp-$^{ipso/O}$CH), 140.75 (N-Dipp-$^{ipso/O}$CH), 123.42 (N-Dipp-$^{m/p}$CH), 123.31 (N-Dipp-$^{m/p}$CH), 123.00(N-Dipp-$^{m/p}$CH), 40.74- 40.55 (m, Cy-C*H*), 39.57-39.44 (m, Cy-C*H*), 33.41-33.37

(m, Cy-C*H*), 30.55 (Cy-C*H*), 30.06 (Cy-C*H*), 30.01(Cy-C*H*), 29.97 (Si-iPr-CH_3), 29.00 (d, $^2J_{PC}$ = 6.6 Hz, Cy-C*H*), 28.41 (N-Dipp-iPr-CH_3), 27.99 (N-Dipp-iPr-*C*H), 27.94, 27.86, 27.79, 27.04, 26.99 (N-Dipp-iPr-*C*H), 26.92, 26.52, 26.27, 23.73, 22.75 (Si-iPr-CH_3), 22.56 (N-Dipp-iPr-CH_3), 19.93, 19.89, 19.84, 18.81, 18.78, 18.28, 15.30, 15.27, 14.74, 13.92, 7.83 (d, $^2J_{PC}$ = 8.8 Hz, Si-CH_2-P).

^{31}P{^{1}H} NMR (C_6D_6, 162 MHz, 298 K): δ = 32.2 (s, CH_2-*P*-(Cy*)*$_2$).

^{29}Si NMR (C_6D_6, 99 MHz, C_6D_6, 298 K): δ = -1.9 (s, CH_2-*Si*-(^{i}Pr*)*$_2$).

MS/LIFDI-HRMS found (calcd.) m/z: 1198.5431 (1198.5539) for [(^{CyiP}DippNGa)$_2$Ni]$^+$.

λ$_{max}$, nm (*ε*, Lmol^{-1} cm^{-1}): 442 (3727), 327 (9674), 280 (15621).

IR v/cm^{-1} (ATR): *no peaks found indicative of a Ga-H or Ni-H stretching mode.*

Anal. calcd. $C_{62}H_{110}Ga_2N_2NiP_2Si_2$: C, 62.07%; H, 9.24%; N, 2.33%; found: C, 63.54%; H, 8.98%;df N, 2.37%.

[{^{Cy}LGa-(*μ*-C_2H_4)-Ga^{Cy}L}·Ni], **3.**

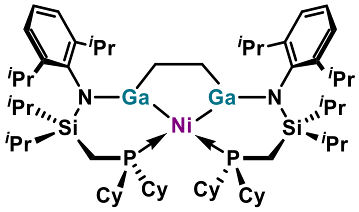

Method A: A gas tight, thick-walled Schlenk tube fitted with a Teflon valve was loaded with **2** (0.100 g, 0.083mmol), and the solid dissolved in benzene (1 mL). The atmosphere of the flask was subsequently exchanged for ethylene gas (2.5 grade, 1.5 bar) through a single freeze-pump-thaw cycle. The reaction was then stirred for 30 minutes at room temperature, and subsequently degassed by three freeze-pump-thaw cycles. The resulting solution was stored at room temperature overnight, leading to the formation of yellow crystals. These crystals were isolated by filtration and dried *in vacuo* yielding **3** as a yellow crystalline solid (87 mg, 0.071 mmol, 85 %).

Methode B: A Teflon-valve NMR tube was loaded with **4** (0.025 g, 0.021mmol), and the solid dissolved in THF-d_8 (0.35 mL). The atmosphere of the tube was subsequently exchanged for ethylene gas (2.5 grade, 1.5 bar) through a single freeze-pump-thaw cycle. The reaction was then shaken for 30 minutes at room temperature, and subsequently degassed by three freeze-pump-thaw cycles.

^{1}H NMR (C_6D_6, 400 MHz, 298 K): δ = 7.17 (m, 3H, Dipp^{m}C*H*), 7.14 – 7.09 (m, 3H, Dipp^P-C*H*), 3.78 (p, $^3J_{HH}$ = 6.8 Hz, 4H, N-Dipp-iPr-C*H*), 2.26 - 2.17 (m, 8H, Cy-C*H*), 1.91 – 1.81 (m, 10H, Cy-C*H*), 1.70 -1.67 (m, 5H, Ali-C*H*), 1.52 - 1.48 (m, 7H, Ali-C*H*), 1.42 (d, $^3J_{HH}$ = 6.8 Hz, 12H, iPr-CH_3), 1.39 – 1.32 (m, 12H, Cy-C*H*/ Ali-C*H*), 1.30 – 1.28 (m, 15H, iPr-CH_3/ Ali-C*H*), 1.24 (m, 3H, Ali-C*H*), 1.19 (d, $^3J_{HH}$ = 6.8 Hz, 14H, iPr-CH_3/ Cy-C*H*/ Ali-C*H*), 1.03 (d, $^3J_{HH}$ = 6.8 Hz, 10H, iPr-CH_{3ff}), 0.34 (s, 4H, Ga-C_2H_4-Ga).

^{13}C NMR (C_6D_6, 101 MHz, 298 K): δ = 146.42 (Dipp-$^{ipso/O}$CH_3), 144.4 (Dipp-$^{ipso/O}$CH_3), 123.5 (Dipp-^{m}CH_3), 123.0 (Dipp-^{p}CH_3), 43.1 (d, $^1J_{PC}$ = 14.8 Hz, Cy-C*H*), 30.78 (Cy-*C*H), 30.51 (Cy-*C*H), 27.86 (Cy-*C*H), 27.76 (iPr-*C*H_3), 27.65 (Ali-*C*H), 27.63 (N-Dipp-iPr-*C*H), 26.48 (Cy-*C*H), 26.42 (iPr-*C*H_3), 23.72 (iPr-*C*H_3), 19.98 (Ali-*C*H), 19.03 (Ali-*C*H), 15.38 (Ali-*C*H), 15.35 (Ali-*C*H), 14.61, 14.6 (d, $^3J_{PC}$ = 3.7 Hz, Ga-C_2H_4-Ga), 14.4 (d, $^3J_{PC}$ = 3.7 Hz, Ga-C_2H_4-Ga), 3.8 (t, $^2J_{PC}$ = 5.0 Hz, Si-*C*H_2-P).

^{31}P{^{1}H} NMR (C_6D_6, 162 MHz, 298 K): δ = 18.1 (s, CH_2-*P*-(Cy)$_2$).

^{29}Si NMR (C_6D_6, 400 MHz, C_6D_6, 298 K): δ = -0.1 (s, CH_2-*Si*-(^{i}Pr)$_2$).

MS/LIFDI-HRMS: The same LIFDI-MS pattern as for **2** was found, due to the analytic set-up, where the sample is heated on the emitter and thereby eliminating ethylene and regenerating **2**.

λ$_{max}$, nm (*ε*, $Lmol^{-1}cm^{-1}$): 514 (12460), 357 (40118), 288 (131772).

Anal. calcd. $C_{64}H_{114}Ga_2N_2NiP_2Si_2$: C, 62.60%; H, 9.36%; N, 2.28%; found: C, 62.72%; H, 9.23%; N, 2.30%.

[{^{Cy}LGa-(*μ*-C_2H_4)-Ga^{Cy}L}·Ni-H_2], **4.**

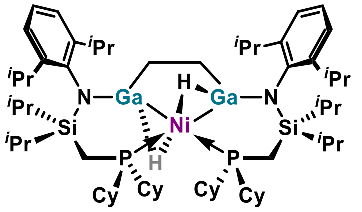

A gas tight, thick-walled Schlenk tube fitted with a Teflon valve was loaded with **1** (0.100 g, 0.083mmol), and the solid dissolved in benzene (1 mL). The atmosphere of the flask was subsequently exchanged for ethylene gas (2.5 grade, 1.5 bar) through a single freeze-pump-thaw cycle. The reaction was then shaken for 10 minutes at room temperature. Subsequently, the reaction mixture was degassed by one freeze-pump-thaw cycle, and pressurised with H_2 (5.0 grade, 1.5 bar). Storage of this reaction

mixture at room temperature overnight led to the formation of large yellow crystals, which were isolated by filtration and dried *in vacuo* yielding **4** as an orange-yellow crystalline solid (93 mg, 0.081 mmol, 93 %).

^{1}H NMR (THF-d_8, 400 MHz, 298 K): δ = 6.99 - 6.96 (m, 4H, Dipp^{Ar}C*H*), 6.88 (t, $^{3}J_{HH}$ = 7.5 Hz, 2H, Dipp^{Ar}C*H*), 3.73 (p, $^{3}J_{HH}$ = 6.7 Hz, 2H, N-Dipp-iPr-C*H*), 3.50 (p, $^{3}J_{HH}$ = 6.9 Hz, 2H, N-Dipp-iPr-C*H*), 2.19 (dd, $^{3}J_{HH}$/$^{3}J_{HP}$ = 7.9 Hz, 10.4 Hz , 5H, Cy-C*H*), 1.92 (d, $^{3}J_{HH}$ = 10.4 Hz, 7H, Cy-C*H*), 1.87 - 1.76 (m, 9H, Cy-C*H*), 1.61 - 1.46 (m, 9H, Cy-C*H*), 1.45 -1.40 (m, 4H, Ali-C*H*), 1.38 - 137 (m, 5H, Ali-C*H*), 1.35 – 1.26 (m, 6H, Cy-C*H*), 1.21 (dd, $^{3}J_{HH}$ = 6.9, 2.5 Hz, 20H, iPr-CH_3/ Cy-C*H*/ Ali-C*H*), 1.14 (d, $^{3}J_{HH}$ = 6.8 Hz, 7H, iPr-CH_3/ Ali-C*H*), 1.05 (m, 8H, Ali-C*H*), 0.97 (d, $^{3}J_{HH}$ = 6.8 Hz, 6H, iPr-CH_3), 0.85 (d, $^{3}J_{HH}$ = 6.7 Hz, 6H, iPr-CH_3), 0.50 (d, $^{3}J_{HP}$ = 7.0 Hz, 4H, Si-CH_2-P), 0.14 – 0.06 (m, 3H, Ga-C_2H_4-Ga/ Ali-C*H*), -0.42 – -0.71 (m, 2H, Ga-C_2H_4-Ga), -8.13 (d, *J* = 7.4 Hz, 2H, Ga-*H*-Ni).

^{13}C NMR (THF-d_8, 101 MHz, 298 K): δ = 147.54 (Dipp-$^{ipso/O}$CH$_3$), 145.88 (Dipp-$^{ipso/O}$CH$_3$), 145.43 (Dipp-$^{ipso/O}$CH$_3$), 124.41 (Dipp-$^{m/p}$CH$_3$), 124.22 (Dipp-$^{m/p}$CH$_3$), 124.03 (Dipp-$^{m/p}$CH$_3$), 123.96 (Dipp-$^{m/p}$CH$_3$), 42.54 (Cy-*C*H), 41.16 (Cy-*C*H), 31.38 (Ali-*C*H), 30.39 (Ali-*C*H), 30.14 (Ali-*C*H), 29.76 (Ali-*C*H), 29.69 (Ali-*C*H), 29.08 (Ali-*C*H), 28.83 (Ali-*C*H), 28.69 (Ali-*C*H), 28.64 (Ali-*C*H), 28.59 (N-Dipp-iPr-*C*H), 27.77 (Ali-*C*H), 27.44 (N-Dipp-iPr-*C*H), 26.59 (Ali-*C*H), 26.02 (Ali-*C*H), 25.82 (iPr-*C*H$_3$), 25.78 (Ali-*C*H), 25.62 (Ali-*C*H), 25.39 (Ali-*C*H), 25.20 (iPr-*C*H$_3$), 24.33 (iPr-*C*H$_3$), 24.13 (iPr-*C*H$_3$), 23.33 (Ali-*C*H), 23.13 (Ali-*C*H), 20.34 (Ali-*C*H), 20.18 (Ali-*C*H), 20.13 (Ali-*C*H), 19.45 (iPr-*C*H$_3$), 18.83 (Si-*C*H$_2$-P), 16.28 (Ali-*C*H), 16.18 (Ali-*C*H), 16.01 (Ali-*C*H), 15.99 (Ali-*C*H), 12.22 (Ga-C_2H_4-Ga), 7.95 (Ali-*C*H), 1.55 (Ali-*C*H).

^{31}P{^{1}H} NMR (THF-d_8, 162 MHz, 298 K): δ = 30.3 (s, CH$_2$-*P*-(Cy)$_2$).

^{29}Si{^{1}H} NMR (THF-d_8, 400 MHz, C_6D_6, 298 K): δ = -2.1 (s, CH$_2$-*Si*-(^{i}Pr)$_2$), -3.9 (s, CH$_2$-*Si*-(^{i}Pr)$_2$).

MS/LIFDI-HRMS: The same LIFDI-MS pattern as for **2** was found, due to the analytic set-up, where the sample is heated on the emitter and thereby eliminating ethane and regenerating **2**.

IR v/cm^{-1} (ATR): no peaks are observed which align with M-H stretching frequencies.

λ_{max}, nm (*ε*, Lmol^{-1} cm^{-1}): 347 (156544), 279 (418104).

Anal. calcd. $C_{64}H_{116}Ga_2N_2NiP_2Si_2$: C, 62.50%; H, 9.51%; N, 2.28%; found: C, 64.40%; H, 9.30%; N, 2.25%.

[[(^{Cy}LGa)-C_2H_4-(^{Cy}LGa)]-Ni-D_2], 4-D.

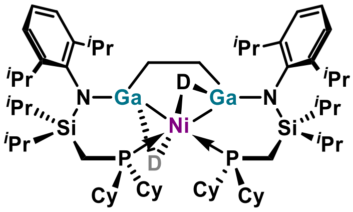

Methode A*:* **4-D** was synthesised in the same manner as **4**, using D_2 in place of H_2.

Methode B: A young type NMR tube was loaded with **4** (0.025 g, 0.021mmol), and the solid dissolved in THF-*d*$_8$ (0.35 mL). The young type NMR tube was subsequently pressurised with D_2 (5.0 grade, 1.5 bar) through a single freeze-pump-thaw cycle. The reaction was then shaken for 30 minutes at room temperature. Then the reaction mixture was degassed by three freeze-pump-thaw cycles. (*N.B.* no isolated yield was determined.)

Analytical data for **4-D** are identical to that for **4** but exhibit different signal intensities/ integer values and coupling patterns relating to Ga-H-Ni (-8.02 ppm [d, *J* = 7.4 Hz, 2H, Ga-H-Ni]) and Ga-C_2H_4-Ga (δ = 0.14 – 0.06 [m, 2.5H, Ga-C_2H_4-Ga/ Ali-CH]/ -0.42 – -0.71 [m, 2H, Ga-C_2H_4-Ga]) resonances in the ^{1}H NMR spectrum (Fig. S25). This indicates H/D scrambling and a ratio of 1/4 of Ga-H-Ni/ Ga-D-Ni and a ratio of 4/1 of Ga-C_2H_4-Ga/ Ga-C_2H_3D-Ga. In addition, in the IR spectrum isotopically shifted stretching frequencies are observed, associated to the Ga-H-Ni vibration (see below).

N.B. Pressurizing a solution of **4** with D_2 leads to H/D exchange and the observation of the same NMR-spectra as for **4-D.**

MS/LIFDI-HRMS: *The same LIFDI-MS pattern as for* **2** *was found, due to the analytic set-up, where the sample is heated on the emitter and thereby eliminating ethane and regenerating* **2***.*

IR v/cm^{-1} (ATR): discernible signals pertaining to Ni-D-Ga stretching modes could not be observed, presumably due to overlapping with the fingerprint region.

3. Printed spectra for novel compounds

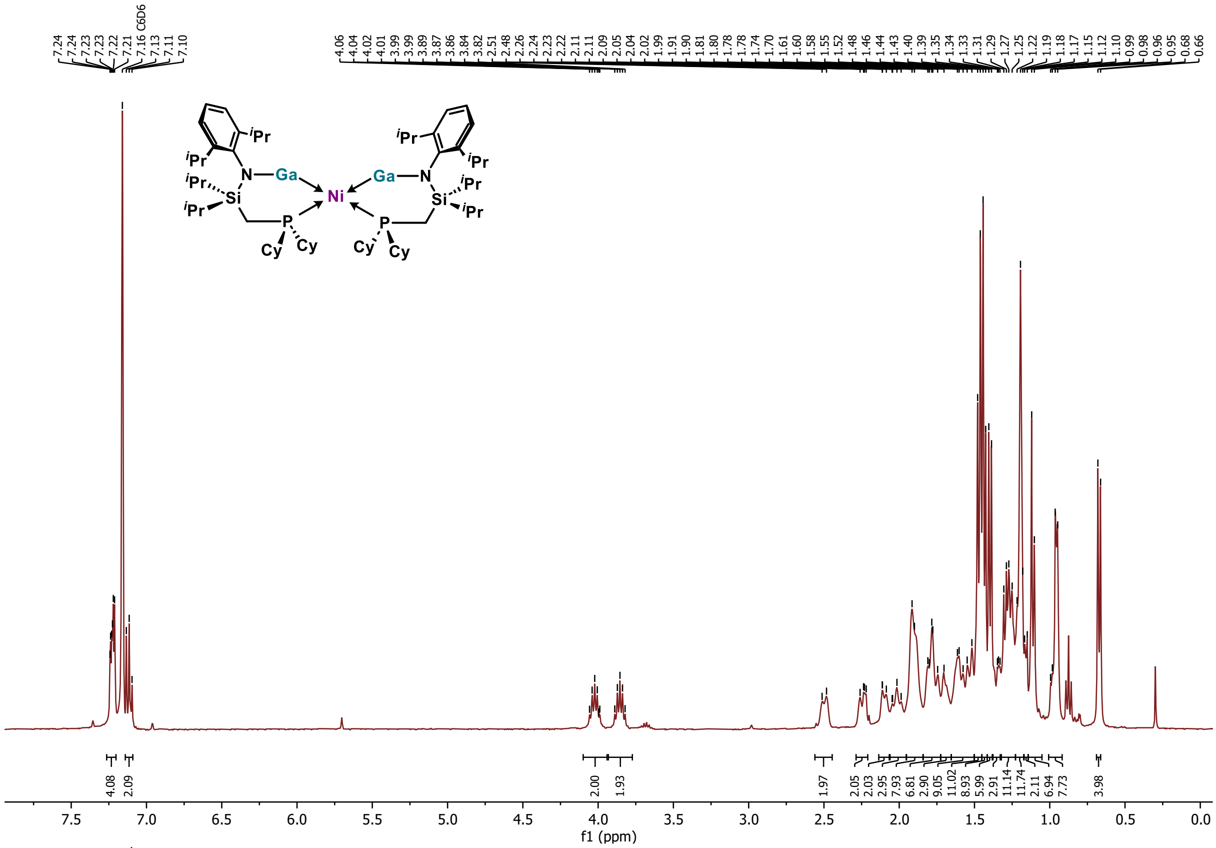

Figure S1. ^{1}H NMR spectrum (400 MHz, C_6D_6, 298 K) of **2**.

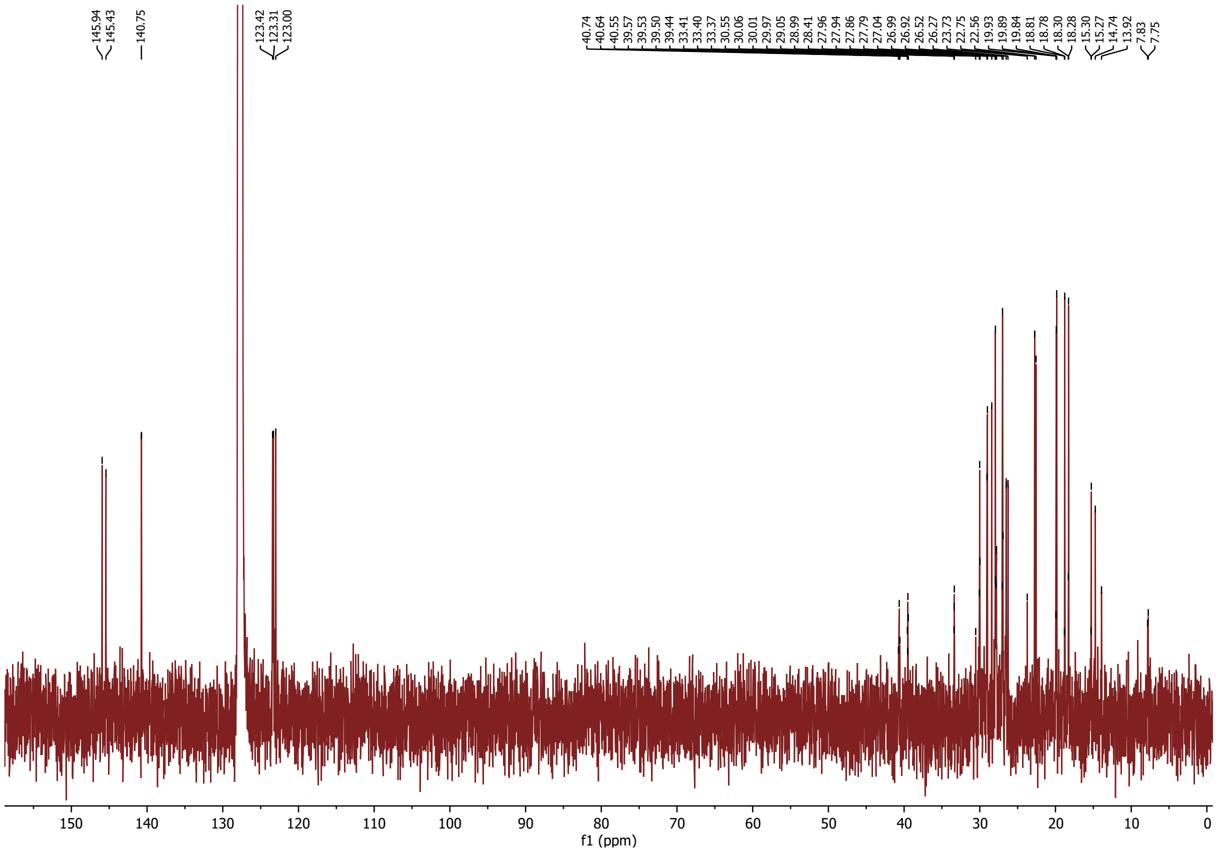

Figure S2. ^{13}C NMR spectrum (101 MHz, C_6D_6, 298 K) of **2**.

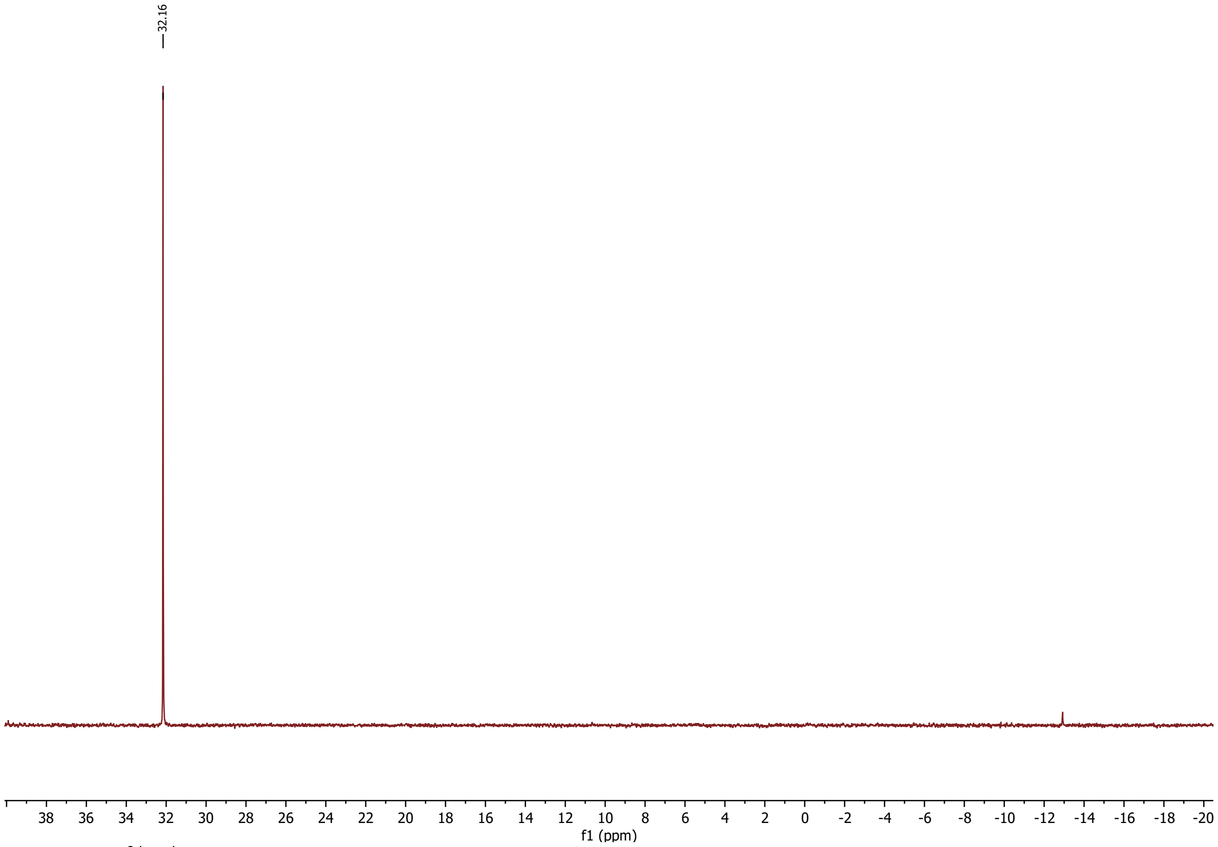

Figure S3. $^{31}P\{^{1}H\}$ NMR spectrum (162 MHz, C_6D_6, 298 K) of **2**.

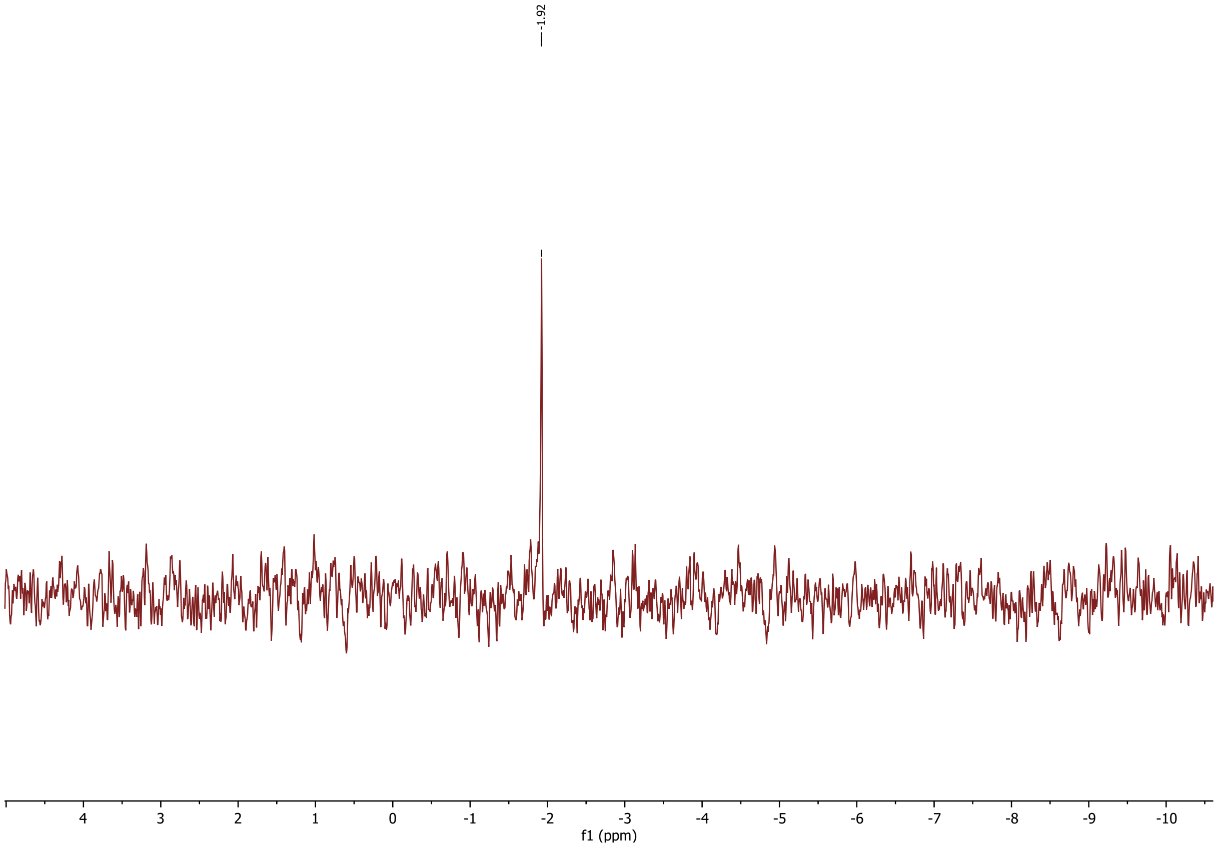

Figure S4. ^{29}Si NMR spectrum (99 MHz, C_6D_6, 298 K) of **2**.

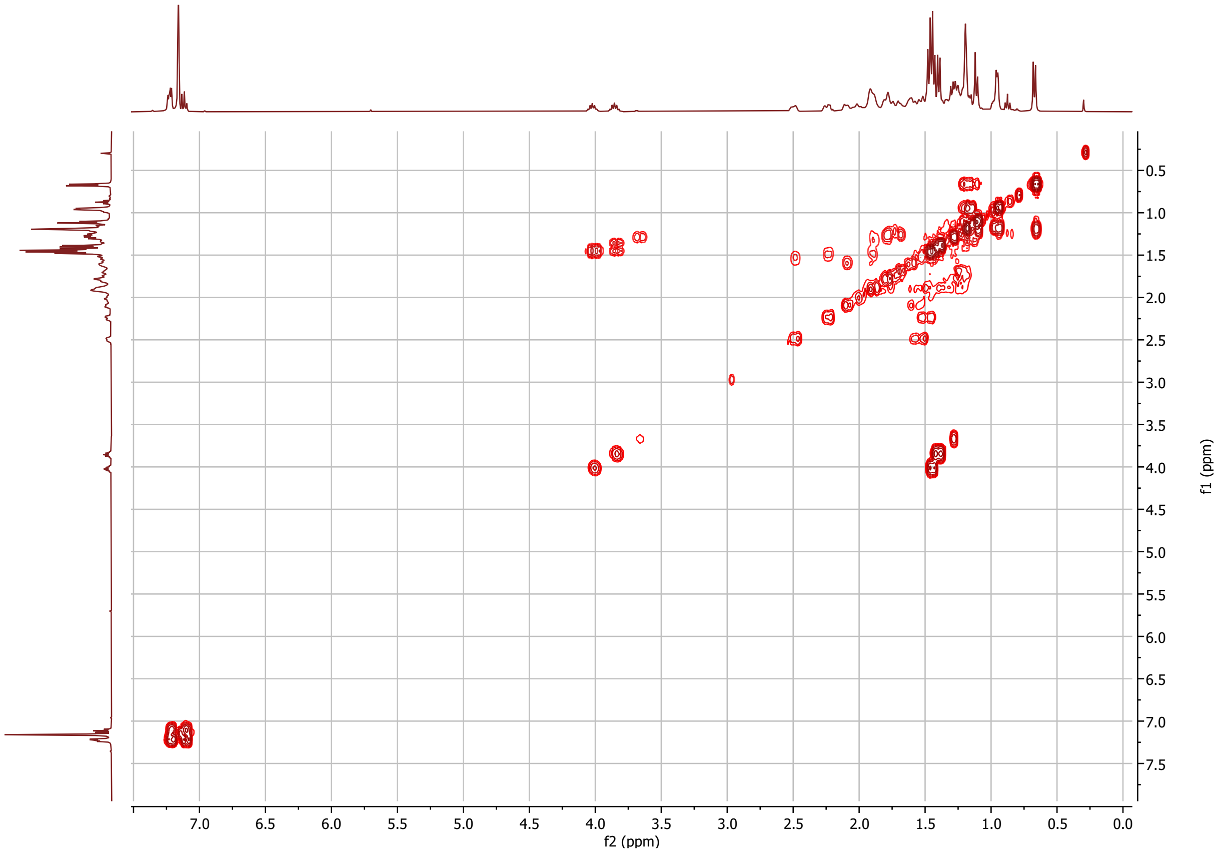

Figure S5. COSY NMR spectrum (400 MHz, C_6D_6, 298 K) of **2**.

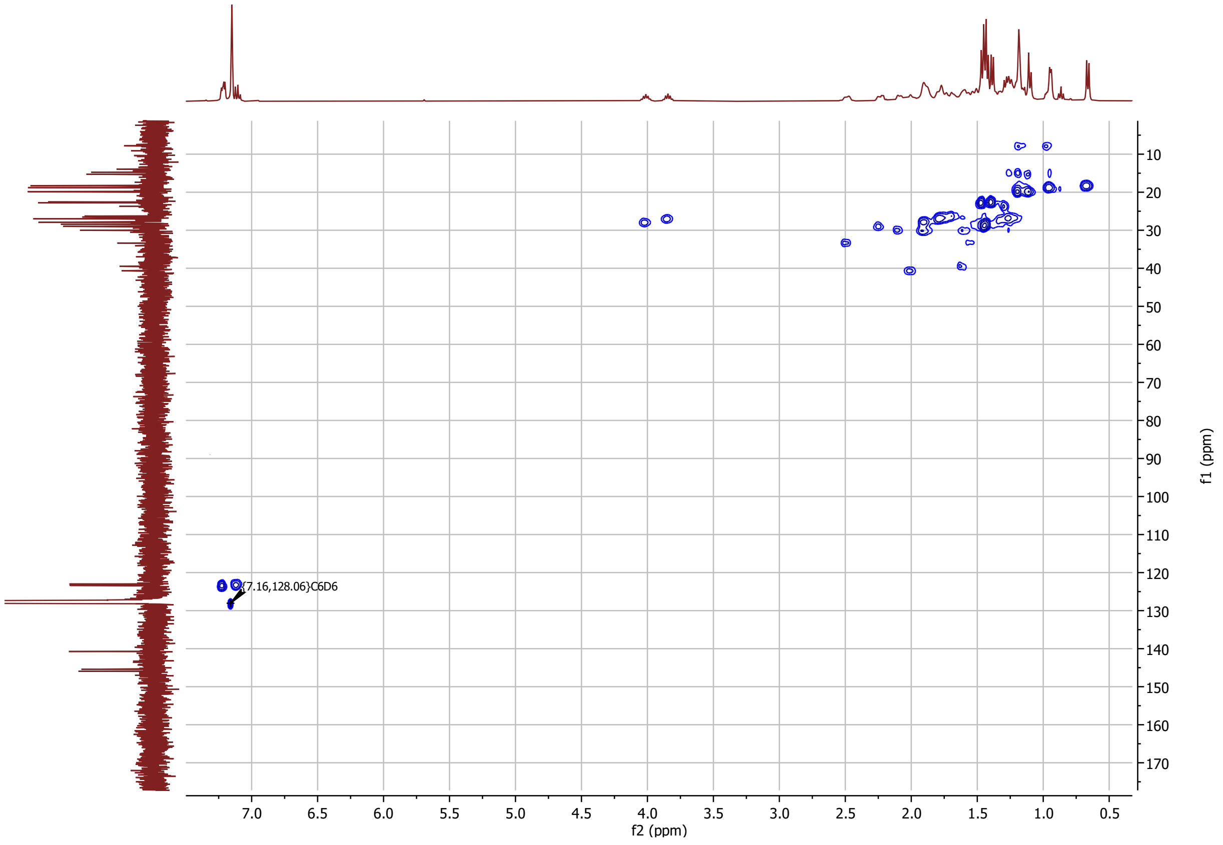

Figure S6. HSQC NMR spectrum (400/101 MHz, C_6D_6, 298 K) of **2**.

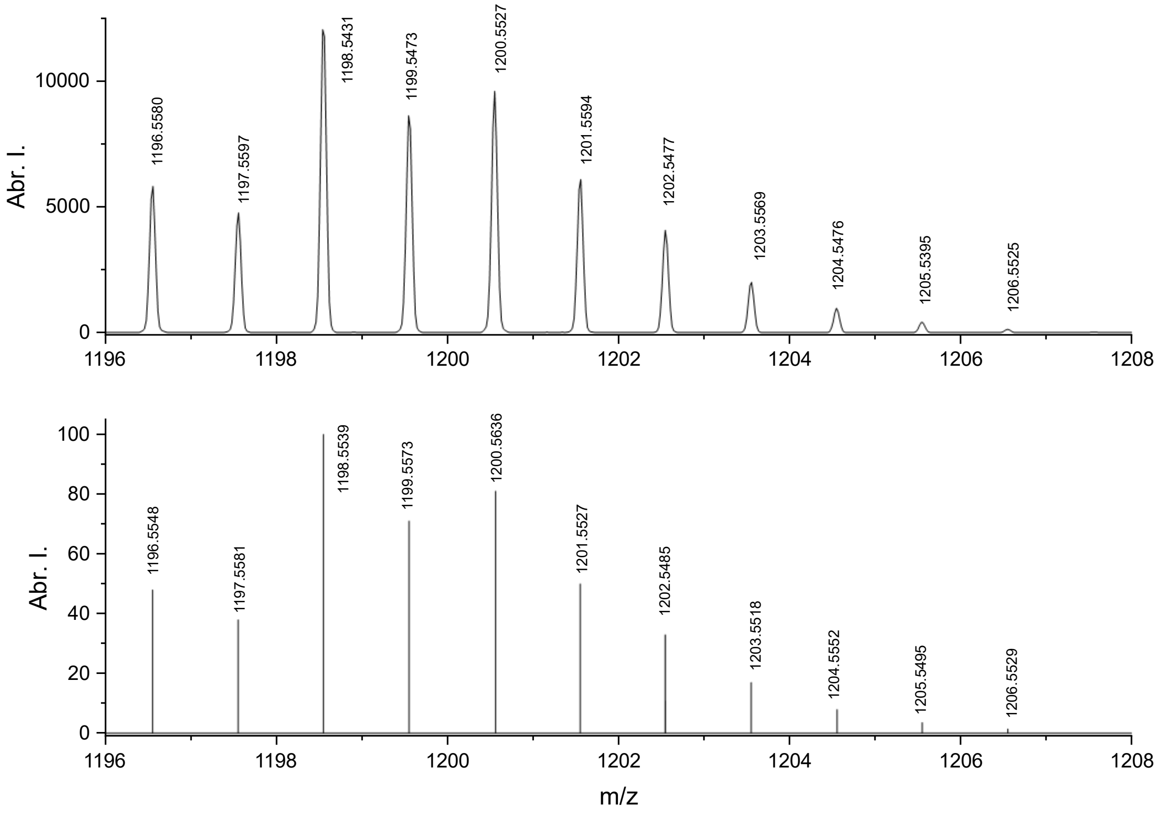

Figure S7. LIFDI/MS of **2**, Top. Found MS for $[C_{62}H_{110}N_2P_2Si_2Ga_2Ni]^+$ Bottom. Calculated MS spectrum of $[C_{62}H_{110}N_2P_2Si_2Ga_2Ni]^+$.

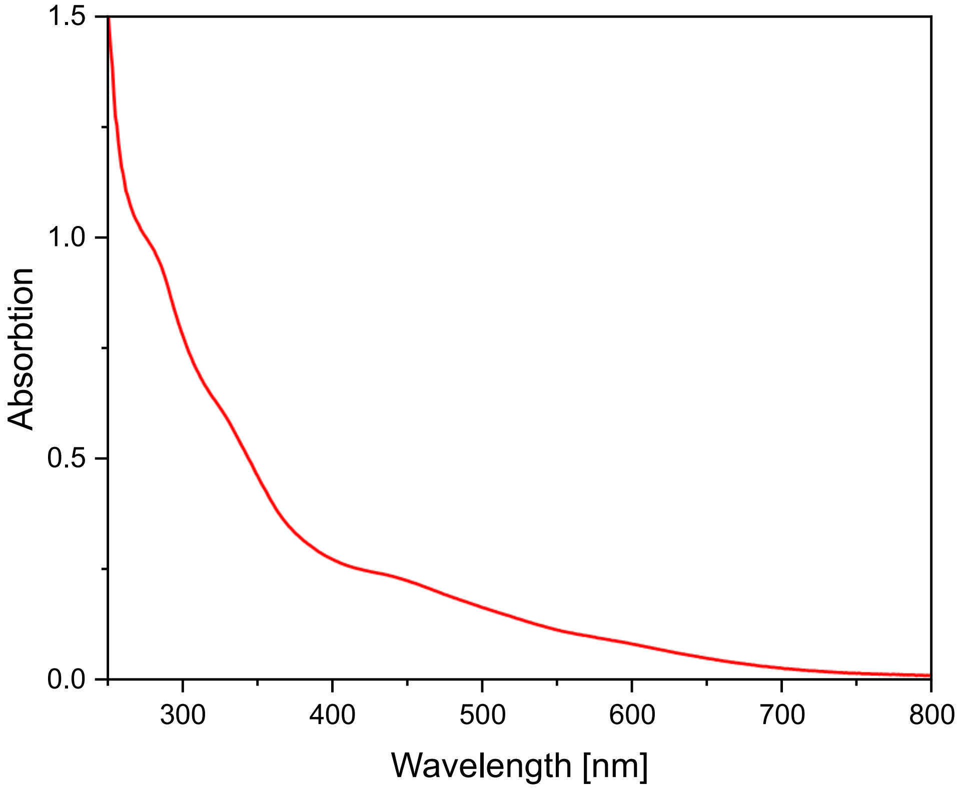

Figure S8. UV/Vis spectrum of a 0.625×10^{-4} M solution of **2** in Toluene at ambient temperature.

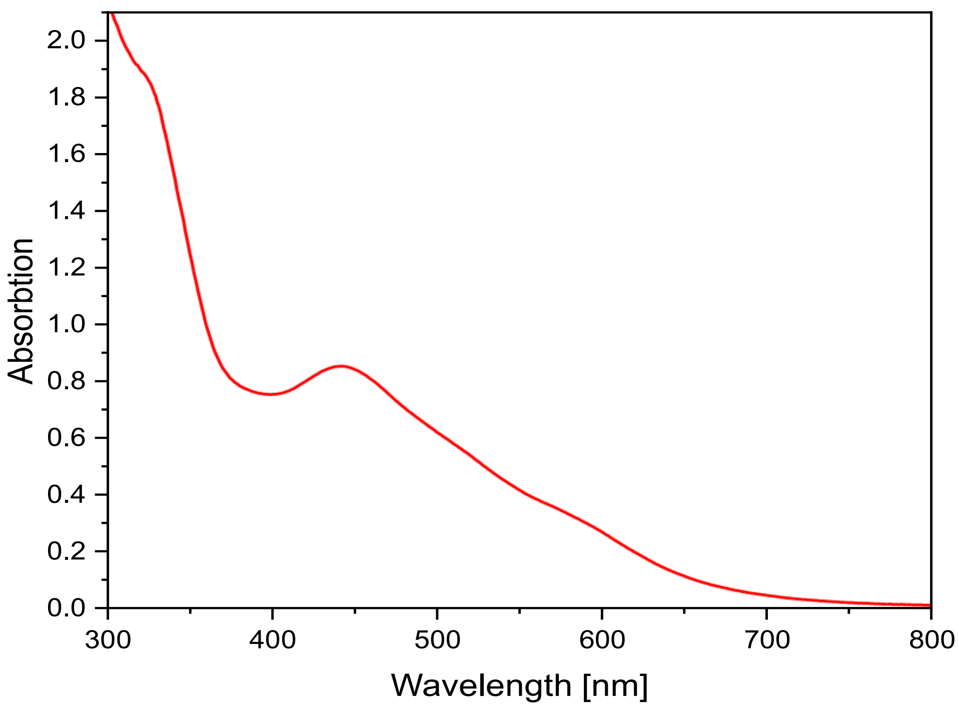

Figure S9. UV/Vis spectrum of a 1.25x10^{-4} M solution of **2** in Toluene at ambient temperature.

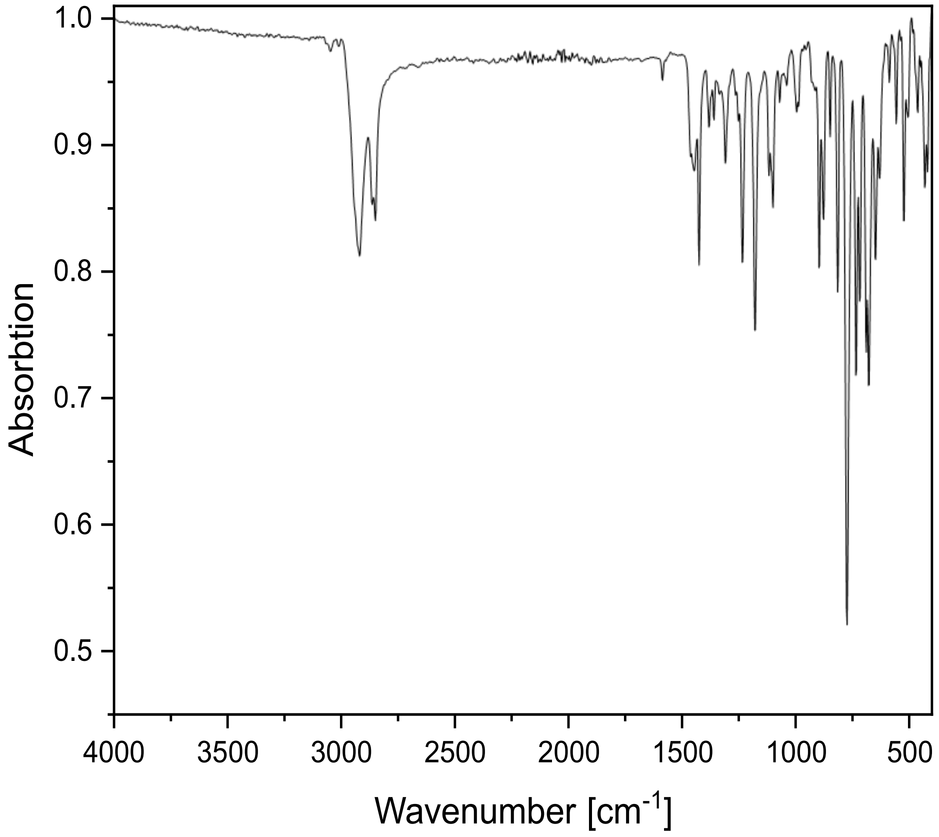

Figure S10. ATR-IR spectrum of **2** at ambient temperature.

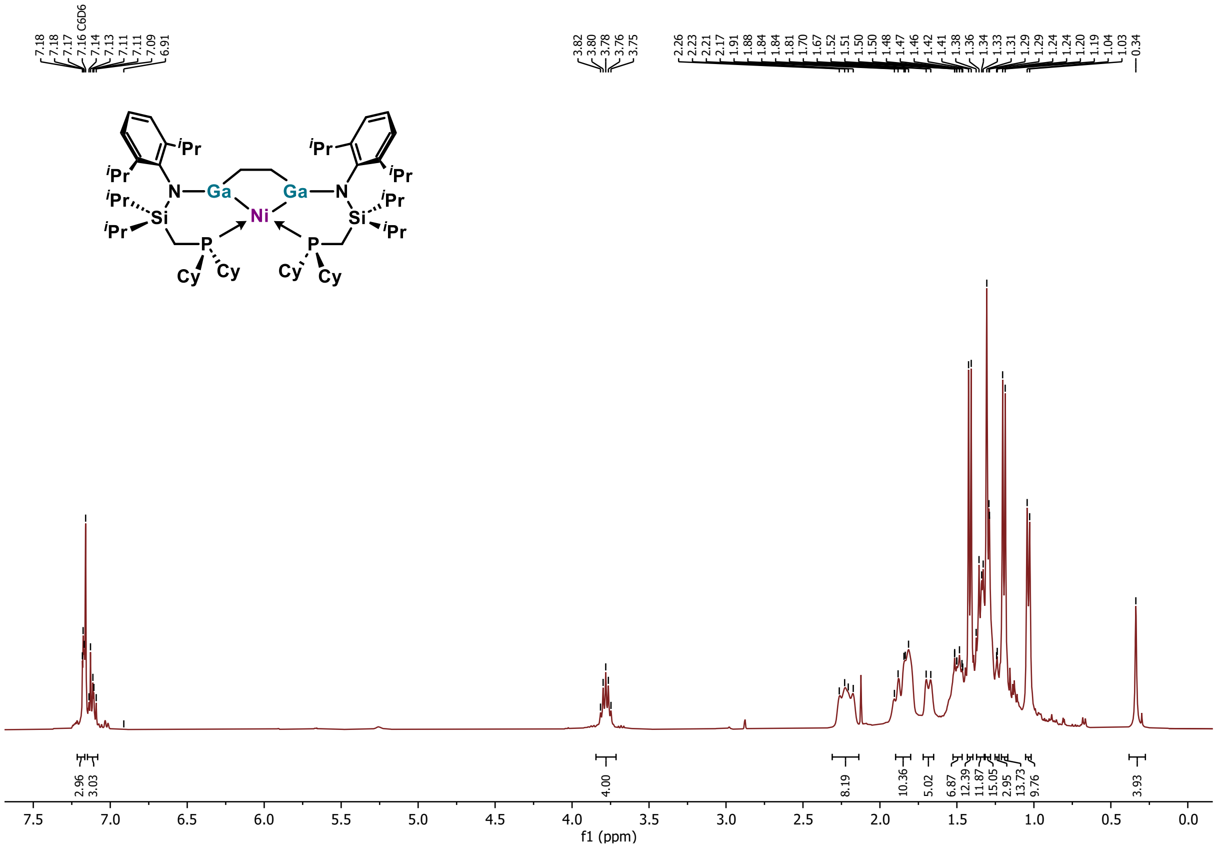

Figure S11. ^{1}H NMR spectrum (400 MHz, C_6D_6, 298 K) of **3**.

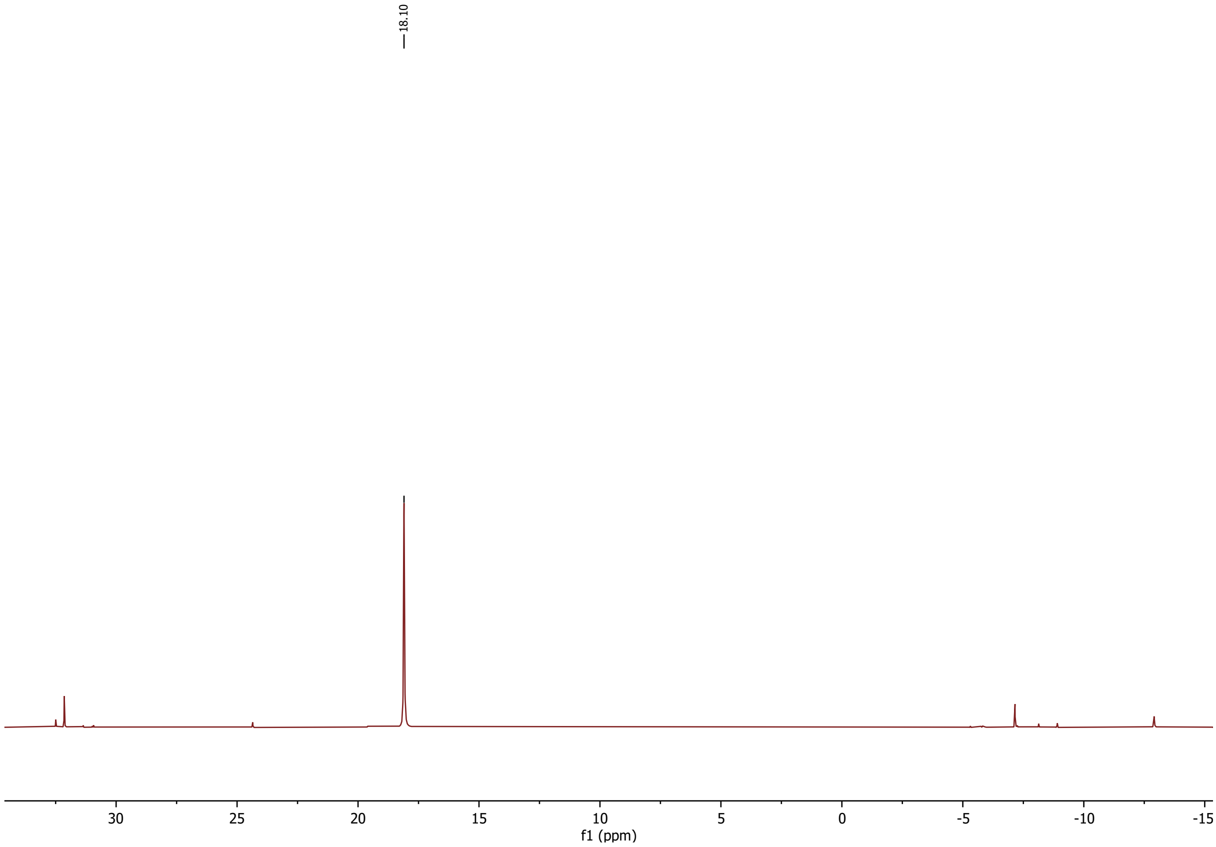

Figure S12. ^{31}P{^{1}H} NMR spectrum (162 MHz, C_6D_6, 298 K) of **3**.

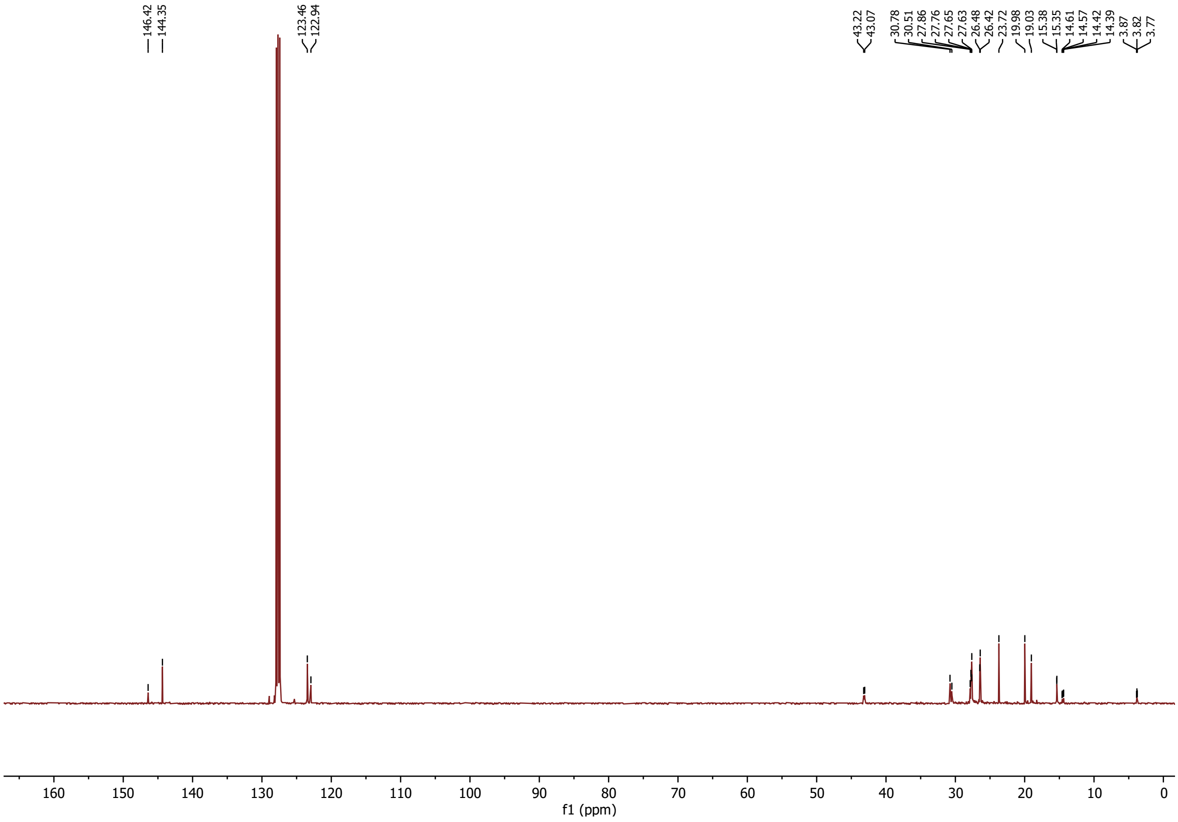

Figure S13. ^{13}C NMR spectrum (101 MHz, C_6D_6, 298 K) of **3**.

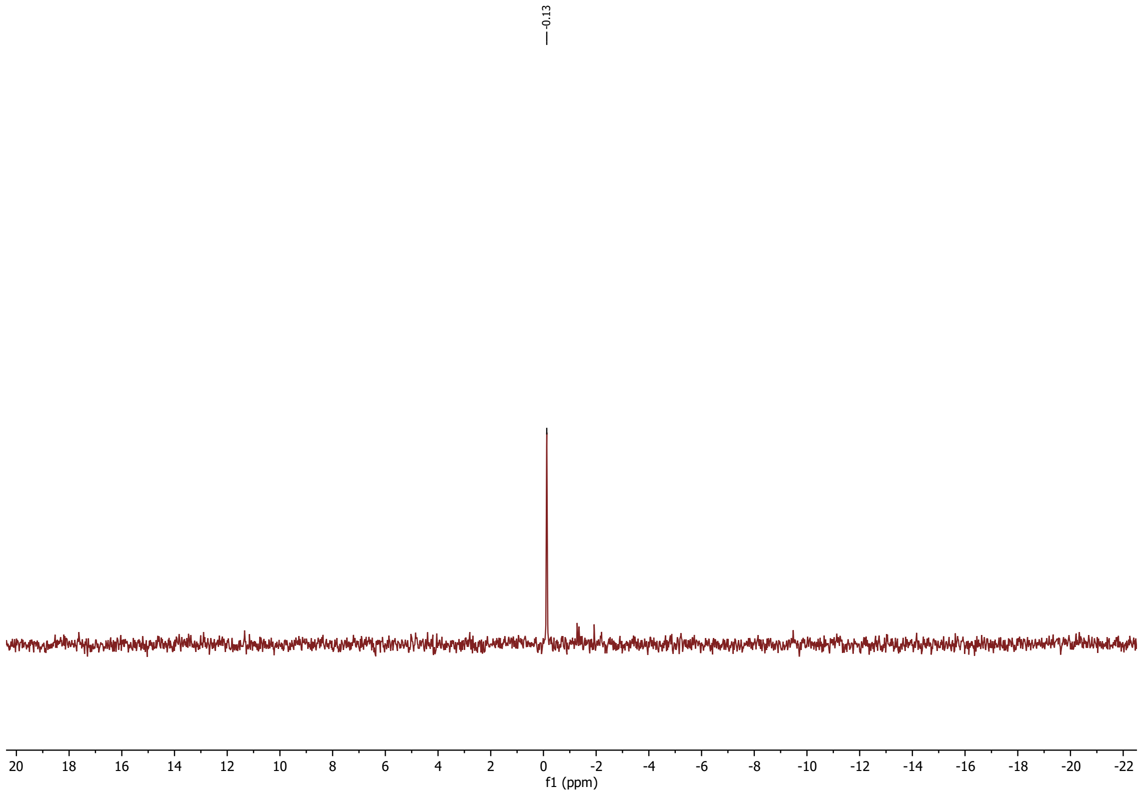

Figure S14. ^{29}Si NMR spectrum (99 MHz, C_6D_6, 298 K) of **3**.

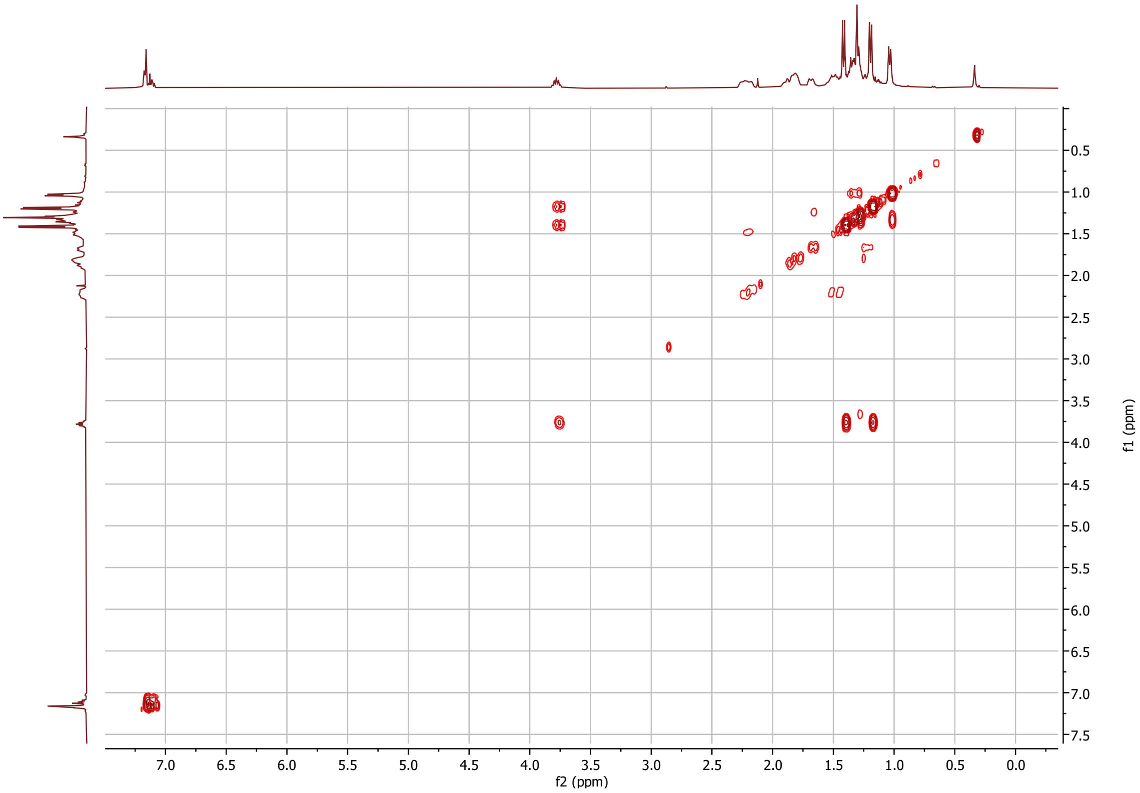

Figure S15. COSY NMR spectrum (400 MHz, C_6D_6, 298 K) of **3**.

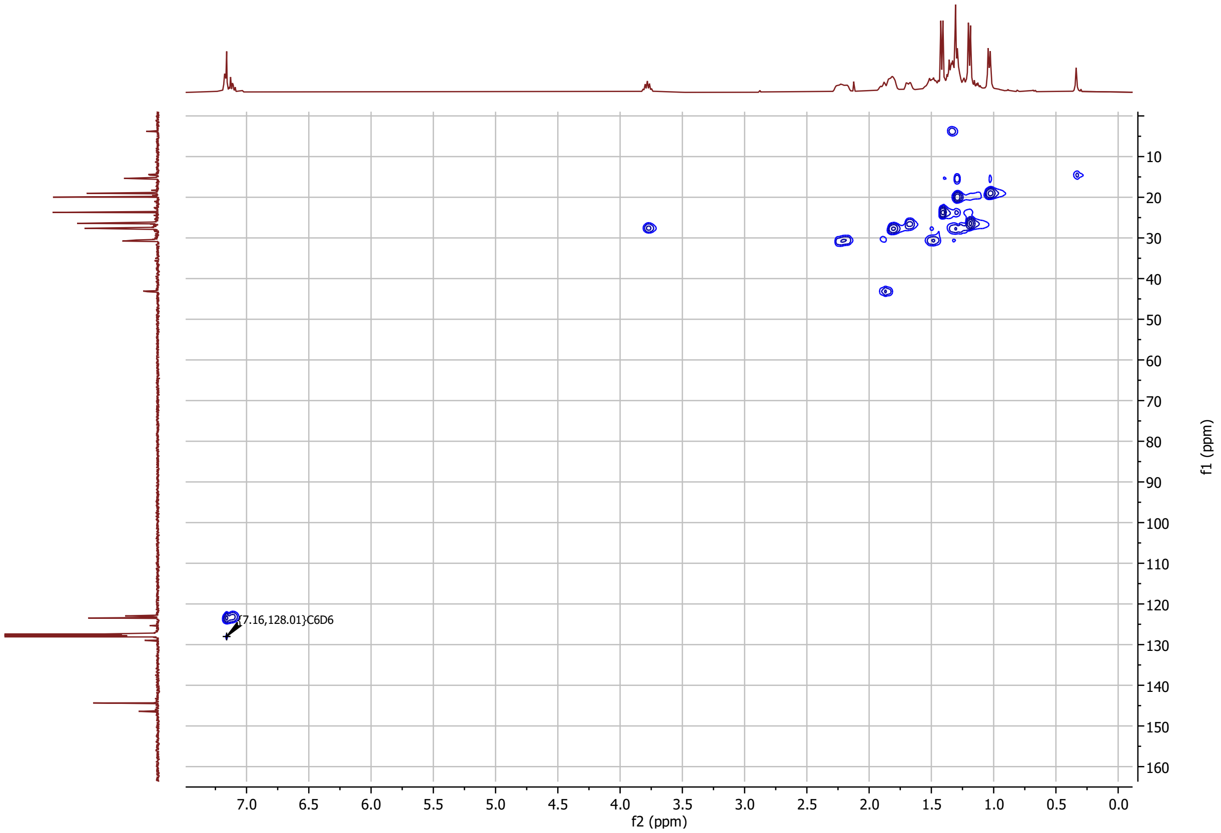

Figure S16. HSQC NMR spectrum (400/101 MHz, C_6D_6, 298 K) of **3**.

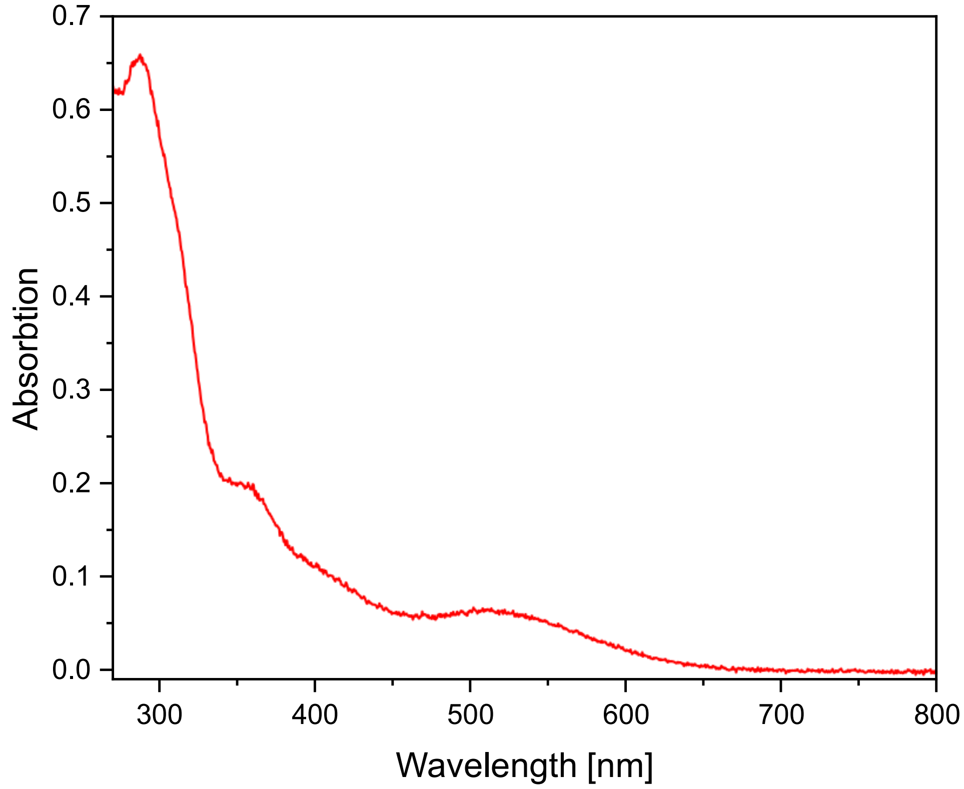

Figure S17. UV/Vis spectrum of a 0.625x10^{-4} M solution of **3** in THF at ambient temperature.

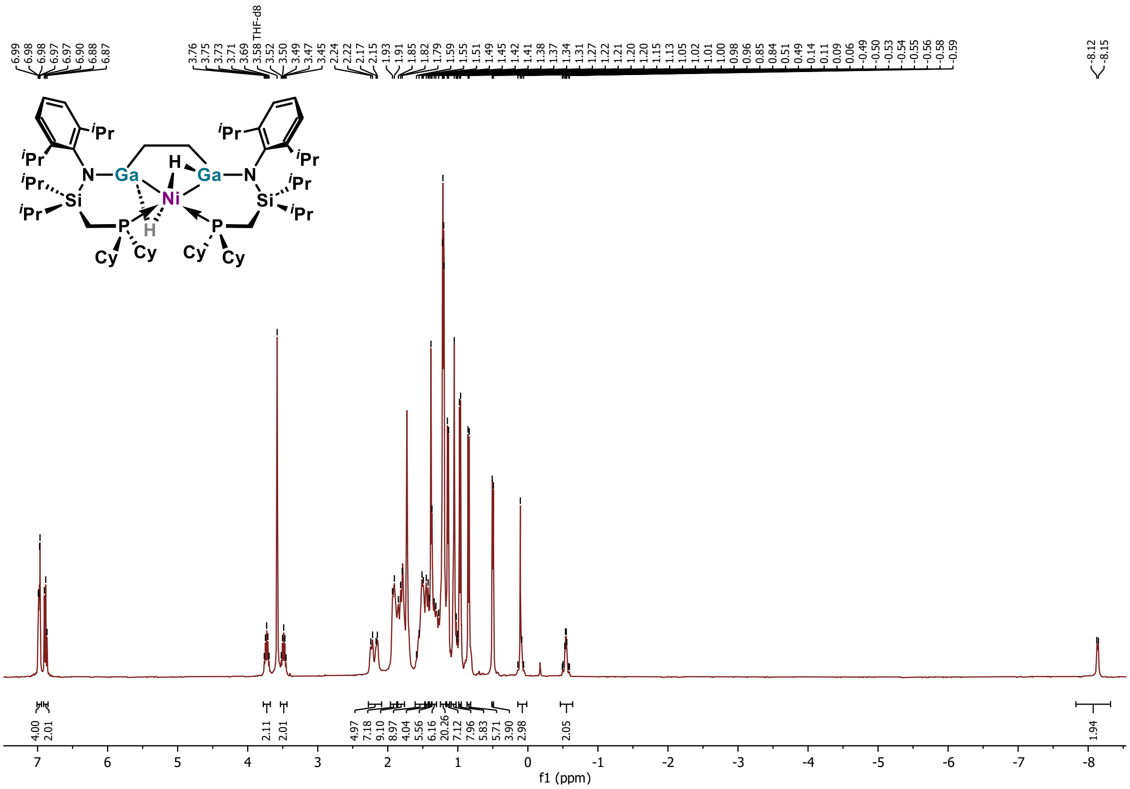

Figure S18. ^{1}H NMR spectrum (400 MHz, THF-d_8, 298 K) of **4**.

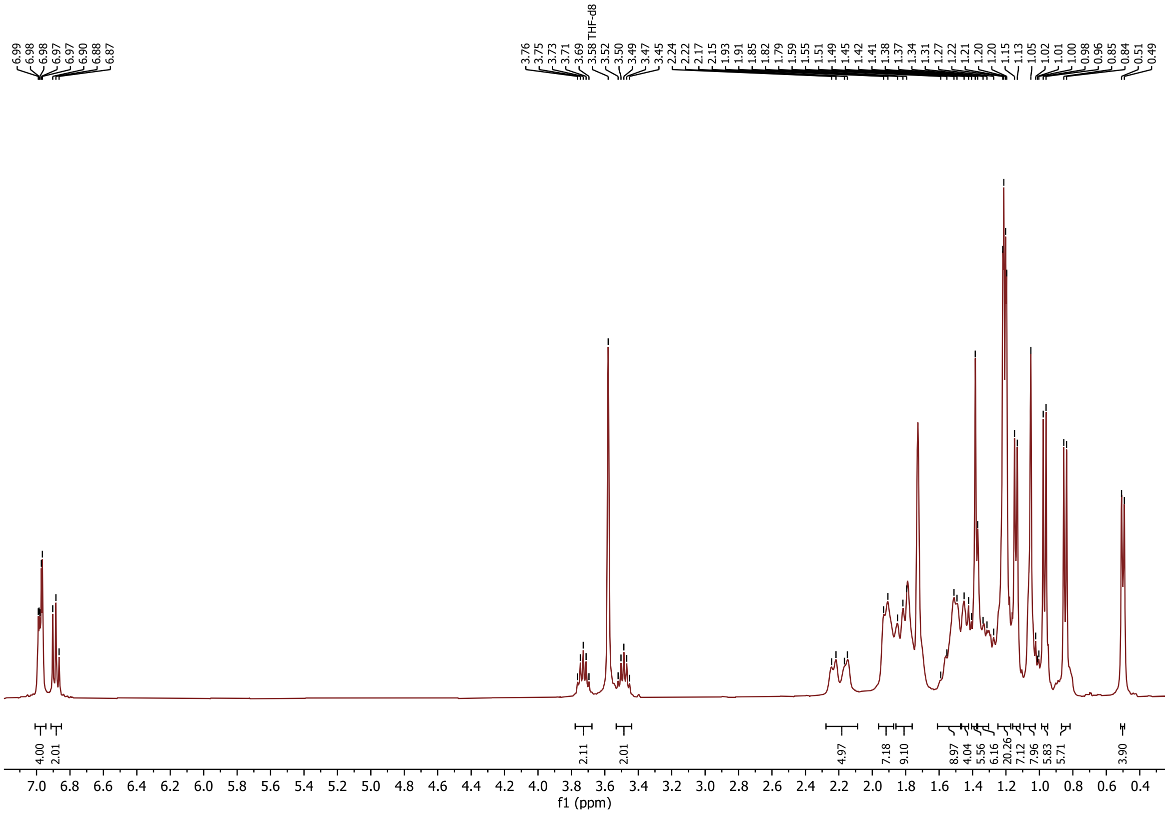

Figure S19. ^{1}H NMR spectrum (400 MHz, THF-d_8, 298 K) of **4,** from 7.2 - 0.4 ppm.

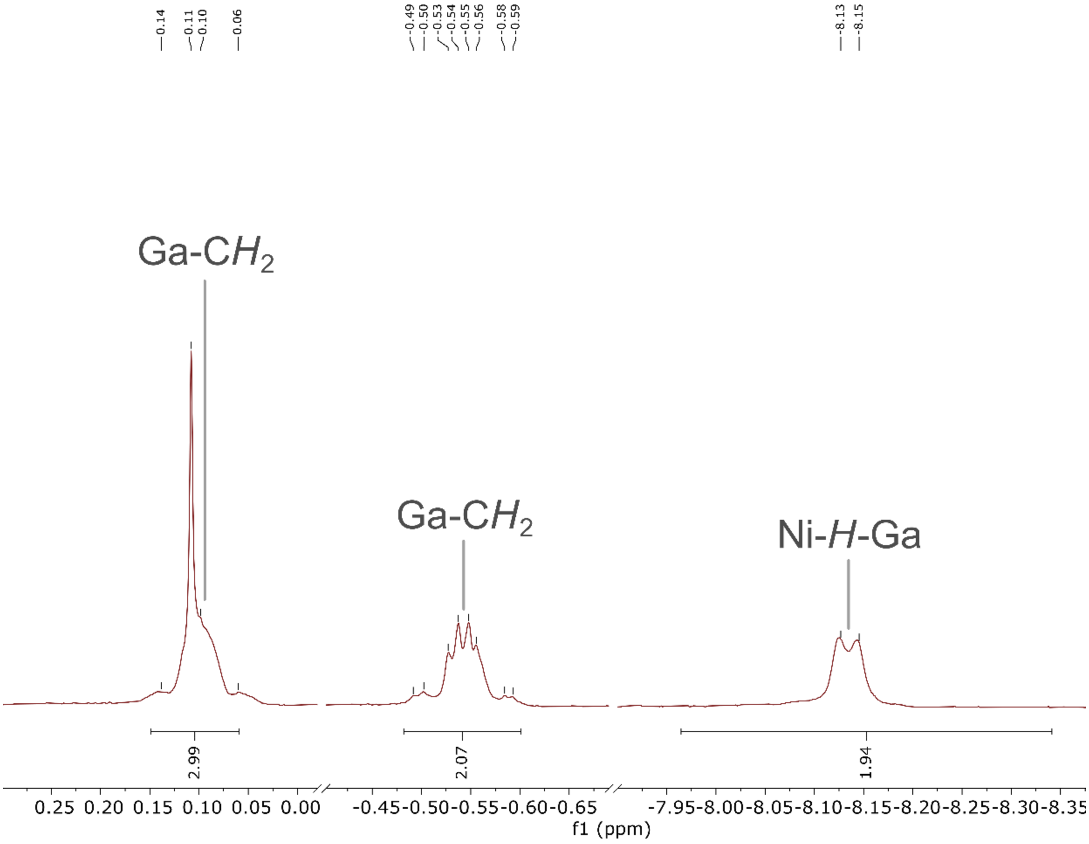

Figure S20. ^{1}H NMR spectrum (400 MHz, THF-d_8, 298 K) of **4** zoomed into into the shift area of the ethyl bridge and the hydride signals.

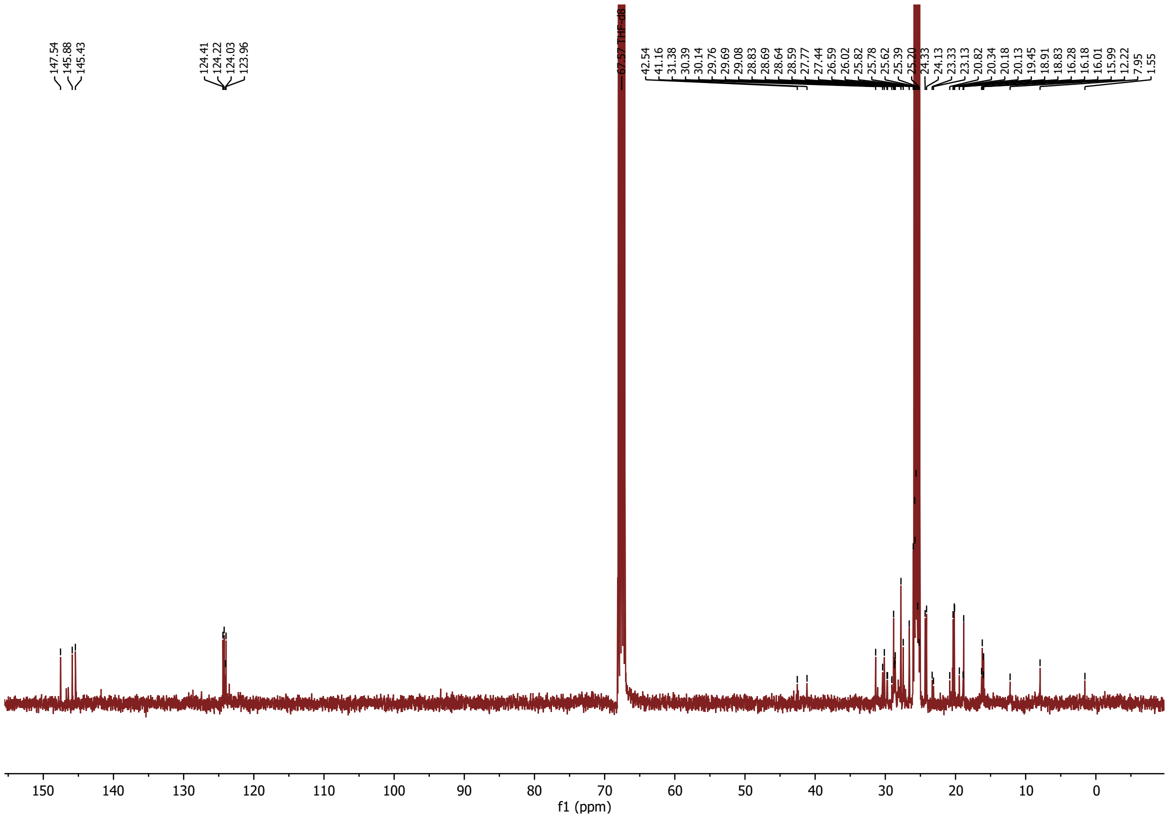

Figure S21. ^{13}C NMR spectrum (101 MHz, THF-d_8, 298 K) of **4**.

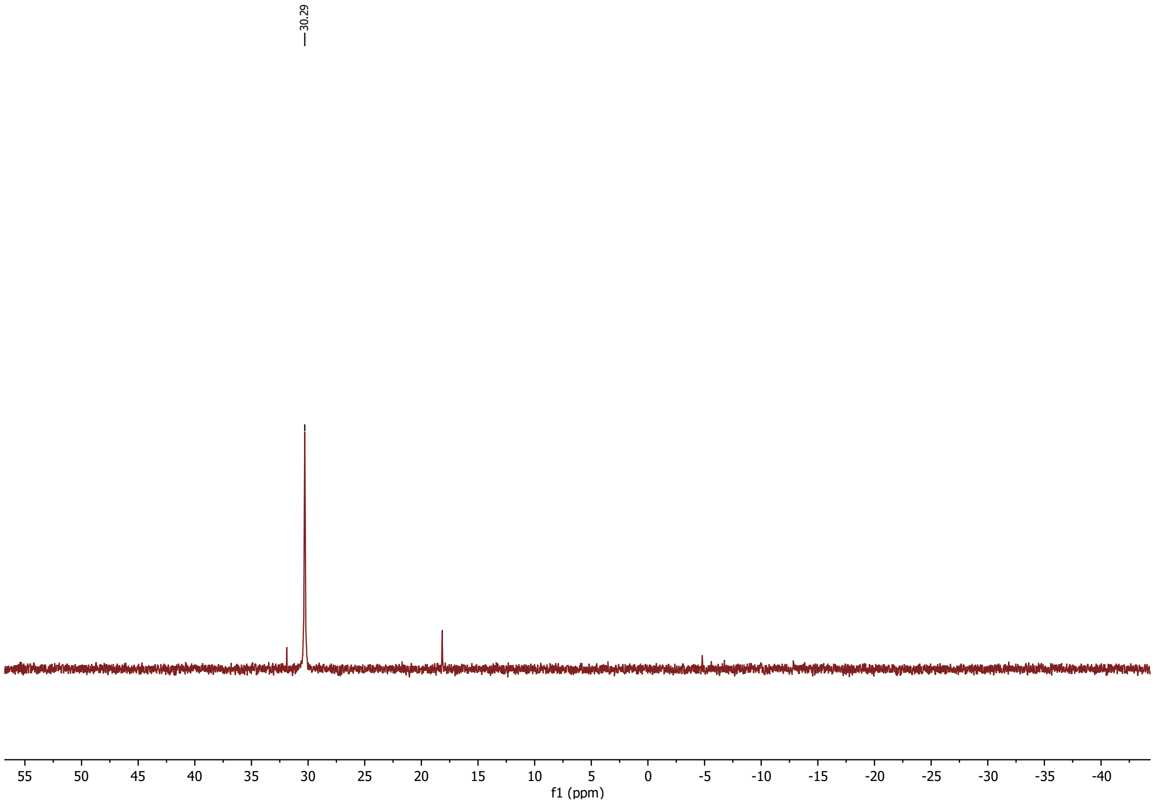

Figure S22. $^{31}P\{^1H\}$ NMR spectrum (162 MHz, THF-d_8, 298 K) of **4**.

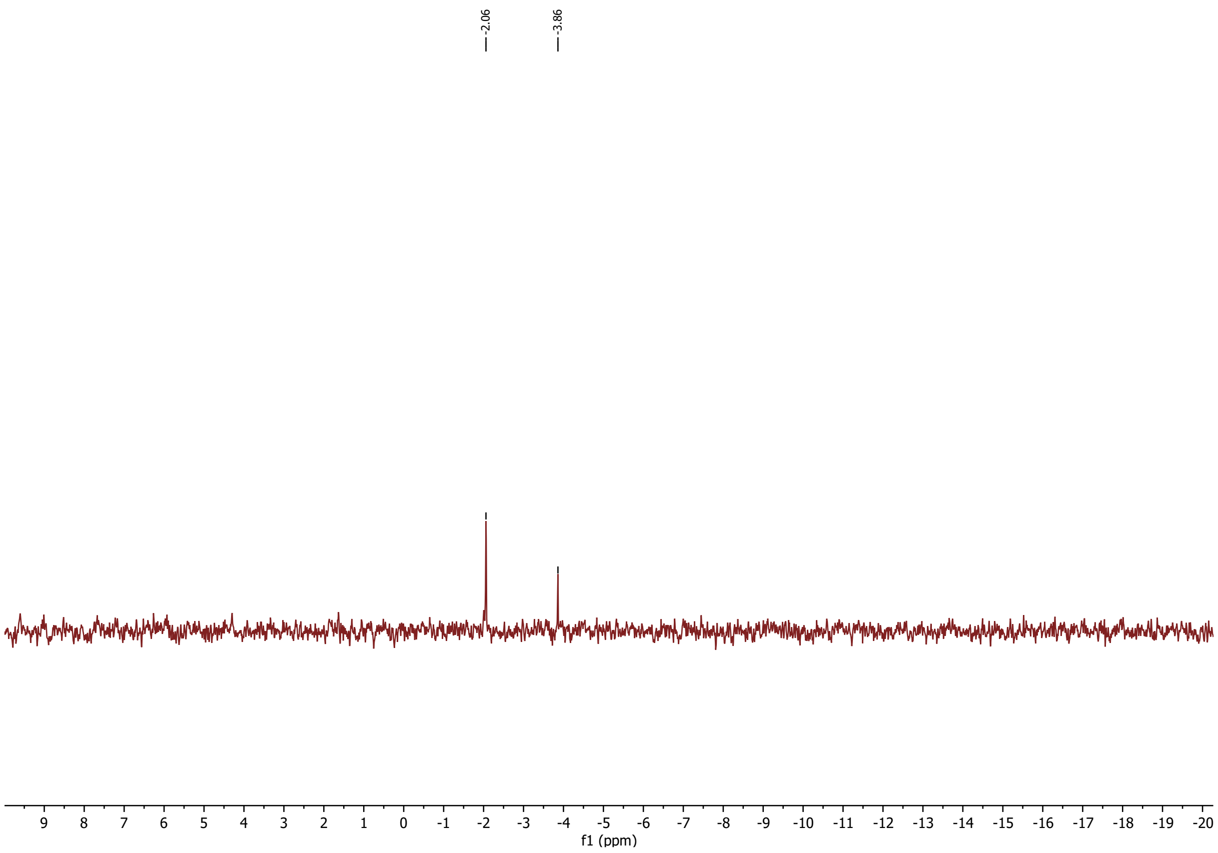

Figure S23. ^{29}Si NMR spectrum (99 MHz, THF-d_8, 298 K) of **4**.

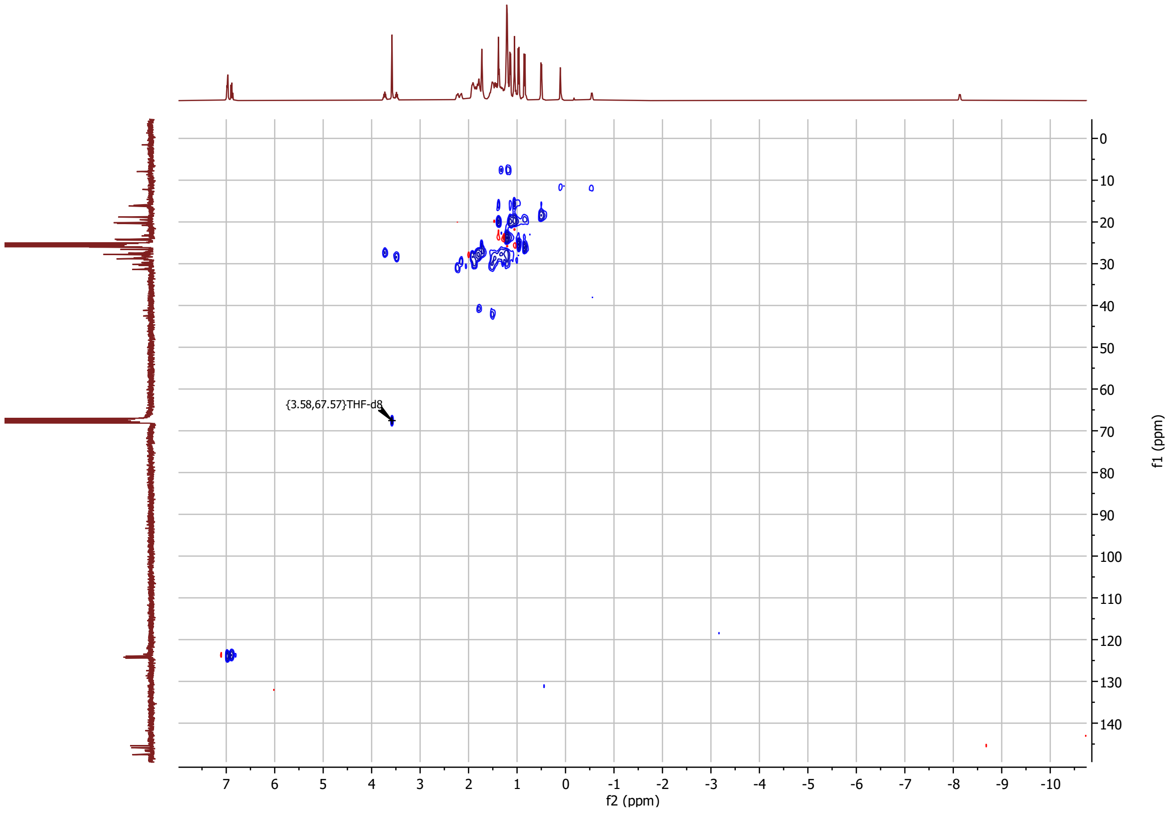

Figure S24. HSQC NMR spectrum (400/101 MHz, THF-d_8, 298 K) of **4**.

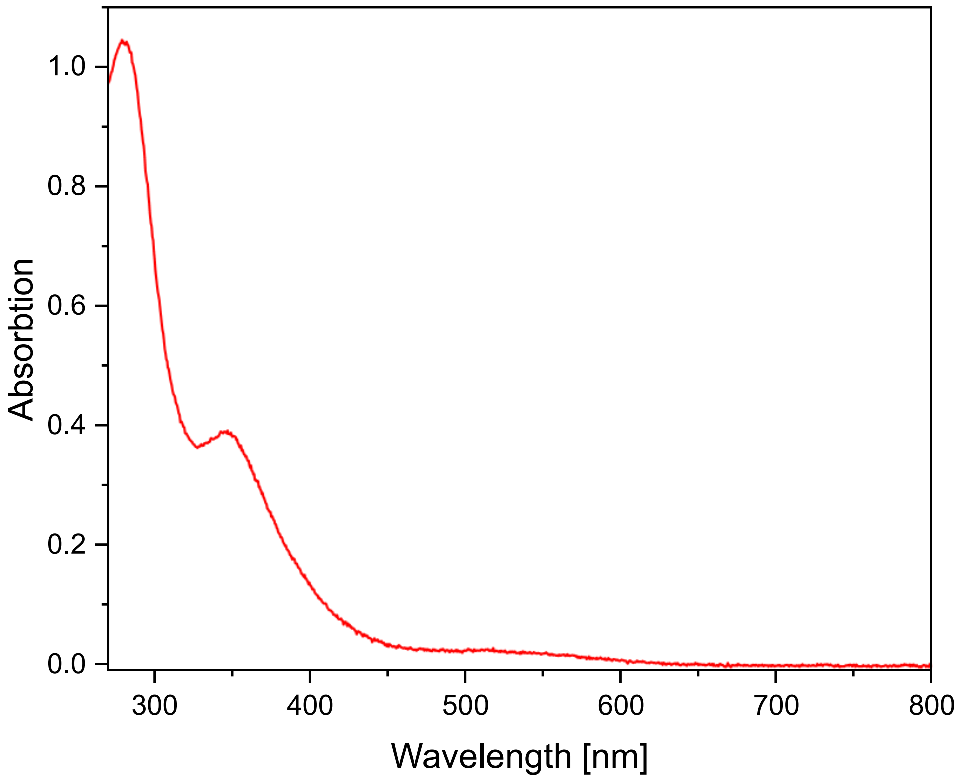

Figure S25. UV/Vis spectrum of a 0.625x10^{-4} M solution of **4** in THF at ambient temperature.

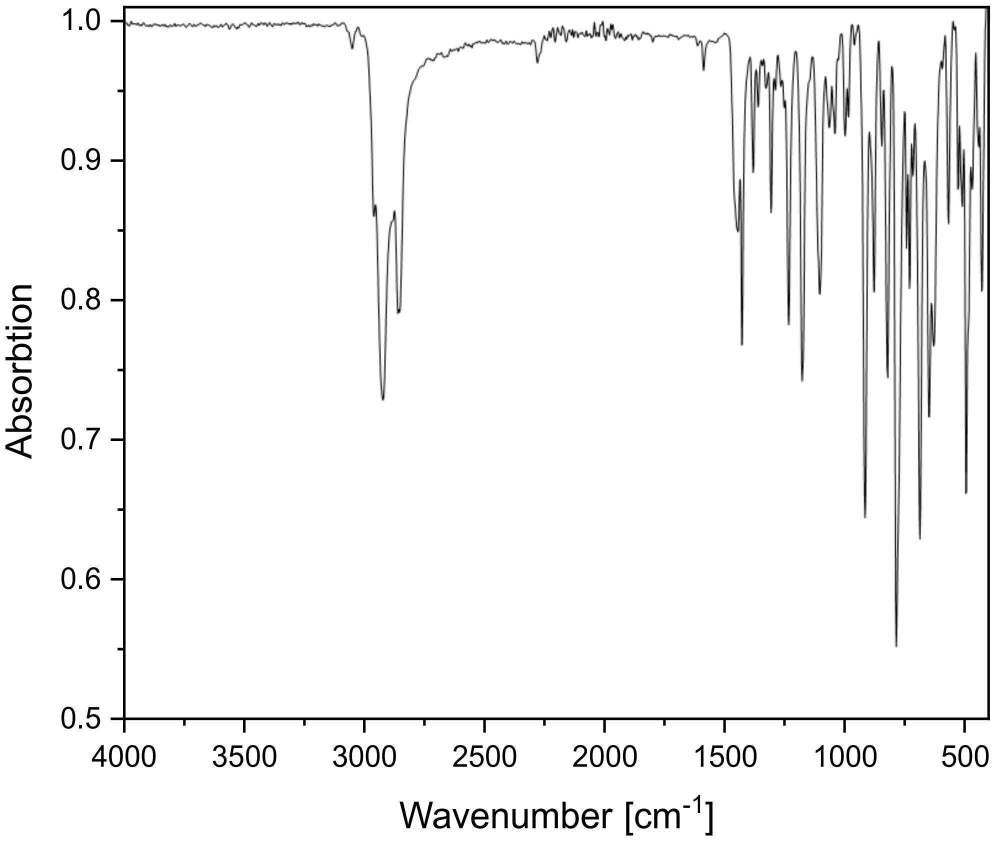

Figure S26. ATR-IR spectrum of **4** at ambient temperature.

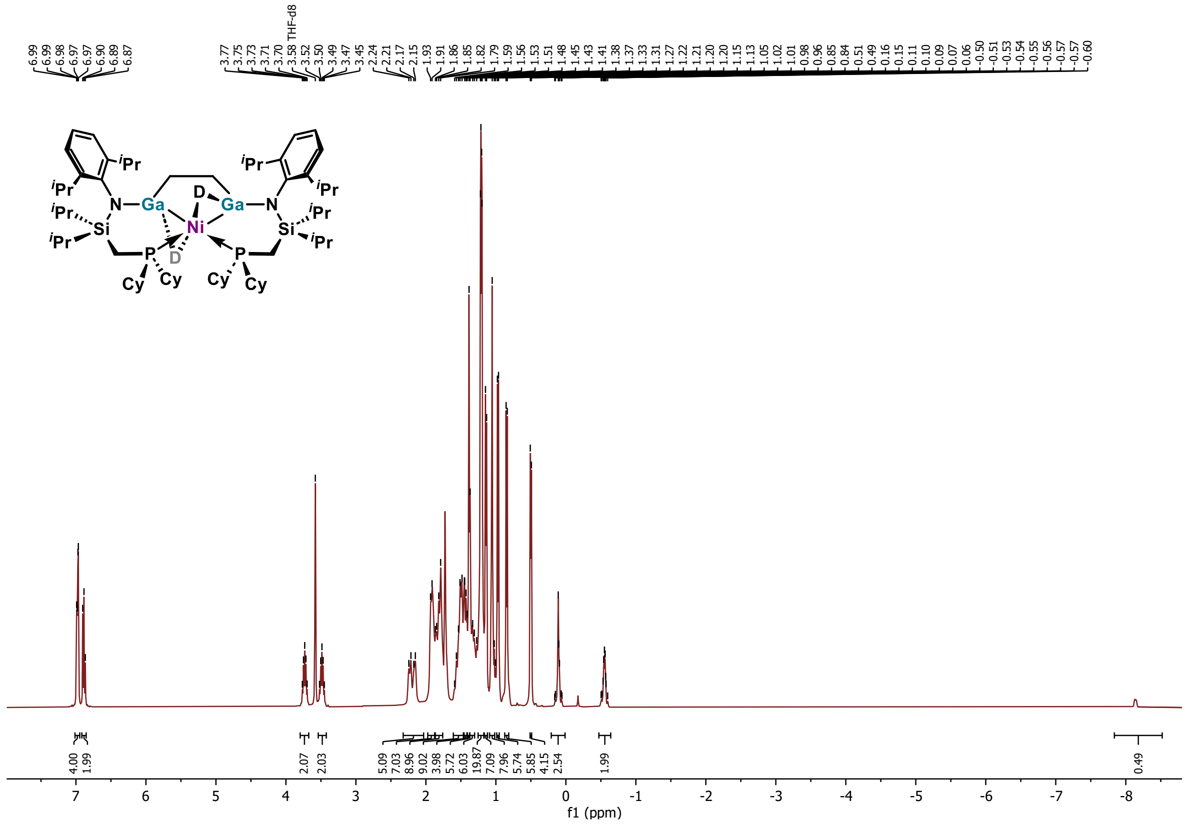

Figure S27. ^{1}H NMR spectrum (400 MHz, THF-d_8, 298 K) of **4-D**.

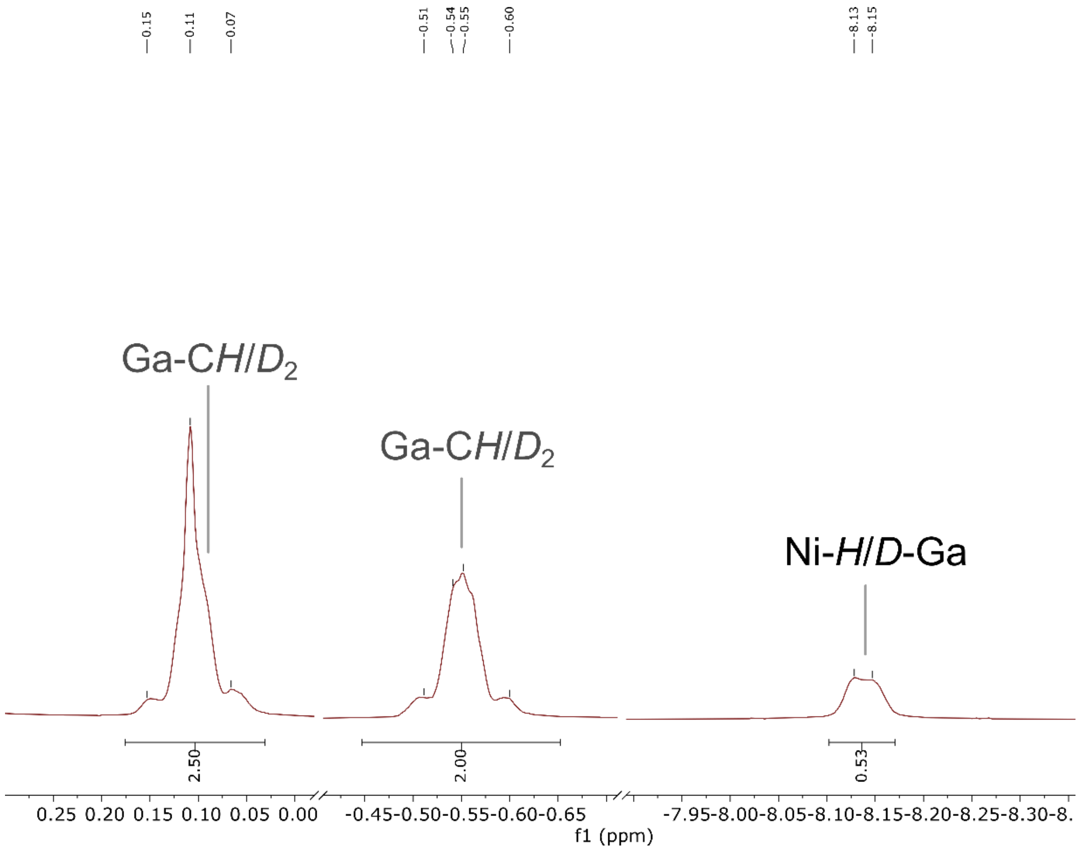

Figure S28. ^{1}H NMR spectrum (400 MHz, THF-d_8, 298 K) of **4-D** zoomed into into the shift area of the ethyl bridge and the hydride signals.

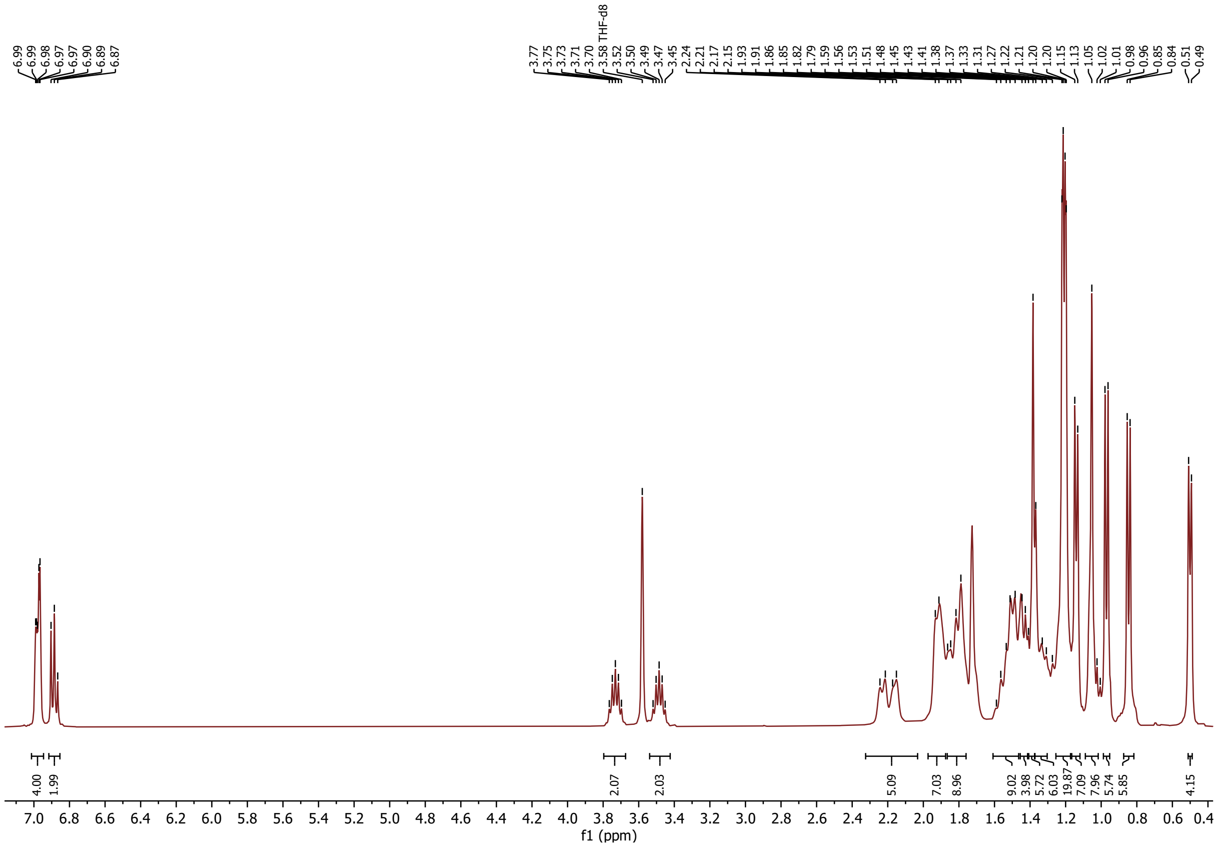

Figure S29. ^{1}H NMR spectrum (400 MHz, THF-d_8, 298 K) of **4-D,** from 7.2 - 0.4 ppm.

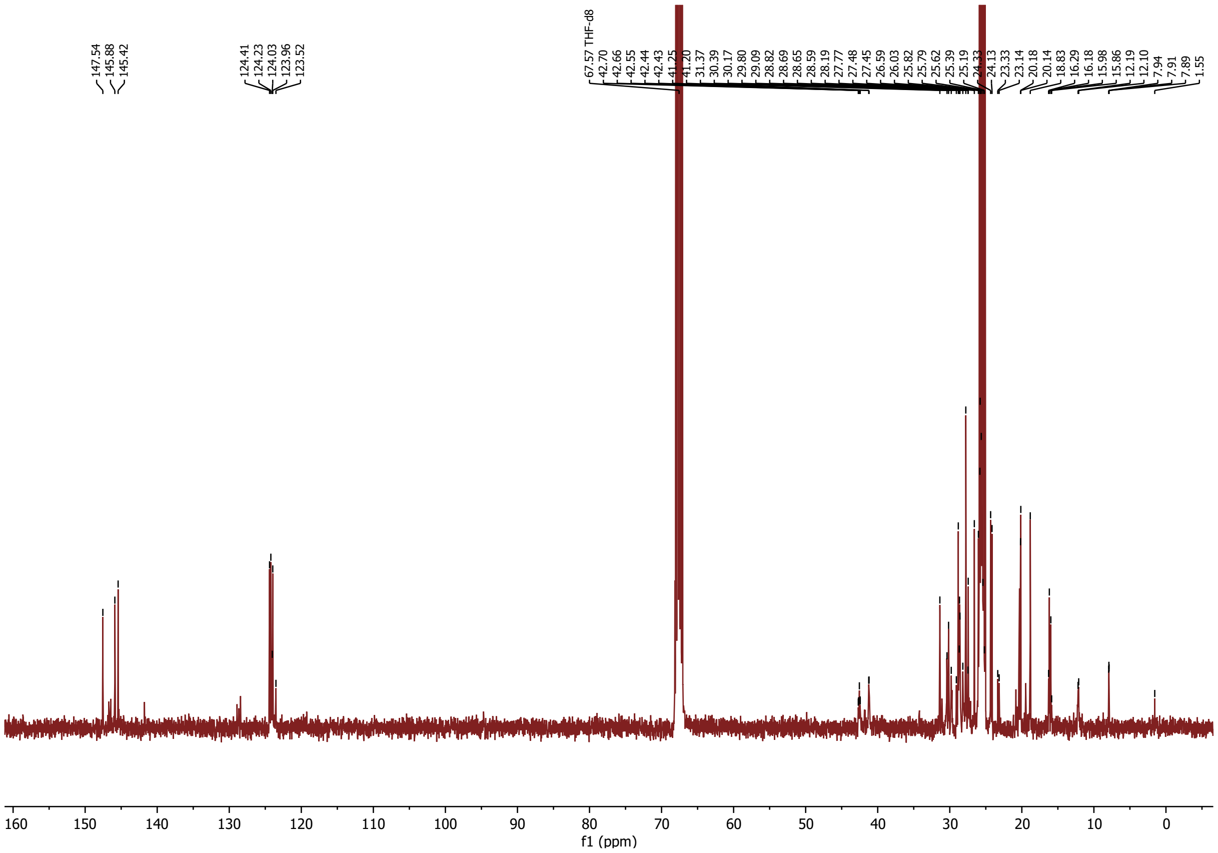

Figure S30. ^{13}C NMR spectrum (101 MHz, THF-d_8, 298 K) of **4-D**.

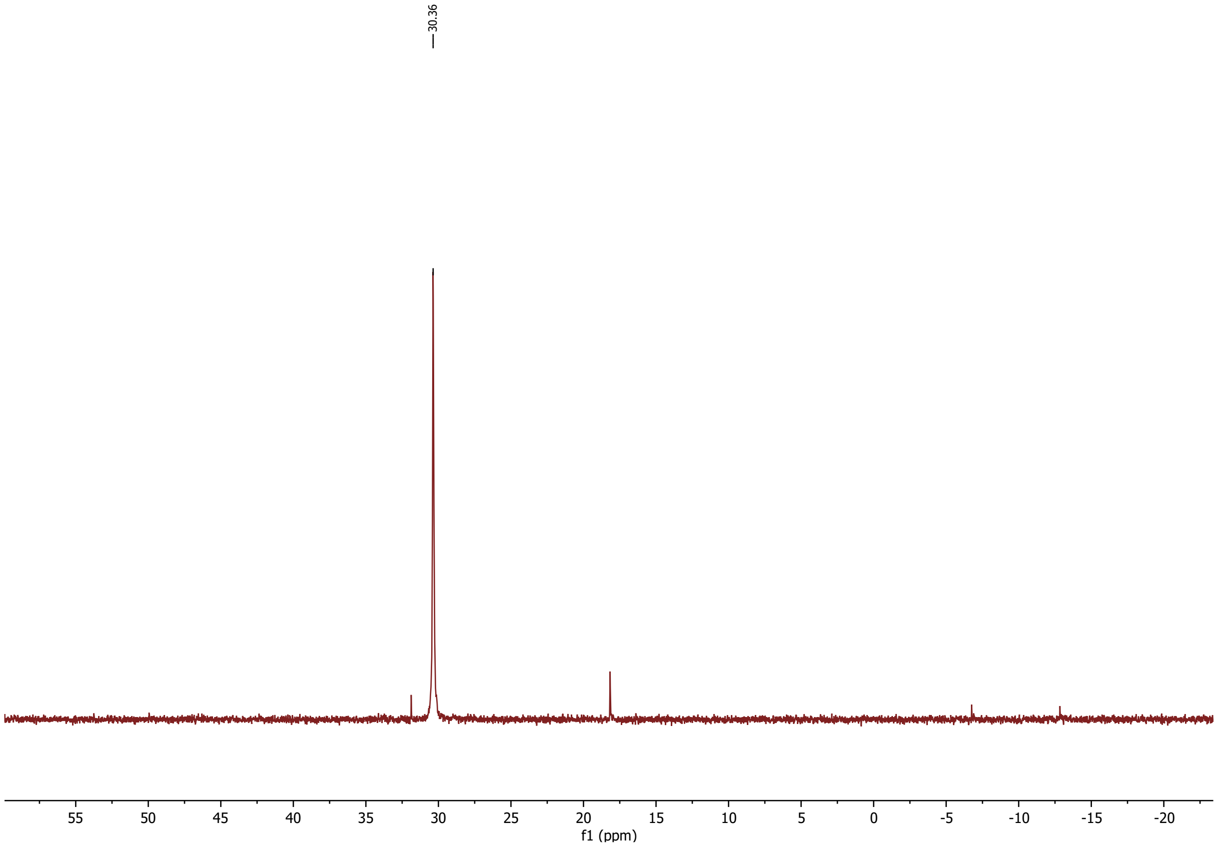

Figure S31. $^{31}P\{^{1}H\}$ NMR spectrum (162 MHz, THF-d_8, 298 K) of **4-D**.

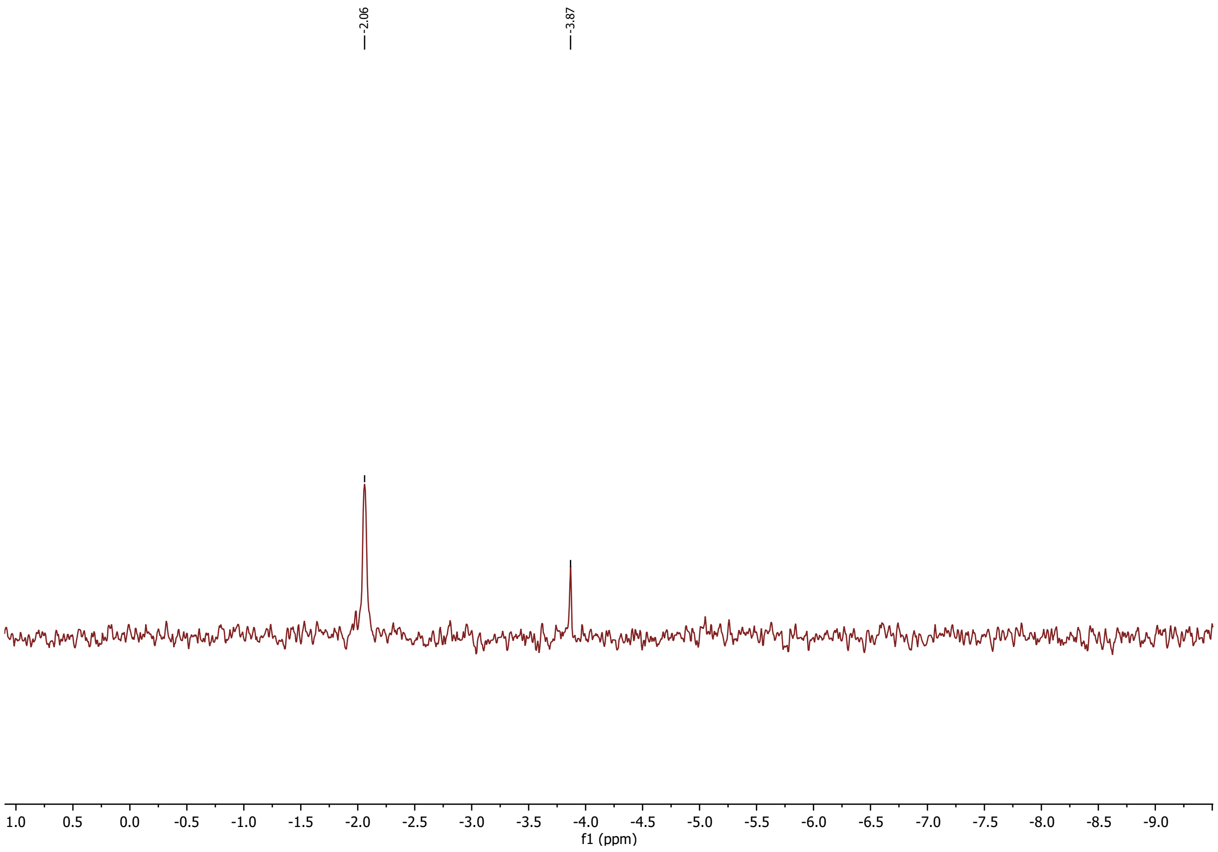

Figure S32. ^{29}Si NMR spectrum (99 MHz, THF-d_8, 298 K) of **4-D**.

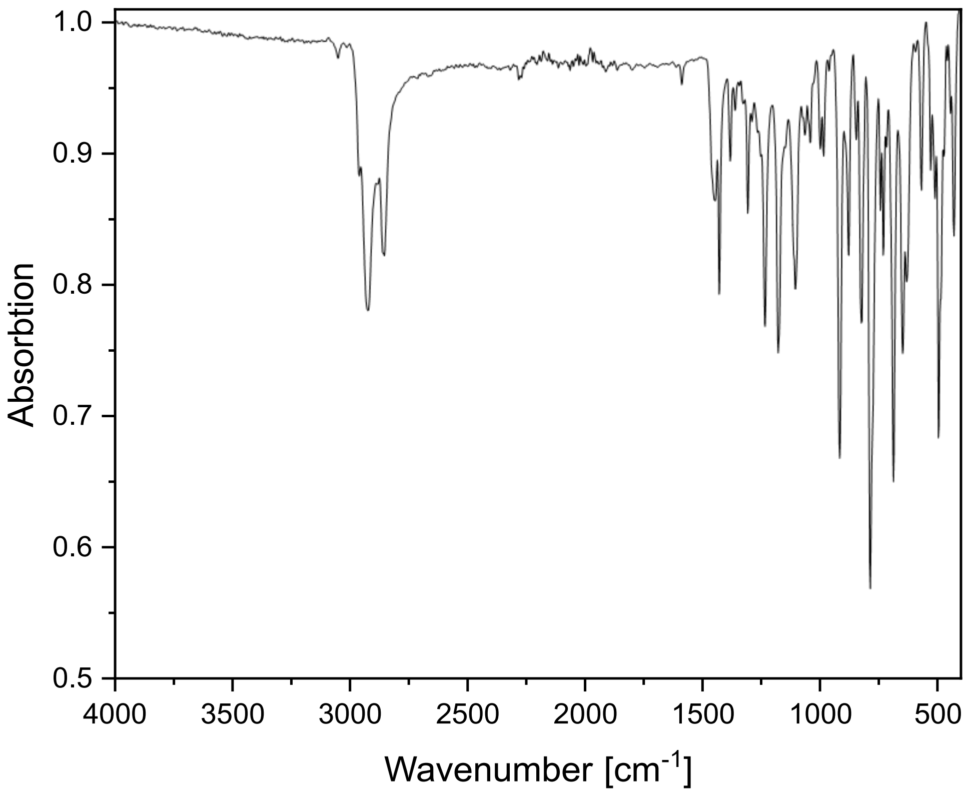

Figure S33. ATR-IR spectrum of **4-D** at ambient temperature.

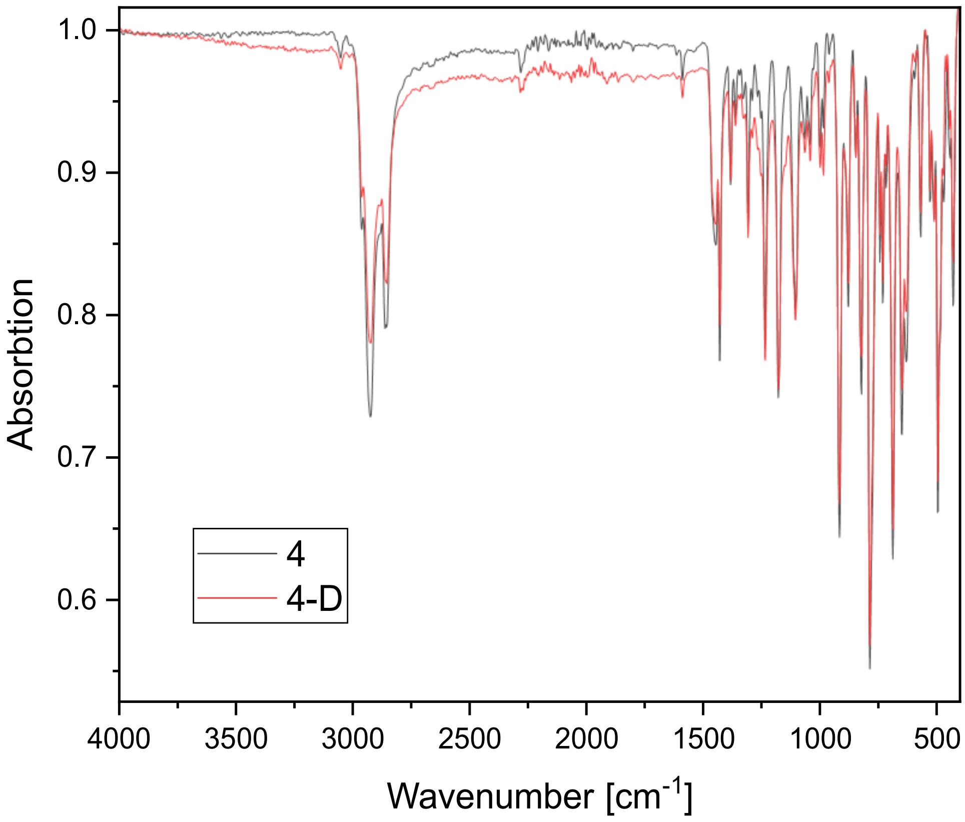

Figure S34. ATR-IR spectrum of **4** and **4-D** overlayered at ambient temperature.

4. Reversibility of Ethylene activation by 2

To investigate the reversibility of the ethylene activation of **2** generating **3**:

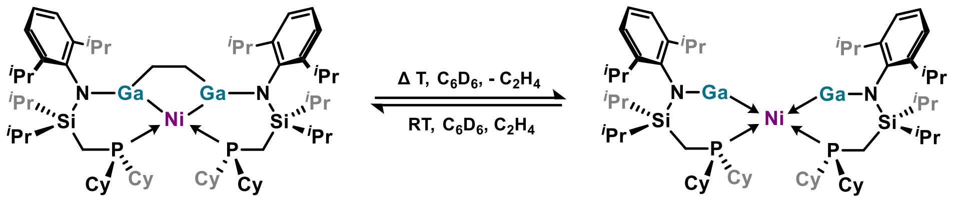

3 **2**

A gas tight Young NMR tube was loaded with 25.0 mg (0.021 mmol) of **2** and 0.5 mL of C_6D_6. Following the NMR tube was pressurized with ethylene 1.5 bar, through a single freeze-pump-thaw cycle. The NMR was then shaken for 15 minutes at room temperature. Then the mixture was degassed by three freeze-pump-thaw cycles and subsequently the reaction mixture was frozen in an -80 °C acetone/dry ice bath until insertion into a preheated NMR spectrometer (298K). Following starting from 298K to 373K in 6 temperature steps a $^{31}P\{^1H\}$ NMR was recorded. The sample was always hold at the given temperature for 10 minutes, before recording the NMR.

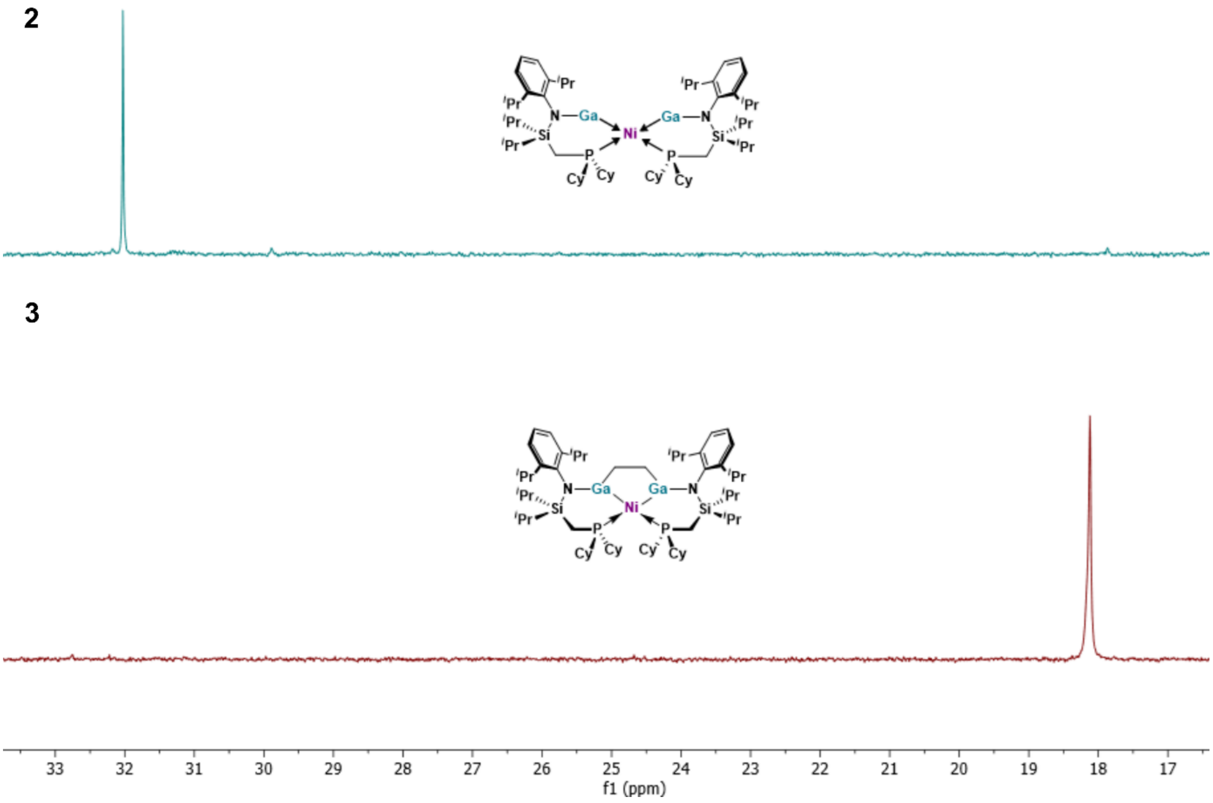

Figure S35. Stacked $^{31}P\{^{1}H\}$ NMR spectrum (162 MHz, C_6D_6) of top **2**, and bottom **3**.

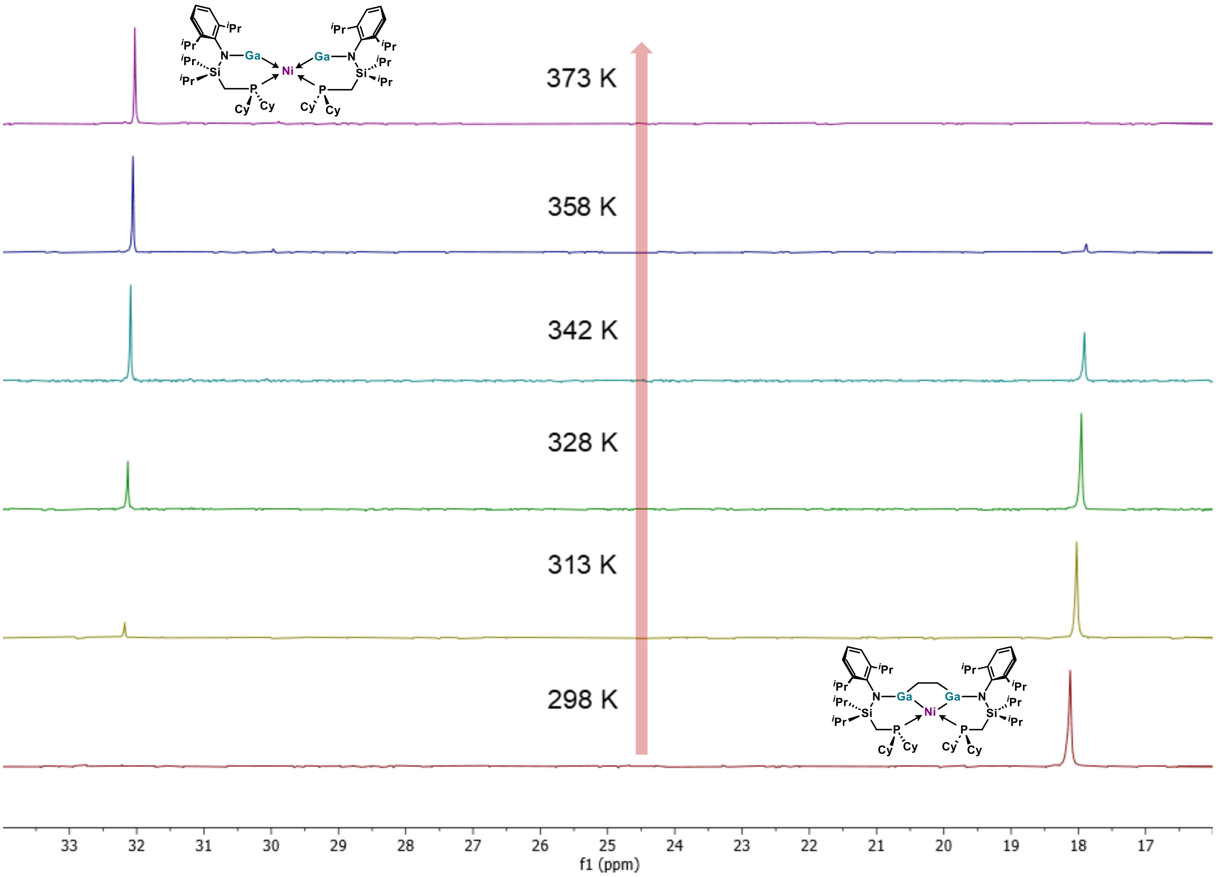

Figure S36. Stacked $^{31}P\{^{1}H\}$ NMR spectrum (162 MHz, C_6D_6) of **3**, liberating ethylene and generating **2**.

5. Ethane liberation from 4, regenerating 2

To investigate the ethane liberation reaction from **4**, generating **2**:

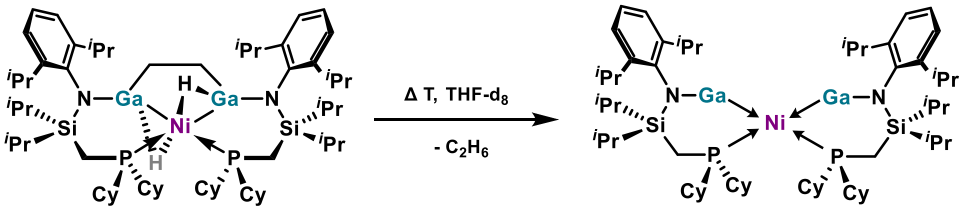

A gas tight Young's NMR tube was loaded with 25.0 mg (0.021 mmol) of **4** and 0.5 mL of THF-*d*8. The a ^{1}H and ^{31}P{^{1}H} NMR was recorded (*top*, Fig. S37). The NMR was then heated to 50°C for 6 hours, and again a ^{1}H and ^{31}P{^{1}H} NMR recorded (Fig. S37, *below*), showing complete transformation of **4** to **2**.

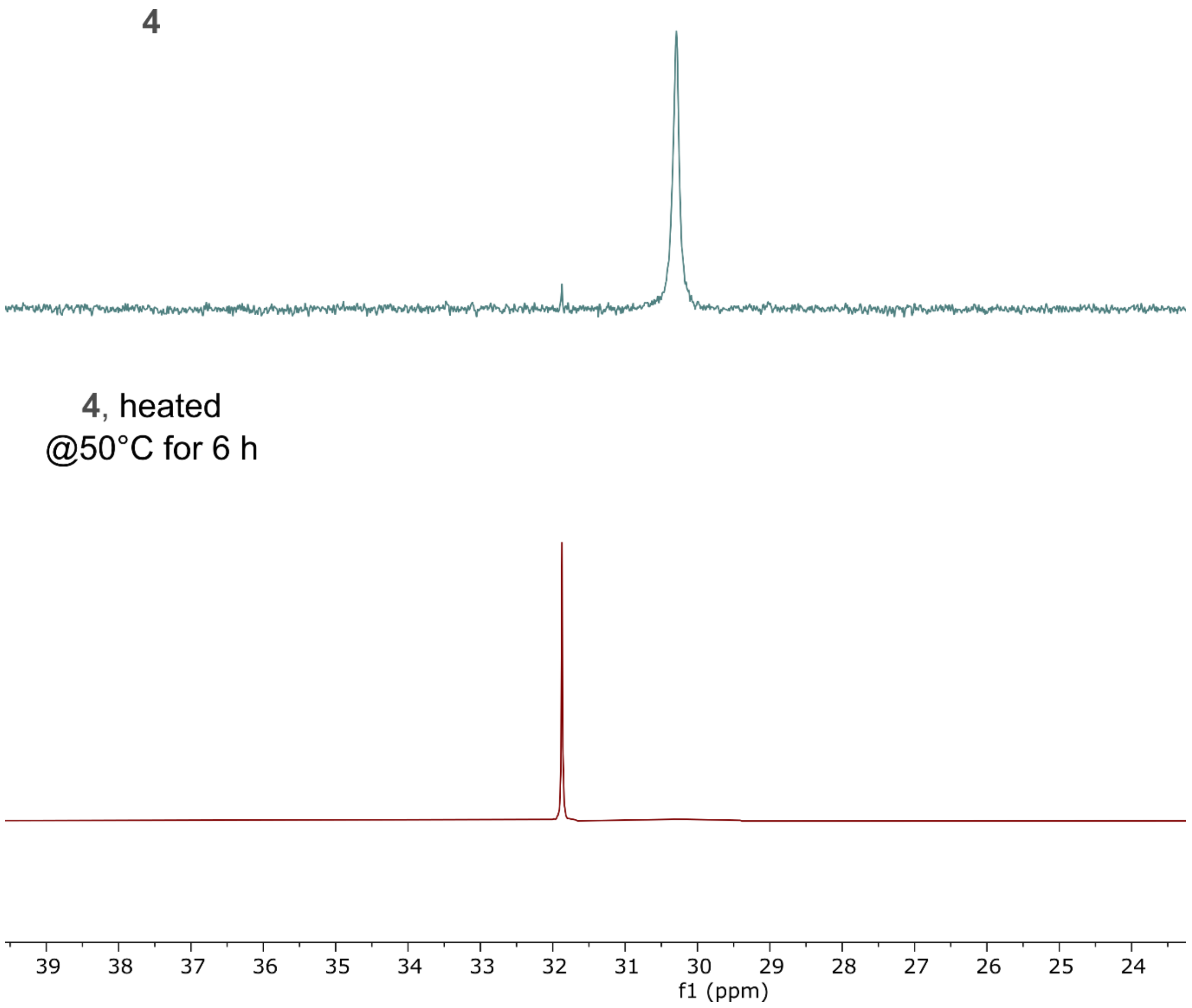

Figure S37. Stacked ^{31}P{^{1}H} NMR spectra (161 MHz, THF-*d*8) of **4** (*top*), and after heating this sample at 50°C for 6 h, generating **2** (*below*).

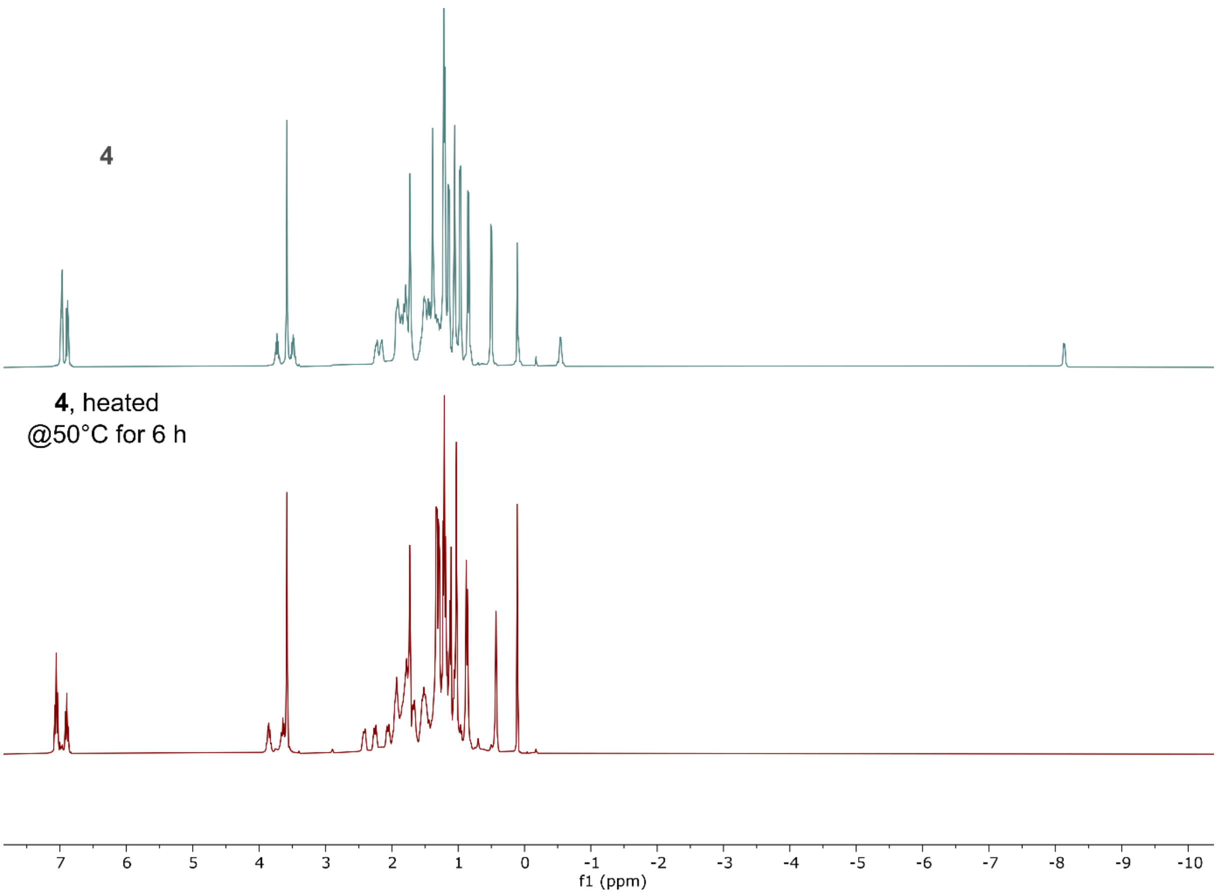

Figure S38. Stacked ^{1}H NMR spectra (161 MHz, THF-*d*8) of **2** (*top*), and after heating this sample at 50°C for 6 h, generating **2** (*below*).

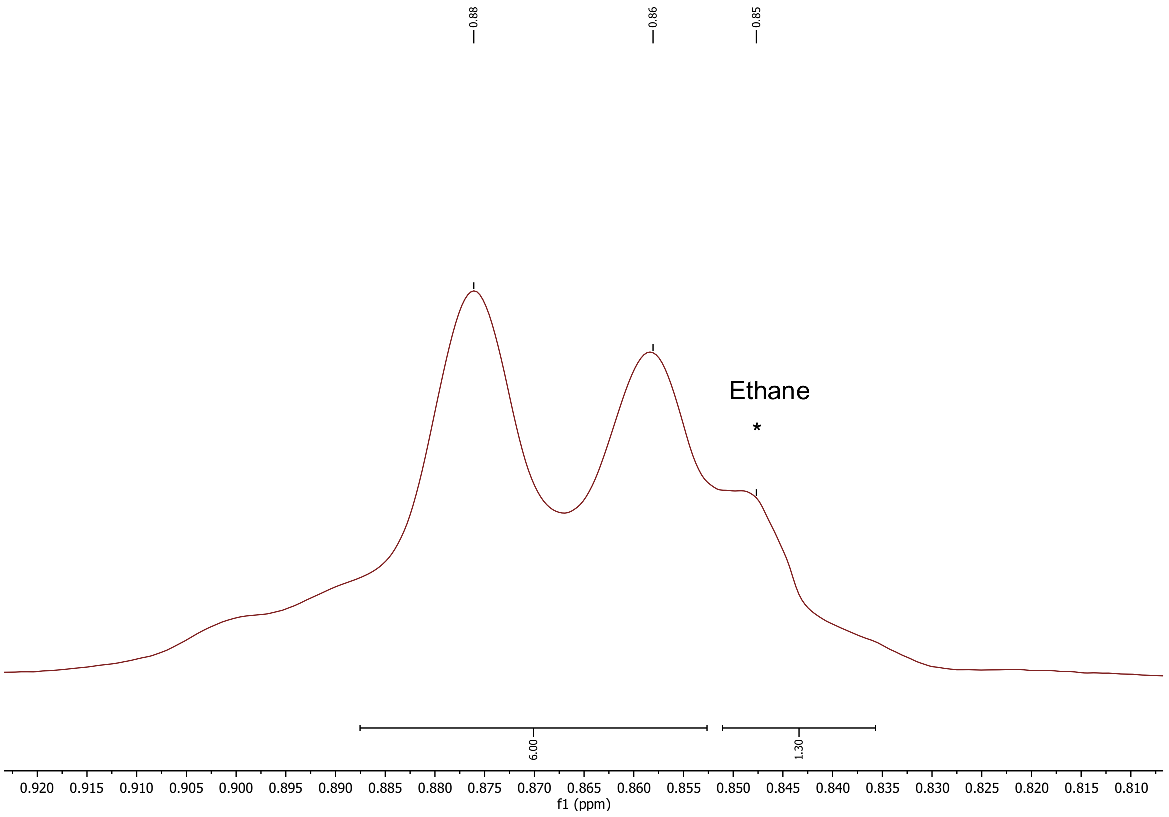

Figure S39. Zoomed area of the ^{1}H NMR spectrum (400 MHz, THF-*d*8) of a solution of **4** heated to 50°C for 6h, liberating ethane and generating **2**. * denotes ethane.

6. Transformation of 4 regenerating 3

To investigate the transformation of **4** to **3** on addition of ethylene gas:

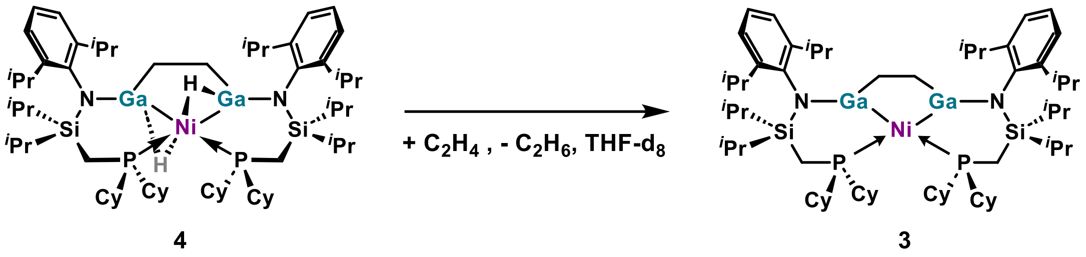

A gas tight Young's NMR tube was loaded with 25.0 mg (0.021 mmol) of **4** and 0.5 mL of THF-*d*8. The the NMR tube was pressurized with ethylene 1.5 bar, through a single freeze-pump-thaw cycle. A $^{31}P\{^1H\}$ NMR was subsequently recorded, showing complete formation of **3**.

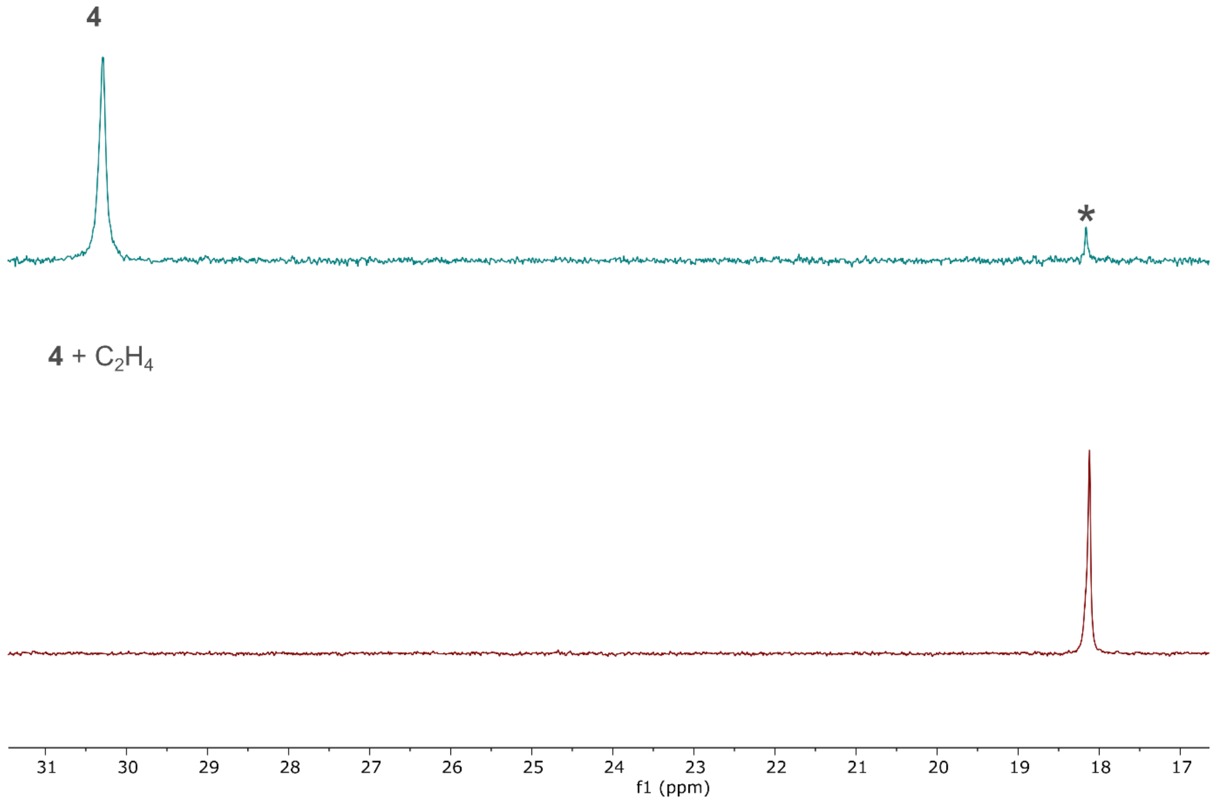

Figure S40. Stacked $^{31}P\{^1H\}$ NMR spectra (161 MHz, THF-*d*8) of **4** (*top*), and after the addition of 1.5 bar ethylene, generating **3** (*bottom*). *indicates small amounts of residual **3**, remaining from the *in-situ* generation of **4**.

7. Consecutive transformation from 2 to 3 to 4

To investigate the transformation from **2** to **3**, and then to **4**, in a single NMR tube:

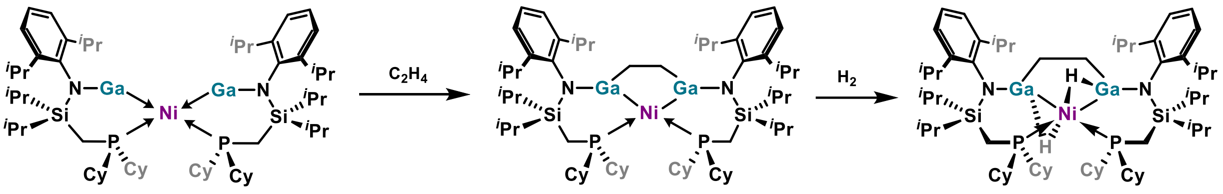

2 **3** **4**

A gas tight Young's NMR tube was loaded with 25.0 mg (0.021 mmol) of **2** and 0.5 mL of C_6D_6, and ^1H and $^{31}P\{^1H\}$ NMR spectra collected (Figs. S42-44, *top*). The NMR tube was then pressurized with 1.5 bar ethylene, through a single freeze-pump-thaw cycle. ^1H and $^{31}P\{^1H\}$ NMR spectra indicated complete formation of **3** (Figs. S42-44, *middle*). The NMR tube was then pressurized with 1.5 bar dihydrogen, through a single freeze-pump-thaw cycle. Subsequently recorded ^1H and $^{31}P\{^1H\}$ NMR spectra demonstrated the complete formation of **4** (Figs. S42-44, *bottom*).

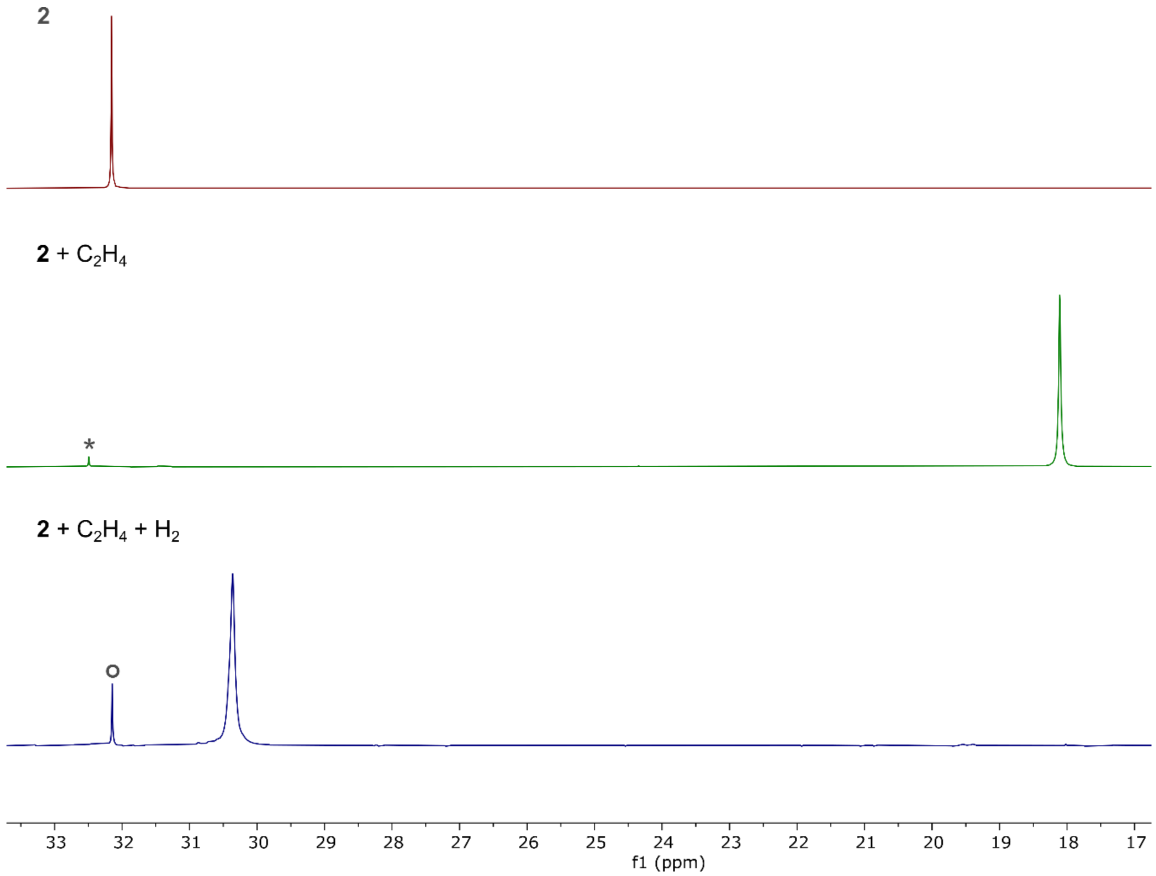

Figure S41. Stacked $^{31}P\{^1H\}$ NMR spectra (161 MHz, C_6D_6) from top to bottom: compound **2**, compound **2** with added ethylene (*e.g.* **3**), and compound **2** with added ethylene and dihydrogen (*e.g.* **4**). *indicates an unknown minor impurity. °indicates small amounts of formed **2**, presumably on elimination of ethane.

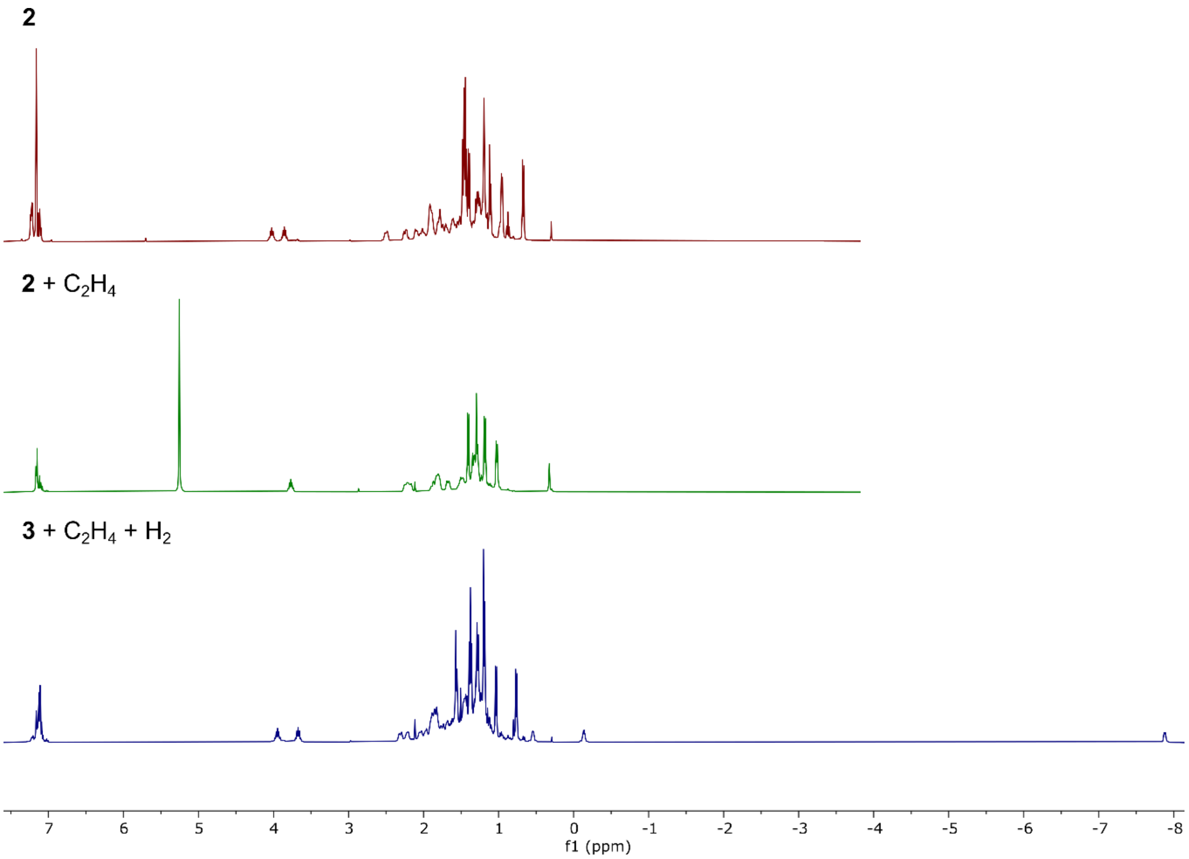

Figure S42. Stacked ^{1}H NMR spectra (400 MHz, C_6D_6) from top to bottom: compound **2**, compound **2** with added ethylene (*e.g.* **3**), and compound **2** with added ethylene and dihydrogen (*e.g.* **4**).

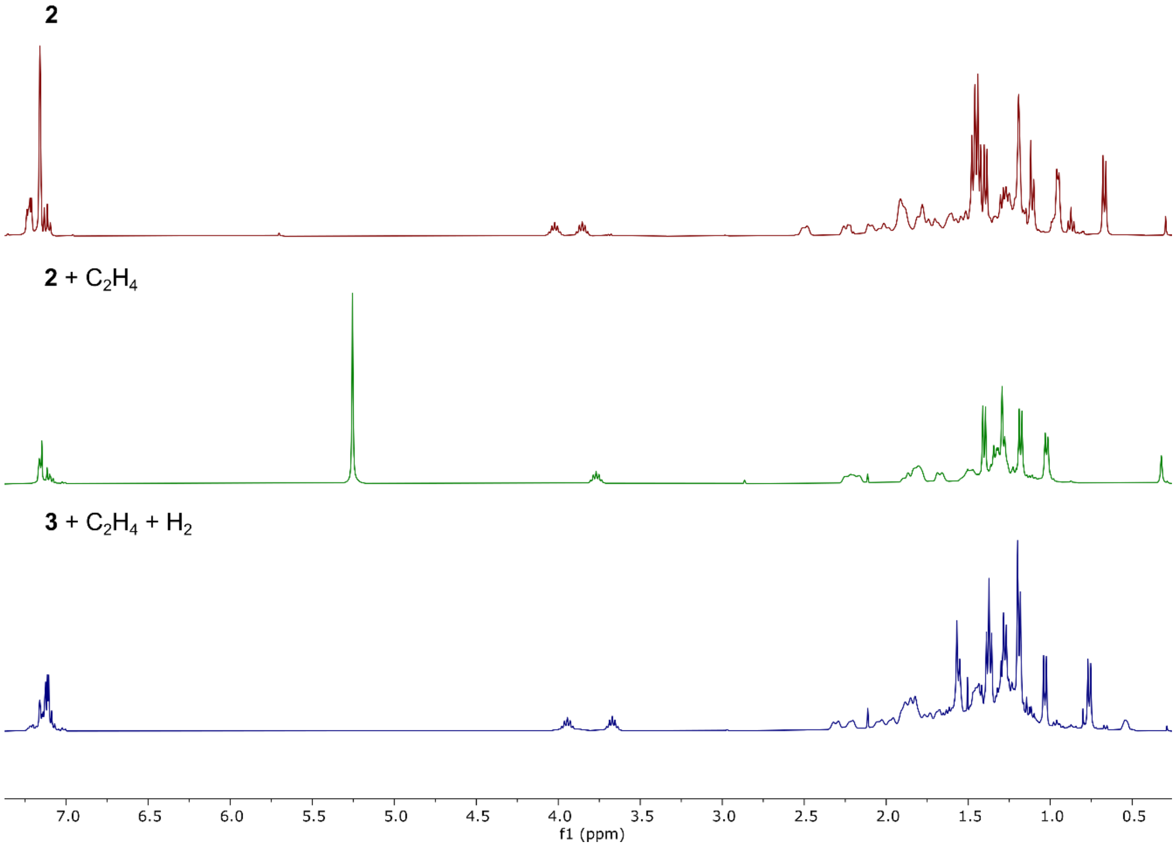

Figure S43. Zoomed and stacked ^{1}H NMR spectra (400 MHz, C_6D_6) from top to bottom: compound **2**, compound **2** with added ethylene (*e.g.* **3**), and compound **2** with added ethylene and dihydrogen (*e.g.* **4**).

8. Eyring-Polanyi Analysis of Ethylene Elimination from 3

To investigate the rate constants, Gibb's free activation energy, activation entropy and activation enthalpy of the ethylene elimination by **3** to **2** at higher temperatures:

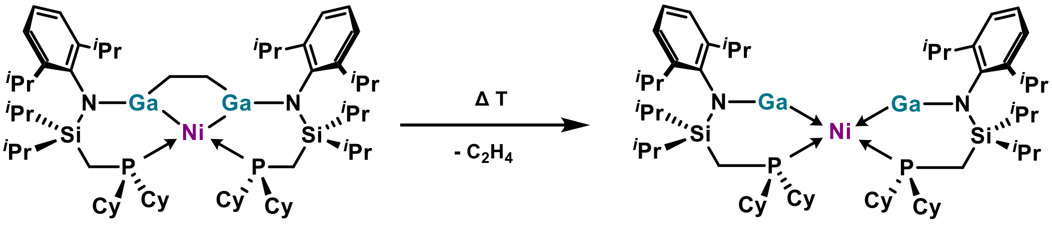

3 **2**

A gas tight Young NMR tube was loaded with 0.5 mL of a stock solution containing 25.0 mg (0.021 mmol) of **2** and 0.025 mmol mesitylene as internal standard in C_6D_6. Following the NMR tube was pressurized with ethylene 1.5 bar, through a single freeze-pump-thaw cycle. The NMR was then shaken for 15 minutes at room temperature. Then the mixture was degassed by three freeze-pump-thaw cycles and subsequently the reaction mixture was frozen in an -80 °C acetone/dry ice bath until insertion into a preheated NMR spectrometer (323 - 328 K). The temperature inside the probe head was externally calibrated *via* monitoring the OH/CH_2 peak separation in an 80 wt% glycol/DMSO-d_6 solution, prior and after the measurement.[6] Subsequently, the reaction was monitored continuously via ^{1}H NMR spectroscopy (NS = 16 Scans, DS = 2 Scans, D = 1 second) for 75 minutes (110 NMR spectra). The progress was evidenced by integration of known resonances compared to the internal standard in the sample. This was carried out at in at five different temperatures for **3** between 323 – 338 K, respectively.

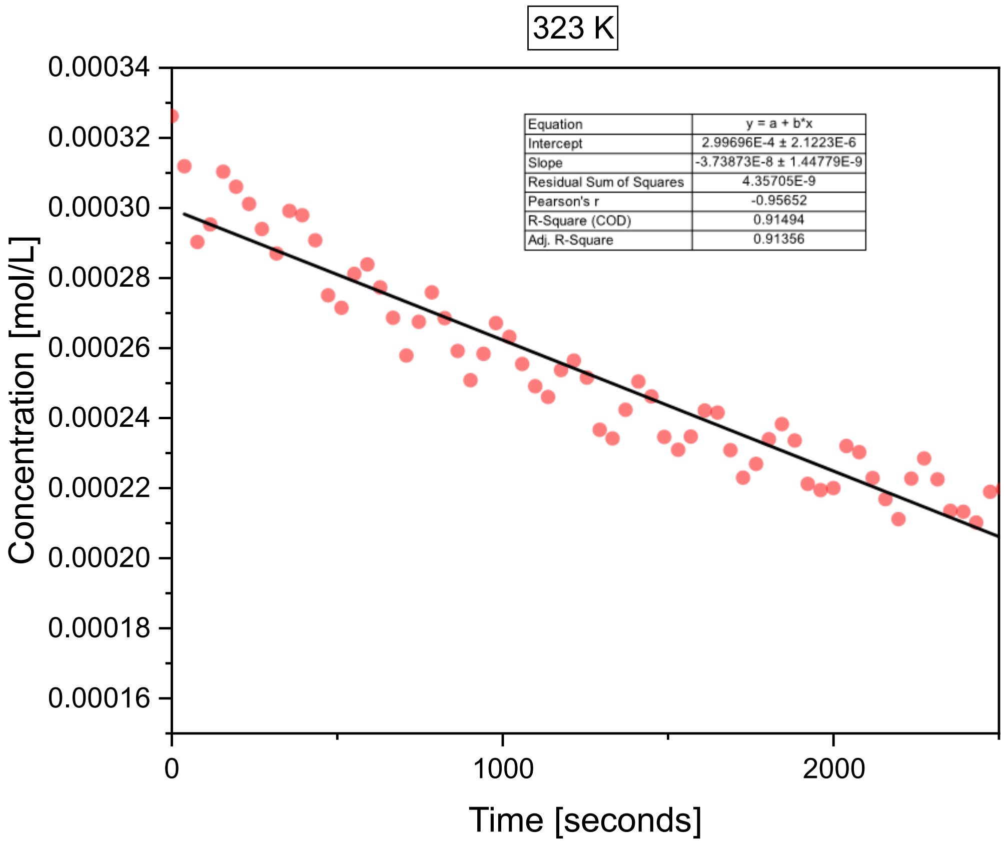

Figure S44. Kinetic data of ethylene elimination from **3** at 323 K.

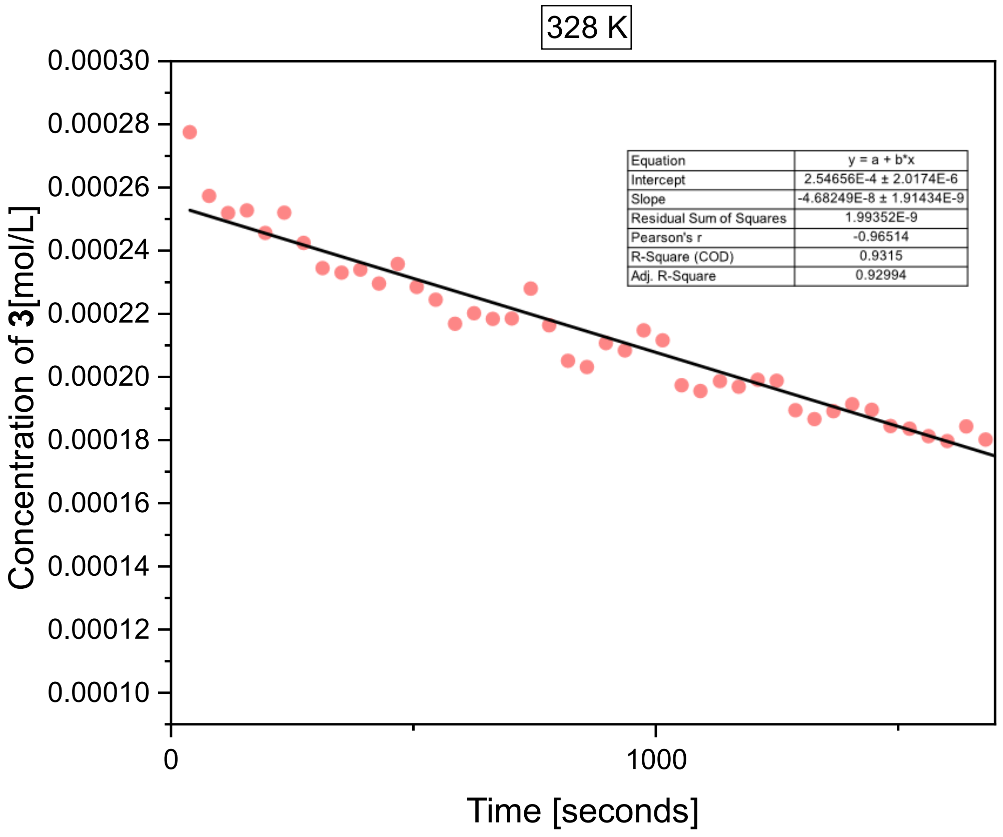

Figure S45. Kinetic data of ethylene elimination from **3** at 328 K.

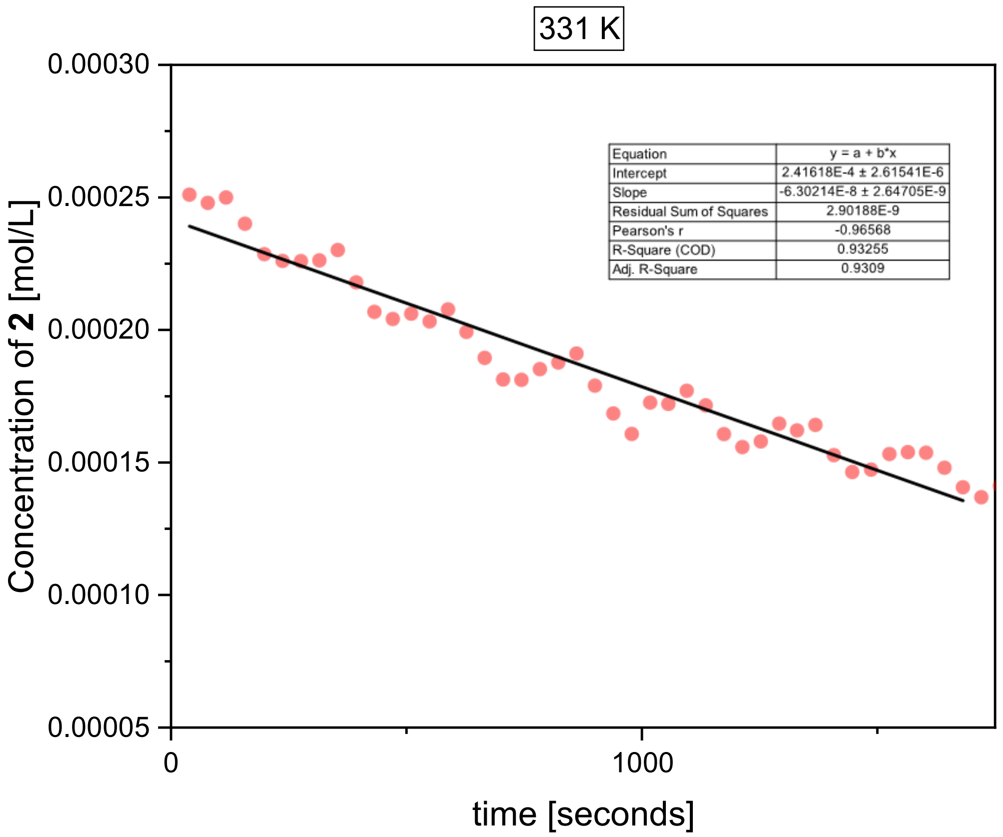

Figure S46. Kinetic data of ethylene elimination from **3** at 331 K.

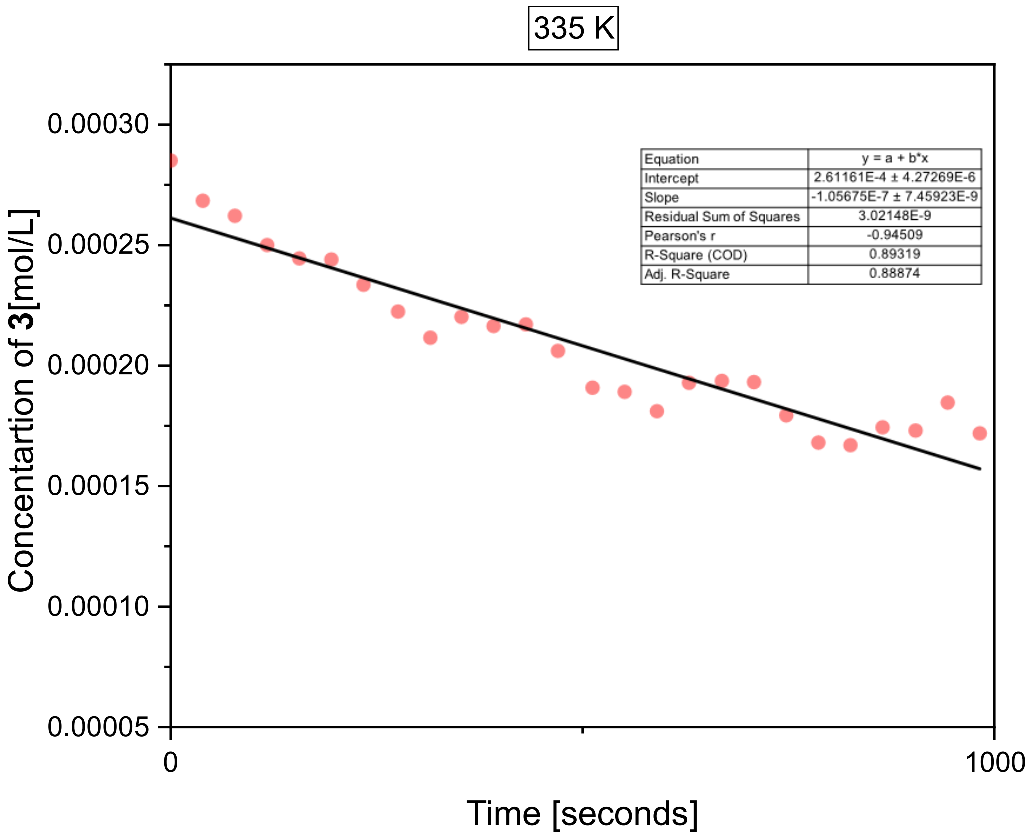

Figure S47. Kinetic data of ethylene elimination from **3** at 335 K.

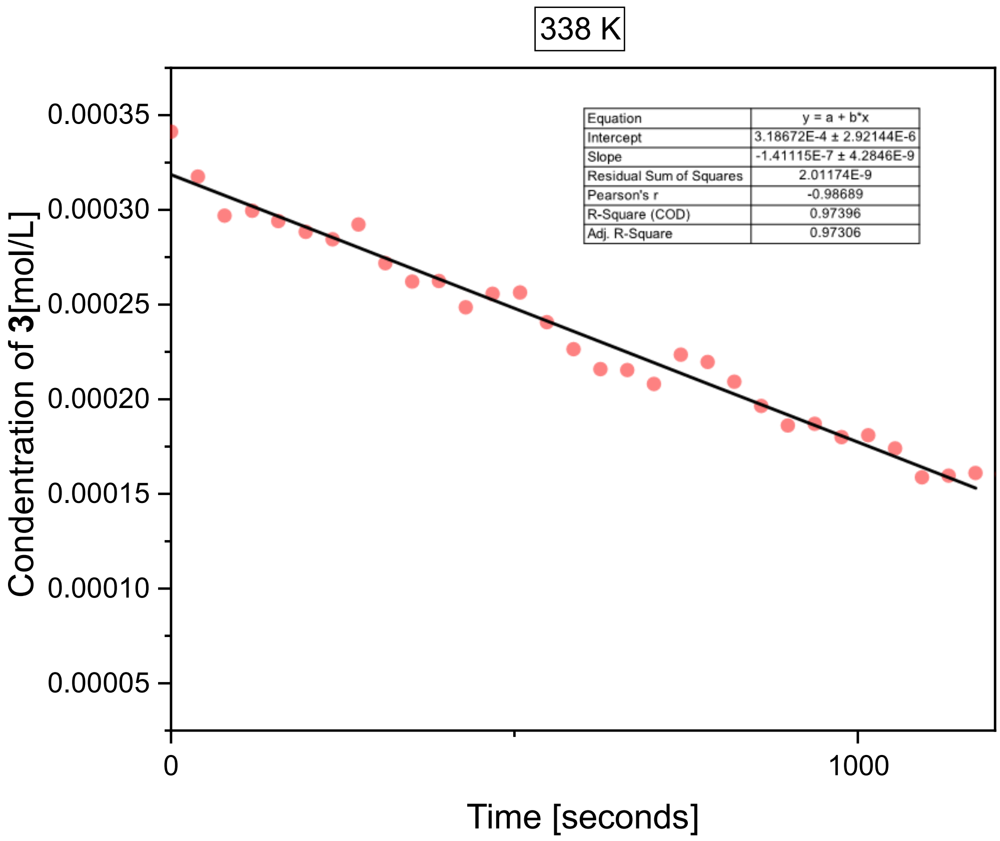

Figure S48. Kinetic data of ethylene elimination from **3** at 338 K.

Table S1. Table of constants derived from kinetic experiments of the ethylene elimination from **3**.

Temperature [K]	***1/T [1/K]***	***k^{H}_{Obs} [mol*s/L]***	***ln(k^{H}_{Obs}/T) [mol*s/L*K]***
323.25	0.00309	$3.73*10^{-8}$	-22.882
327.74	0.00305	$4.68*10^{-8}$	-22.669
330.74	0.00302	$6.32*10^{-8}$	-22.378
335.23	0.00298	$1.05*10^{-7}$	-21.884
338.23	0.00296	$1.41*10^{-7}$	-21.597

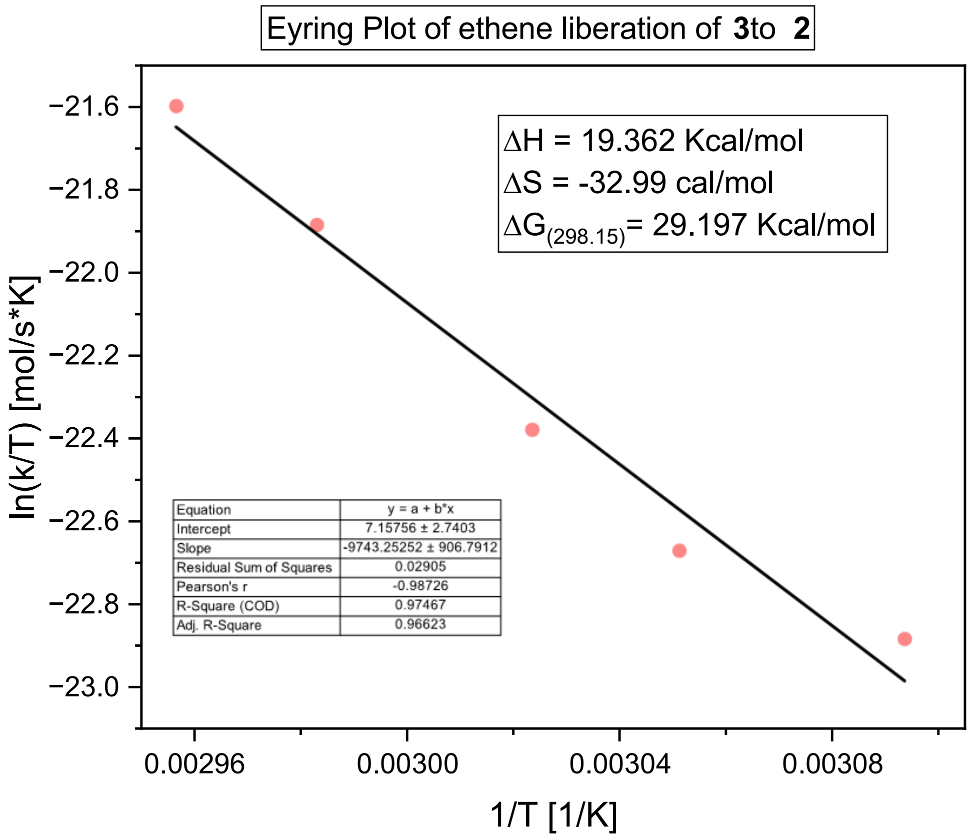

Figure S49. Eyring plot for the ethylene liberation process from **3**, using data from Table S1.

Thermodynamic Parameters

To determine the thermodynamic parameters, from the Eyring Plot the following mathematical relations were used. b = slope, a = y-intercept, k_b = Boltzman constant, h = Plank constant, R = ideal gas constant, k = rate constant, $\Delta H^‡$ = enthalpy of activation, $\Delta S^‡$ = entropy of activation, $\Delta G^‡$ = Gibbs energy of activation, T = temperature.

$$(13)\ k = \frac{k_b * T}{h} * e^{\frac{\Delta S^‡}{R}} * e^{-\frac{\Delta H^‡}{RT}}$$

$$(14)\ \Delta H^‡ = -b * R$$

$$(15)\ \Delta S^‡ = (a - \ln\left(\frac{k_b}{h}\right)) * R$$

$$(16)\ \Delta G^‡ = \Delta H^‡ - T\Delta S^‡$$

Table S2. Thermodynamic data for ethylene elimination from **3**, derived from the Eyring plot in Fig. S49.

	For 3 to 2
$\Delta H^{‡}$ **[Kcal/Mol]**	19.362
$\Delta S^{‡}$ **[cal/Mol*K]**	-32.99
$\Delta G^{‡}_{298}$ **[Kcal/Mol]**	29.197

9. Eyring-Polanyi Analysis of Ethane Elimination of 4/4-D

To investigate the rate constants, Gibb's free activation energy, activation entropy and activation enthalpy of the ethane elimination by **4** and **4-D** to **2** at higher temperatures:

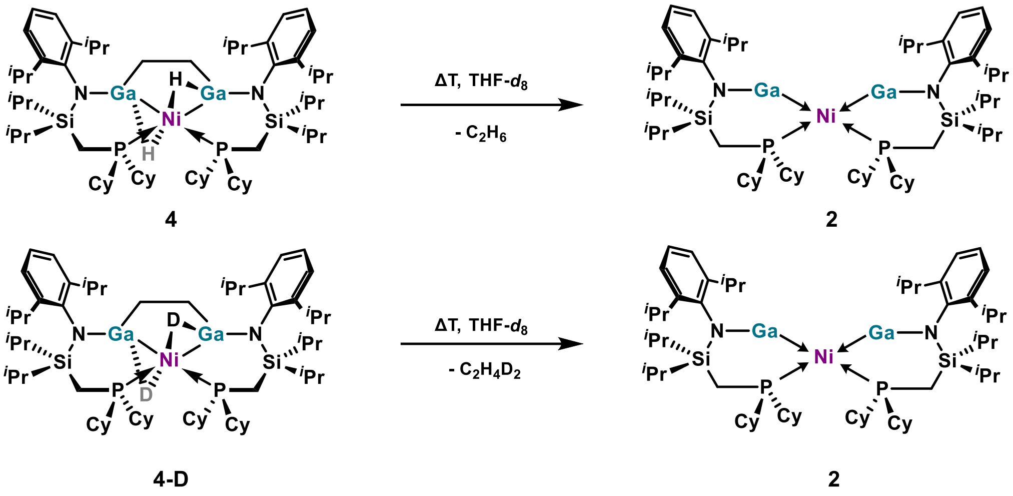

A gas tight Young NMR tube was loaded with 0.5 mL of a freshly prepared stock solution containing 10.0 mg (0.008 mmol) of **4**/**4-D** and 0.025 mmol mesitylene as internal standard in THF-d_8. Following the mixture was cooled in an -80 °C acetone/dry ice bath until insertion into a preheated NMR spectrometer (313 - 350 K). The temperature inside the probe head was externally calibrated *via* monitoring the OH/CH_2 peak separation in an 80 wt% glycol/DMSO-d_6 solution, prior and after the measurement.[6] Subsequently, the reaction was monitored continuously via ^{1}H NMR spectroscopy (NS = 16 Scans, DS = 2 Scans, D = 1 second) for 75 minutes (110 NMR spectra). The progress was evidenced by integration of known resonances compared to the internal standard in the sample. This was carried out at in at five different temperatures for **4**/**4-D** between 313 – 350 K, respectively.

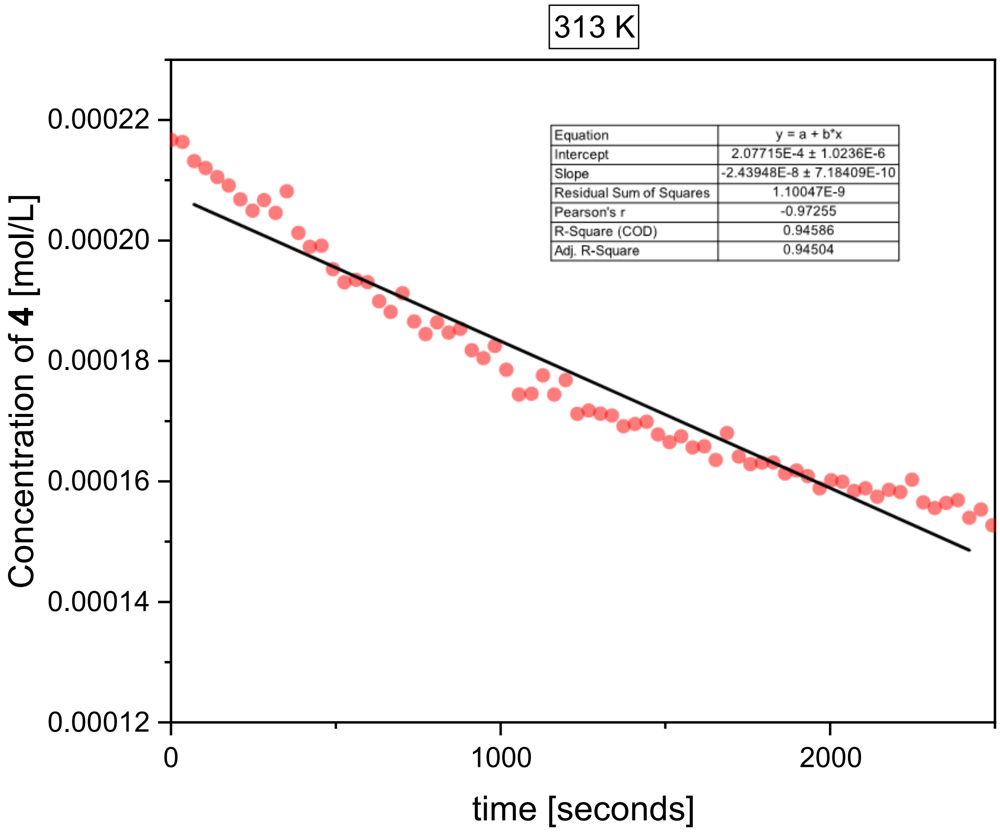

Figure S50. Kinetic data of ethane Elimination of **4** to **2** at 313 K.

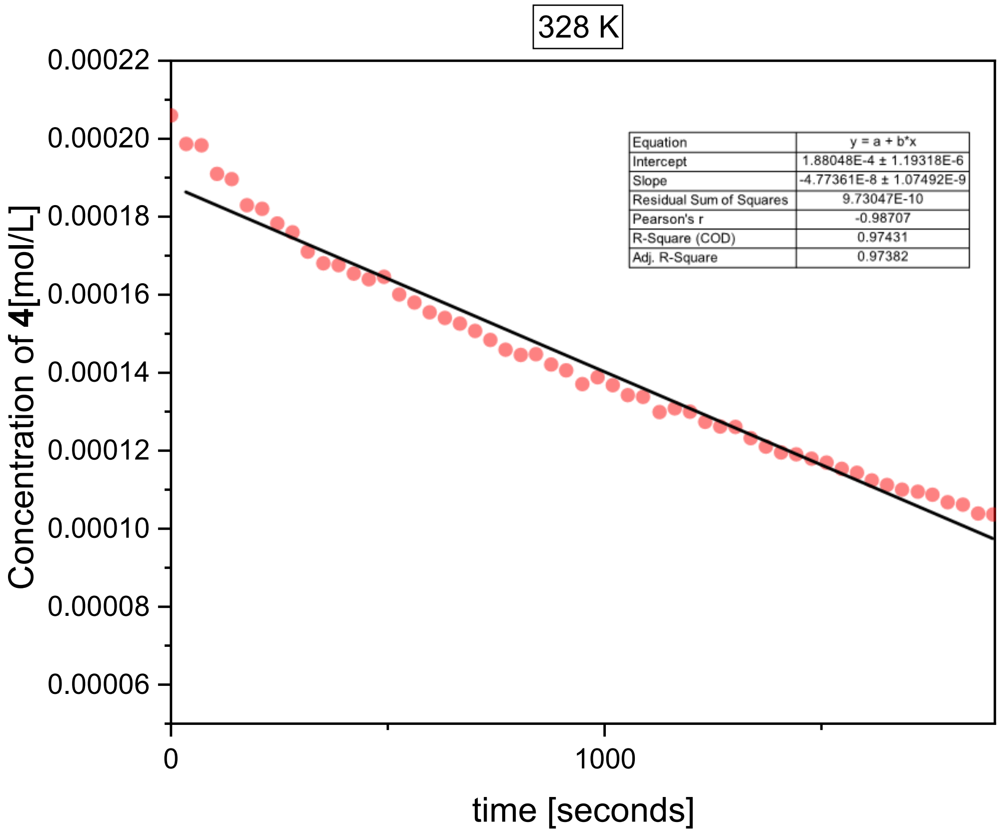

Figure S51. Kinetic data of ethane Elimination of **4** to **2** at 328 K.

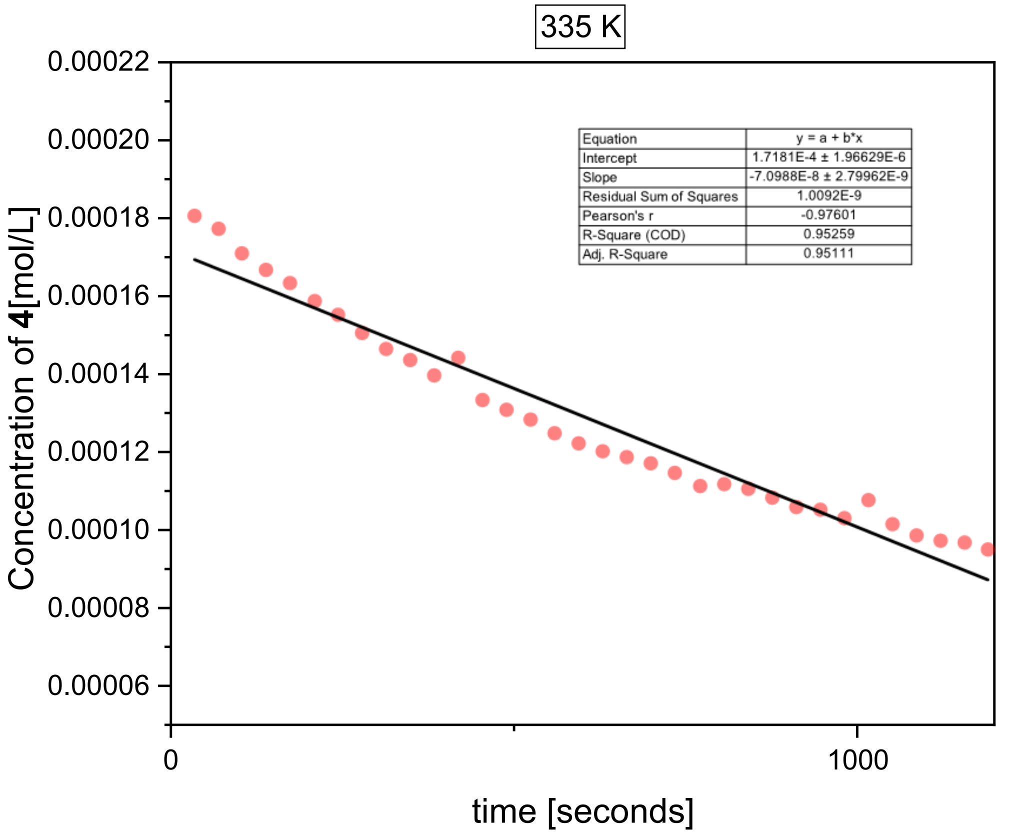

Figure S52. Kinetic data of ethane Elimination of **4** to **2** at 335 K.

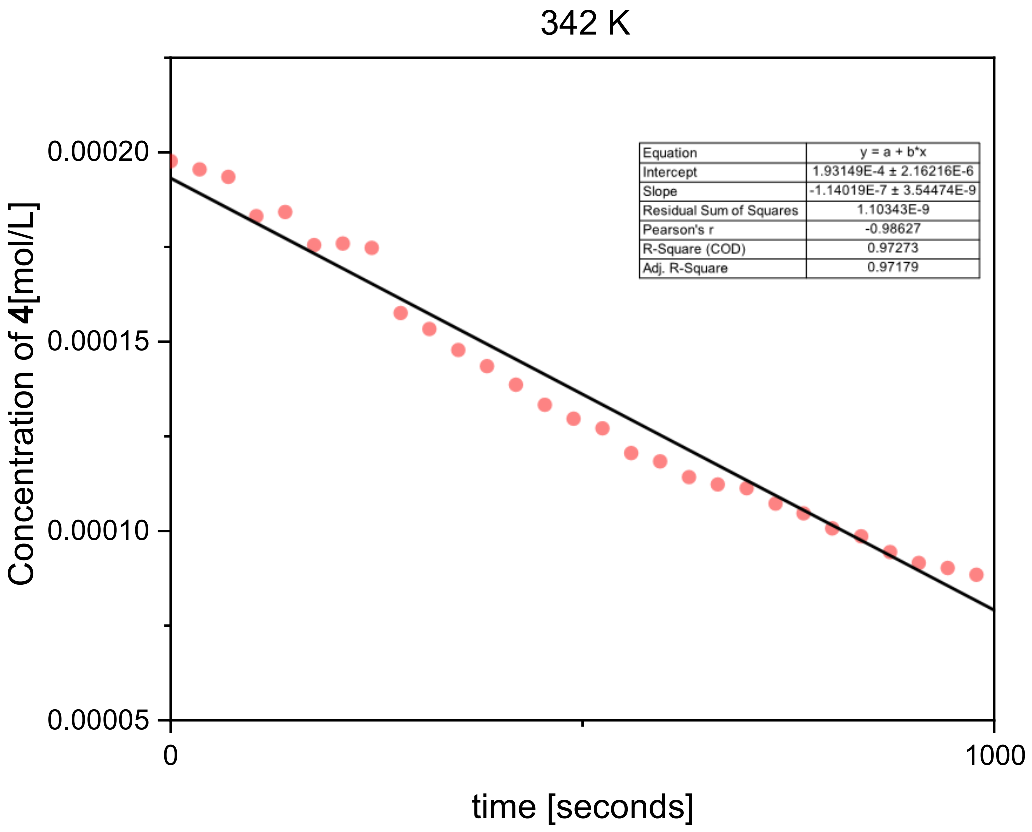

Figure S53. Kinetic data of ethane Elimination of **4** to **2** at 342 K.

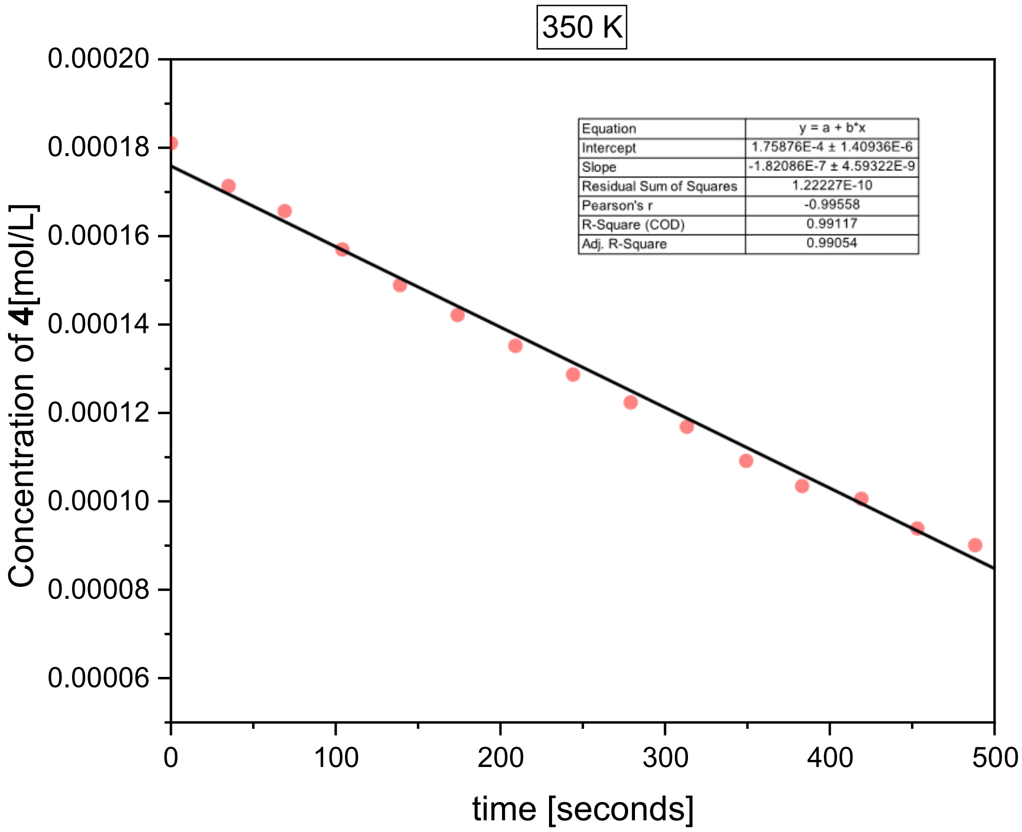

Figure S54. Kinetic data of ethane Elimination of **4** to **2** at 350 K.

Table S3. Table of constants derived from kinetic experiments of the ethane elimination from **4**..

Temperature [K]	***1/T [1/K]***	***k^{H}_{Obs} [mol*s/L]***	***ln(k^{H}_{Obs}/T) [mol*s/L*K]***
312.77	0.0032	$2.44*10^{-8}$	-23.274
327.75	0.00305	$4.77*10^{-8}$	-22.649
335.23	0.00298	$7.09*10^{-8}$	-22.276
342.72	0.00292	$1.14*10^{-7}$	-21.823
350.21	0.00286	$1.82*10^{-7}$	-21.377

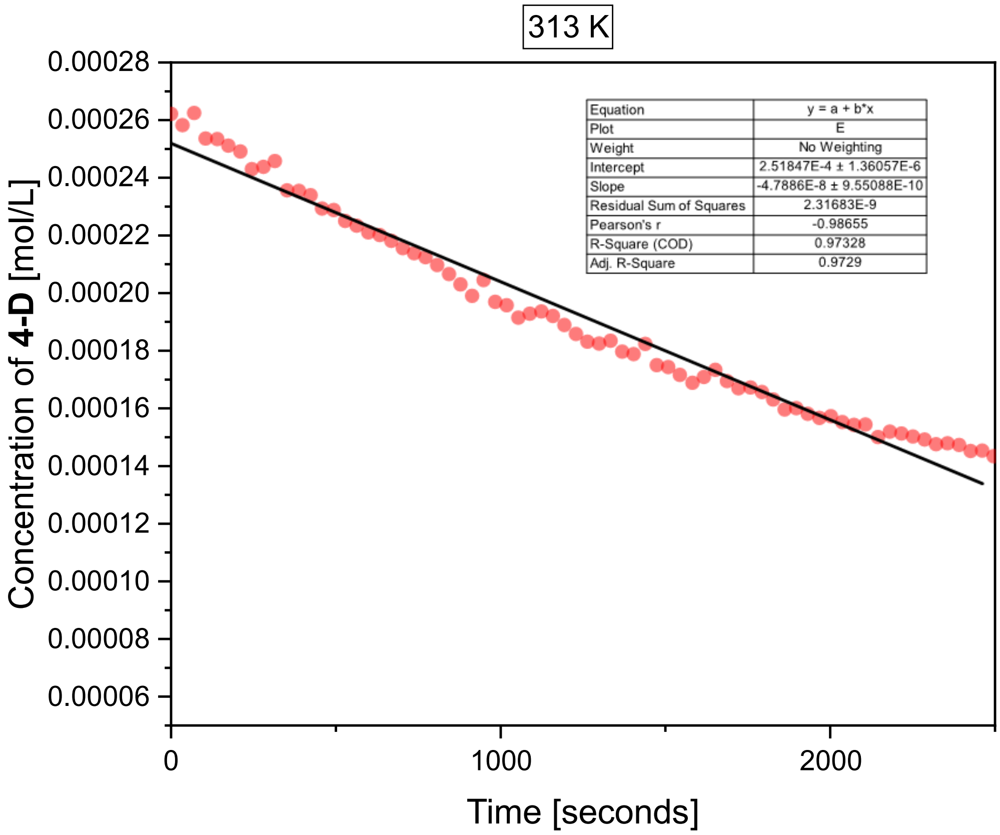

Figure S55. Kinetic data of ethane elimination from **4-D** at 313 K.

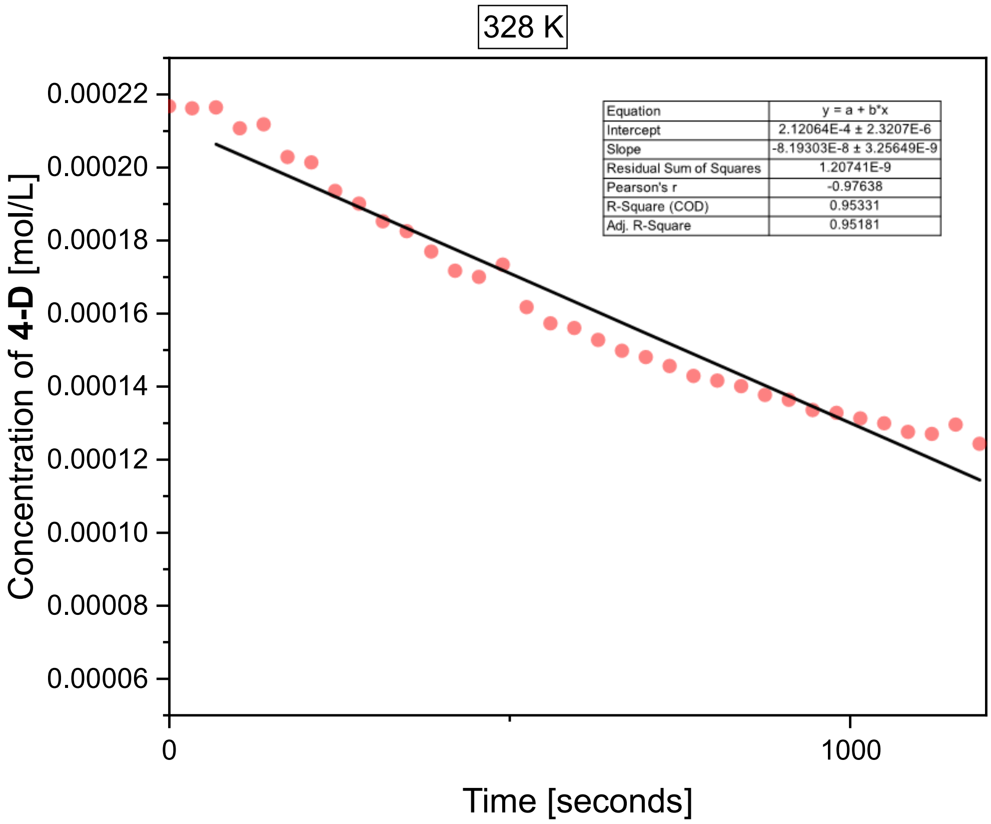

Figure S56. Kinetic data of ethane elimination from **4-D** at 328 K.

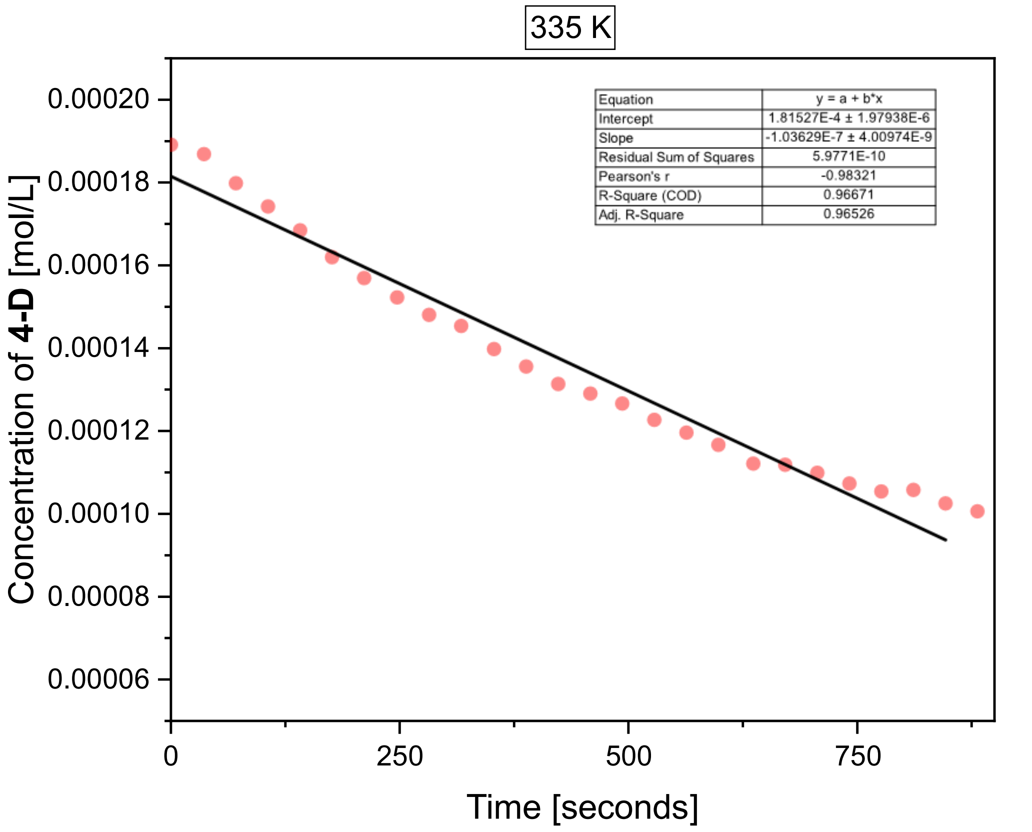

Figure S57. Kinetic data of ethane elimination from **4-D** at 335 K.

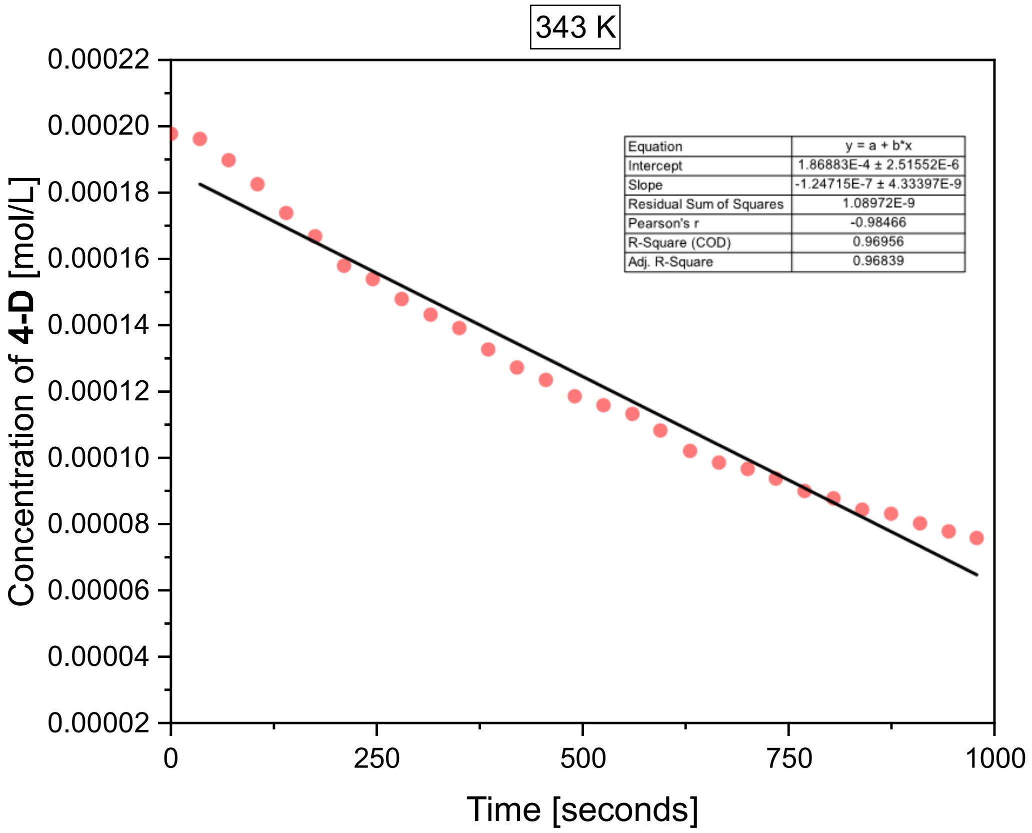

Figure S58. Kinetic data of ethane elimination from **4-D** at 343 K.

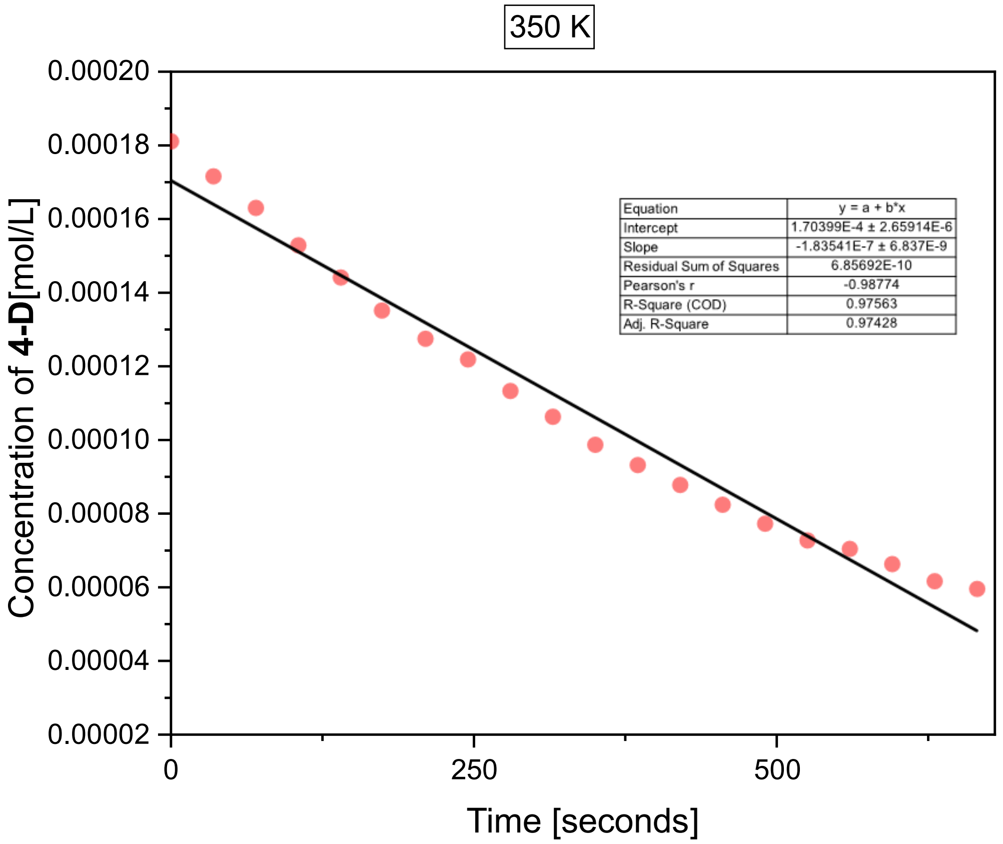

Figure S59. Kinetic data of ethane elimination from **4-D** at 350 K.
Table S4. Table of constants derived from kinetic experiments of the ethane elimination from **4-D**.

Temperature [K]	***1/T [1/K]***	***k^{H}_{Obs} [mol*s/L]***	***ln(k^{H}_{Obs}/T) [mol*s/L*K]***
312.77	0.0032	$4.79*10^{-8}$	-22.600
327.75	0.00305	$8.19*10^{-8}$	-22.109
335.23	0.00298	$1.04*10^{-7}$	-21.898
342.72	0.00292	$1.25*10^{-7}$	-21.734
350.21	0.00286	$1.83*10^{-7}$	-21.372

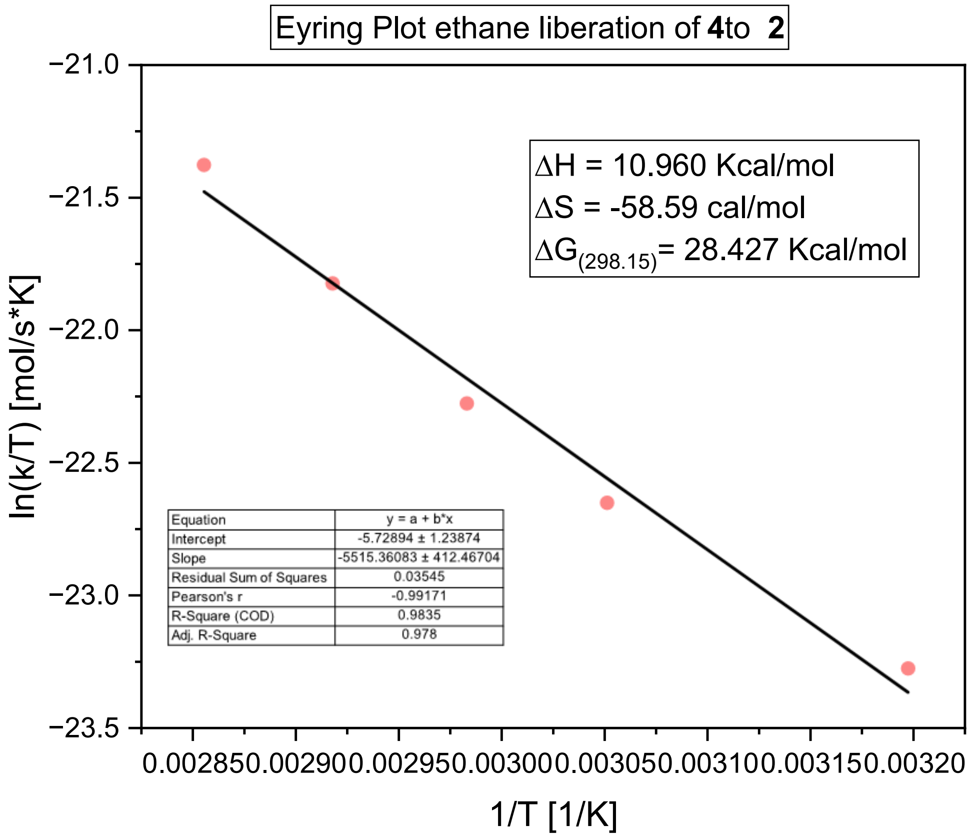

Figure S60. Eyring plot for the ethane liberation process from **4**, using data from Table S3.

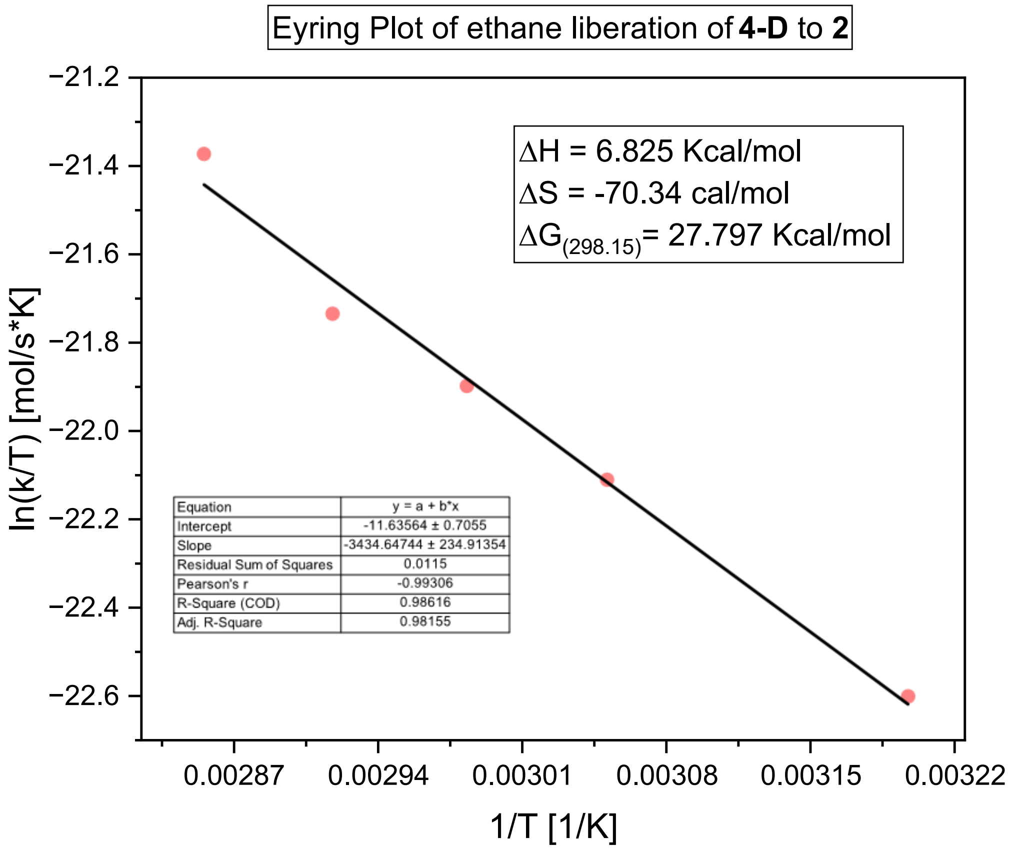

Figure S61. Eyring plot for the ethane liberation process from **4-D,** using data from Table S4.

Thermodynamic Parameters

To determine the thermodynamic parameters, from the Eyring Plot the following mathematical relations were used. b = slope, a = y-intercept, k_b = Boltzman constant, h = Plank constant, R = ideal gas constant, k = rate constant, $\Delta H^{‡}$ = enthalpy of activation, $\Delta S^{‡}$ = entropy of activation, $\Delta G^{‡}$ = Gibbs energy of activation, T = temperature.

$$k = \frac{k_b * T}{h} * e^{\frac{\Delta S^{‡}}{R}} * e^{-\frac{\Delta H^{‡}}{RT}}$$

$$\Delta H^{‡} = -b * R$$

$$\Delta S^{‡} = (a - \ln\left(\frac{k_b}{h}\right)) * R$$

$$\Delta G^{‡} = \Delta H^{‡} - T\Delta S^{‡}$$

Table S5. Thermodynamic data for the elimination of (D_n-)ethane from **4**, taken from the Eyring plot in Fig. S60 and S61.

	For 4-H₂	***For 4-D₂***
$\Delta \boldsymbol{H}^{‡}$ **[Kcal/Mol]**	10.960	6.825
$\Delta \boldsymbol{S}^{‡}$ **[cal/Mol*K]**	-58.59	-70.34
$\Delta \boldsymbol{G}^{‡}_{298}$ **[Kcal/Mol]**	28.427	27.797

Calculation of KIE at room temperature

$$KIE = \frac{k^H}{k^D} = e^{\frac{(\Delta G^{D‡} - \Delta G^{H‡})}{RT}} = e^{\frac{(-630\,[\frac{cal}{mol}])}{298.15R}} = \mathbf{0.35}$$

10. Eyring-Polanyi Analysis of Hydrogenation catalysis utilising 2

The kinetic kid “X206 man on the moon operator” was used to investigate the rate constants, Gibb’s free activation energy, activation entropy, activation enthalpy, and kinetic isotope effect of the catalytic hydrogenation of cyclopentene by **2**.

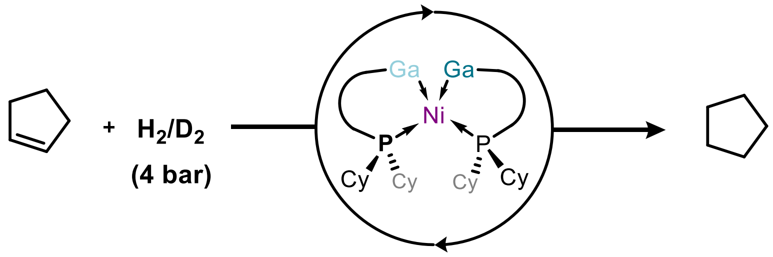

This system allows one to track gas uptake over time in a closed system. The conversion of cyclopentene to cyclopentane (*e.g.* hydrogenation rate) was determined by the consumption of H_2/D_2. For this, a GC-MS vial was loaded with 0.7 mL of a freshly prepared stock solution containing **2** (4.8 mg, 0.004 mmol) and cyclopentene (13.63 mg, 0.2 mmol) in toluene. The GC vial was put in a high-pressure reactor of the “Man on the Moon” company, and the reactor was connected to the gas burette (*e.g.* gas reservoir). This was charged with 10 atm of H_2/D_2 pressure, and the reaction set at a constant pressure of 4 atm. The s^{-1} pressure decrease in the gas reservoir was monitored, until no further gas uptake was observed. This was carried out at five different temperatures between 295 – 318 K, leading to the data given in Figs. S62 to S66.

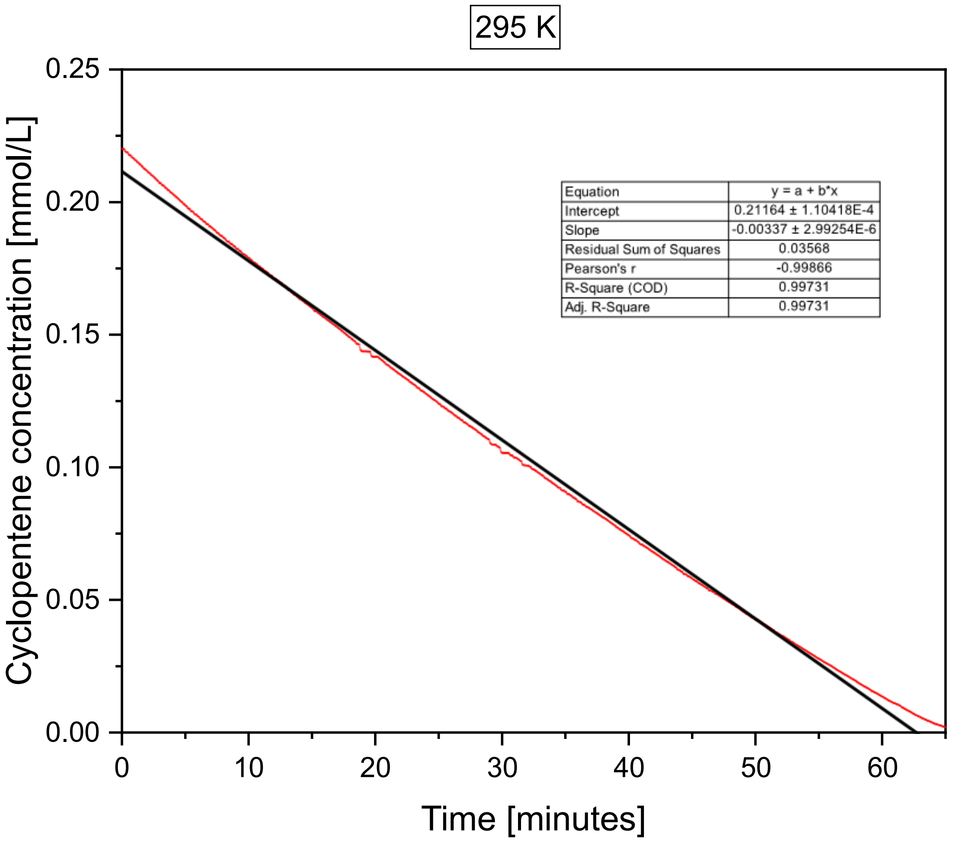

Figure S62. Kinetic data for the hydrogenation of cyclopentene by **2** at 295 K.

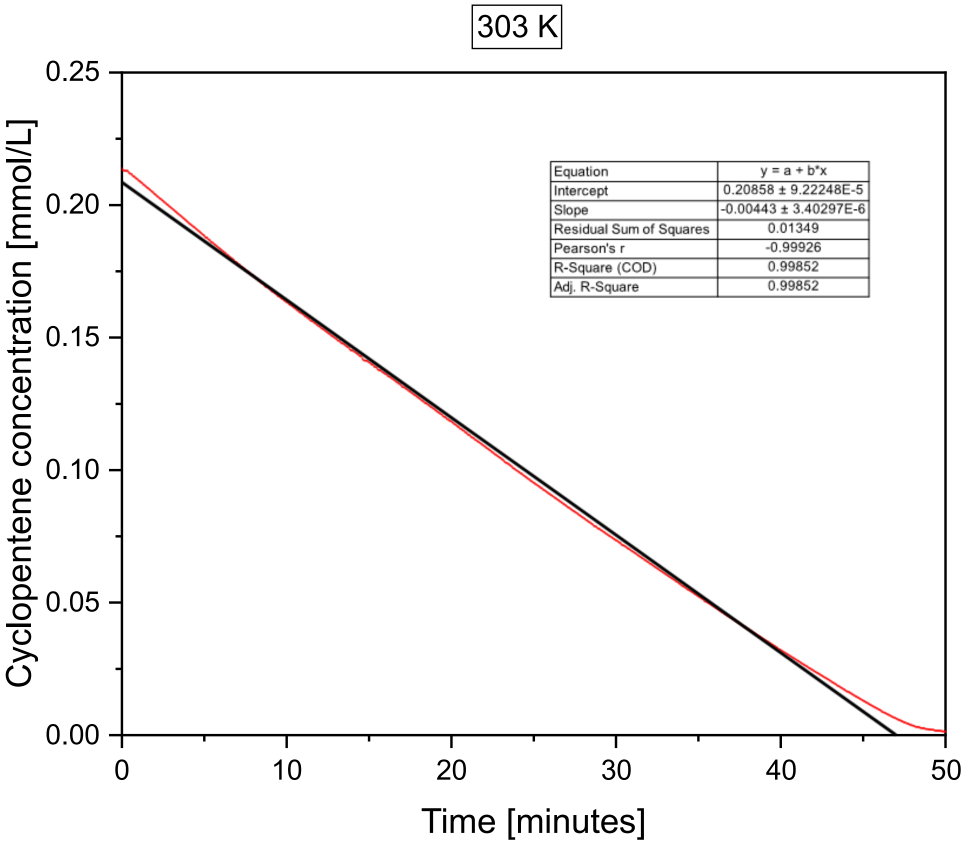

Figure S63. Kinetic data for the hydrogenation of cyclopentene by **2** at 303 K.

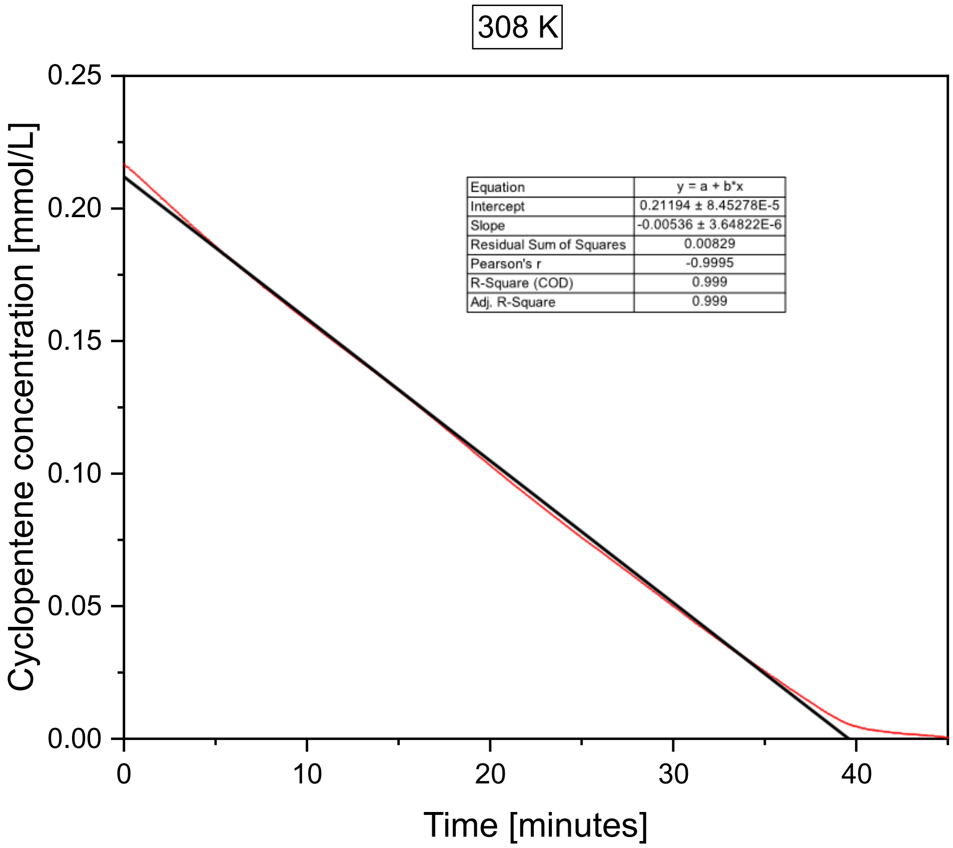

Figure S64. Kinetic data for the hydrogenation of cyclopentene by **2** at 308 K.

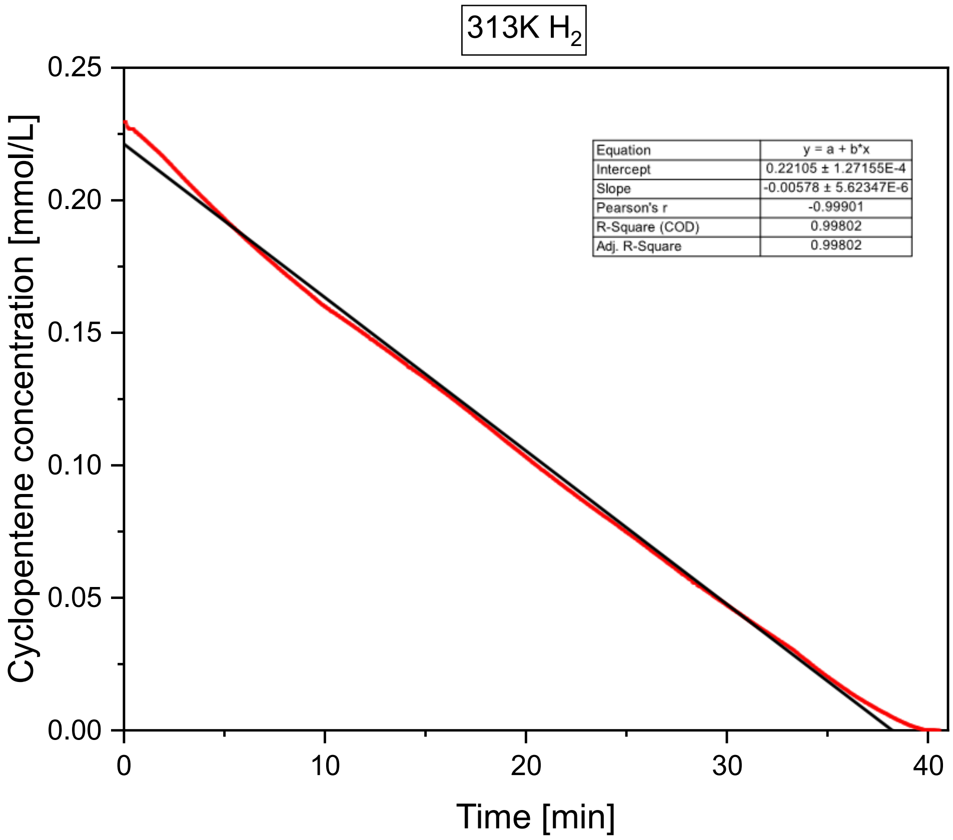

Figure S65. Kinetic data for the hydrogenation of cyclopentene by **2** at 313 K.

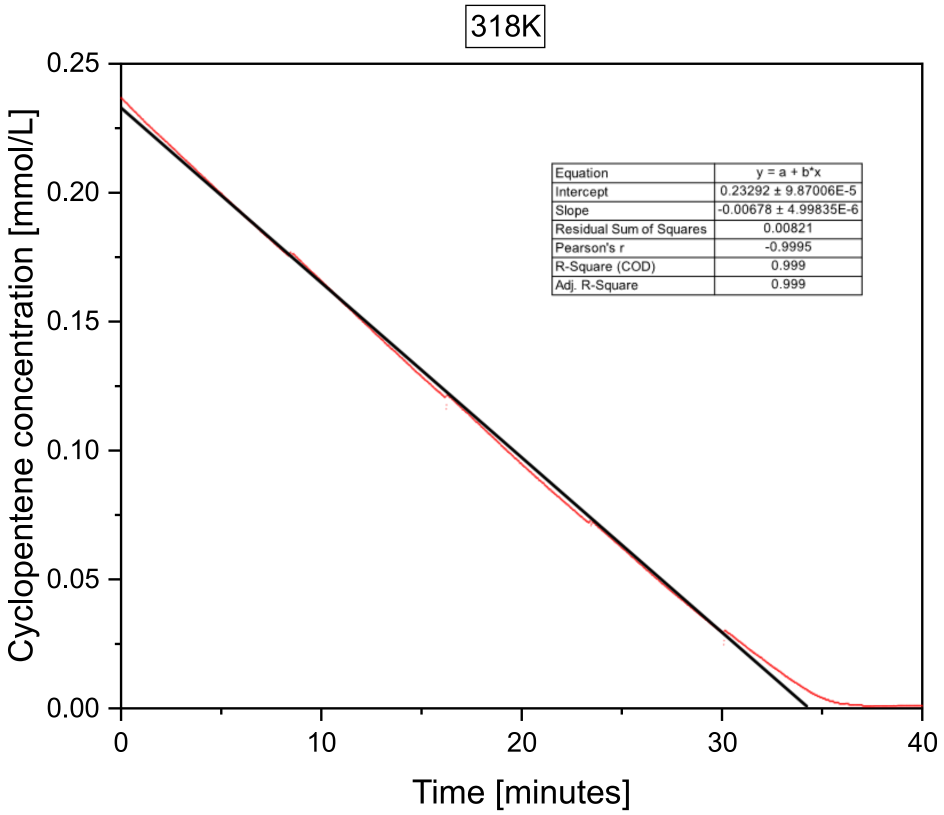

Figure S66. Kinetic data for the hydrogenation of cyclopentene by **2** at 318 K.

Table S6. Table of constants derived from kinetic experiments for the hydrogenation of cyclopentene by **2**.

Temperature [K]	***1/T [1/K]***	***k^{H}_{Obs} [mmol*min/L]***	***ln(k^{H}_{Obs}/T) [mol*s/L*K]***
295.15	0.00339	$3.37*10^{-3}$	-11.380
303.15	0.00330	$4.43*10^{-3}$	-11.134
308.15	0.00325	$5.36*10^{-3}$	-10.959
313.15	0.00319	$5.78*10^{-3}$	-10.900
318.15	0.00314	$6.78*10^{-3}$	-10.756

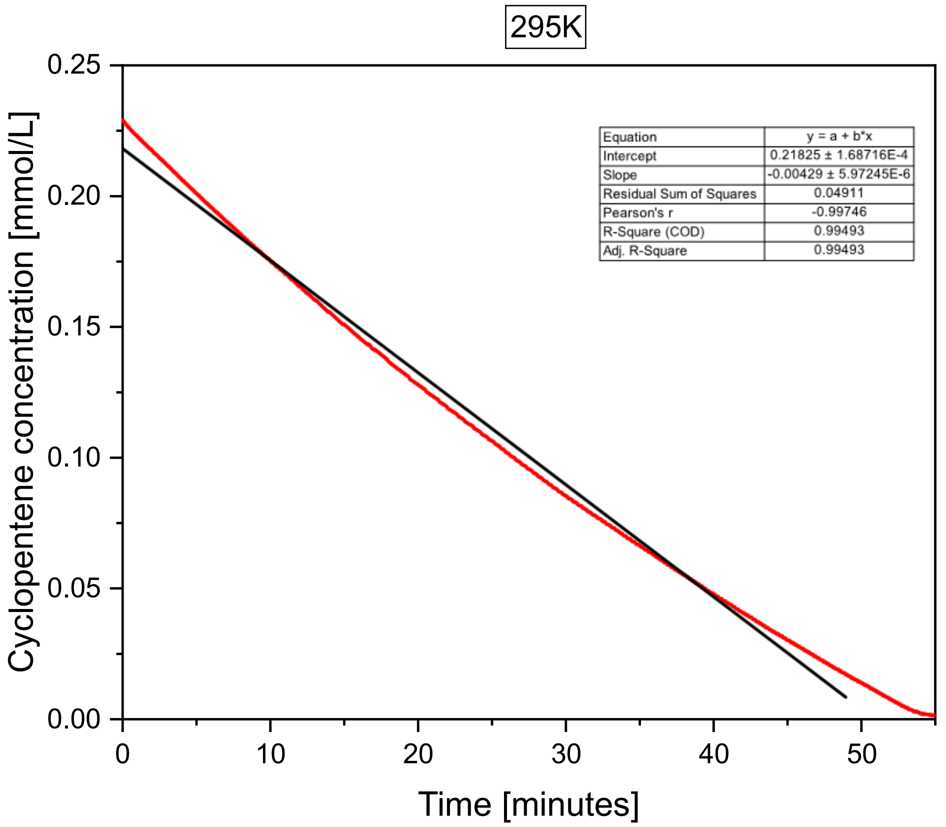

Figure S67. Kinetic data for deuteration of cyclopentene by **2** at 295 K.

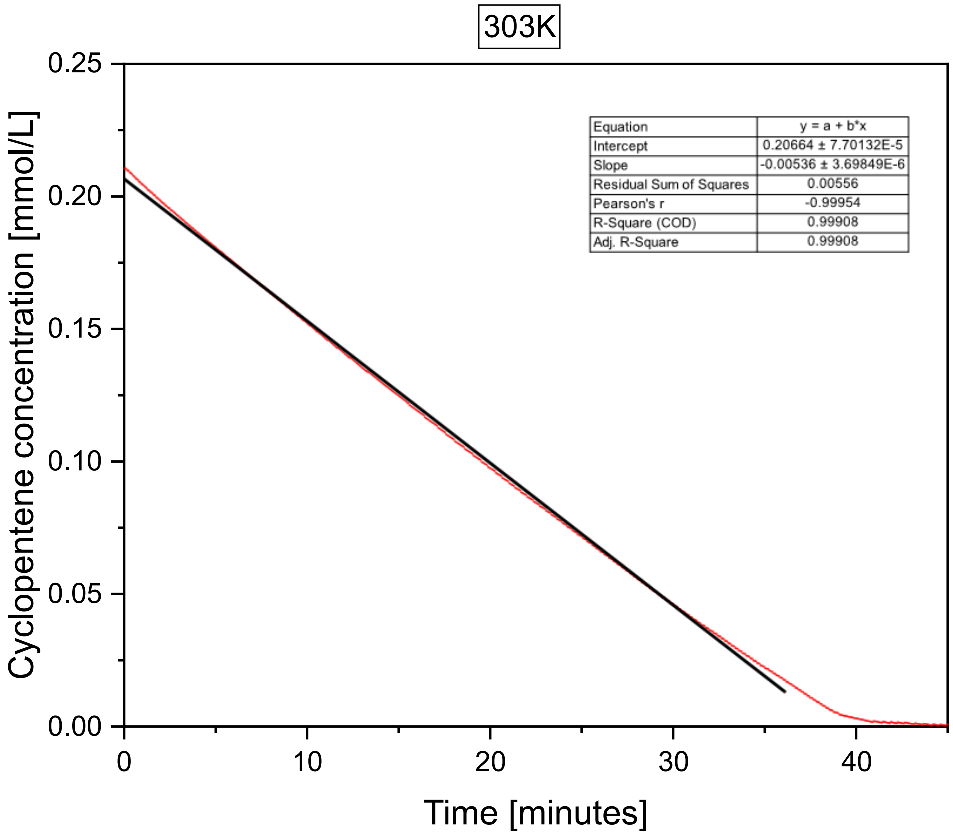

Figure S68. Kinetic data for deuteration of cyclopentene by **2** at 303 K.

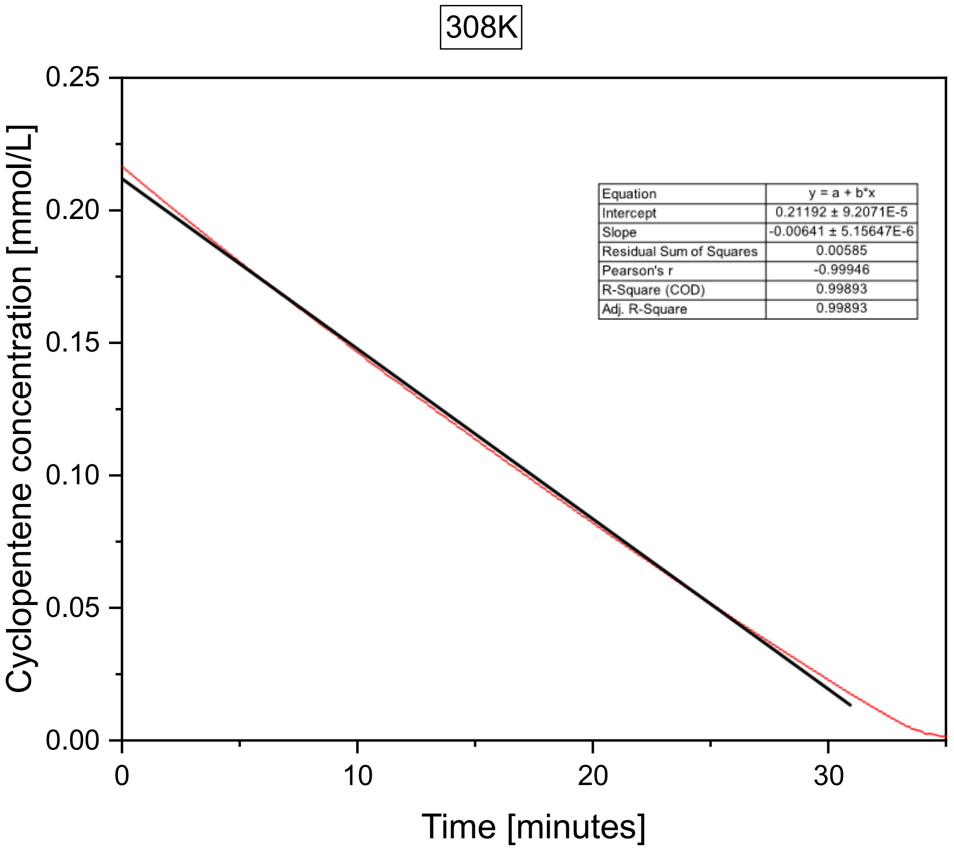

Figure S69. Kinetic data for deuteration of cyclopentene by **2** at 308 K.

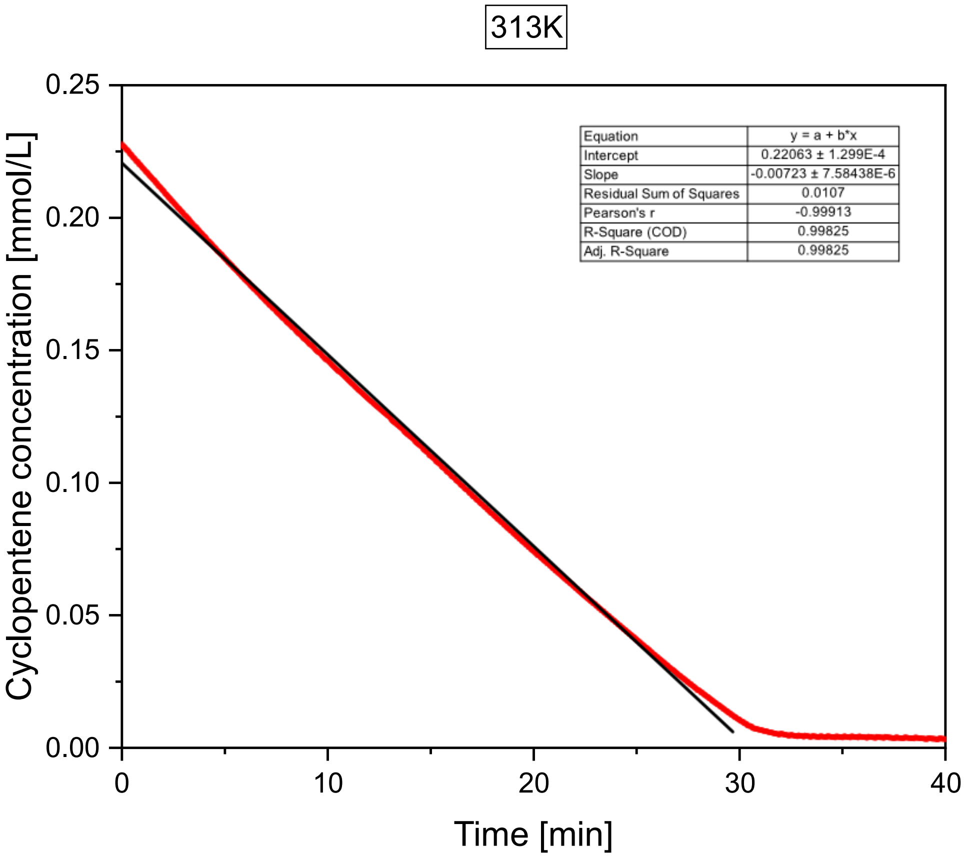

Figure S70. Kinetic data for deuteration of cyclopentene by **2** at 313 K.

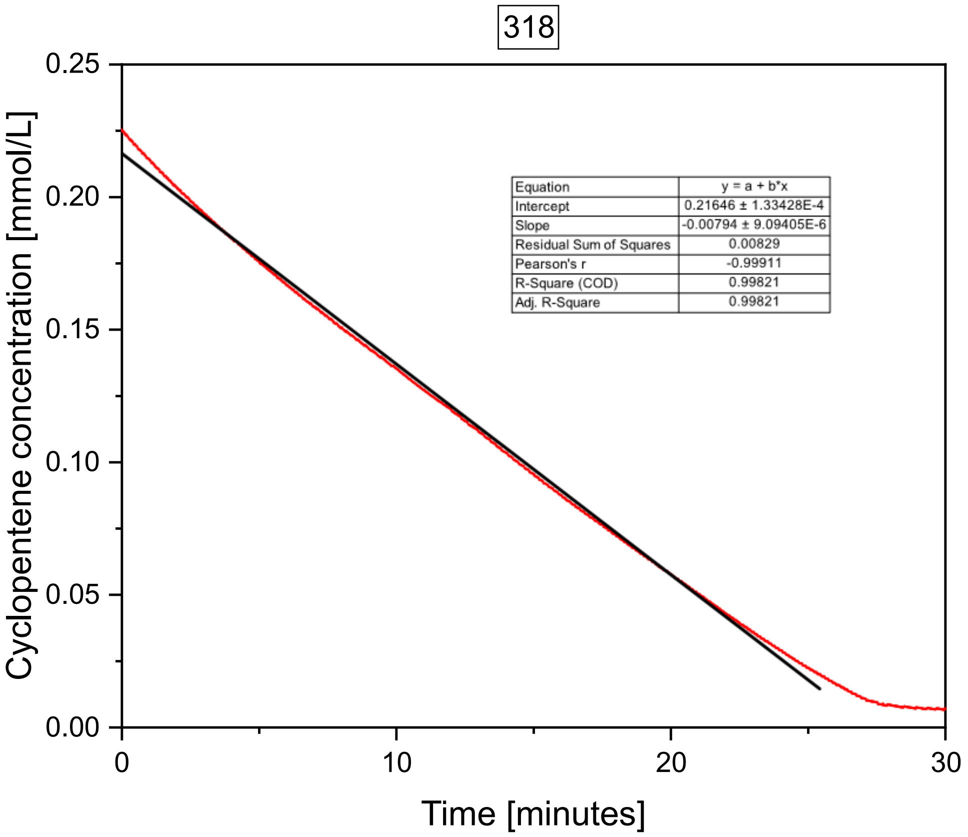

Figure S71. Kinetic data for deuteration of cyclopentene by **2** at 318 K.

Table S7. Table of constants derived from kinetic experiments for the deuteration of cyclopentene by **2**.

Temperature [K]	***1/T [1/K]***	***k^H_{Obs} [mmol*min/L]***	***$ln(k^H_{Obs}/T)$ [mol*s/L*K]***
295.15	0.00339	$4.29*10^{-3}$	-11.139
303.15	0.00330	$5.36*10^{-3}$	-10.943
308.15	0.00325	$6.41*10^{-3}$	-10.780
313.15	0.00319	$7.26*10^{-3}$	-10.672
318.15	0.00314	$7.94*10^{-3}$	-10.598

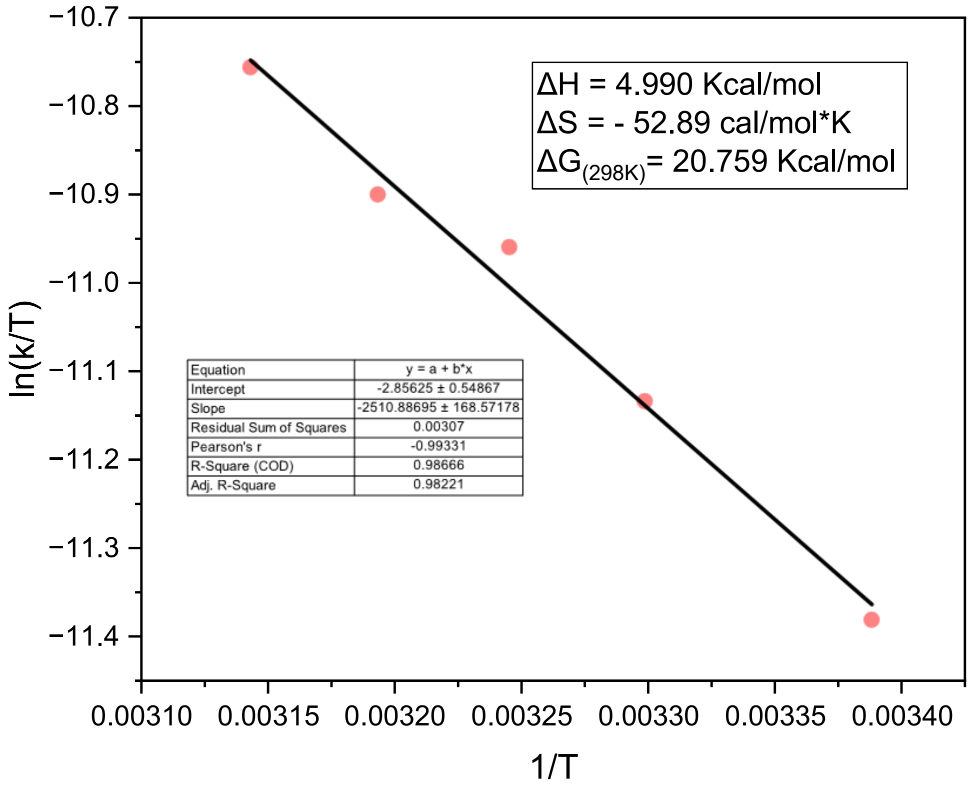

Figure S72. Eyring plot for the hydrogenation of cyclopentene to cyclopentane by **2**.

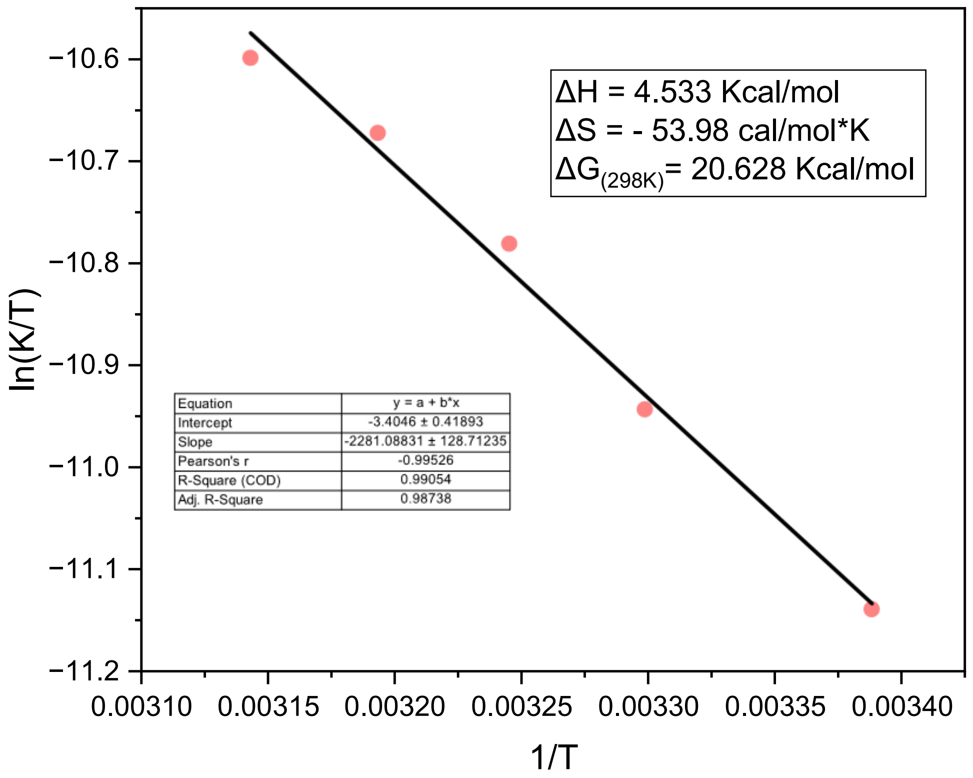

Figure S73. Eyring plot for the deuteration of cyclopentene to cyclopentane by **2**.

Thermodynamic Parameters

To determine the thermodynamic parameters, from the Eyring Plot the following mathematical relations were used: b = slope, a = y-intercept, k_b = Boltzman constant, h = Plank constant, R = ideal gas constant, k = rate constant, $\Delta H^{\ddagger}$ = enthalpy of activation, $\Delta S^{\ddagger}$ = entropy of activation, $\Delta G^{\ddagger}$ = Gibbs energy of activation, T = temperature.

$$k = \frac{k_b * T}{h} * e^{\frac{\Delta S^{\ddagger}}{R}} * e^{-\frac{\Delta H^{\ddagger}}{RT}}$$

$$\Delta H^{\ddagger} = -b * R$$

$$\Delta S^{\ddagger} = (a - \ln\left(\frac{k_b}{h}\right)) * R$$

$$\Delta G^{\ddagger} = \Delta H^{\ddagger} - T\Delta S^{\ddagger}$$

Table S8. Thermodynamic data for dydrogenation/deuteration of cyclopentene to cyclopentane by **2**.

	For H_2	*For D_2*
**$\Delta H^{\ddagger}$ [Kcal/Mol]**	4.990	4.533
**$\Delta S^{\ddagger}$ [cal/Mol*K]**	-52.89	-53.98
**$\Delta G^{\ddagger}_{298}$ [Kcal/Mol]**	20.759	20.628

Calculation of KIE at room temperature

$$KIE = \frac{k^H}{k^D} = e^{\frac{(\Delta G^{D\ddagger} - \Delta G^{H\ddagger})}{RT}} = e^{\frac{(-131\,[\frac{cal}{mol}])}{298.15R}} = \mathbf{0.80}$$

11. Determination of the maximum turnover number

The kinetic kit "X206 Man on the Moon Operator" was used to investigate the maximum turnover number for hydrogenation of cyclopentene, catalyzed by **2**. The conversion of cyclopentene to cyclopentane (*e.g.* hydrogenation rate) was determined by the consumption of H_2/D_2. For this a GC-MS vial was loaded with 0.7 mL of a freshly prepared stock solution containing **2** (0.024 mg, 0.00002 mmol, 0.029 mM) and cyclopentene (13.63 mg, 0.2 mmol, 290 mM) in toluene. The GC vial was then placed in a high-pressure reactor of the "Man on the Moon" company. The reactor was connected to the gas burette (*i.e.* gas reservoir), which was charged at 10 atm of H_2 pressure, and the reaction was set to a constant pressure of 4 atm. The reaction was run at room temperature. The s^{-1} pressure decrease in the gas reservoir was monitored, until no further gas uptake was observed. This led to turnover numbers of ~10,000 in 50 hours of reaction time (Fig. S64), equating to a turn over frequency (TOF) of 200 h^{-1}.

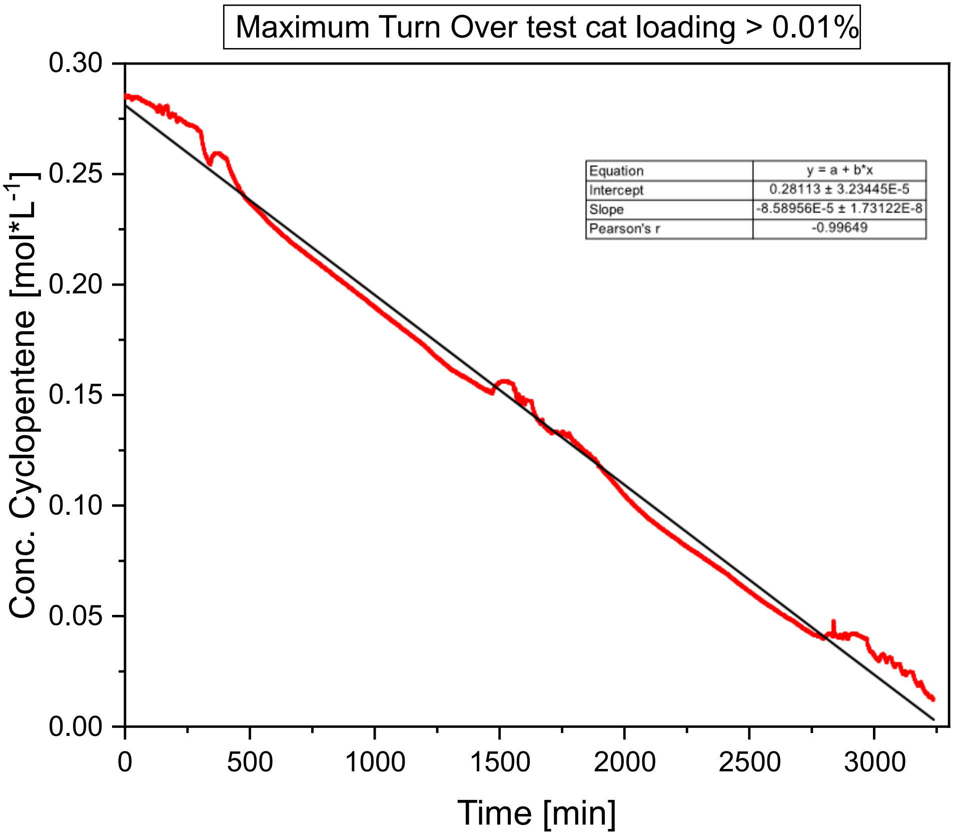

Figure S74. Concentration vs time plot of the hydrogenation of cyclopentene by 0.01mol% of **2** at 298 K.

12. Catalytic hydrogenation of alkenes and alkynes

General Method for Catalytic Hydrogenation.

Catalytic alkene and alkyne hydrogenation was carried out using a 0.5 mL of a stock solution of the catalyst (**2**, 3 mM) in C_6D_6, and mesitylene (50 mM) as internal standard. These solutions were first transferred into a 1mL GC vial containing a 4 mm magnetic stir bar, and the alkene/alkyne (0.150 mmol) subsequently added using a micro-pipette for liquid alkenes, and alkynes or weighed out in an inert atmosphere glovebox for solid alkenes. Following alkene/alkyne addition, the GC vials were capped and placed in a 1L Parr autoclave reactor fitted with custom Teflon or aluminium multi-well inserts (Fig. S75). Each vial was pierced with a fresh needle, the reactor sealed, and subsequently pressurized with dihydrogen (grade 5.0, 5 bar). The resulting solutions were stirred at 500 rpm at the given temperature (*i.e.* between RT and 100 °C) for the given time (*i.e.* between 5 and 48h). The reaction mixtures were filterer into an NMR tubes under ambient conditions, and the reaction progress was determined *via* ^{1}H NMR spectroscopic analysis. The progress was evidenced by integration of the starting material/product compared to the internal standard.

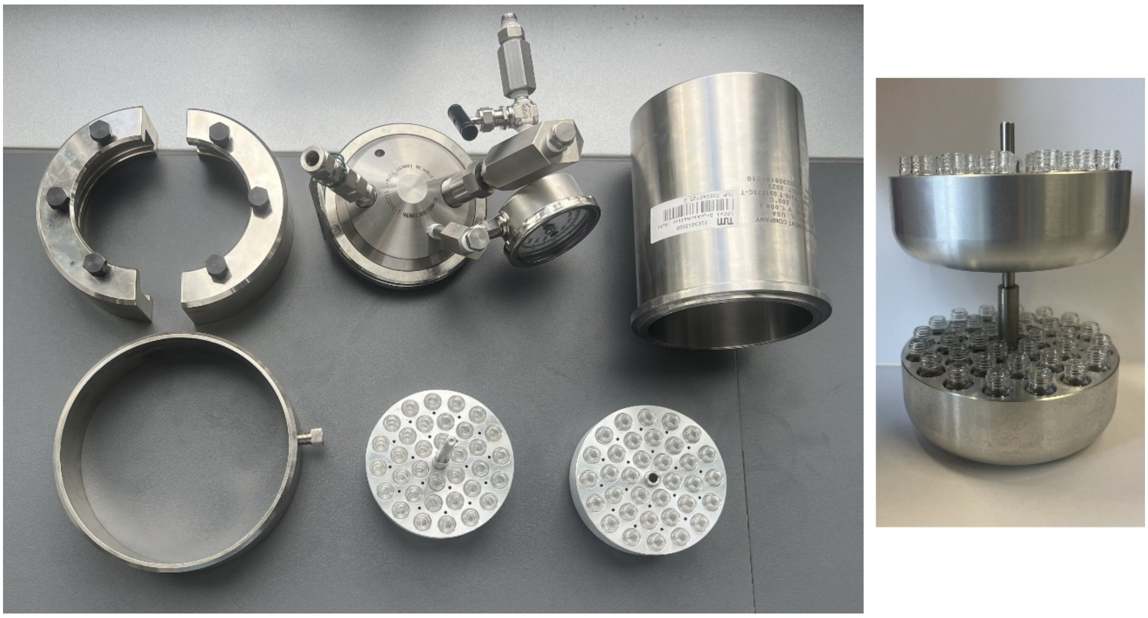

Figure S75. The 1L Parr autoclave utilised for catalytic screening. Custom multi-well aluminium inserts shown on the bottom middle, filled with GC vials; 'stacked' pucks filled with vials are shown on the right, which sits directly inside the reactor.

13. Substrate Scope of alkene/alkyne hydrogenation catalysis

Table S9. Substrate scope for the hydrogenation of alkenes/ alkynes, following the general procedure given above with 1 mol% of **2** under the given time and temperature under 5atm H_2 pressure, unless noted otherwise.

Entry	*T [°C]*	*Time*	*Substrate*	*[%]*	*Product*	*[%]*
1	RT	5 h	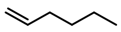	<5	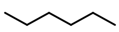	>95
2	RT	5 h	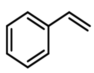	45	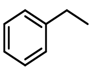	55
3	60	20 h		<5		>95
4	RT	5 h	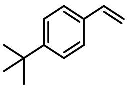	61	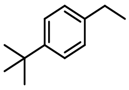	39
5	60	20 h		<5		>95
6	60	2.5 h	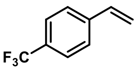	41	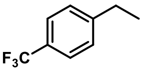	59
7	60	20 h		<5		>95
8	60	2.5 h	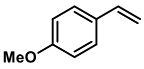	<5	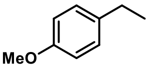	>95
9	60	20 h		<5		>95
10	60	2.5 h	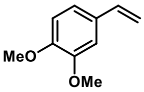	<5	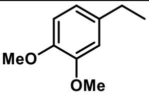	>95
11	60	20 h		<5		>95
12	60	20 h	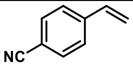	>95	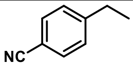	<5
14	60	20 h	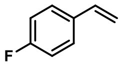	12	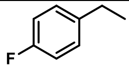	78
15	60	2.5 h	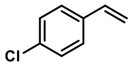	<5	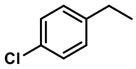	>95
16	60	20 h		<5		>95
17	60	2.5 h	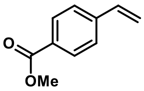	35	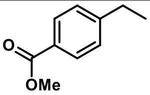	65
18	60	20 h		<5		>95
19	RT	5 h	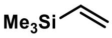	<5	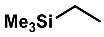	95
20	RT	5 h	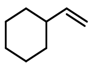	85	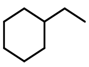	15
21	60	20 h		<5		>95
22	RT	5 h	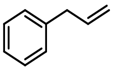	>95	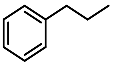	<5
23	60	20 h		<5		68[a]
24	RT	5 h	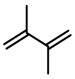	>95	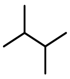	<5
25	60	20 h		<5		65[b]
26	RT	5 h	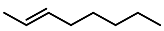	10	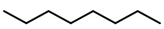	90
27	60	20 h		<5		>95
28	RT	5 h	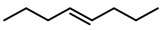	<5	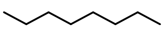	>95
29	60	20 h	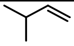	<5	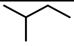	>95
30	RT	5 h	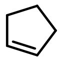	<5	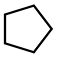	>95
31[c]	RT	50 h		<5		>95
32	RT	5 h	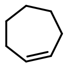	13	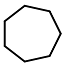	87
33	60	20 h		<5		>95

Entry	T [°C]	Time	Substrate	[%]	Product	[%]
34	RT	5 h	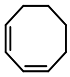	>95	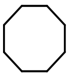	>5
35	60	20 h		<5		>95
36	RT	5 h	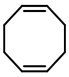	>95	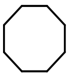	<5
37	60	20		<5		>95
38	RT	5 h	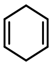	<5	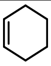	>95
39	60	20 h		<5	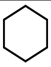	>95
40	RT	5 h	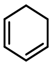	>95	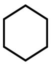	<5
41	60	20 h		<5		>95
42	RT	5 h	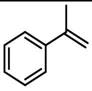	22	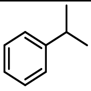	78
43	RT	5 h	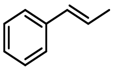	>95	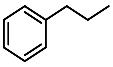	<5
44	60	20 h		<5		>95
45	RT	5 h	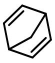	>95	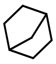	<5
46	60	20 h		<5		<95
47	RT	5 h	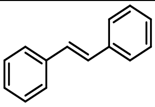	91	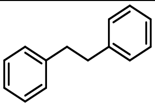	9
48	60	20 h		<5		>95
49	RT	5 h	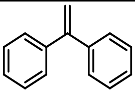	>95	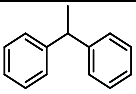	<5
50	60	20 h		>95		<5
51	RT	5 h	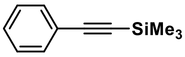	>95	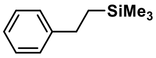	<5
52	60	20 h		<5		>95
53	RT	5 h	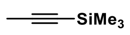	<5	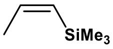	67
54	60	20 h		<5	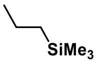	>95
55	RT	5 h	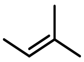	>95	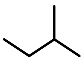	<5
56	60	20 h		<5		>95
57	RT	5 h	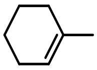	>95	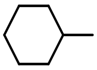	<5
58	60	20 h		55		45
59	RT	5 h	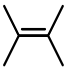	>95	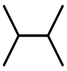	<5
60[d]	60	20 h		85		15
61	100	48 h		60		40
62	RT	5 h	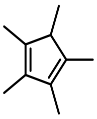	>95	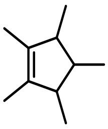	<5
63	60	20 h		>95		<5
64[d]	100	48 h		45		41[e]

[a]32% isomerization to *trans*-β-methylstyrene was observed. [b]35% isomerization to 2,3-dimethyl-but-2-ene was observed. [c]The reaction was carried out with 0.01 mol% of **2** as catalyst. [d]The was carried out with 1.5 mol% of **2** as catalyst at 100°C, 48h with 5 atm H_2 pressure.[e]In addition 14% of full hydrogenation.

14. Catalyst poisoning tests

To ensure that **2** is stable under catalytic conditions (dihydrogen pressure (e.g. 5 bar), elevated temperatures (e.g. 60 °C) and the presence of a substrate (e.g. styrene)), and does not decompose into Ni nanoparticles, which are known to be effective hydrogenation catalysts, poisoning studies were conducted with elemental mercury (Hg). Therefore a 0.5 mL of a stock solution of the catalyst (**2**, 3 mM) in C_6D_6, and mesitylene (50 mM) as internal standard was prepared. This solution was first transferred into a 1mL GC vial containing a 4 mm magnetic stir bar, and styrene (0.150 mmol) subsequently added using a micro-pipette in an inert atmosphere glovebox. Next a couple of drops of elemental Hg were added. Following, the GC vials were capped and placed in a 1L Parr autoclave reactor fitted with custom Teflon or aluminium multi-well inserts (Fig. S75). Each vial was pierced with a fresh needle, the reactor sealed, and subsequently pressurized with dihydrogen (grade 5.0, 5 bar). The resulting solutions were stirred at 500 rpm at 60 °C for 20h. The reaction mixtures were filterer into an NMR tubes under ambient conditions, and the reaction progress was determined *via* ^{1}H NMR spectroscopic analysis. The progress was evidenced by integration of the starting material/product compared to the internal standard. This was done in parallel with two separate samples, for both no difference in the catalytic performance was observed, therefore indicating **2** to be a stable catalyst at elevated temperatures and in fact no formation of Ni nanoparticles.

Styrene

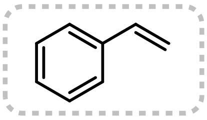

The catalysis was done via the described method, with excess mercury being present. The yield was determined to be >95% of ethyl benzene, after 20 hours at 60°C with 1 mol% of **2** as catalyst. The ^{1}H NMR spectroscopic data matched those found in the literature.[7]

^{1}H NMR (C_6D_6, 400 MHz, 298 K): δ = 7.18 – 7.14 (m, 2H), 7.09-7.05 (m, 3H), 2.44 (q, *J* = 7.6 Hz, 2H), 1.08 (t, *J* = 7.6 Hz, 3H).

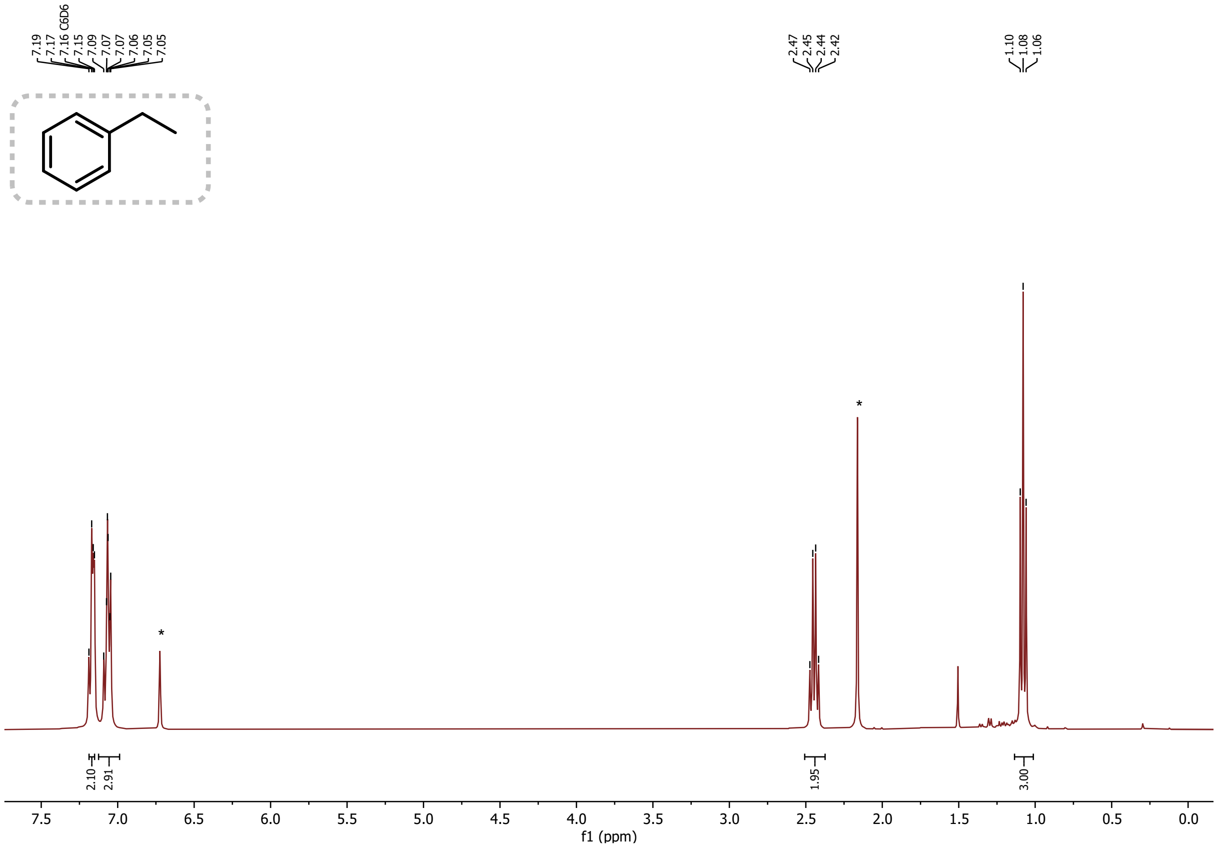

Figure S76. ^{1}H NMR spectrum (C_6D_6, 400 MHz, 298 K) of styrene, after 20 h at 60°C with 1 mol% of **2** as catalyst and excess mercury forming ethylbenzene. *denotes the internal standard mesitylene.

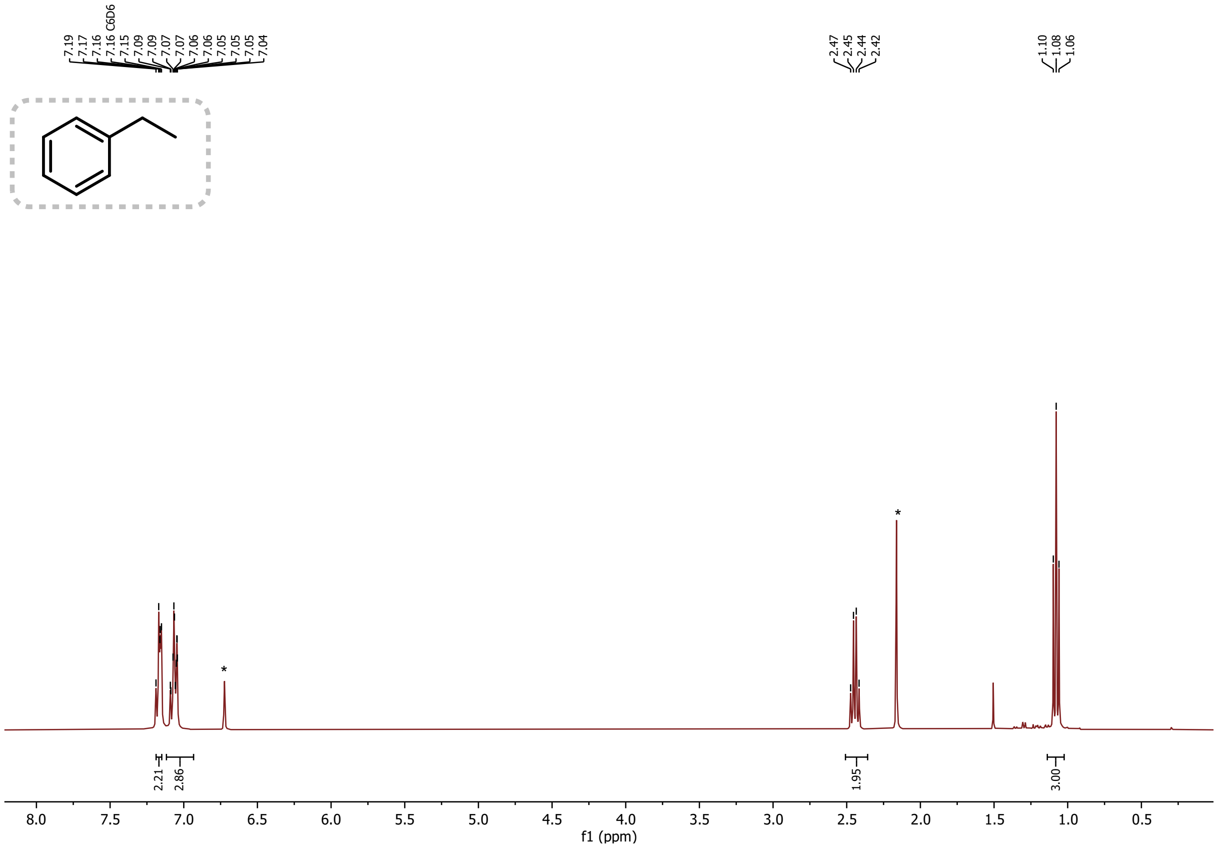

Figure S77. ^{1}H NMR spectrum (C_6D_6, 400 MHz, 298 K) of styrene, after 20 h at 60°C with 1 mol% of **2** as catalyst and excess mercury forming ethylbenzene. *denotes the internal standard mesitylene

15. Analytical data for alkene/ alkyne hydrogenation catalysis

The products of hydrogenation were assigned based on comparison of ^{1}H-NMR resonances to literature data for possible hydrogenation and isomerization products, in addition to oligomerization products.

1-hexene

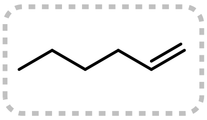

The catalysis was done via the general method. The yield was determined to be >95% of hexane, after five hours at room temperature with 1 mol% of **2** as catalyst. The ^{1}H NMR spectroscopic data matched those found in the literature.[8]

^{1}H NMR (C_6D_6, 400 MHz, 298 K): δ = 1.25 (m, 8H), 0.88 (m, 6H).

styrene

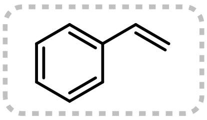

The catalysis was done via the general method. The yield was determined to be 55% of ethyl benzene, after five hours at room temperature with 1 mol% of **2** as catalyst and >95% of ethyl benzene, after 20 hours at 60°C with 1 mol% of **2** as catalyst. The ^{1}H NMR spectroscopic data matched those found in the literature.[7]

^{1}H NMR (C_6D_6, 400 MHz, 298 K): δ = 7.18 – 7.14 (m, 2H), 7.09-7.05 (m, 3H), 2.44 (q, *J* = 7.6 Hz, 2H), 1.08 (t, *J* = 7.6 Hz, 3H).

4-tertbutyl-Styrene

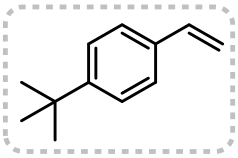

The catalysis was done via the general method. The yield was determined to be 39% of 1-ethyl-4-tertbutyl-benzene, after five hours at room temperature with 1 mol% of **2** as catalyst. The ^{1}H NMR spectroscopic data matched those found in the literature, and >95% of 1-ethyl-4-tertbutyl-benzene, after 20 hours at 60°C with 1 mol% of **2** as catalyst. The ^{1}H NMR spectroscopic data matched those found in the literature.[9]

^{1}H NMR (C_6D_6, 400 MHz, 298 K): δ = 7.28 (d, *J* = 8.3 Hz, 2H), 7.09 (d, *J* = 8.1 Hz, 2H), 2.50 (q, *J* = 7.6 Hz, 2H), 1.26 (s, 9H), 1.14 (t, *J* = 7.6 Hz, 3H).

4-(trifluoromethyl)styrene

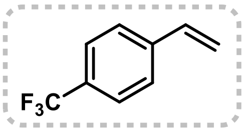

The catalysis was done via the general method. The yield was determined to be 59% of 1-ethyl-4-(trifluoromethyl)benzene, after 2.5 hours at 60°C with 1 mol% of **2** as catalyst and 41% of 4-(trifluoromethyl)styrene, and >95% of 1-ethyl-4-(trifluoromethyl)benzene, after 20 hours at 60°C with 1 mol% of **2** as catalyst. The ^{1}H NMR spectroscopic data matched those found in the literature.[10]

^{1}H NMR (C_6D_6, 400 MHz, 298 K): δ = 7.33 (d, *J* = 8.0 Hz, 2H), 6.79 (d, *J* = 7.9 Hz, 2H), 2.25 (q, *J* = 7.6 Hz, 2H), 0.92 (t, *J* = 7.6 Hz, 3H).

4-(methoxy)styrene

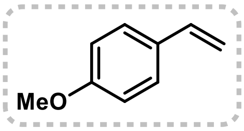

The catalysis was done via the general method. The yield was determined to be >95% of 1-ethyl-4-(methoxy)benzene, after 2.5 hours at 60°C with 1 mol% of **2** as catalyst, and >95% of 1-ethyl-4-(methoxy)benzene, after 20 hours at 60°C with 1 mol% of **2** as catalyst. The ^{1}H NMR spectroscopic data matched those found in the literature.[10]

^{1}H NMR (C_6D_6, 400 MHz, 298 K): δ = 6.99 (d, *J* = 8.6 Hz, 2H), 6.80 (d, *J* = 8.6 Hz, 2H), 3.35 (s, 3H), 2.46 (q, *J* = 7.6 Hz, 2H), 1.11 (t, *J* = 7.6 Hz, 3H).

3,4-(dimethoxy)styrene

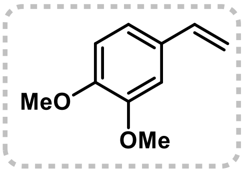

The catalysis was done via the general method. The yield was determined to be >95% of 1-ethyl-3,4-(dimethoxy)benzene, after 2.5 hours at 60°C with 1 mol% of **2** as catalyst and, >95% of 1-ethyl-3,4-(dimethoxy)benzene, after 20 hours at 60°C with 1 mol% of **2** as catalyst. The ^{1}H NMR spectroscopic data matched those found in the literature.[11]

^{1}H NMR (C_6D_6, 400 MHz, 298 K): δ = 6.72 – 6.67 (m, 1H), 6.66 – 6.60 (m, 2H), 3.45 (s, 6H), 2.50 (q, *J* = 7.6 Hz, 2H), 1.16 (t, *J* = 7.6 Hz, 3H).

4-(cyano)styrene

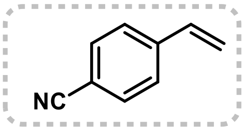

The catalysis was done via the general method. The yield was determined to be <5% of 1-ethyl-4-(cyano)benzene and >95% of 4-cyanostyrene, after 20 hours at 60°C with 1 mol% of **2** as catalyst.

4-(fluoro)styrene

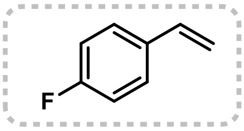

The catalysis was done via the general method. The yield was determined to be 78% of 1-ethyl-4-(fluoro)benzene and 22% of 4-(fluoro)styrene, after 20 hours at 60°C with 1 mol% of **2** as catalyst. The ^{1}H NMR spectroscopic data matched those found in the literature.[10]

^{1}H NMR (C_6D_6, 400 MHz, 298 K): δ = 6.84 – 6.73 (m, 4H), 2.29 (q, *J* = 7.6 Hz, 2H), 0.98 (t, *J* = 7.6 Hz, 3H).

4-(chloro)styrene

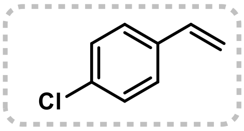

The catalysis was done via the general method. The yield was determined to be >95% of 1-ethyl-4-(chloro)benzene, after 2.5 hours at 60°C with 1 mol% of **2** as catalyst, and >95% of 1-ethyl-4-(chloro)benzene, after 20 hours at 60°C with 1 mol% of **2** as catalyst. The ^{1}H NMR spectroscopic data matched those found in the literature.[12]

^{1}H NMR (C_6D_6, 400 MHz, 298 K): δ = 7.10 (d, *J* = 8.4 Hz, 2H), 6.70 (d, *J* = 8.4 Hz, 2H), 2.24 (q, *J* = 7.6 Hz, 2H), 0.94 (t, *J* = 7.6 Hz, 3H).

4-(acetoxy)styrene

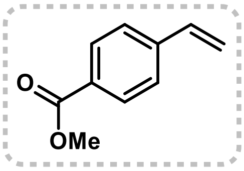

The catalysis was done via the general method. The yield was determined to be 65% of 1-(acetoxy)-4-ethylbenzene, after 2.5 hours at 60°C with 1 mol% of **2** as catalyst, and >95% of 1-(acetoxy)-4-ethylbenzene, after 20 hours at 60°C with 1 mol% of **2** as catalyst. The ^{1}H NMR spectroscopic data matched those found in the literature.[9]

^{1}H NMR (C_6D_6, 400 MHz, 298 K): δ = 7.01 (d, *J* = 8.5 Hz, 2H), 6.93 (d, *J* = 8.5 Hz, 2H), 2.36 (q, *J* = 7.6 Hz, 2H), 1.77 (s, 3H), 1.02 (t, *J* = 7.6 Hz, 3H).

vinyl-trimethylsilane

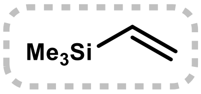

The catalysis was done via the general method. The yield was determined to be >95% of ethyl-TMS, after five hours at room temperature with 1 mol% of **2** as catalyst. The ^{1}H NMR spectroscopic data matched those found in the literature.[13]

^{1}H NMR (C_6D_6, 400 MHz, 298 K): δ = 0.93 (t, *J* = 7.9 Hz, 3H), 0.45 (q, *J* = 8.3 Hz, 2H), -0.02 (s, 9H).

allyl-cyclohexane

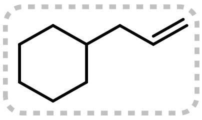

The catalysis was done via the general method. The yield was determined to be 15% of propyl-cyclohexane, after 5 hours at room temperature with 1 mol% of **2** as catalyst, and >95% of propyl-cyclohexane, after 20 hours at 60°C with 1 mol% of **2** as catalyst. The ^{1}H NMR spectroscopic data matched those found in the literature.[14]

^{1}H NMR (C_6D_6, 400 MHz, 298 K): δ = 1.68 (m, 5H), 1.33 – 1.26 (m, 2H), 1.16 (m, 6H), 0.96 – 0.75 (m, 5H).

allyl-benzene

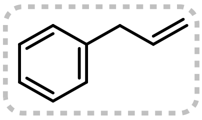

The catalysis was done via the general method. The yield was determined to be >5% of propyl-benzene, after 5 hours at room temperature with 1 mol% of **2** as catalyst, and 68% of propyl-benzene, after 20 hours at 60°C with 1 mol% of **2** as catalyst. 32% of isomerization to beta-methyl-styrene were observed. The ^{1}H NMR spectroscopic data matched those found in the literature.[14]

^{1}H NMR (C_6D_6, 400 MHz, 298 K): δ = 7.24 (d, *J* = 7.7 Hz, 1H), 7.17 (d, *J* = 5.6 Hz, 3H), 7.05 (d, *J* = 7.4 Hz, 2H), 2.42 (t, *J* = 7.6 Hz, 2H), 1.51 (q, *J* = 7.4 Hz, 2H), 0.83 (t, *J* = 7.4 Hz, 3H).

2,3-dimethyl-1,4-butadiene

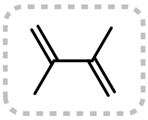

The catalysis was done via the general method. The yield was determined to be >5% of 2,3-dimethyl-butane, after 5 hours at room temperature with 1 mol% of **2** as catalyst, and 65% of 2,3-dimethyl-butane, after 20 hours at 60°C with 1 mol% of **2** as catalyst. 35% of isomerization to 2,3-dimethyl-but-2-ene were observed. The ^{1}H NMR spectroscopic data matched those found in the literature.[15]

^{1}H NMR (C_6D_6, 400 MHz, 298 K): δ =1.38 - 1.32 (m, 2H), 0.86 – 0.80 (m, 12H).

3,3-dimethyl-but-1-ene

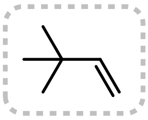

The catalysis was done via the general method. The yield was determined to be >95% of 3,3-dimethyl-butane, after 20 hours at 60°C with 1 mol% of **2** as catalyst. The ^{1}H NMR spectroscopic data matched those found in the literature.[15]

^{1}H NMR (C_6D_6, 400 MHz, 298 K): δ =1.16 (m, 2H), 0.84 (s, 9H), 0.80 (t, *J* = 7.5 Hz, 3H).

1,3-cyclooctadiene

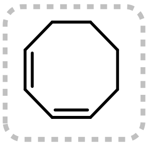

The catalysis was done via the general method. The yield was determined to be >5% of cyclooctane, after 5 hours at room temperature with 1 mol% of **2** as catalyst, and >95% of cyclooctane, after 20 hours at 60°C with 1 mol% of **2** as catalyst. The ^{1}H NMR spectroscopic data matched those found in the literature.[16]

^{1}H NMR (C_6D_6, 400 MHz, 298 K): δ =1.51 (s, 16H).

***trans*-2-octene**

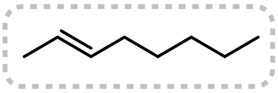

The catalysis was done via the general method. The yield was determined to be 90% of octane, after 5 hours at room temperature with 1 mol% of **2** as catalyst, and >95% of octane, after 20 hours at 60°C with 1 mol% of **2** as catalyst. The ^{1}H NMR spectroscopic data matched those found in the literature.[17]

^{1}H NMR (C_6D_6, 400 MHz, 298 K): δ =1.26 (s, 12H), 0.91 (t, J = 6.7 Hz, 6H).

***trans*-4-octene**

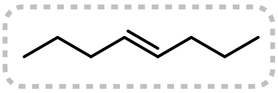

The catalysis was done via the general method. The yield was determined to be >95% of octane, after 5 hours at room temperature with 1 mol% of **2** as catalyst. The ^{1}H NMR spectroscopic data matched those found in the literature.[17]

^{1}H NMR (C_6D_6, 400 MHz, 298 K): δ =1.26 (s, 12H), 0.91 (t, J = 6.7 Hz, 6H).

***trans*-stilbene**

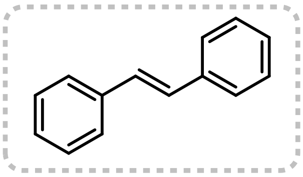

The catalysis was done via the general method. The yield was determined to be 9% of diphenyl ethane, after 5 hours at room temperature with 1 mol% of **2** as catalyst, and >95% of diphenyl ethane, after 20 hours at 60°C with 1 mol% of **2** as catalyst.[16]

^{1}H NMR (C_6D_6, 400 MHz, 298 K): δ = 7.18 – 7.12 (m, 4H), 7.07 (d, *J* = 6.6 Hz, 2H), 7.03 – 6.96 (m, 4H), 2.74 (s, 4H).

cyclopentene

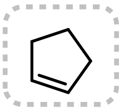

The catalysis was done via the general method. The yield was determined to be >95% of cyclopentane, after 5 hours at room temperature with 1 mol% of **2** as catalyst. The ^{1}H NMR spectroscopic data matched those found in the literature.[18]

^{1}H NMR (C_6D_6, 400 MHz, 298 K): δ = 1.46 (m, 10H)

1,4-cyclohexadiene

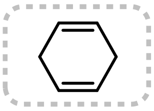

The catalysis was done via the general method. The yield was determined to be >95% of cyclohexene, after 5 hours at room temperature with 1 mol% of **2** as catalyst. The ^{1}H NMR spectroscopic data matched those found in the literature.[19]

^{1}H NMR (C_6D_6, 400 MHz, 298 K): δ = 5.70 (t, *J* = 1.8 Hz, 2H), 1.91 (mz, 4H), 1.57 – 1.47 (m, 4H).

After 20 hours at 60°C with 1 mol% of **2** as catalyst, full reduction to cyclohexane was seen; the yield was determined to be >95%. The ^{1}H NMR spectroscopic data matched those found in the literature.[7]

^{1}H NMR (C_6D_6, 400 MHz, 298 K): δ = 1.40 (s, 12H).

1,3-cyclohexadiene

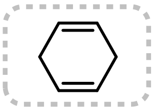

The catalysis was done via the general method. The yield was determined to be >95% of cyclohexane, after 20 hours at 60°C with 1 mol% of **2** as catalyst. The ^{1}H NMR spectroscopic data matched those found in the literature.[7]

^{1}H NMR (C_6D_6, 400 MHz, 298 K): δ = 1.41 (s, 12H).

Cycloheptene

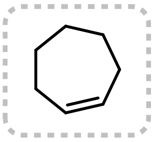

The catalysis was done via the general method. The yield was determined to be 87% of cycloheptane, after 5 hours at room temperature with 1 mol% of **2** as catalyst, and >95% of cycloheptane, after 20 hours at 60°C with 1 mol% of **2** as catalyst. The ^{1}H NMR spectroscopic data matched those found in the literature.[20]

^{1}H NMR (C_6D_6, 400 MHz, 298 K): δ = 1.50 (s, 14H).

1,5-cyclooctadiene

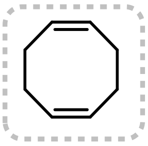

The catalysis was done via the general method. The yield was determined to be >95% of cyclooctane, after 20 hours at 60°C with 1 mol% of **2** as catalyst. The ^{1}H NMR spectroscopic data matched those found in the literature.[16]

^{1}H NMR (C_6D_6, 400 MHz, 298 K): δ =1.51 (s, 16H).

2,5-norbornadiene

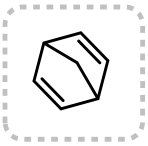

The catalysis was done via the general method. The yield was determined to be >95% of norborane, after 20 hours at 60°C with 1 mol% of **2** as catalyst. The ^{1}H NMR spectroscopic data matched those found in the literature.[9]

^{1}H NMR (C_6D_6, 400 MHz, 298 K): δ = 2.17 - 2.14 (m, 2H), 1.46 - 1.41 (m, 4H), 1.15 - 1.10 (m, 6H). (multiplet at 2.14 overlayered by internal standard mesitylene).

α-methyl-styrene

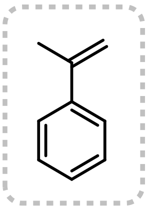

The catalysis was done via the general method. The yield was determined to be 78% of isopropyl benzene, after 5 hours at RT with 1 mol% of **2** as catalyst. The ^{1}H NMR spectroscopic data matched those found in the literature.[7]

^{1}H NMR (C_6D_6, 400 MHz, 298 K): δ = δ 7.18 (s, 2H), 7.11 – 7.05 (m, 3H), 2.70 (p, J = 6.9 Hz, 1H), 1.13 (d, J = 6.9 Hz, 6H).

***trans*-β-methylstyrene**

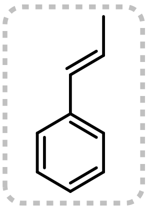

The catalysis was done via the general method. The yield was determined to be >95% of propyl benzene, after 20 hours at 60°C with 1 mol% of **2** as catalyst. The ^{1}H NMR spectroscopic data matched those found in the literature.[14]

^{1}H NMR (C_6D_6, 400 MHz, 298 K): δ = 7.16 (m, 2H), 7.05 (m, 3H), 2.41 (t, J = 7.6 Hz, 2H), 1.50 (q, J = 7.4 Hz, 2H), 0.82 (t, J = 7.4 Hz, 3H).

1-trimethylsilyl-2-propyne

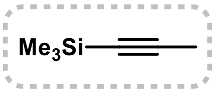

The catalysis was done via the general method. The yield was determined to be 67% of *Z*-1-trimethylsilyl-prop-2-ene, after 5 hours at room temperature with 1 mol% of **2** as catalyst. The ^{1}H NMR spectroscopic data matched those found in the literature.[21]

^{1}H NMR (C_6D_6, 400 MHz, 298 K): δ = 6.01 (m, 1H), 5.70 (dd, *J* = 18.4, 1.4 Hz, 1H), 1.70 (dd, *J* = 6.2, 1.6 Hz, 3H), 0.09 (s, 9H).

After 20 hours at 60°C with 1 mol% of **2** as catalyst, trimethyl(propyl)silane is formed, with a yield of >95%. The ^{1}H NMR spectroscopic data matched those found in the literature.[3]
^{1}H NMR (C_6D_6, 400 MHz, 298 K): δ = 1.34-1.27 (m, 2H), 0.98 (m, 3H), 0.50 – 0.42 (m, 2H), -0.00 (s, 9H).

1-trimethylsilyl-2-phenylacetylene

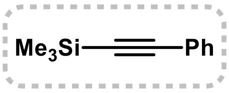

The catalysis was done via the general method. The yield was determined to be >95% of trimethyl(phenethyl)silane, after 20 hours at 60°C with 1 mol% of **2** as catalyst. The ^{1}H NMR spectroscopic data matched those found in the literature. [3]

^{1}H NMR (C_6D_6, 400 MHz, 298 K): δ = 7.38 (d, *J* = 7.8 Hz, 2H), 6.87 (d, *J* = 7.8 Hz, 2H), 2.37 (t, *J* = 8.6 Hz, 2H), 0.62 (m, 2H), -0.05 (s, 9H).

2-methyl-but-2-ene

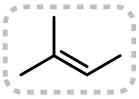

The catalysis was done via the general method. The yield was determined to be >95% of 2-methyl-butane, after 20 hours at 60°C with 1 mol% of **2** as catalyst. The ^{1}H NMR spectroscopic data matched those found in the literature.[22]

^{1}H NMR (C_6D_6, 400 MHz, 298 K): δ = 1.36 (h, *J* = 7.0 Hz, 1H), 1.14 (q, *J* = 7.3 Hz, 2H), 0.85 (d, *J* = 7.7 Hz, 9H).

1-methyl-cyclohex-1-ene

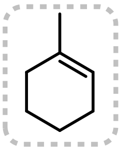

The catalysis was done via the general method. The yield was determined to be 45% of methyl-cyclohexane, after 20 hours at 60°C with 1 mol% of **2** as catalyst. The ^{1}H NMR spectroscopic data matched those found in the literature.[15]

^{1}H NMR (C_6D_6, 400 MHz, 298 K): 1.51-1.29 (m, 6H), 1.15-1.05 (m, 5H), 0.86 (d, *J* = 6.5 Hz, 3H).

2,3-dimethyl-but-2-ene

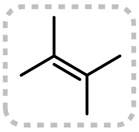

The catalysis was done via the general method. The yield was determined to be 15% of 2,3-dimethylbutane after 20 hours at 60°C with 1 mol% of **2** as catalyst, and 40% of 2,3-dimethylbutane after 48 hours at 100°C with 1.5 mol% of **2** as catalyst. The ^{1}H NMR spectroscopic data matched those found in the literature.[15]

^{1}H NMR (C_6D_6, 400 MHz, 298 K): δ = 1.38 - 1.32 (m, 2H), 0.86 – 0.80 (m, 12H).

Cp*H

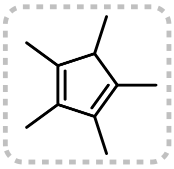

The catalysis was done via the general method. The yield was determined to be 41% of pentamethylcyclopentene and 14% of 1,2,3,4,5-pentamethyl-cyclopentanne after 48 hours at 100°C with 1.5 mol% of **2** as catalyst.

^{1}H NMR of 1,2,3,4,5-pentamethylcyclopent-1-ene (C_6D_6, 400 MHz, 298 K): δ = 2.34 (m, 2H), 1.57 (s, 6H), 0.92 (d, *J* = 6.7 Hz, 6H), 0.89 (d, *J* = 6.4 Hz, 3H). (N.B. one H atom overlayered by Cp*H).

^{1}H NMR of 1,2,3,4,5-pentamethyl-cyclopentane (C_6D_6, 400 MHz, 298 K): δ = 1.52 (s, 5H), 0.99 (d, *J* = 7.7 Hz, 16H).

16. Spectroscopic data for alkene/alkyne hydrogenation catalysis

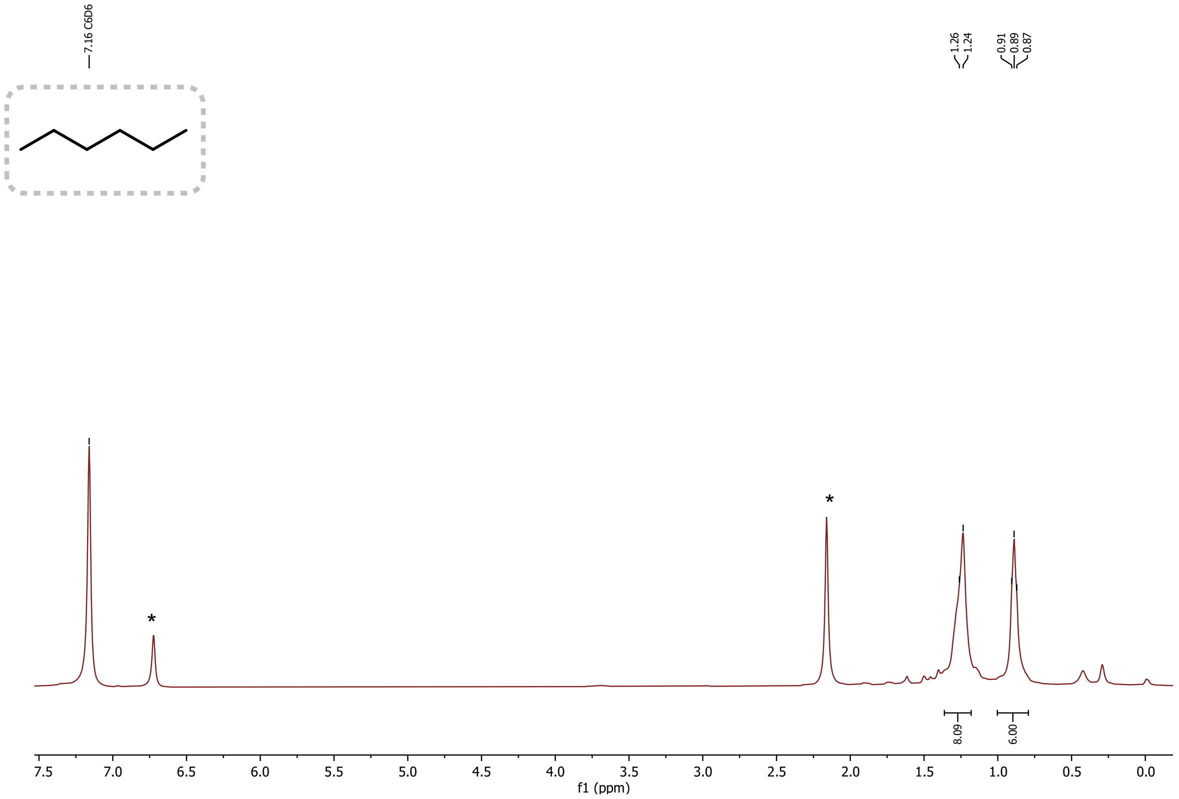

Figure S78. ^{1}H NMR spectrum (C_6D_6, 400 MHz, 298 K) of 1-hexene, after 5 h at room temperature with 1 mol% of **2** as catalyst forming hexane. *denotes the internal standard mesitylene.

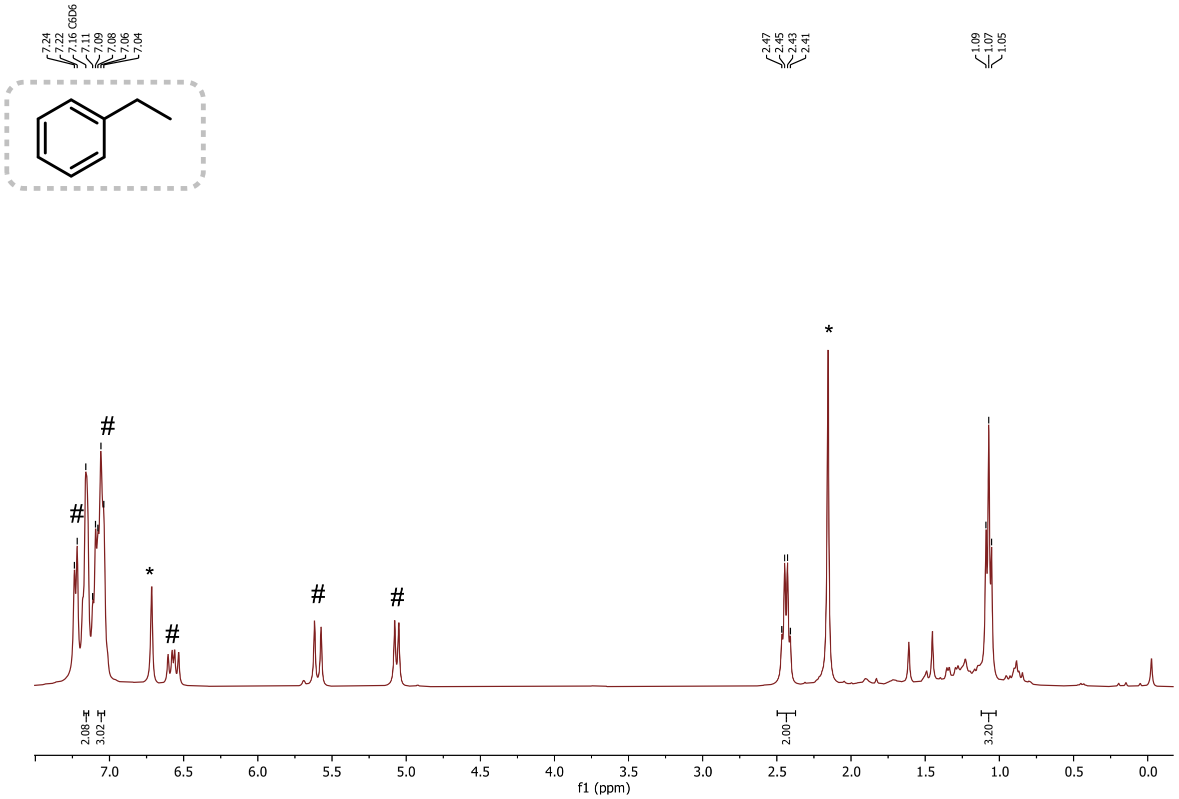

Figure S79. ^{1}H NMR spectrum (C_6D_6, 400 MHz, 298 K) of styrene, after 5 h at RT with 1 mol% of **2** as catalyst forming ethylbenzene. *denotes the internal standard mesitylene. #denotes 45% of styrene.

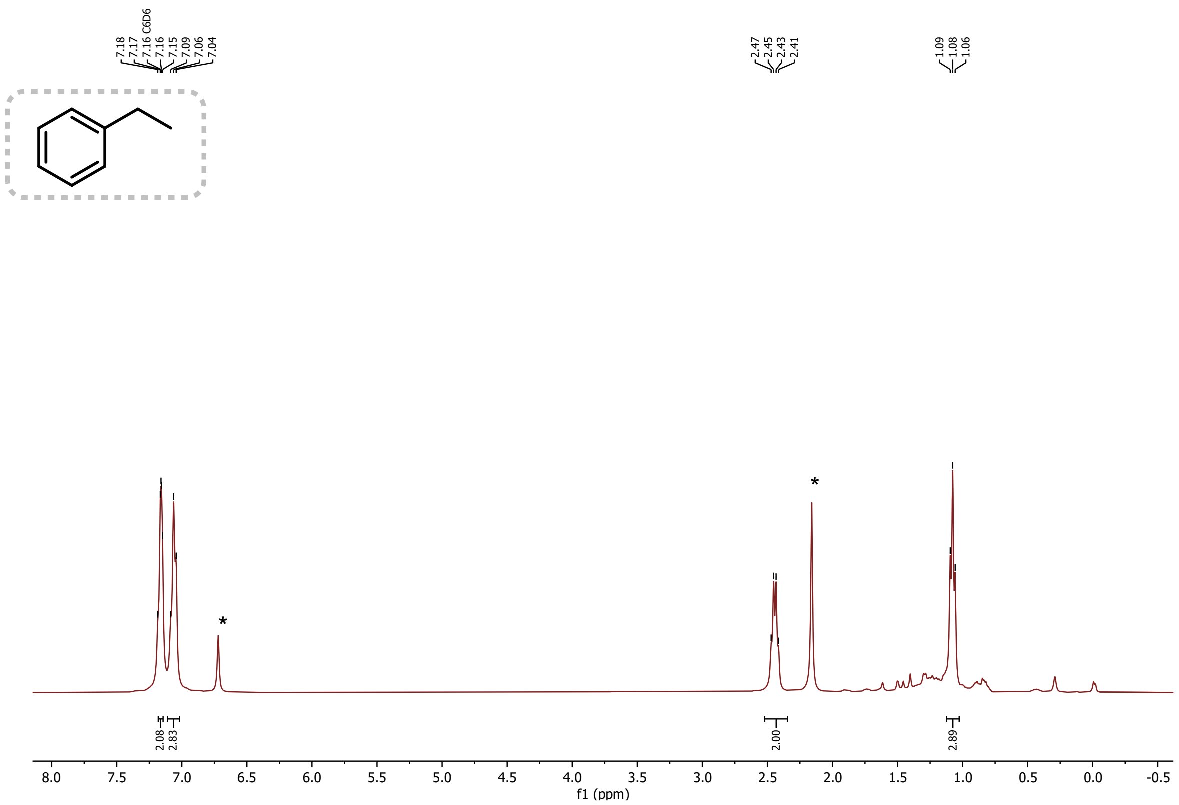

Figure S80. ^{1}H NMR spectrum (C_6D_6, 400 MHz, 298 K) of styrene, after 20 h at 60°C with 1 mol% of **2** as catalyst forming ethylbenzene. *denotes the internal standard mesitylene.

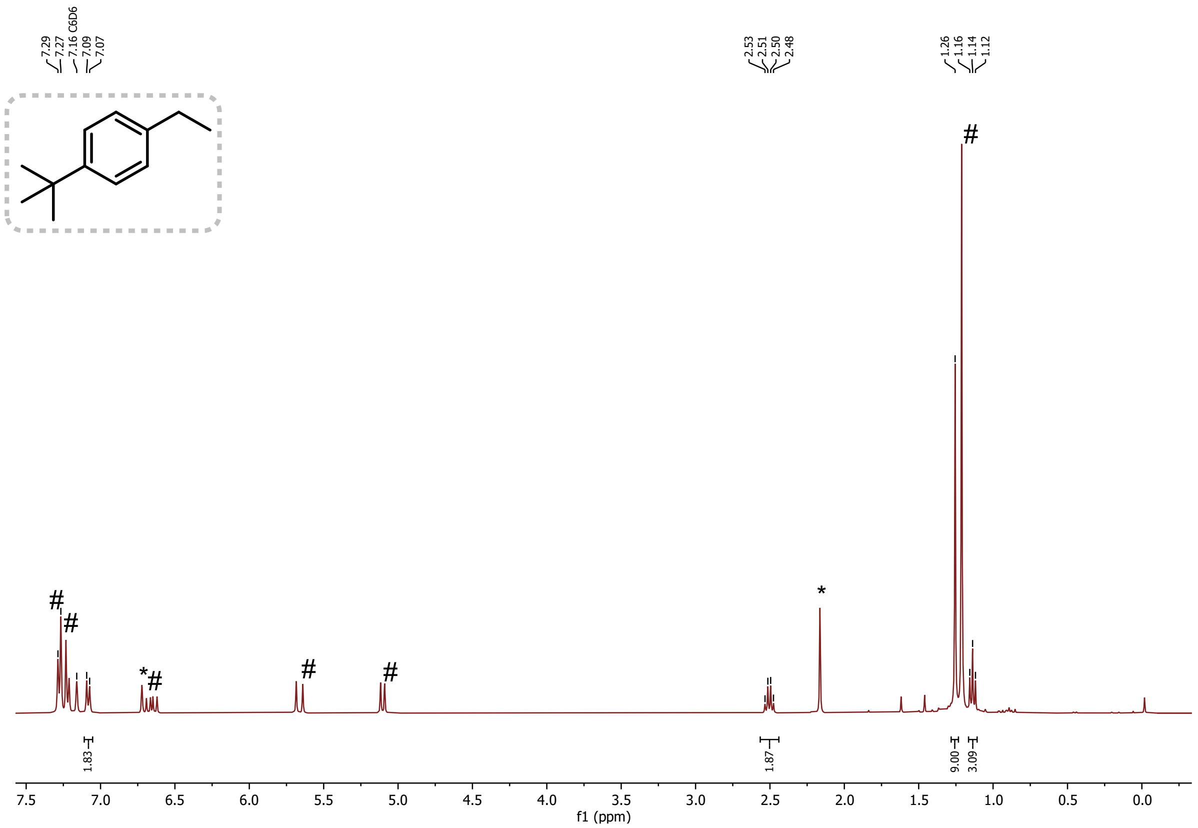

Figure S81. ^{1}H NMR spectrum (C_6D_6, 400 MHz, 298 K) of 4-*tert*-butylstyrene, after 5 h at RT with 1 mol% of **2** as catalyst forming 1-ethyl-4-*tert*-butylbenzene. *denotes the internal standard mesitylene. #denotes 61% of 4-tertbutyl-styrene.

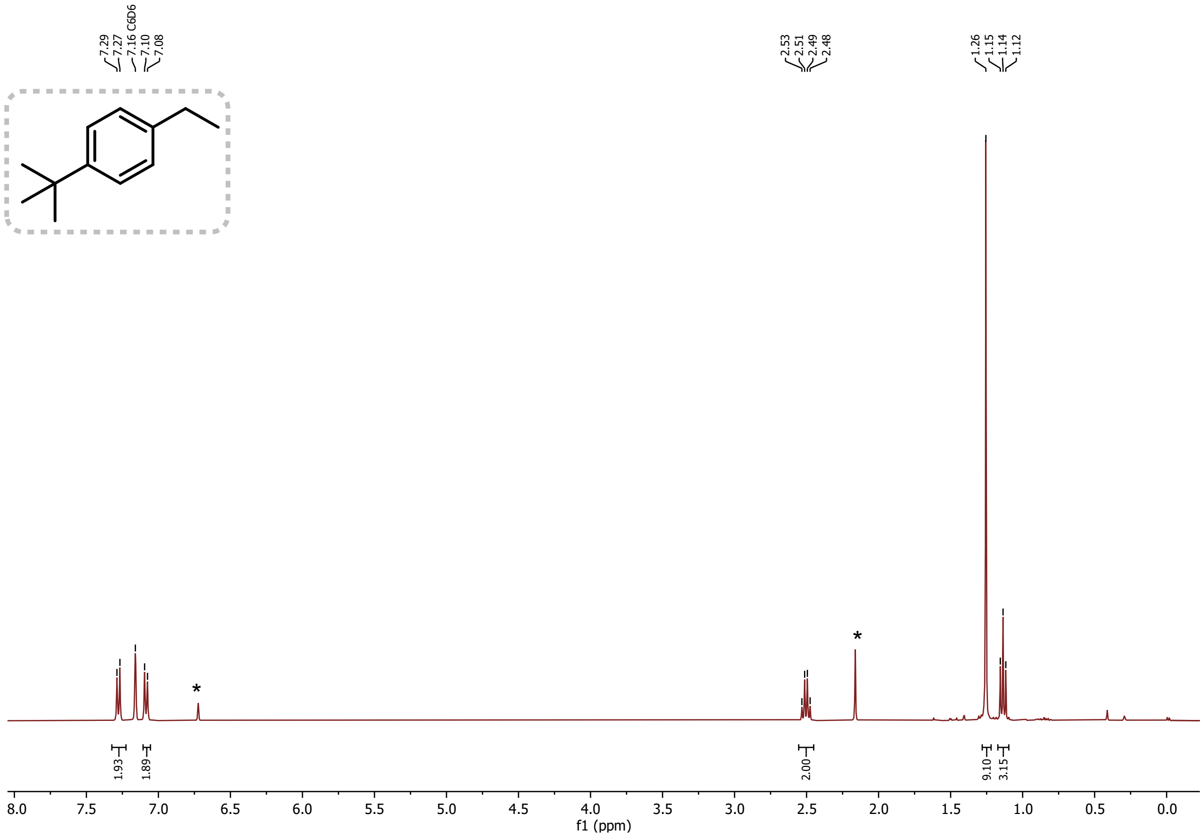

Figure S82. ^{1}H NMR spectrum (C_6D_6, 400 MHz, 298 K) of 4-*tert*-butylstyrene, after 20 h at 60°C with 1 mol% of **2** as catalyst forming 1-ethyl-4-*tert*-butylbenzene. *denotes the internal standard mesitylene.

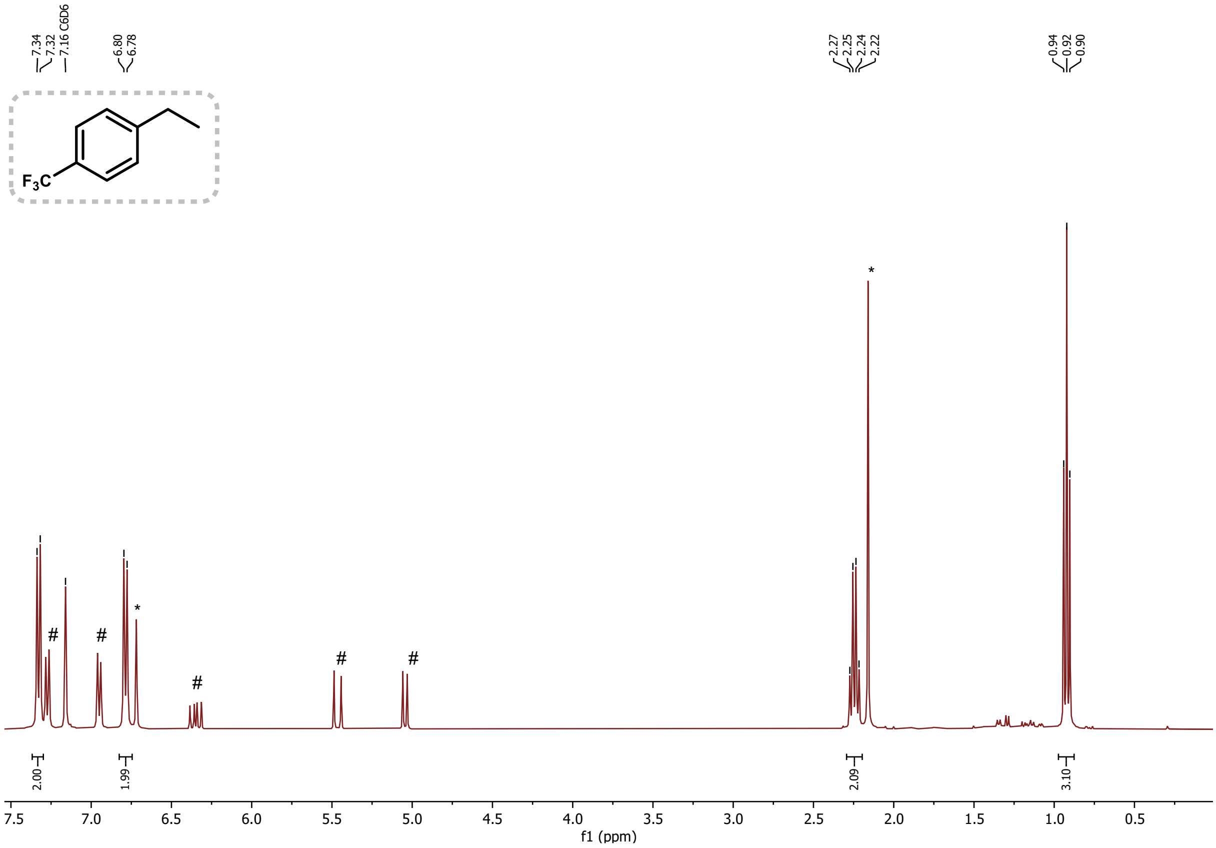

Figure S83. ^{1}H NMR spectrum (C_6D_6, 400 MHz, 298 K) of 4-(trifluoromethyl)-styrene, after 2.5 h at 60°C with 1 mol% of **2** as catalyst forming 59% of 1-ethyl-4-(trifluoromethyl)-benzene. *denotes the internal standard mesitylene. # denotes 4-(trifluoromethyl)styrene.

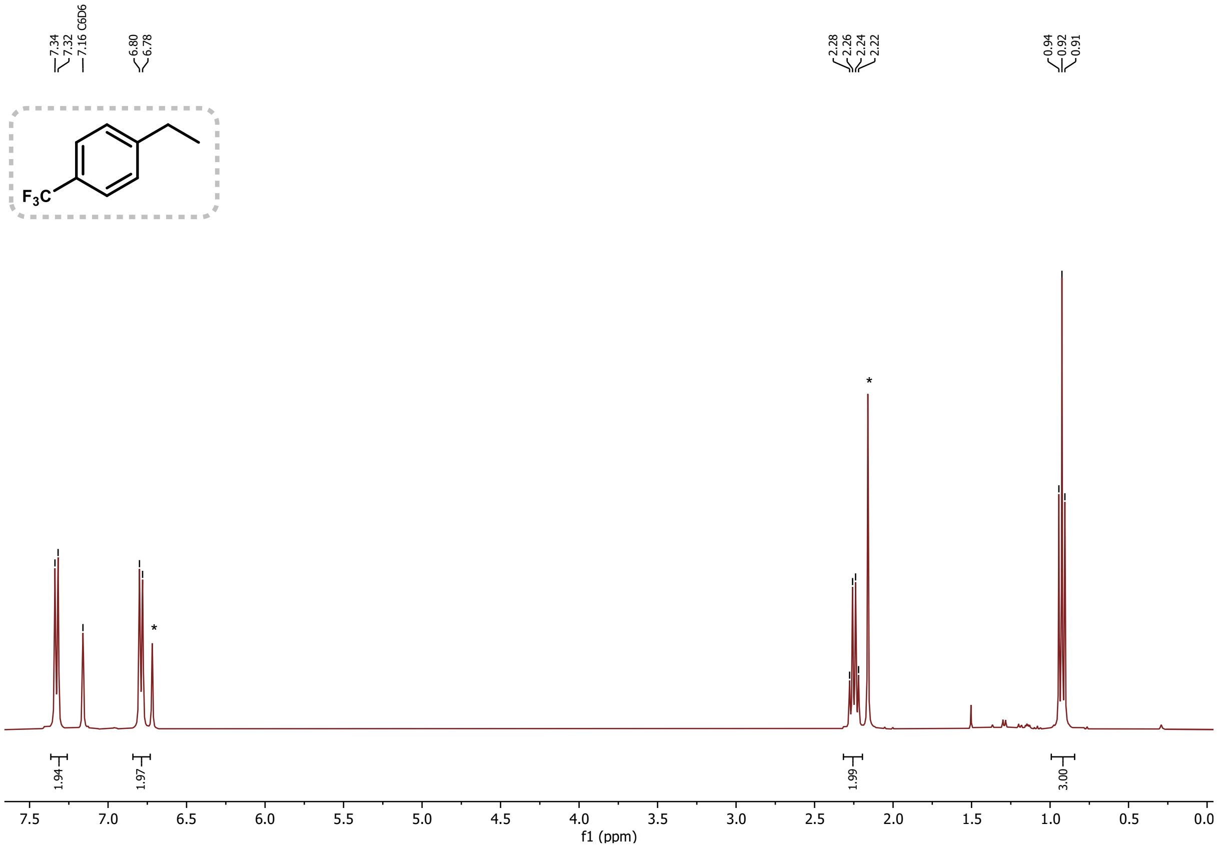

Figure S84. ^{1}H NMR spectrum (C_6D_6, 400 MHz, 298 K) of 4-(trifluoromethyl)-styrene, after 20 h at 60°C with 1 mol% of **2** as catalyst forming 1-ethyl-4-(trifluoromethyl)-benzene. *denotes the internal standard mesitylene.

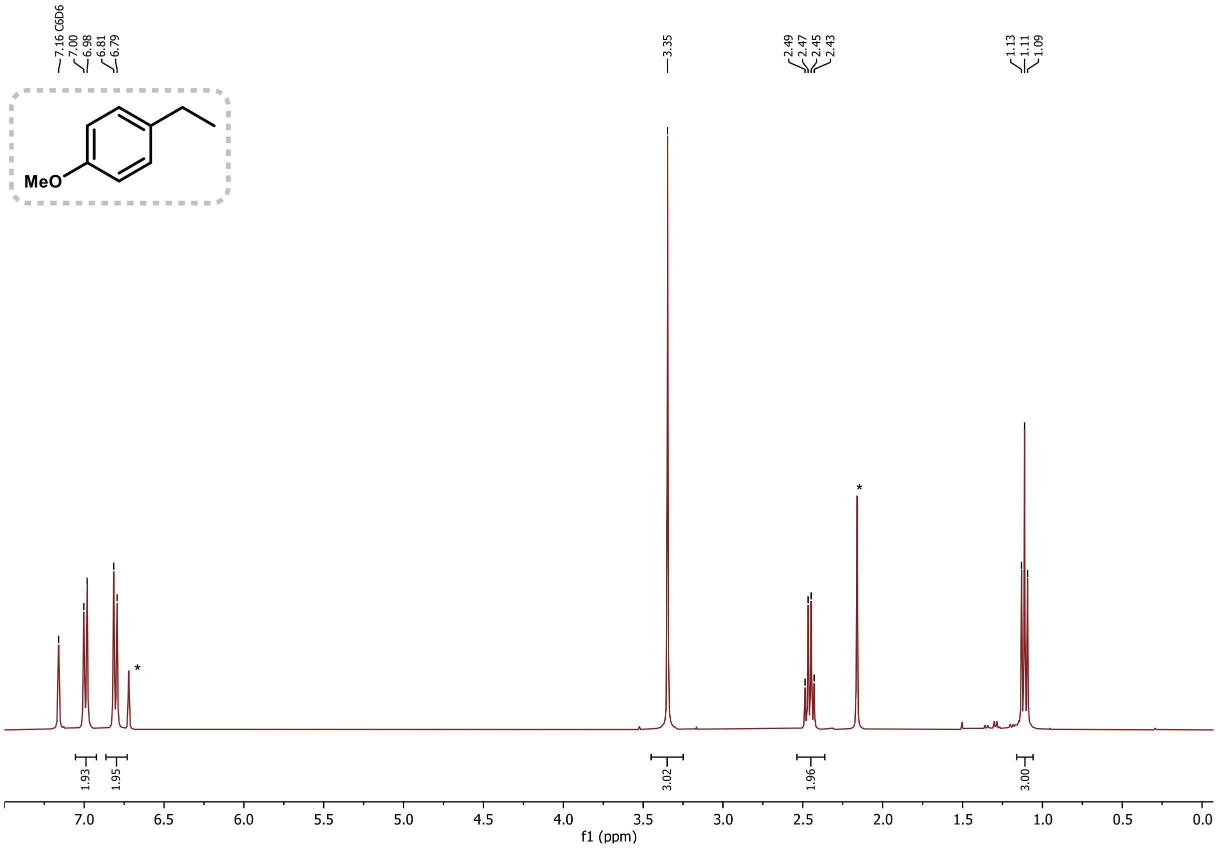

Figure S85. ^{1}H NMR spectrum (C_6D_6, 400 MHz, 298 K) of 4-(methoxy)-styrene, after 2.5 h at 60°C with 1 mol% of **2** as catalyst forming 1-ethyl-4-(methoxy)-benzene. *denotes the internal standard mesitylene.

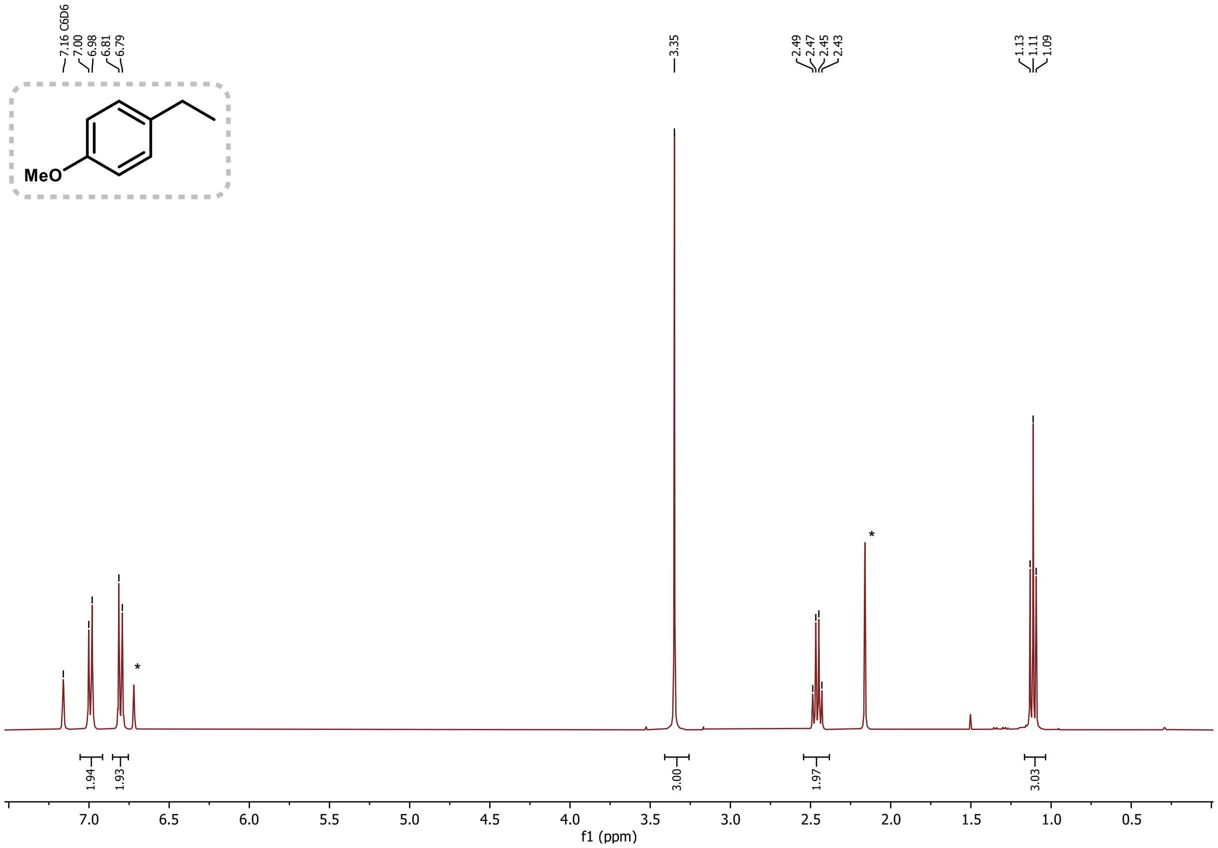

Figure S86. ^{1}H NMR spectrum (C_6D_6, 400 MHz, 298 K) of 4-(methoxy)-styrene, after 20 h at 60°C with 1 mol% of **2** as catalyst forming 1-ethyl-4-(methoxy)-benzene. *denotes the internal standard mesitylene.

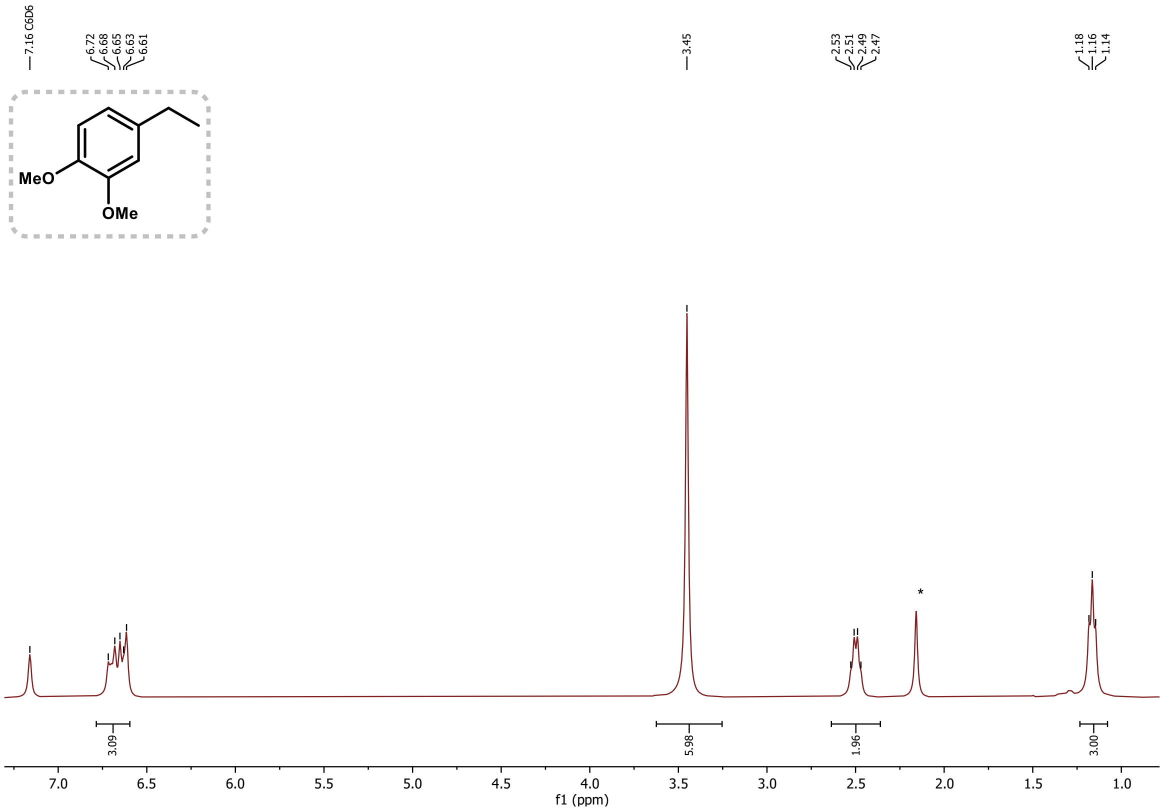

Figure S87. ^{1}H NMR spectrum (C_6D_6, 400 MHz, 298 K) of 3,4-(dimethoxy)-styrene, after 2.5 h at 60°C with 1 mol% of **2** as catalyst forming 1-ethyl-3,4-(dimethoxy)-benzene. *denotes the internal standard mesitylene.

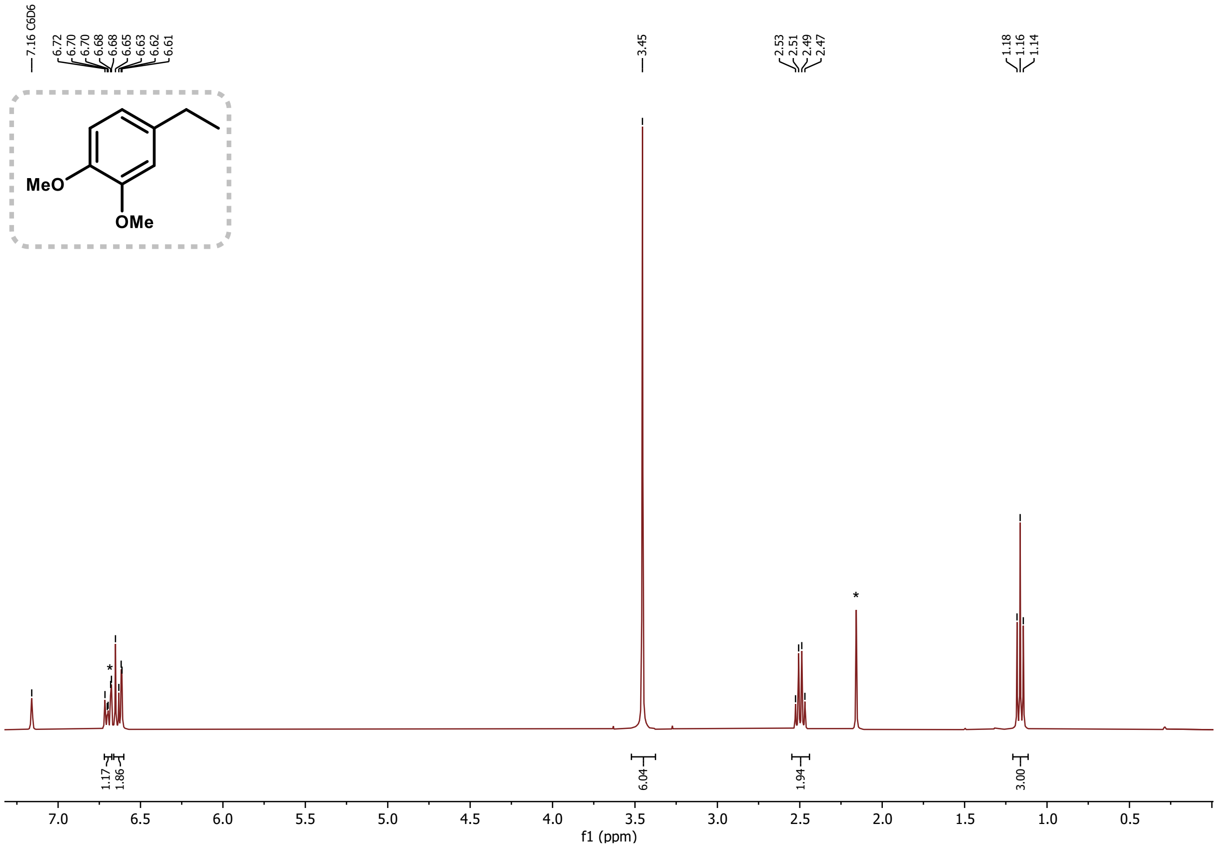

Figure S88. ^{1}H NMR spectrum (C_6D_6, 400 MHz, 298 K) of 3,4-(dimethoxy)-styrene, after 20 h at 60°C with 1 mol% of **2** as catalyst forming 1-ethyl-3,4-(dimethoxy)-benzene. *denotes the internal standard mesitylene.

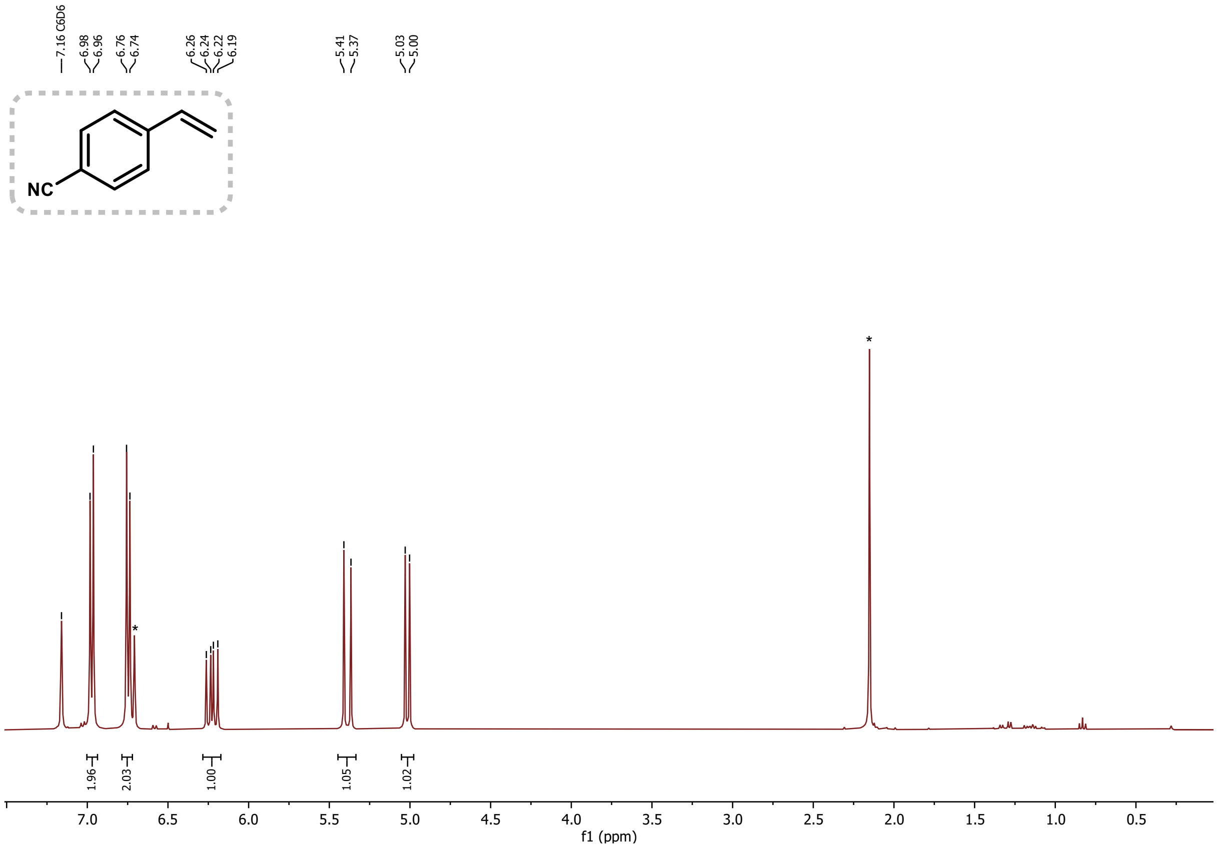

Figure S89. ^{1}H NMR spectrum (C_6D_6, 400 MHz, 298 K) of 4-(cyano)-styrene, after 20 h at 60°C with 1 mol% of **2** as catalyst showing no conversion of 4-(cyano)-styrene. *denotes the internal standard mesitylene.

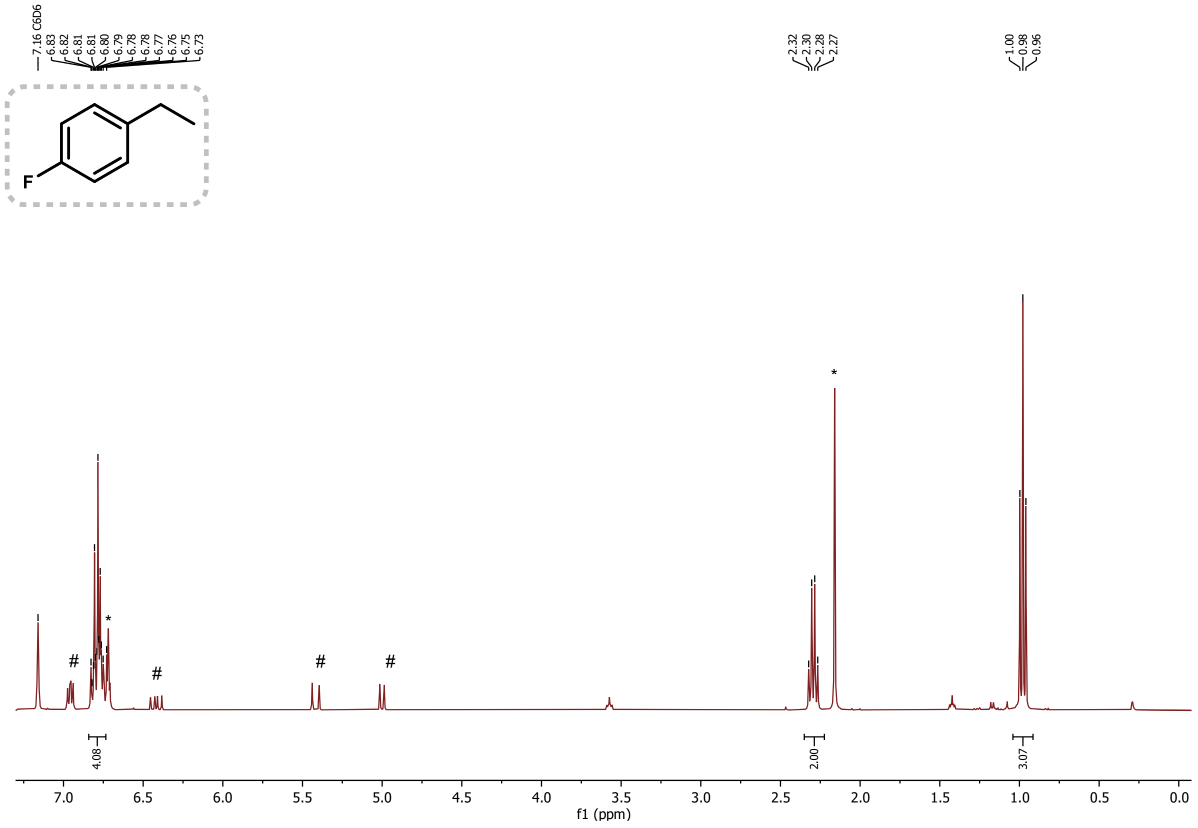

Figure S90. ^{1}H NMR spectrum (C_6D_6, 400 MHz, 298 K) of 4-(fluoro)-styrene, after 20 h at 60°C with 1 mol% of **2** as catalyst forming to 78% 1-ethyl-4-(fluoro)-benzene. *denotes the internal standard mesitylene. # denotes 22% of 4-(fluoro)-styrene.

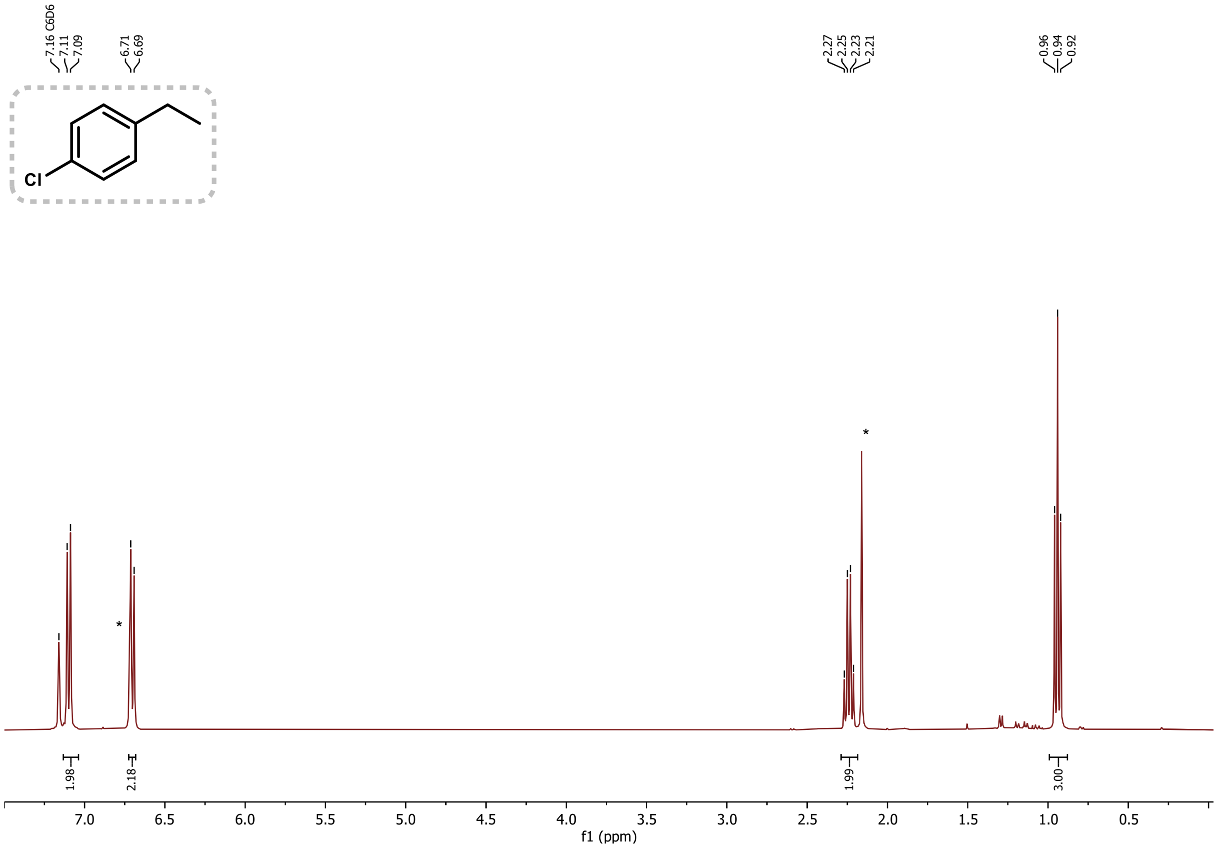

Figure S91. ^{1}H NMR spectrum (C_6D_6, 400 MHz, 298 K) of 4-(chloro)-styrene, after 2.5 h at 60°C with 1 mol% of **2** as catalyst forming to 1-ethyl-4-(chloro)-benzene. *denotes the internal standard mesitylene.

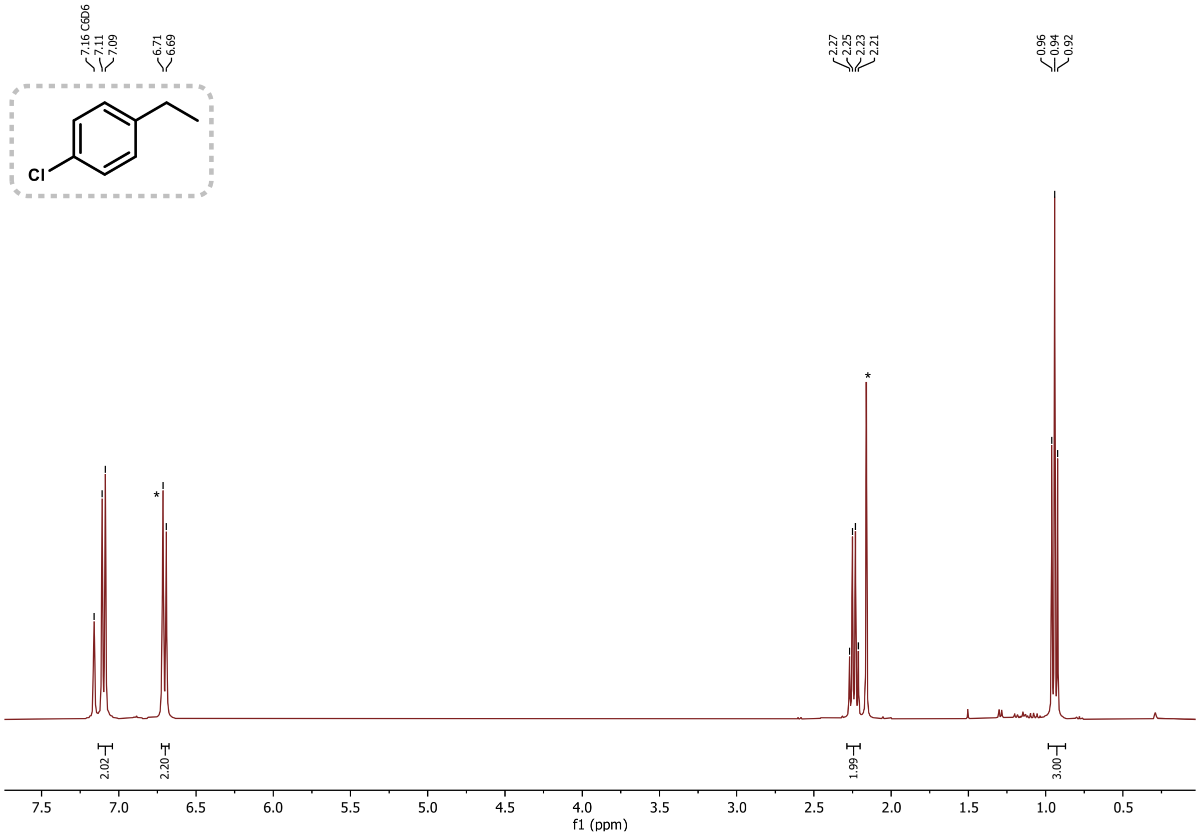

Figure S92. ^{1}H NMR spectrum (C_6D_6, 400 MHz, 298 K) of 4-(chloro)-styrene, after 20 h at 60°C with 1 mol% of **2** as catalyst forming to 1-ethyl-4-(chloro)-benzene. *denotes the internal standard mesitylene.

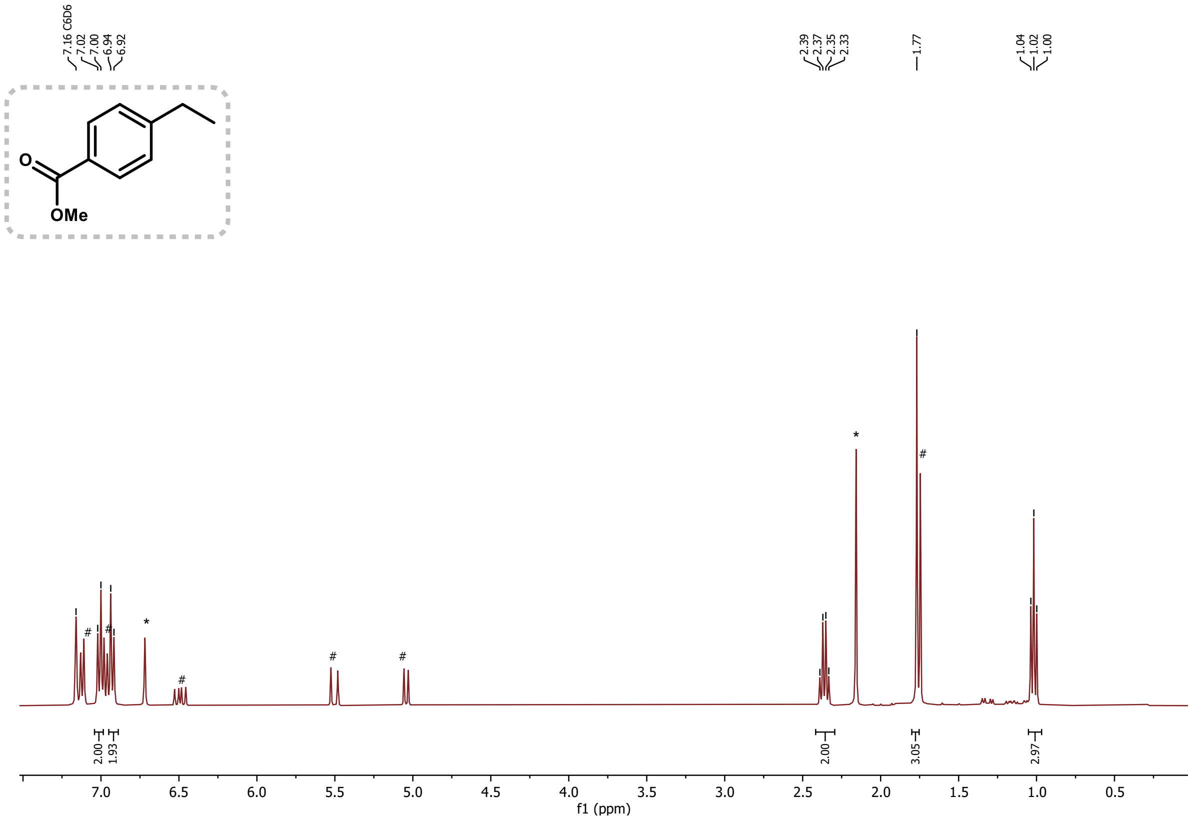

Figure S93. ^{1}H NMR spectrum (C_6D_6, 400 MHz, 298 K) of 4-(acetoxy)-styrene, after 20 h at 60°C with 1 mol% of **2** as catalyst forming 65% 1-ethyl-4-(acetoxy)-benzene. *denotes the internal standard mesitylene. # denotes 35% of 4-(acetoxy)-styrene.

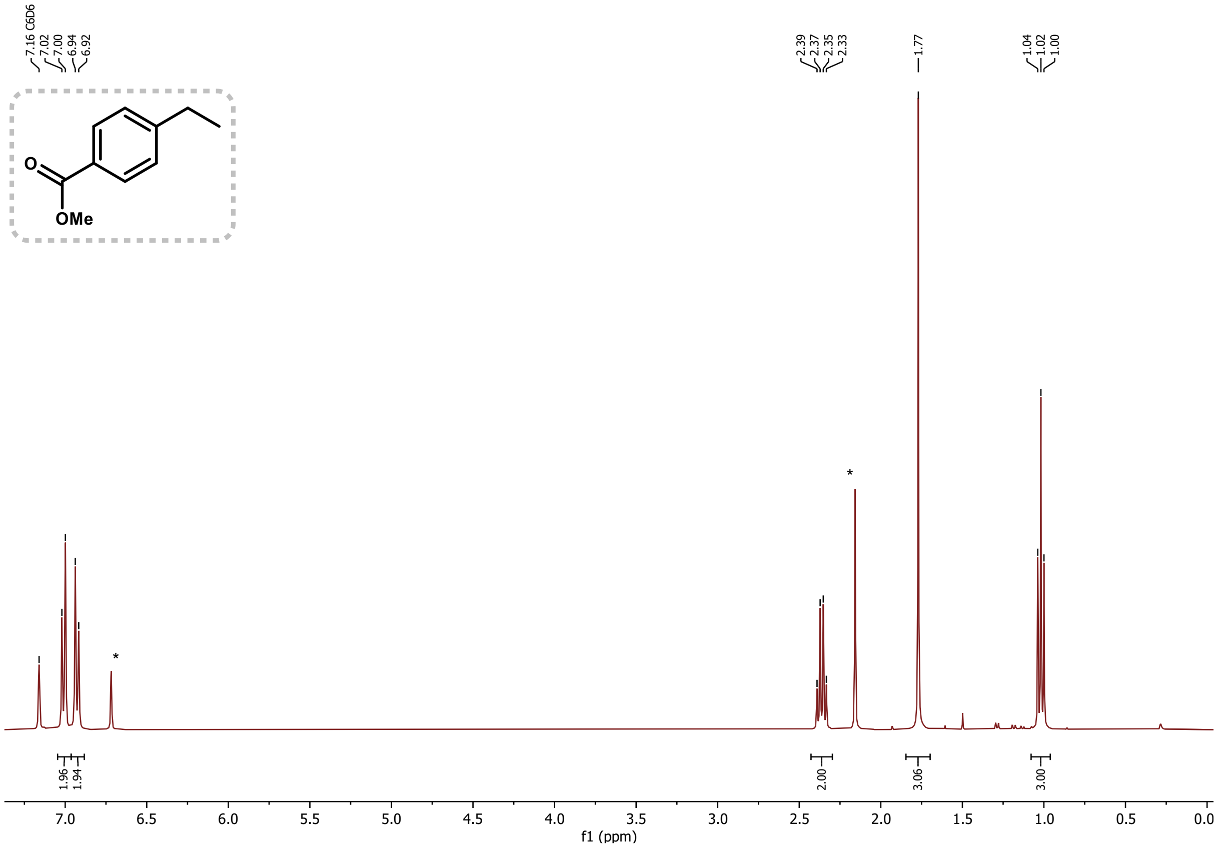

Figure S94. ^{1}H NMR spectrum (C_6D_6, 400 MHz, 298 K) of 4-(acetoxy)-styrene, after 20 h at 60°C with 1 mol% of **2** as catalyst forming to 1-ethyl-4-(acetoxy)-benzene. *denotes the internal standard mesitylene.

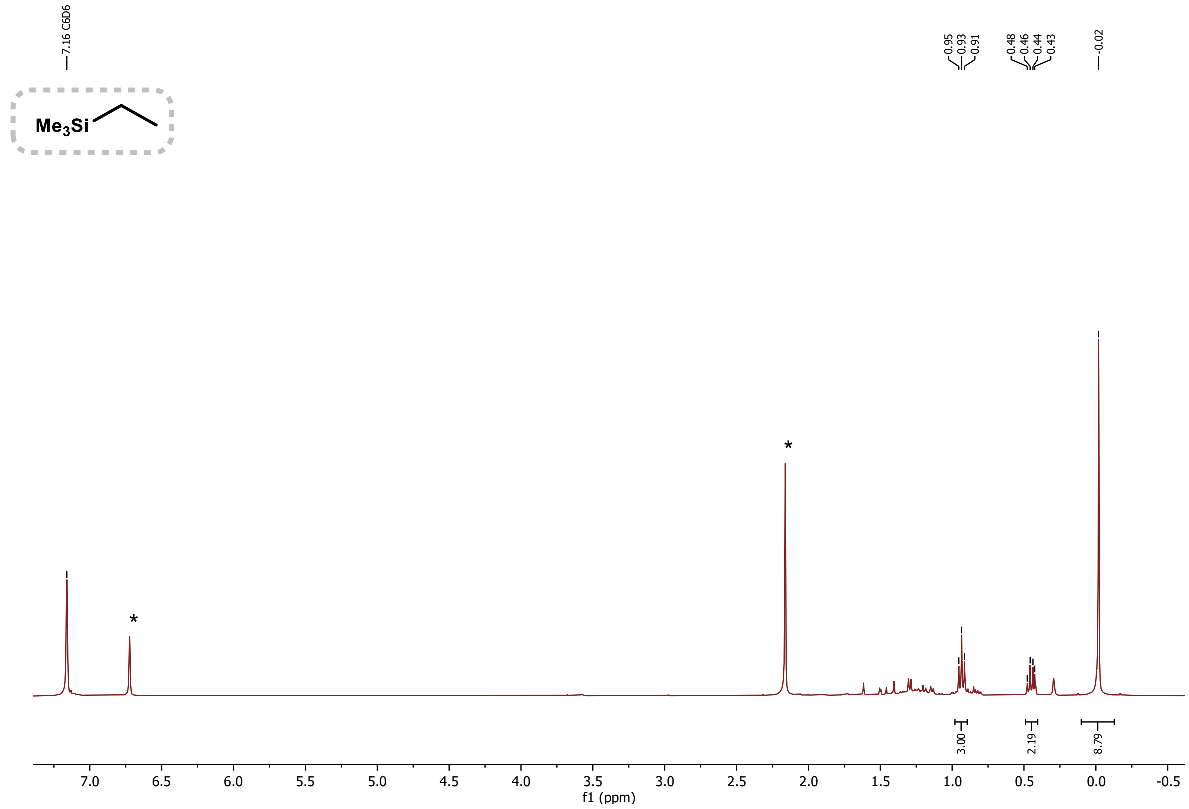

Figure S95. ^{1}H NMR spectrum (C_6D_6, 400 MHz, 298 K) of vinyltrimethylsilane after 5 h at RT with 1 mol% of **2** as catalyst forming trimethyl(ethyl)silane. *denotes the internal standard mesitylene.

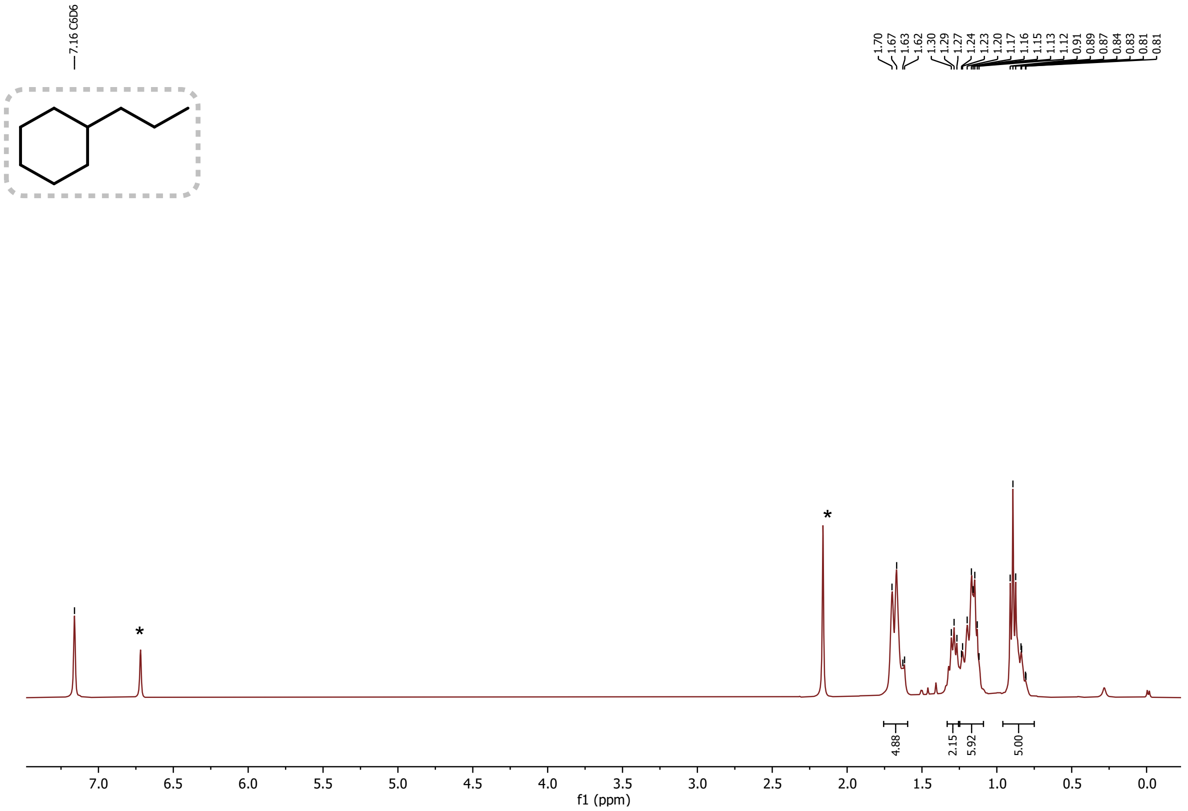

Figure S96. ^{1}H NMR spectrum (C_6D_6, 400 MHz, 298 K) of allyl-cyclohexane, after 20 h at 60°C with 1 mol% of **2** as catalyst forming propyl-cyclohexane. *denotes the internal standard mesitylene.

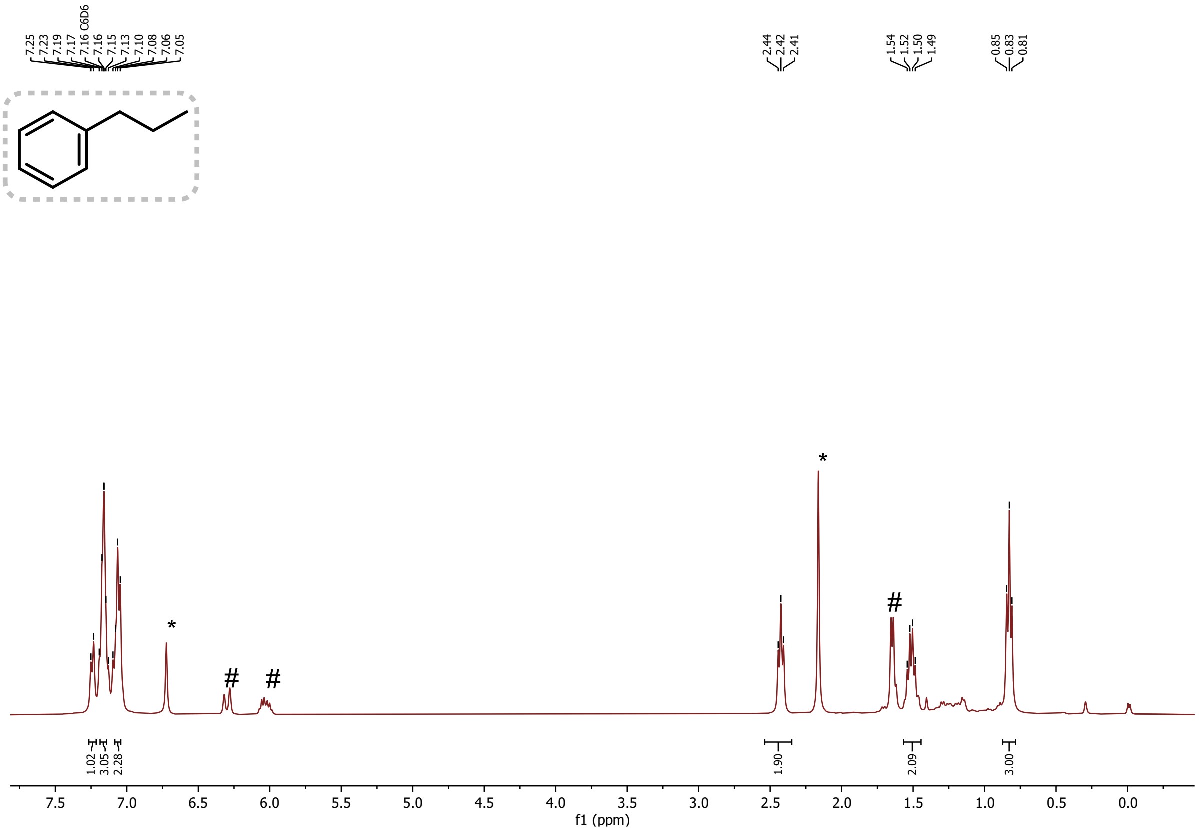

Figure S97. ^{1}H NMR spectrum (C_6D_6, 400 MHz, 298 K) of allyl-benzene, after 20 hours at 60°C with 1 mol% of **2** as catalyst forming propyl-benzene. *denotes the internal standard mesitylene. $^{\#}$denotes 32% isomerization to β-methylstyrene.

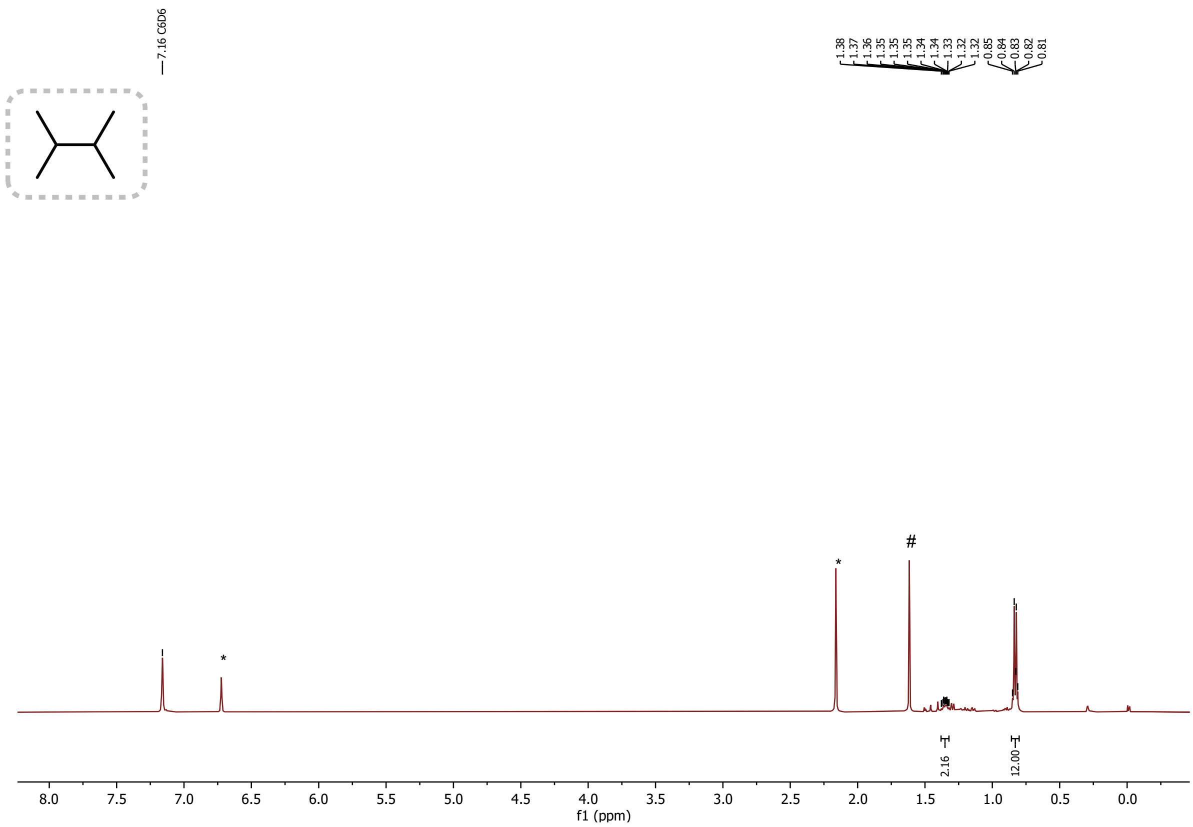

Figure S98. ^{1}H NMR spectrum (C_6D_6, 400 MHz, 298 K) of 2,3-dimethyl-1,4-butadiene, after 20 h at 60°C with 1 mol% of **2** as catalyst forming 2,3-dimethylbutane. *denotes the internal standard mesitylene. #denotes 35% isomerization to 2,3-dimethylbut-2-ene.

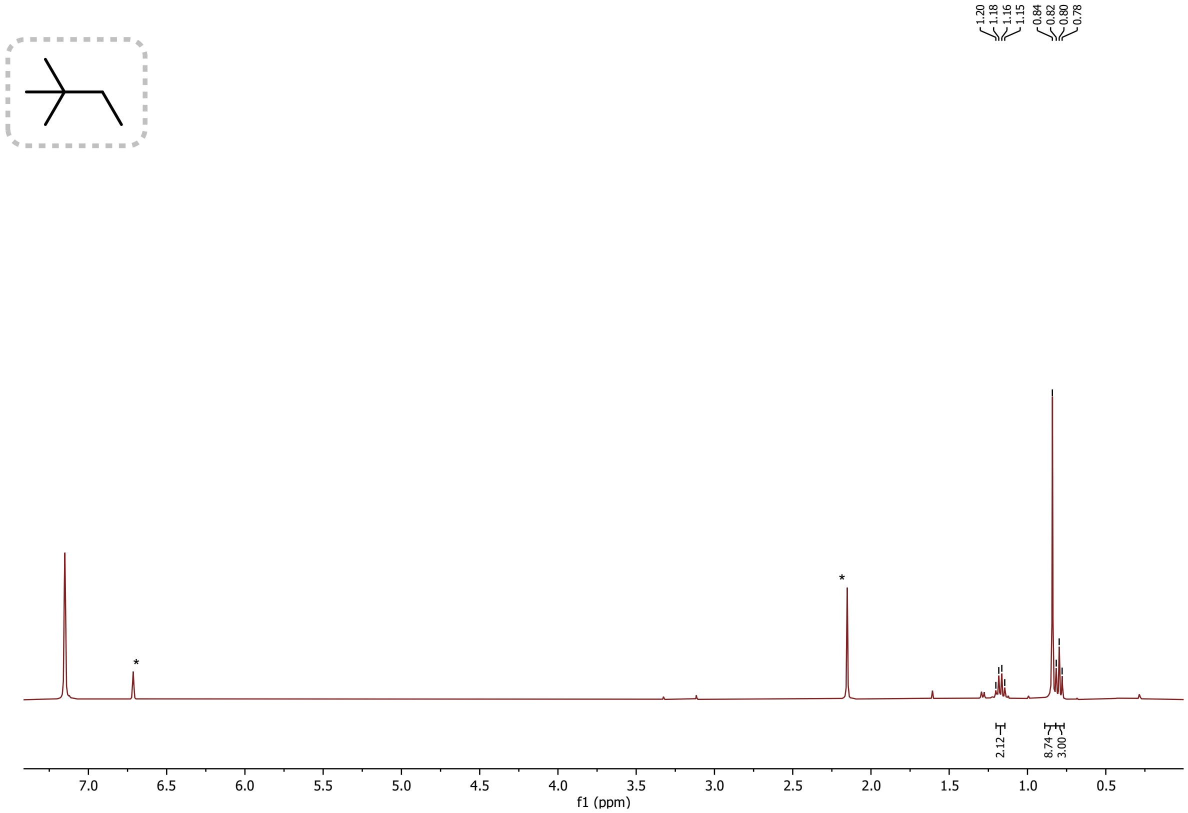

Figure S99. ^{1}H NMR spectrum (C_6D_6, 400 MHz, 298 K) of 3,3-dimethyl-but-1-ene, after 20 h at 60°C with 1 mol% of **2** as catalyst forming 3,3-dimethylbutane. *denotes the internal standard mesitylene.

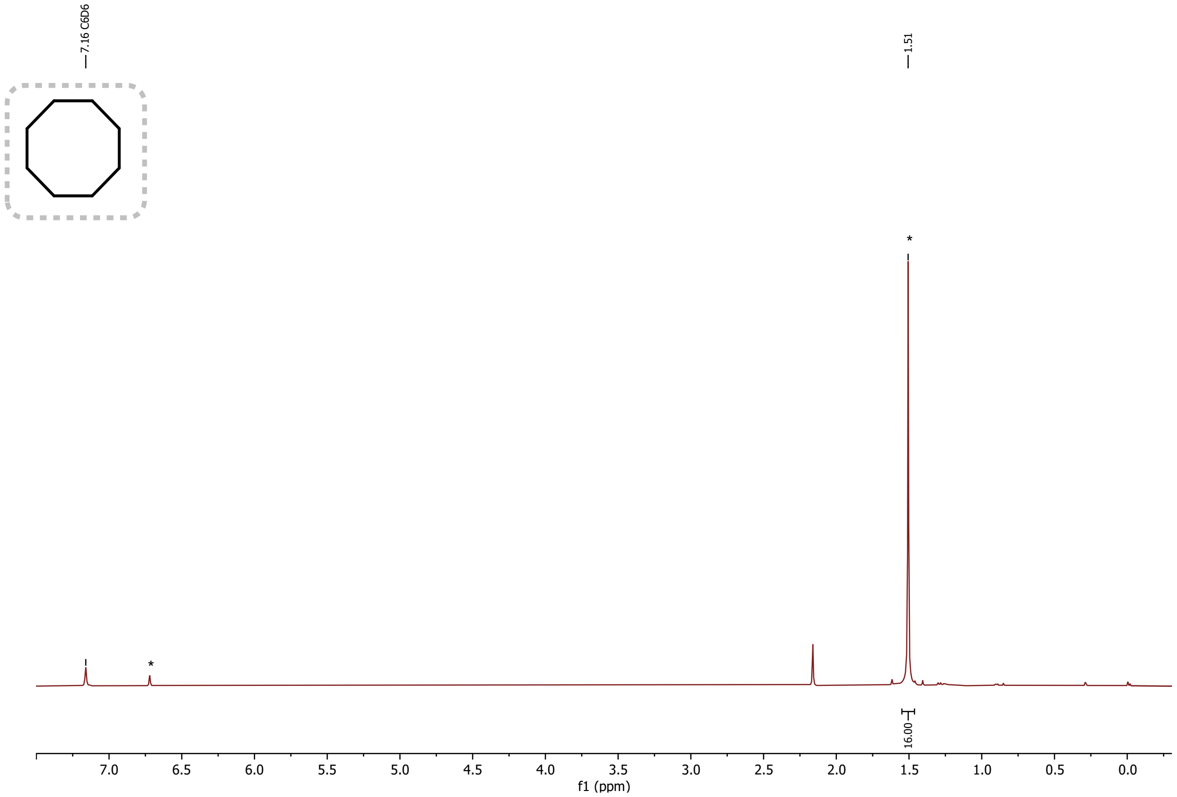

Figure S100. ^{1}H NMR spectrum (C_6D_6, 400 MHz, 298 K) of 1,3-cyclooctadiene, after 20 h at 60°C with 1 mol% of **2** as catalyst forming cyclooctane. *denotes the internal standard mesitylene.

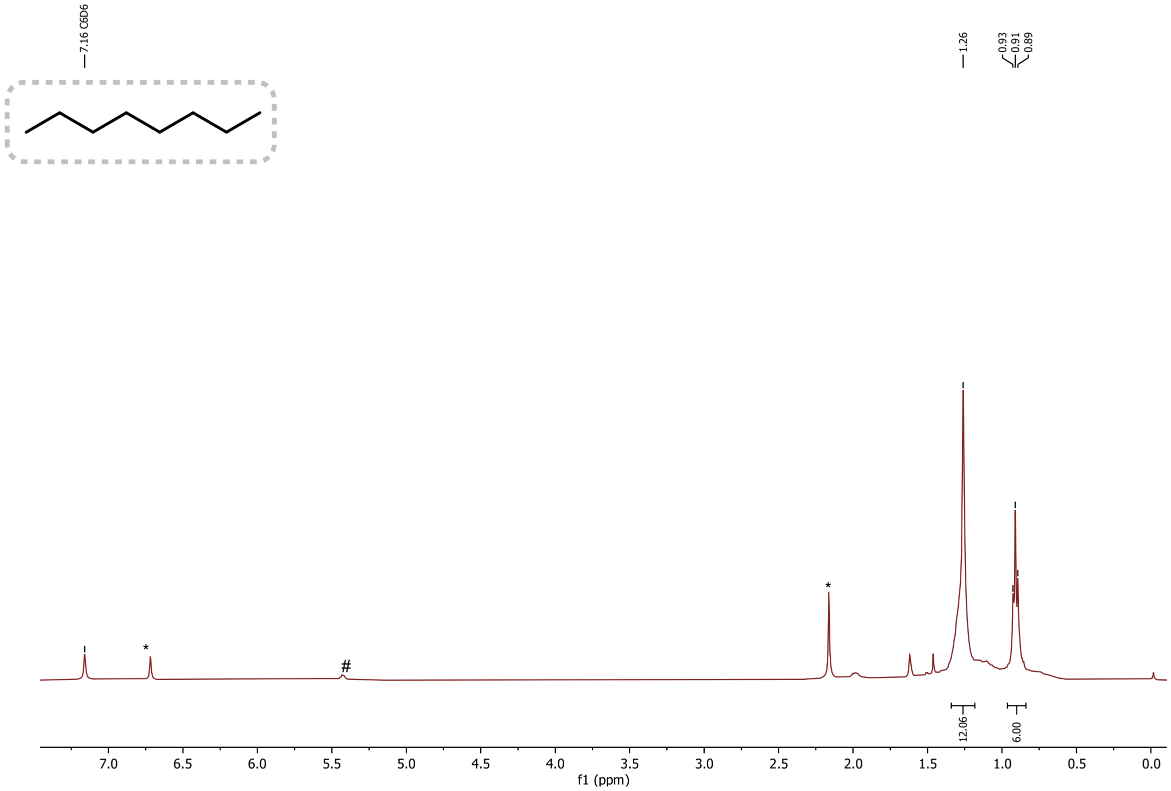

Figure S101. ^{1}H NMR spectrum (C_6D_6, 400 MHz, 298 K) of *trans*-2-octene, after 5 h at RT with 1 mol% of **2** as catalyst forming octane. *denotes the internal standard mesitylene. #denotes *trans*-2-octene.

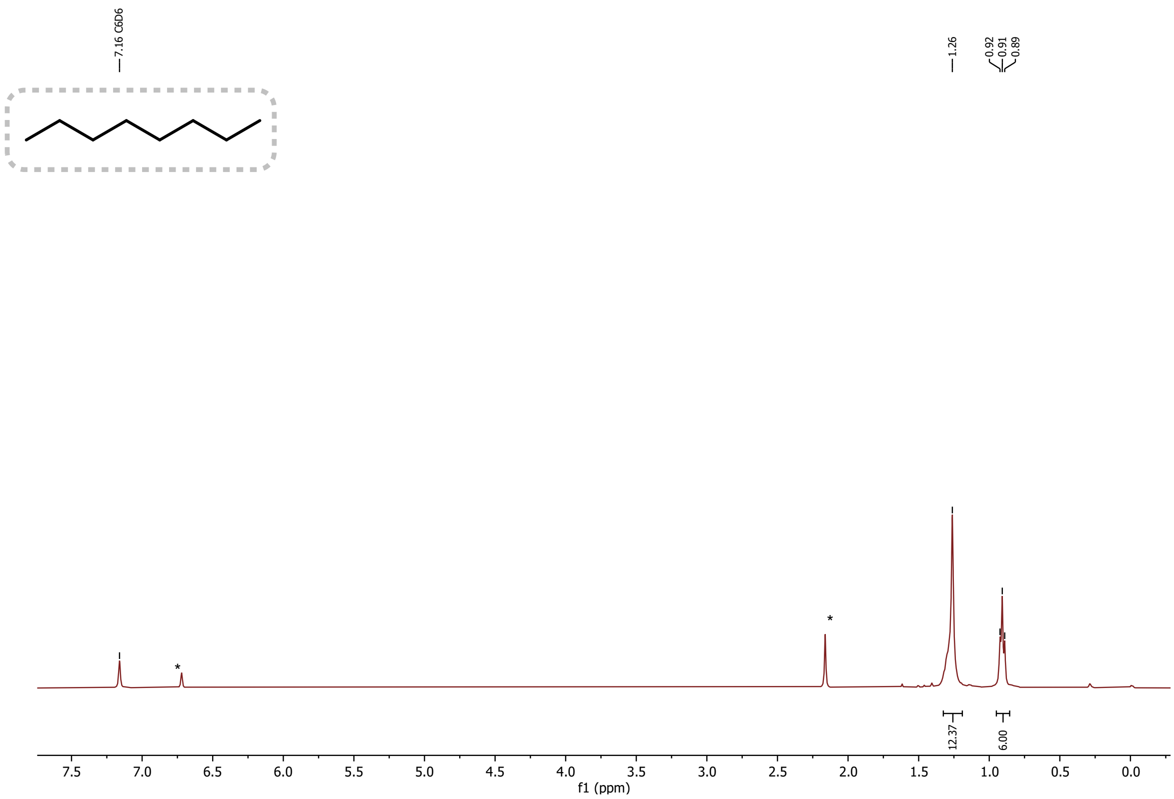

Figure S102. ^{1}H NMR spectrum (C_6D_6, 400 MHz, 298 K) of *trans*-2-octene, after 20 h at 60°C with 1 mol% of **2** as catalyst forming octane. *denotes the internal standard mesitylene.

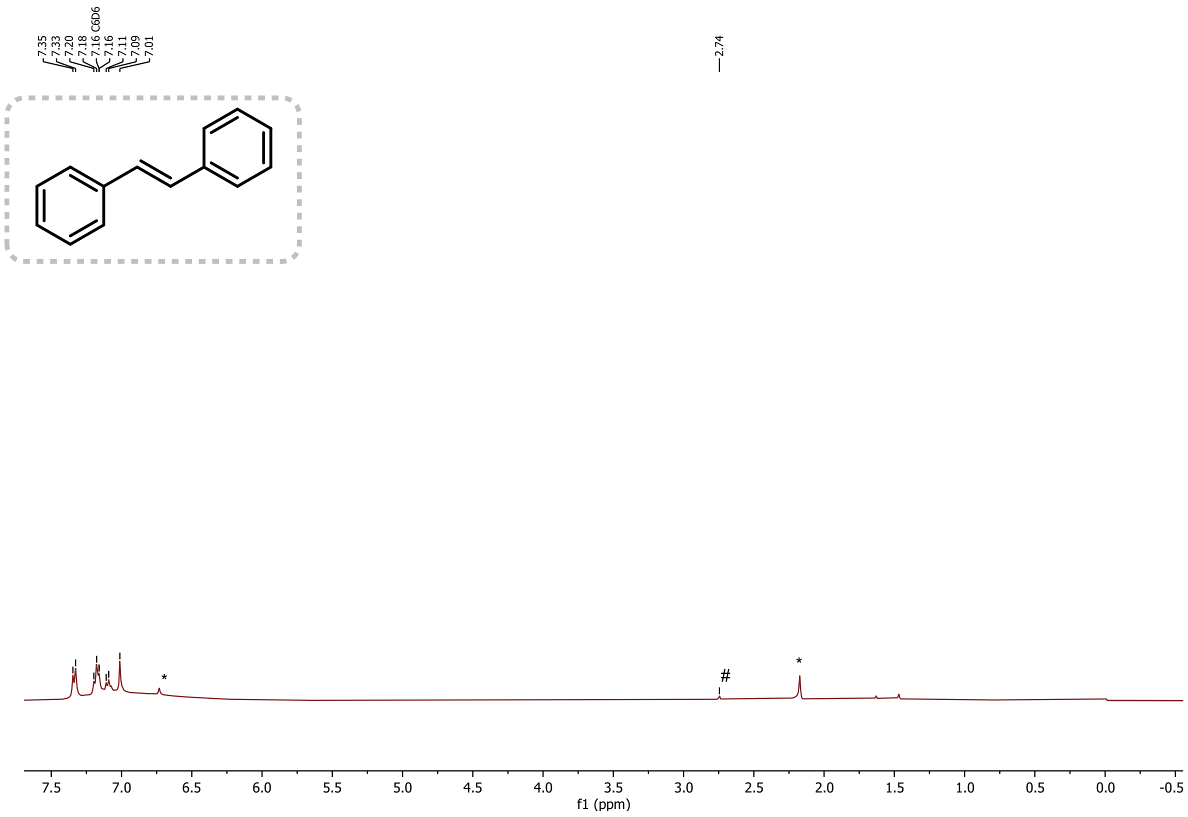

Figure S103. ^{1}H NMR spectrum (C_6D_6, 400 MHz, 298 K) of *trans*-stilbene, after 5 h at RT with 1 mol% of **2** as catalyst. *denotes the internal standard mesitylene. #denotes 1,2 diphenyl ethane.

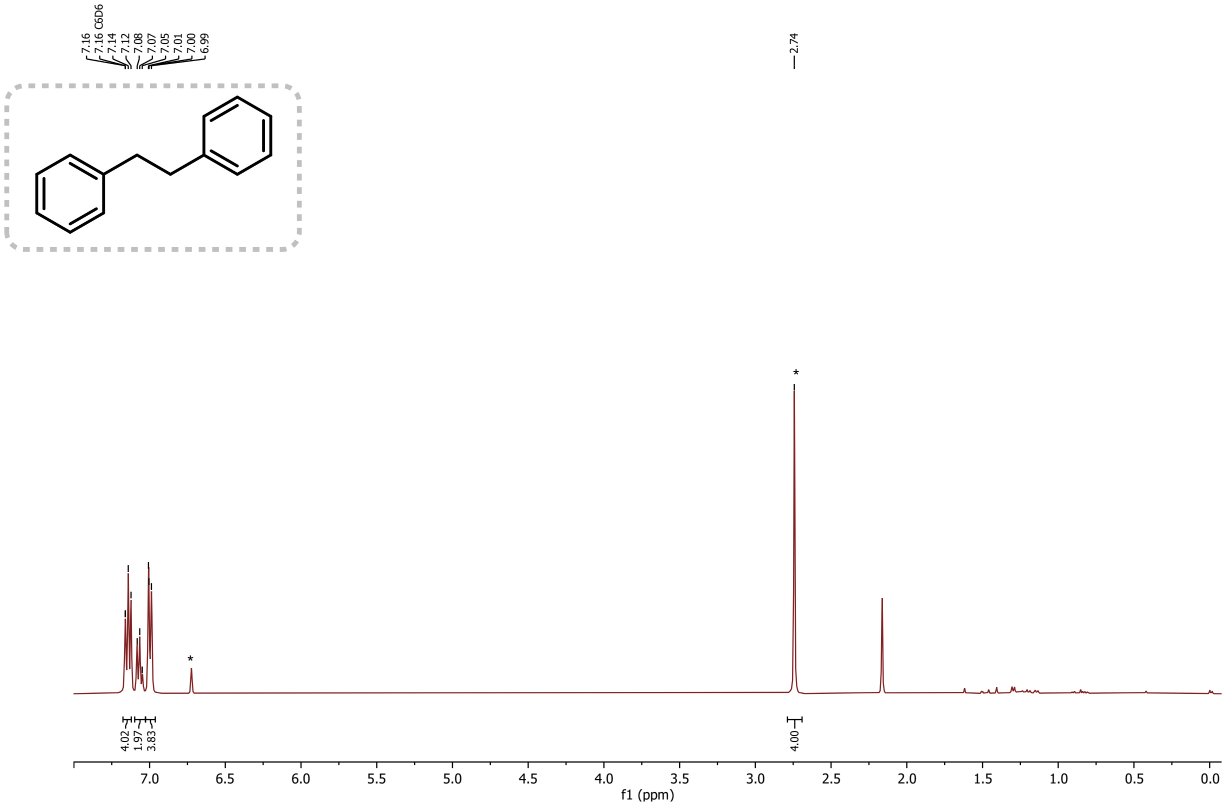

Figure S104. ^{1}H NMR spectrum (C_6D_6, 400 MHz, 298 K) of *trans*-stilbene, after 20 h at 60°C with 1 mol% of **2** as catalyst forming 1,2 diphenyl ethane. *denotes the internal standard mesitylene.

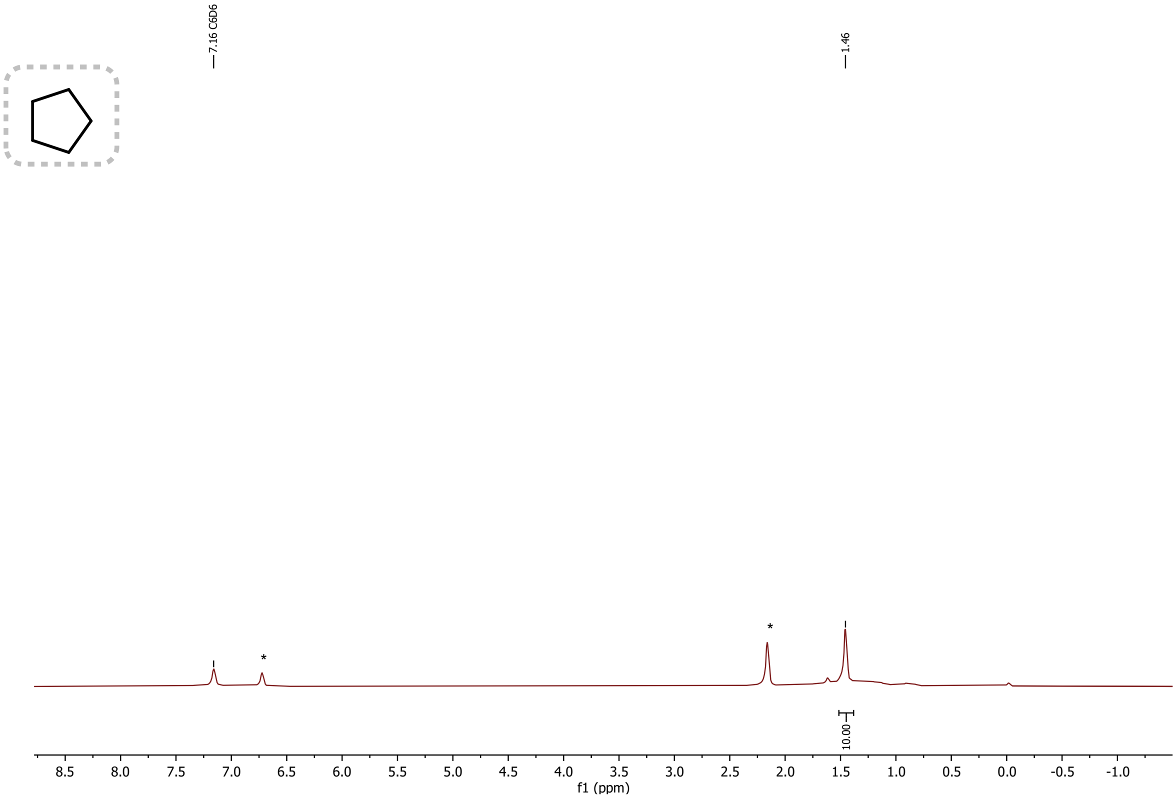

Figure S105. ^{1}H NMR spectrum (C_6D_6, 400 MHz, 298 K) of cyclopentene, after 5 h at RT with 1 mol% of **2** as catalyst forming cyclopentane. *denotes the internal standard mesitylene.

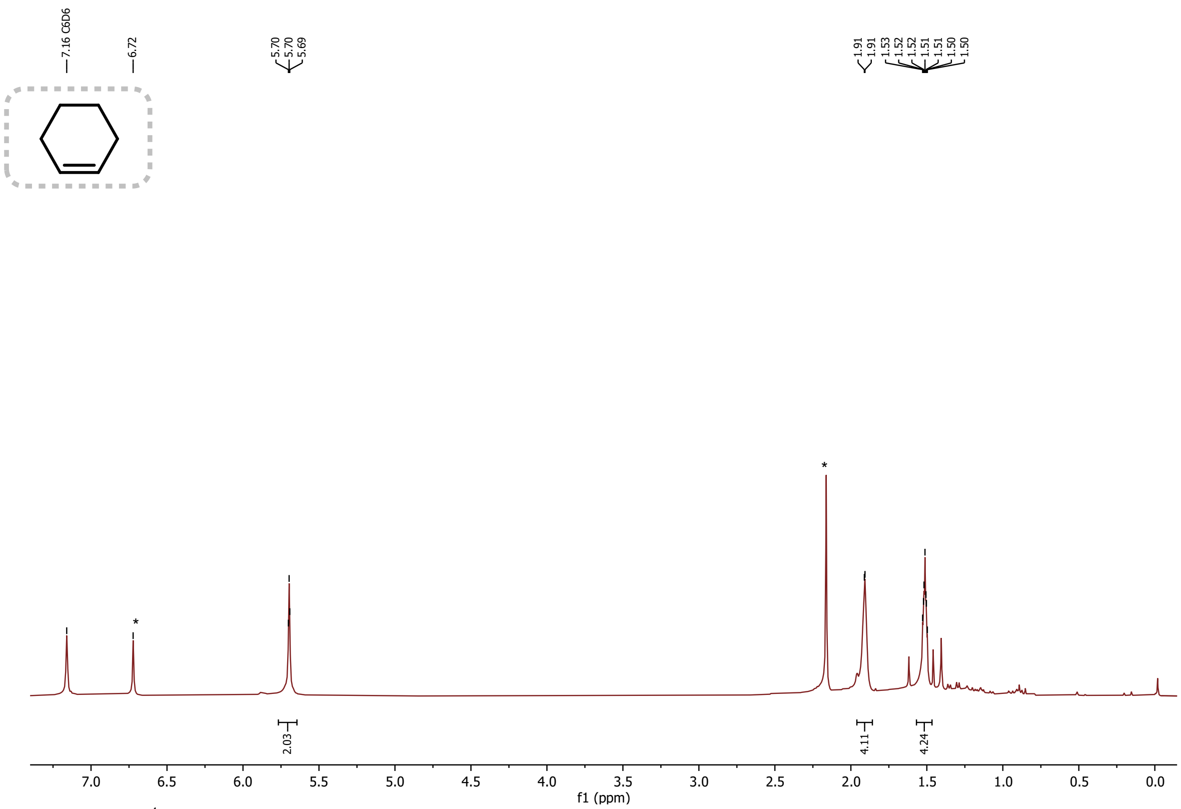

Figure S106. ^{1}H NMR spectrum (C_6D_6, 400 MHz, 298 K) of 1,4-cyclohexadiene, after 5 h at RT with 1 mol% of **2** as catalyst forming cyclohexene. *denotes the internal standard mesitylene.

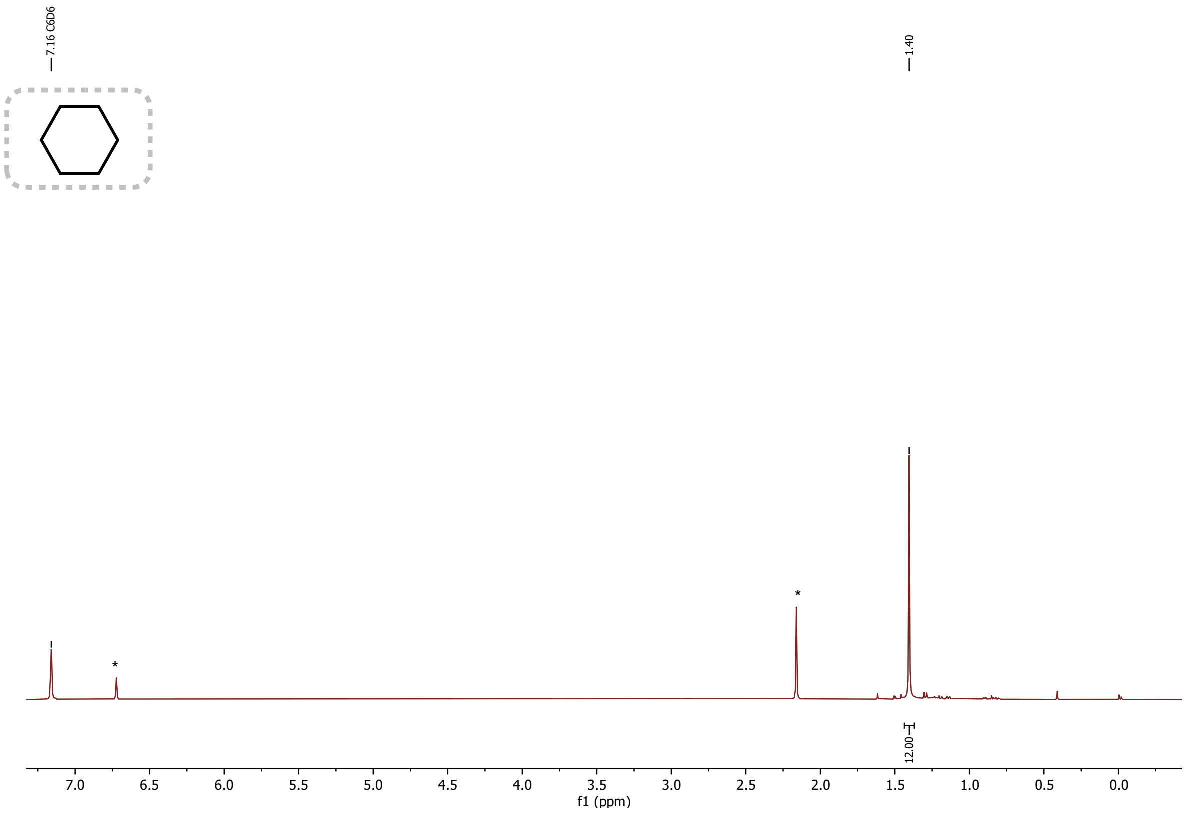

Figure S107. ^{1}H NMR spectrum (C_6D_6, 400 MHz, 298 K) of 1,4-cyclohexadiene, after 20 h at 60°C with 1 mol% of **2** as catalyst forming cyclohexane. *denotes the internal standard mesitylene.

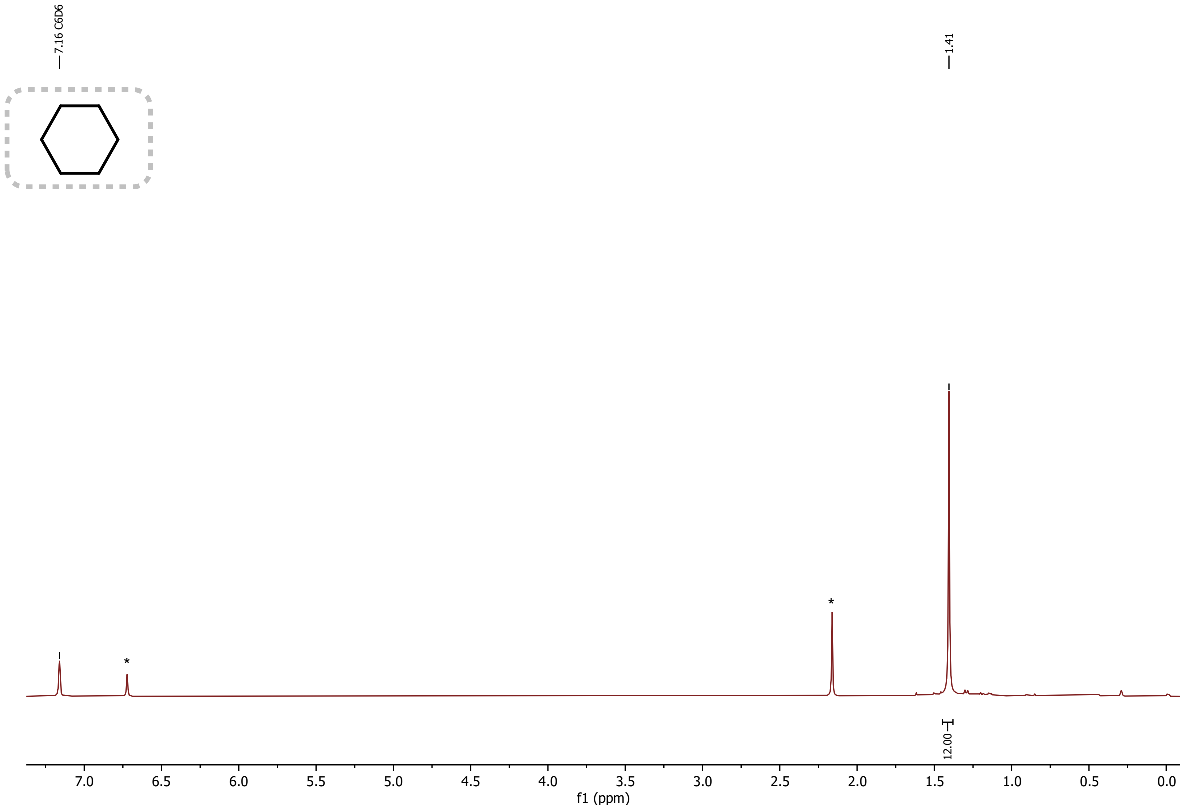

Figure S108. ^{1}H NMR spectrum (C_6D_6, 400 MHz, 298 K) of 1,3-cyclohexadiene, after 20 h at 60°C with 1 mol% of **2** as catalyst forming cyclohexane. *denotes the internal standard mesitylene.

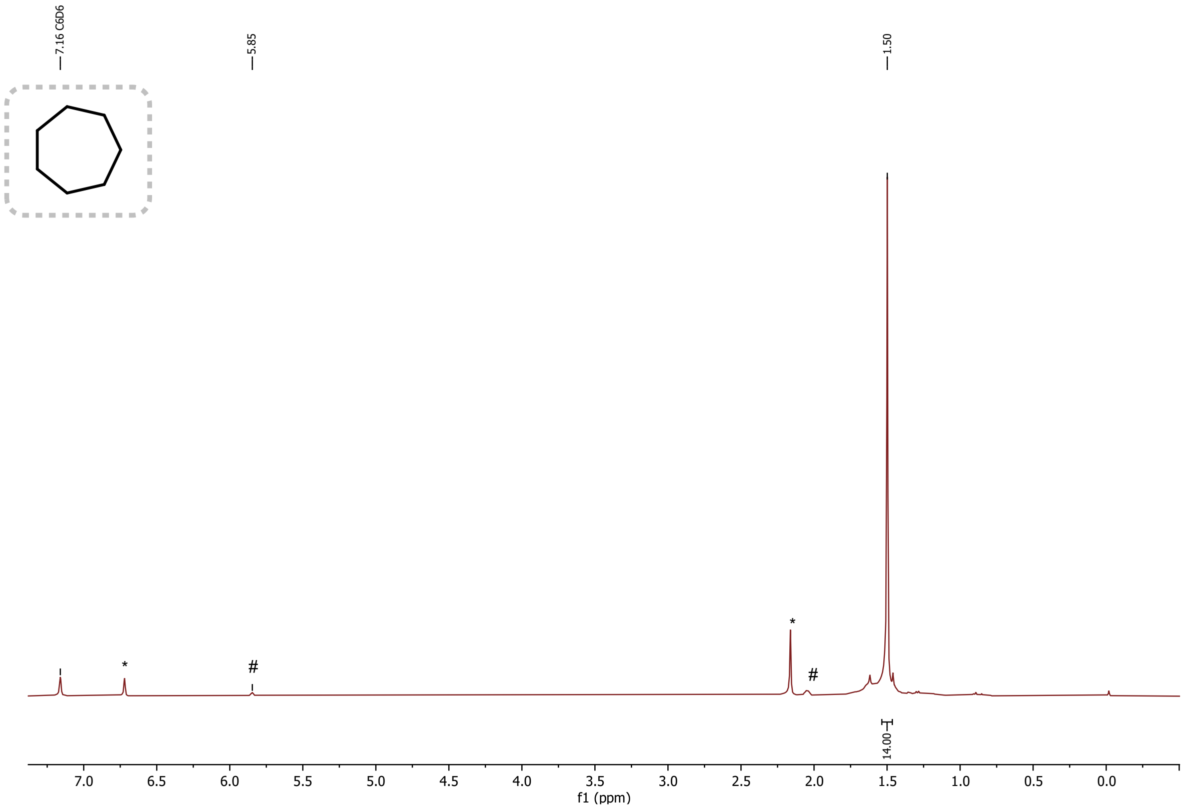

Figure S109. ^{1}H NMR spectrum (C_6D_6, 400 MHz, 298 K) of cycloheptene, after 5 h at RT with 1 mol% of **2** as catalyst forming cycloheptane. *denotes the internal standard mesitylene. # denotes cycloheptene.

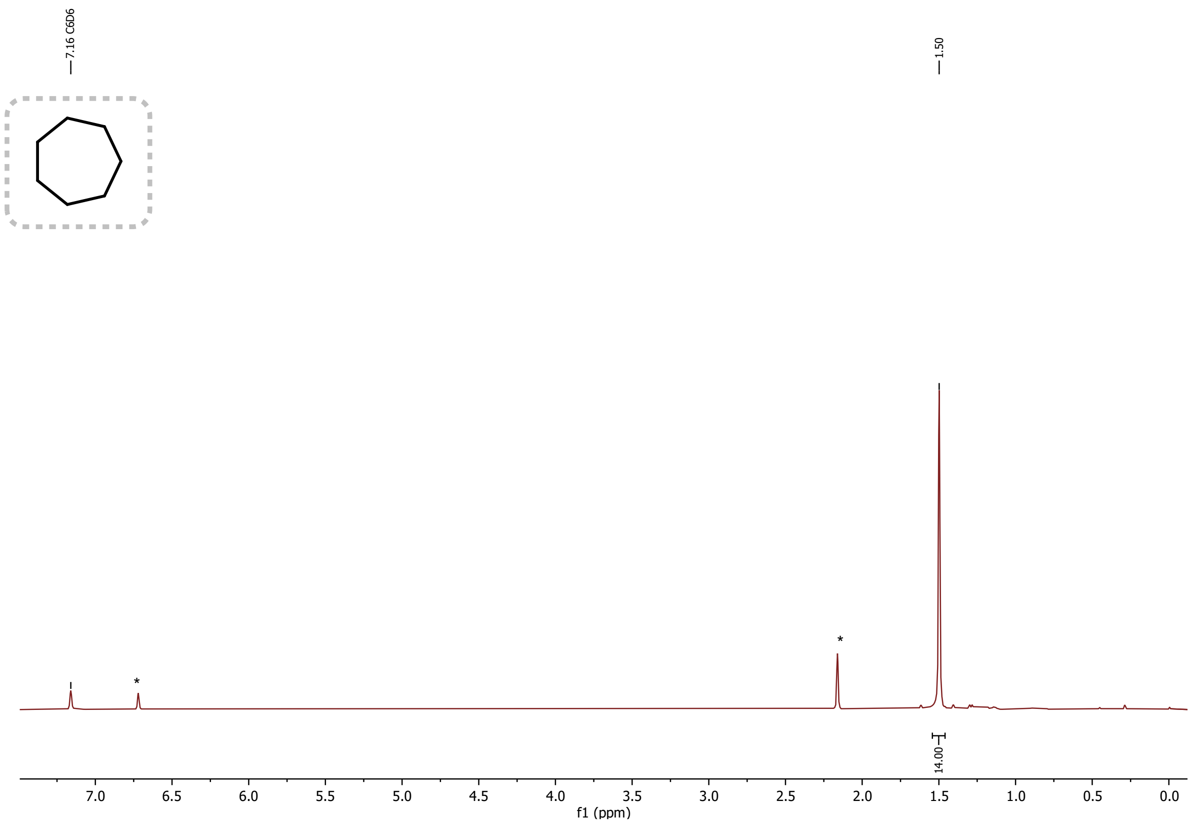

Figure S110. ^{1}H NMR spectrum (C_6D_6, 400 MHz, 298 K) of cycloheptene, after 20 h at 60°C with 1 mol% of **2** as catalyst forming cycloheptane. *denotes the internal standard mesitylene.

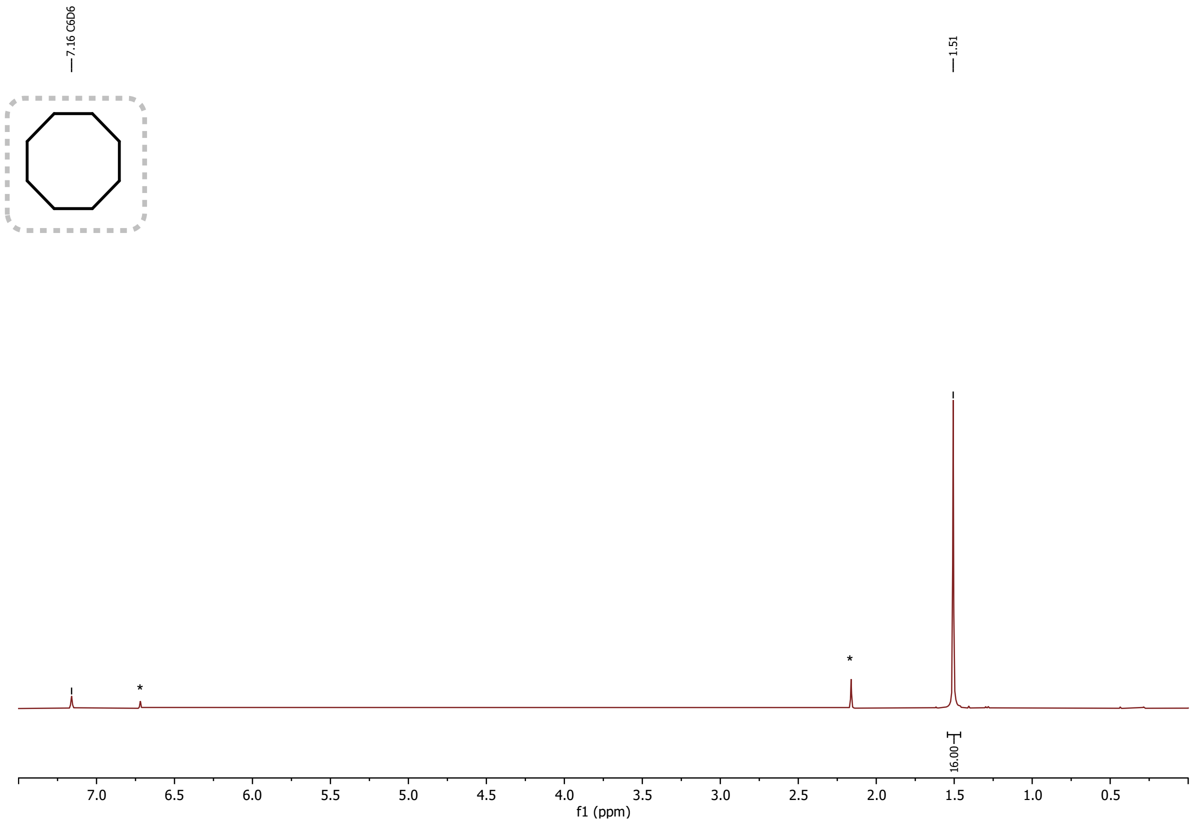

Figure S111. ^{1}H NMR spectrum (C_6D_6, 400 MHz, 298 K) of 1,5-cyclooctadiene, after 20 h at 60°C with 1 mol% of **2** as catalyst forming cyclooctane. *denotes the internal standard mesitylene.

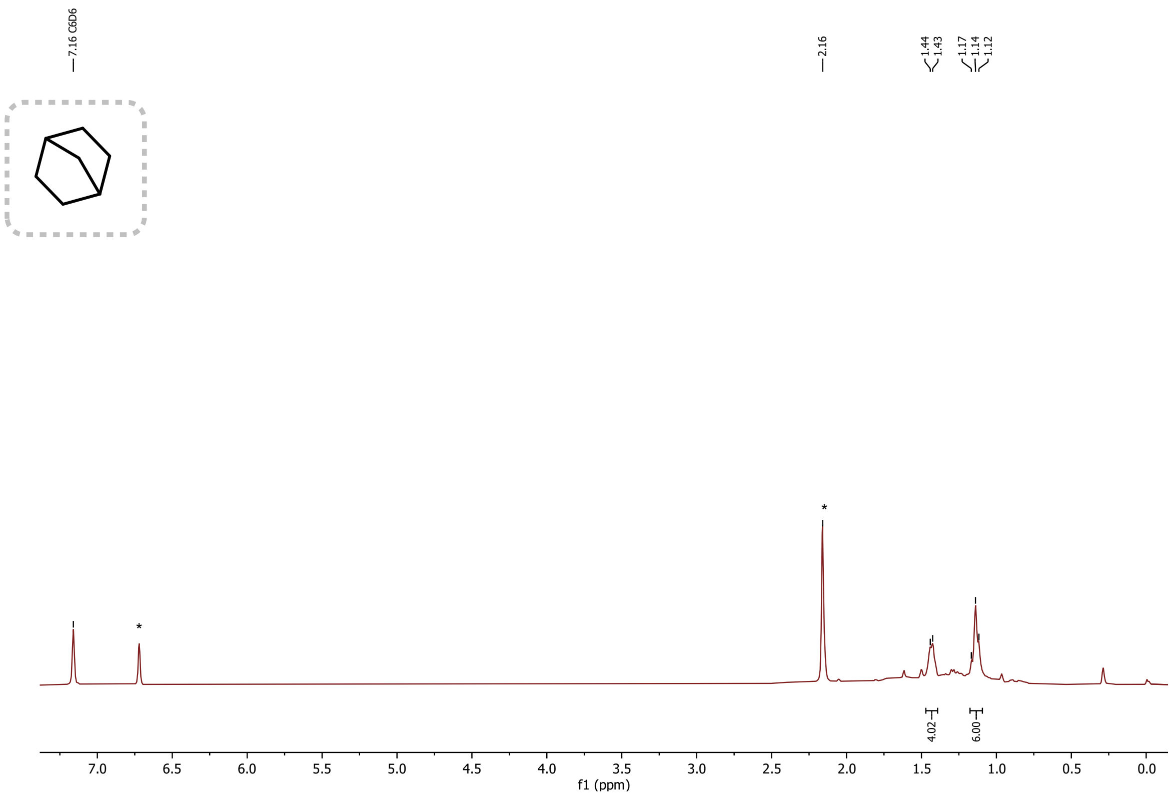

Figure S112. ^{1}H NMR spectrum (C_6D_6, 400 MHz, 298 K) of 2,5-norbornadiene, after 20 h at 60°C with 1 mol% of **2** as catalyst forming norbornane. *denotes the internal standard mesitylene. (multiplet at 2.14 of norbornane overlayered by internal standard mesitylene.)

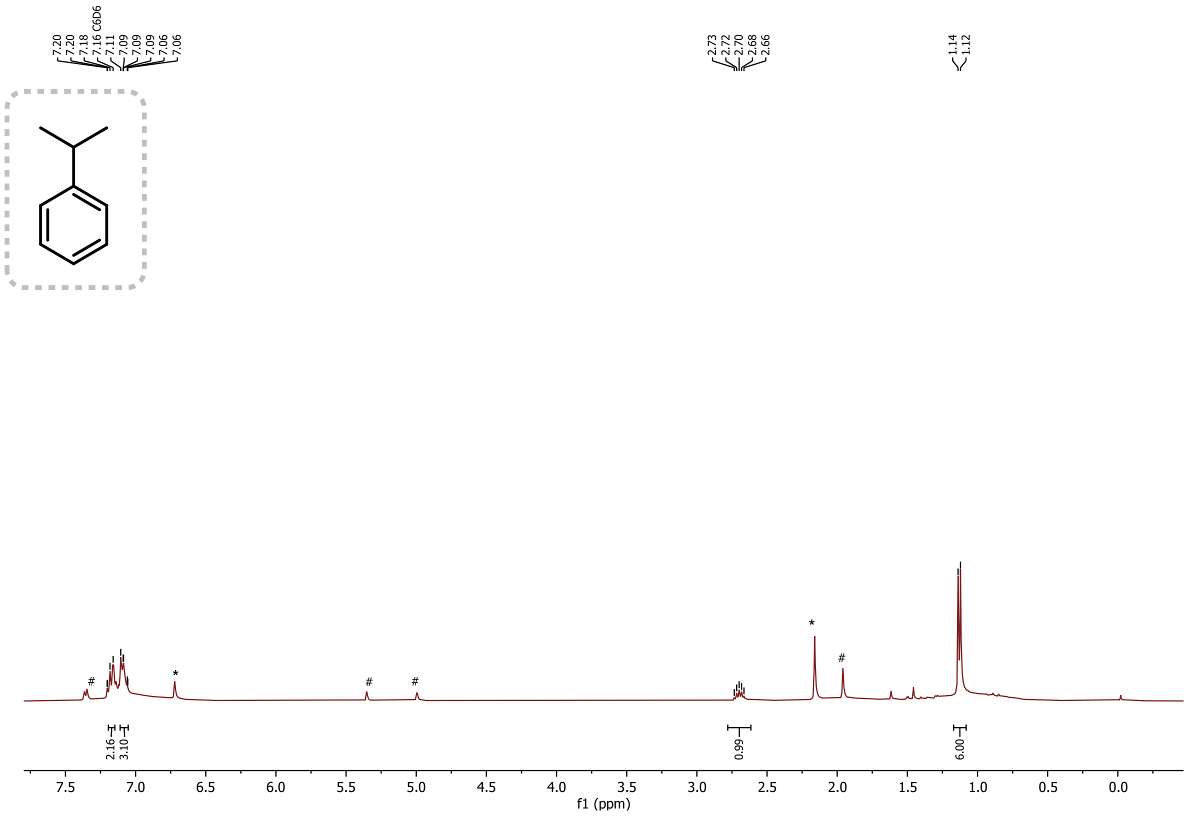

Figure S113. ^{1}H NMR spectrum (C_6D_6, 400 MHz, 298 K) of *α*-methylstyrene, after 5 h at RT with 1 mol% of **2** as catalyst forming *iso*-propylbenzene. *denotes the internal standard mesitylene. #denotes α-methylstyrene.

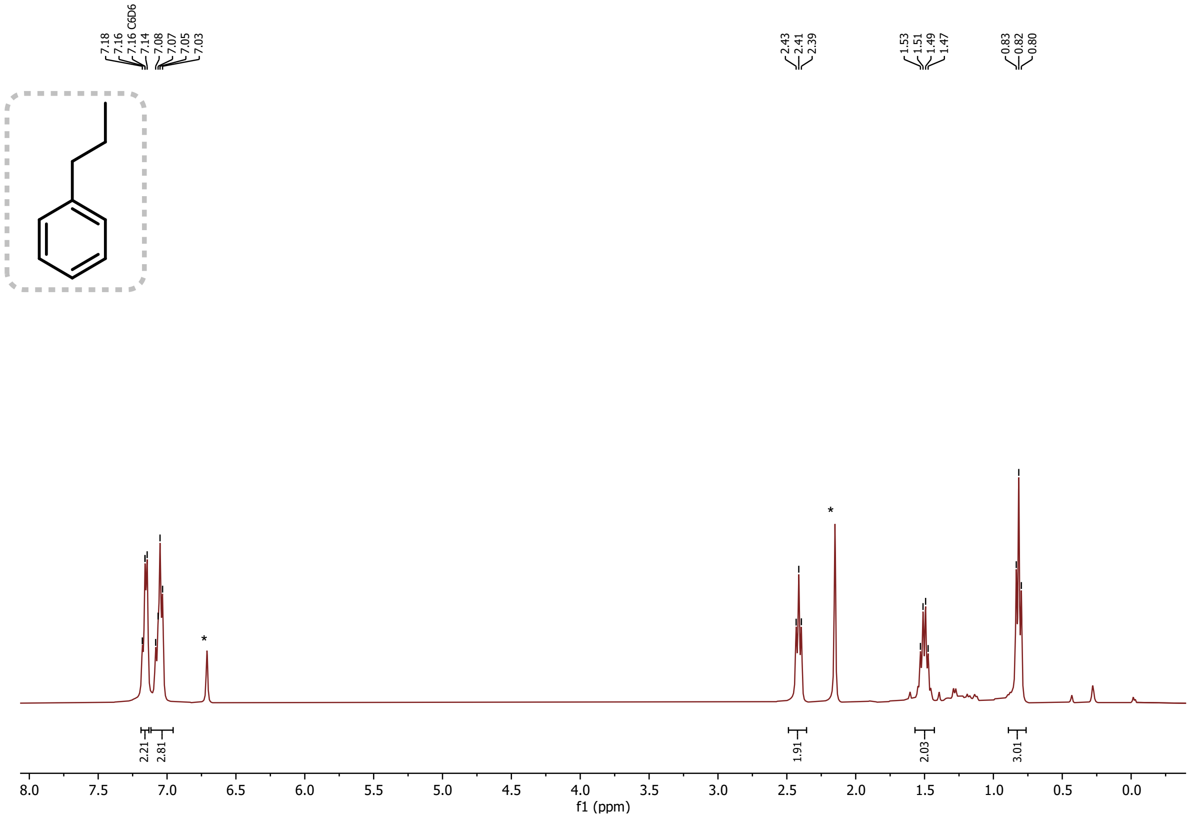

Figure S114. ^{1}H NMR spectrum (C_6D_6, 400 MHz, 298 K) of *β*-methylstyrene, after 20 h at 60°C with 1 mol% of **2** as catalyst forming propylbenzene. *denotes the internal standard mesitylene.

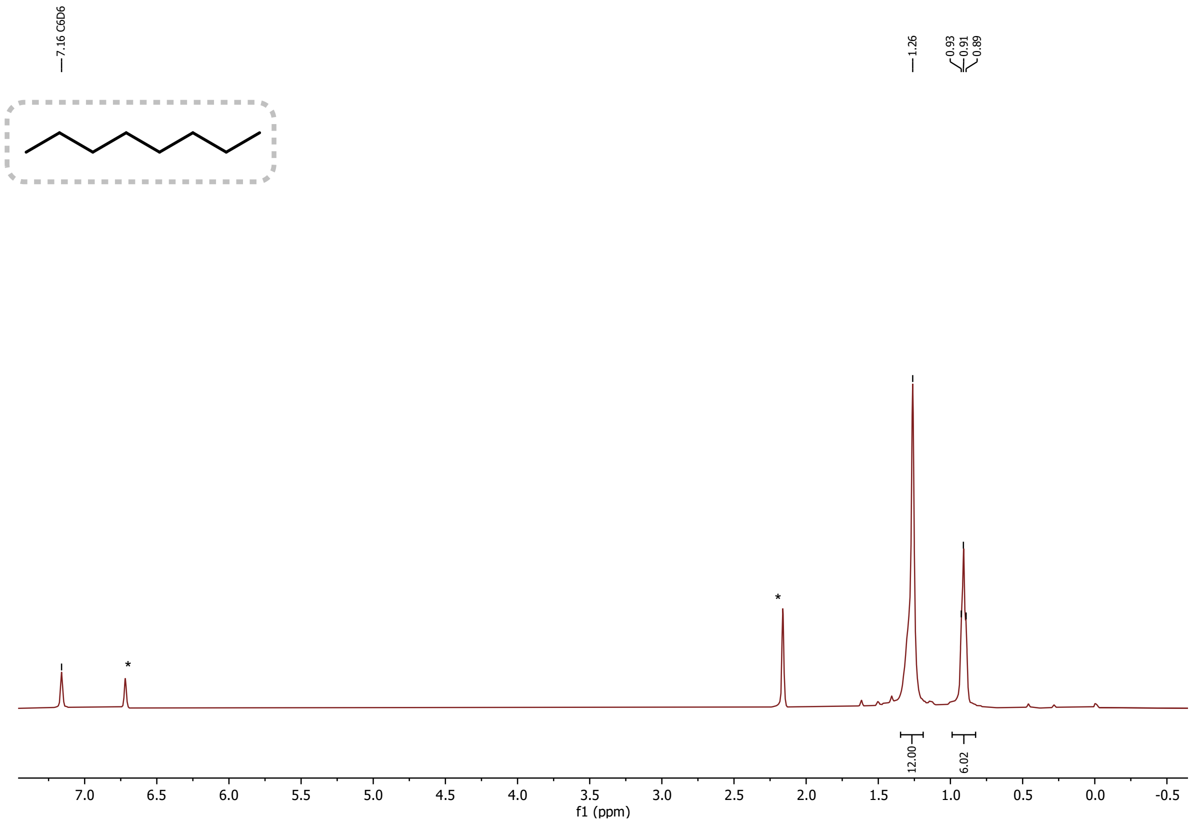

Figure S115. ^{1}H NMR spectrum (C_6D_6, 400 MHz, 298 K) of *trans*-4-octene, after 5 h at RT with 1 mol% of **2** as catalyst forming octane. *denotes the internal standard mesitylene.

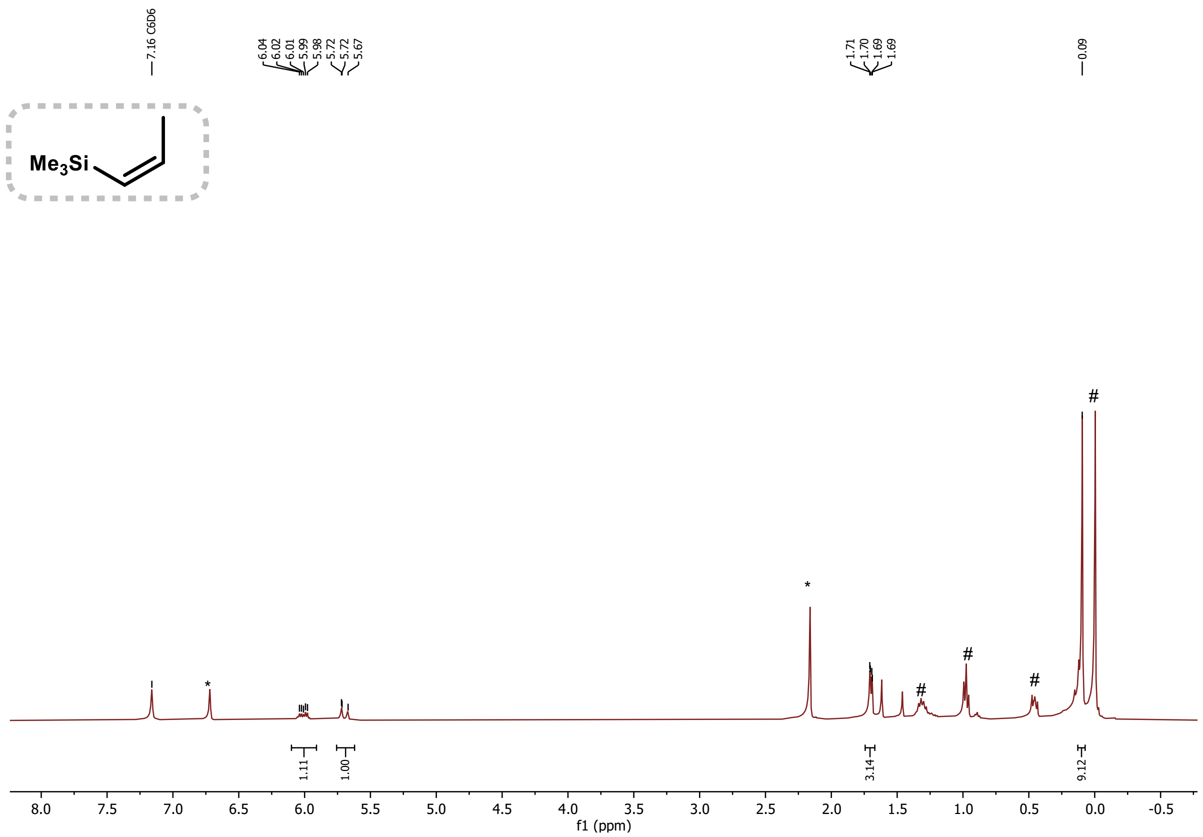

Figure S116. ^{1}H NMR spectrum (C_6D_6, 400 MHz, 298 K) of 1-trimethylsilyl-2-propyne, after 5 h at RT with 1 mol% of **2** as catalyst forming 1-trimethylsilyl-prop-2-ene. *denotes the internal standard mesitylene. #denotes trimethyl(propyl)silane.

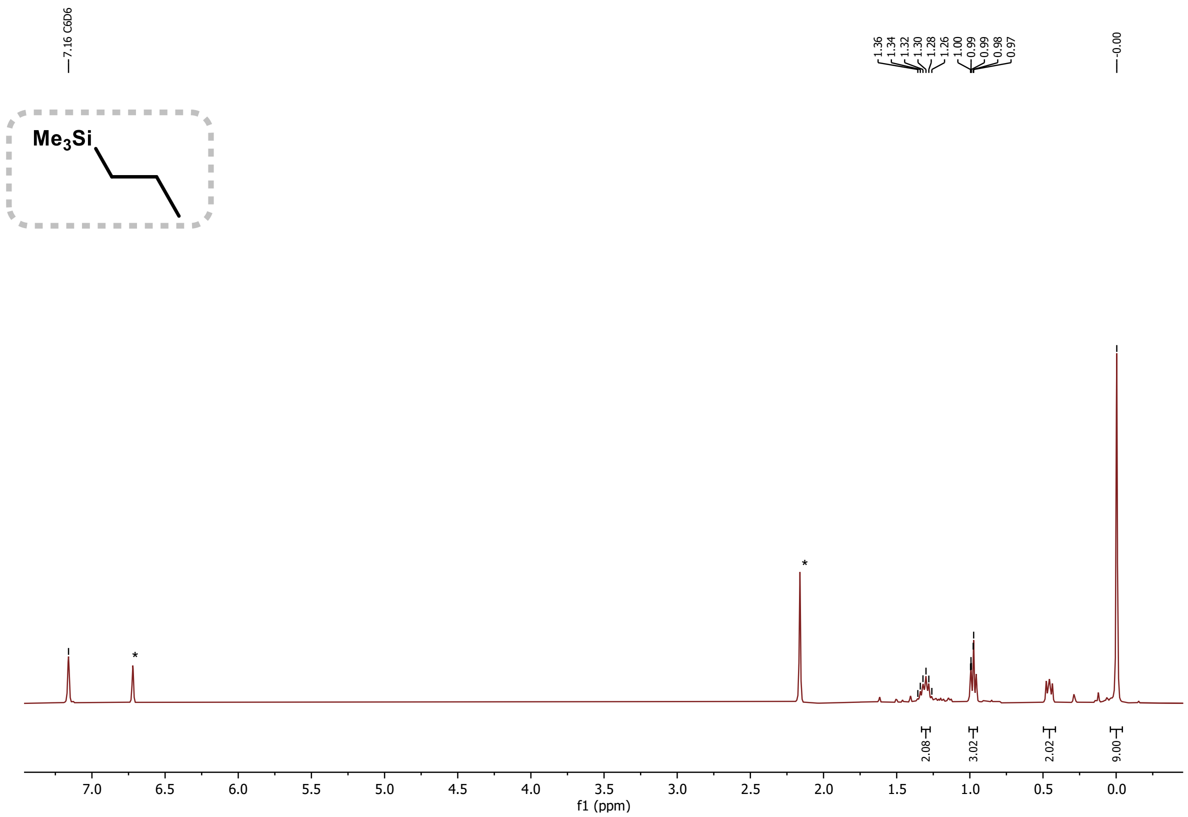

Figure S117. ^{1}H NMR spectrum (C_6D_6, 400 MHz, 298 K) of 1-trimethylsilyl-2-propyne, after 20 h at 60°C with 1 mol% of **2** as catalyst forming trimethyl(propyl)silane. *denotes the internal standard mesitylene.

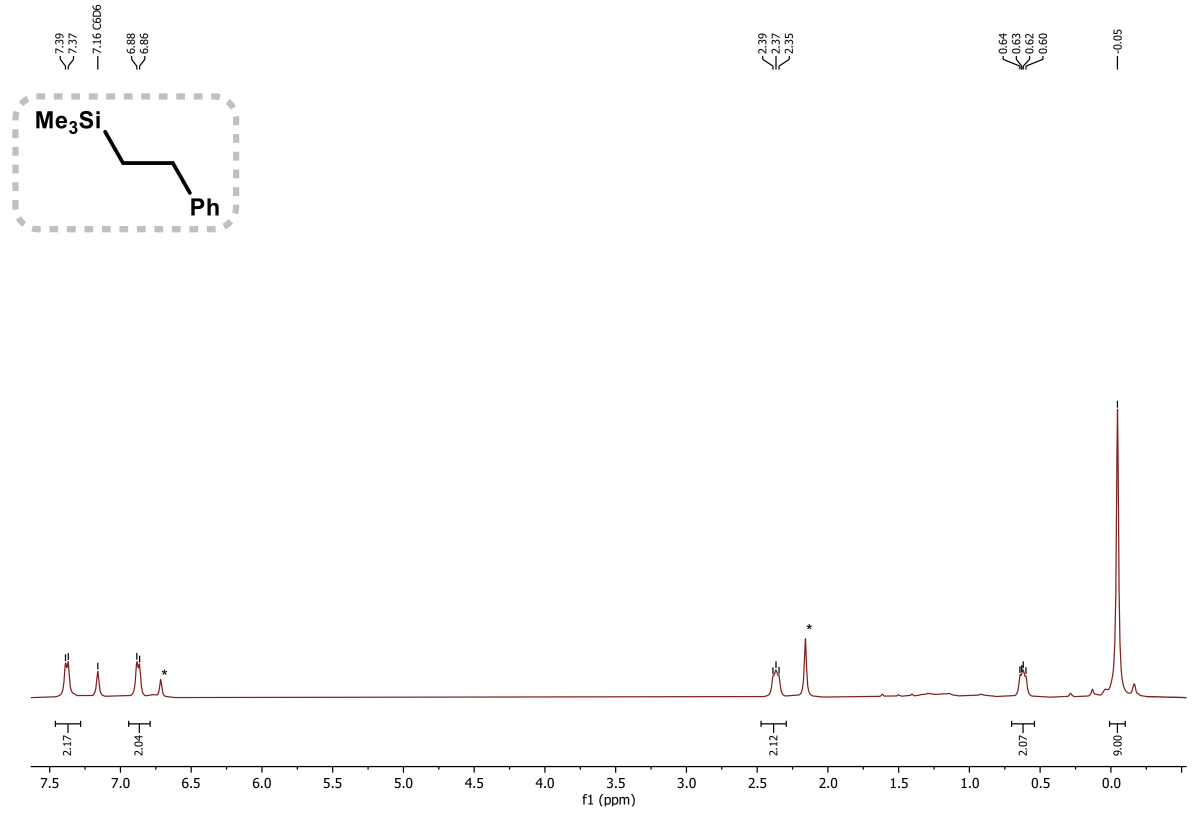

Figure S118. ^{1}H NMR spectrum (C_6D_6, 400 MHz, 298 K) of 1-phenyl-2-trimethylsilylacetylene, after 20 h at 60°C with 1 mol% of **2** as catalyst forming trimethyl(phenylethyl)silane. *denotes the internal standard mesitylene.

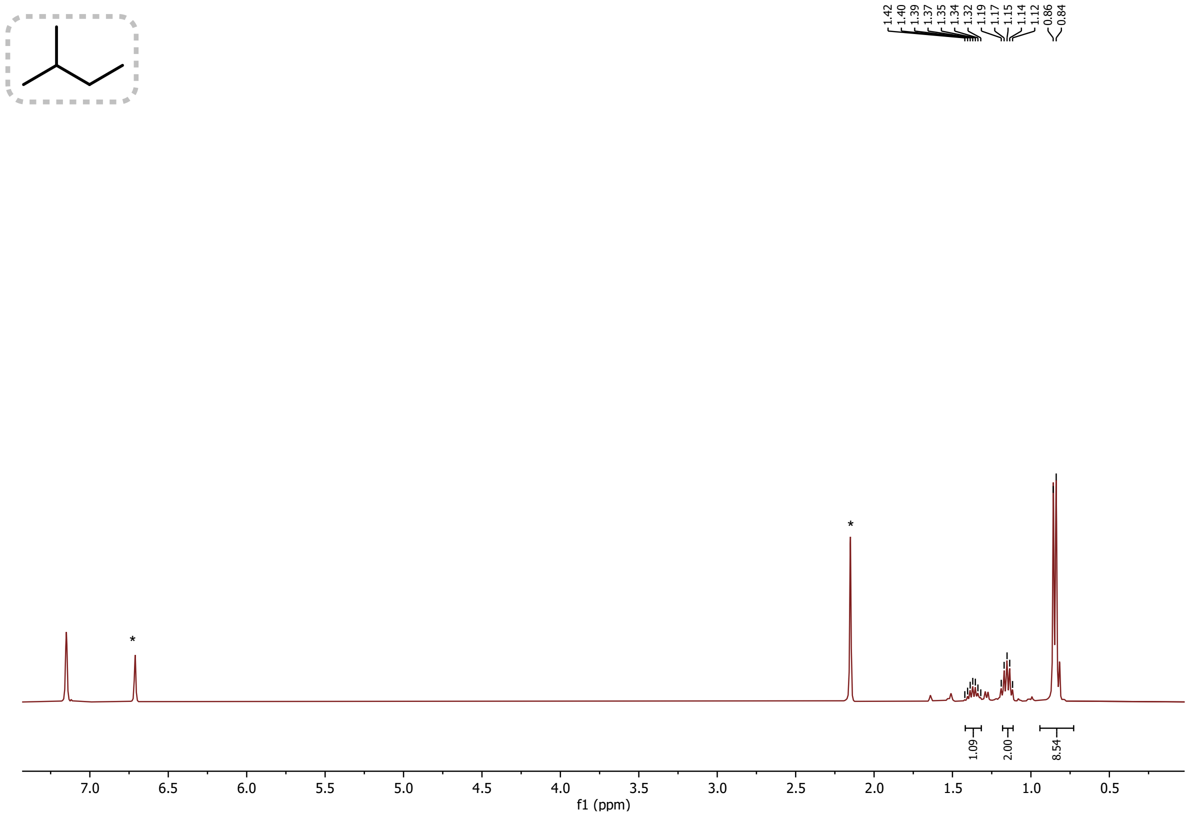

Figure S119. ^{1}H NMR spectrum (C_6D_6, 400 MHz, 298 K) of 2-methylbut-2-ene, after 20 h at 60°C with 1 mol% of **2** as catalyst forming 2-methyl-butane. *denotes the internal standard mesitylene.

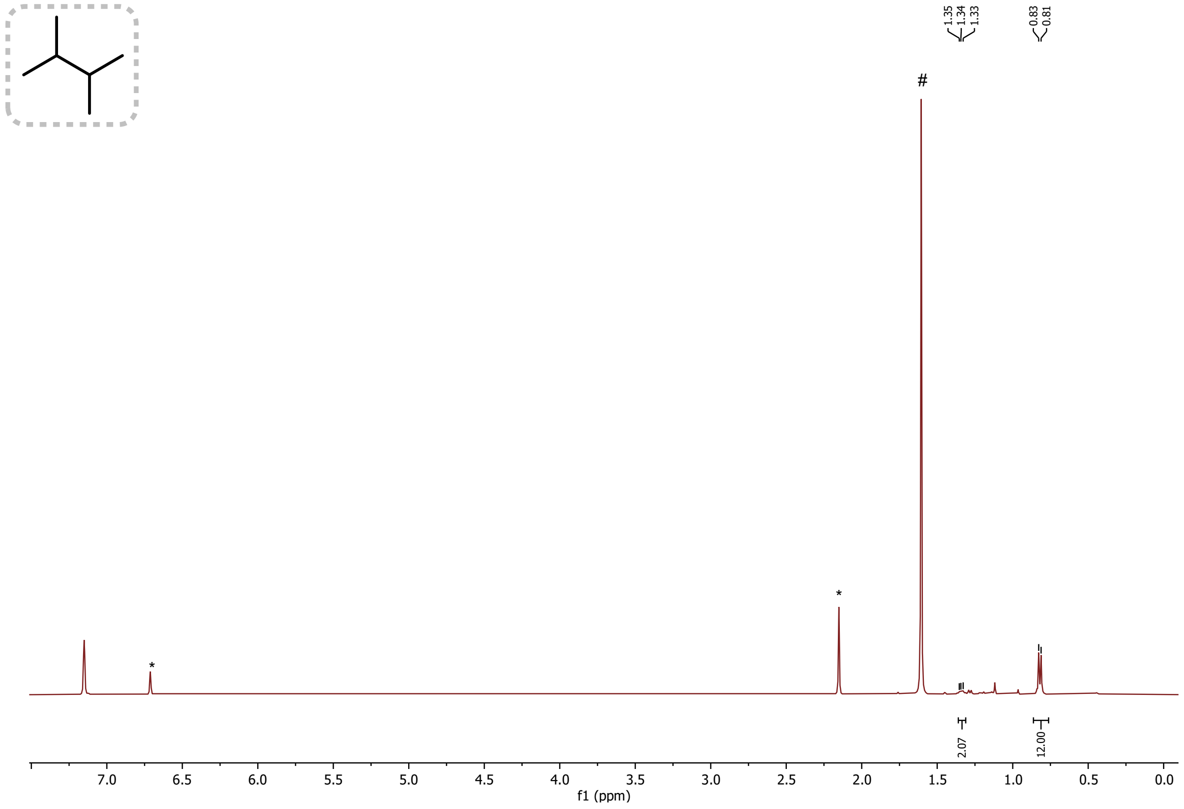

Figure S120. ^{1}H NMR spectrum (C_6D_6, 400 MHz, 298 K) of 2,3-dimethyl-but-2-ene, after 20 h at 60°C with 1 mol% of **2** as catalyst forming 2,3-dimethyl-butane. *denotes the internal standard mesitylene. #denotes 2,3-dimethyl-but-2-ene.

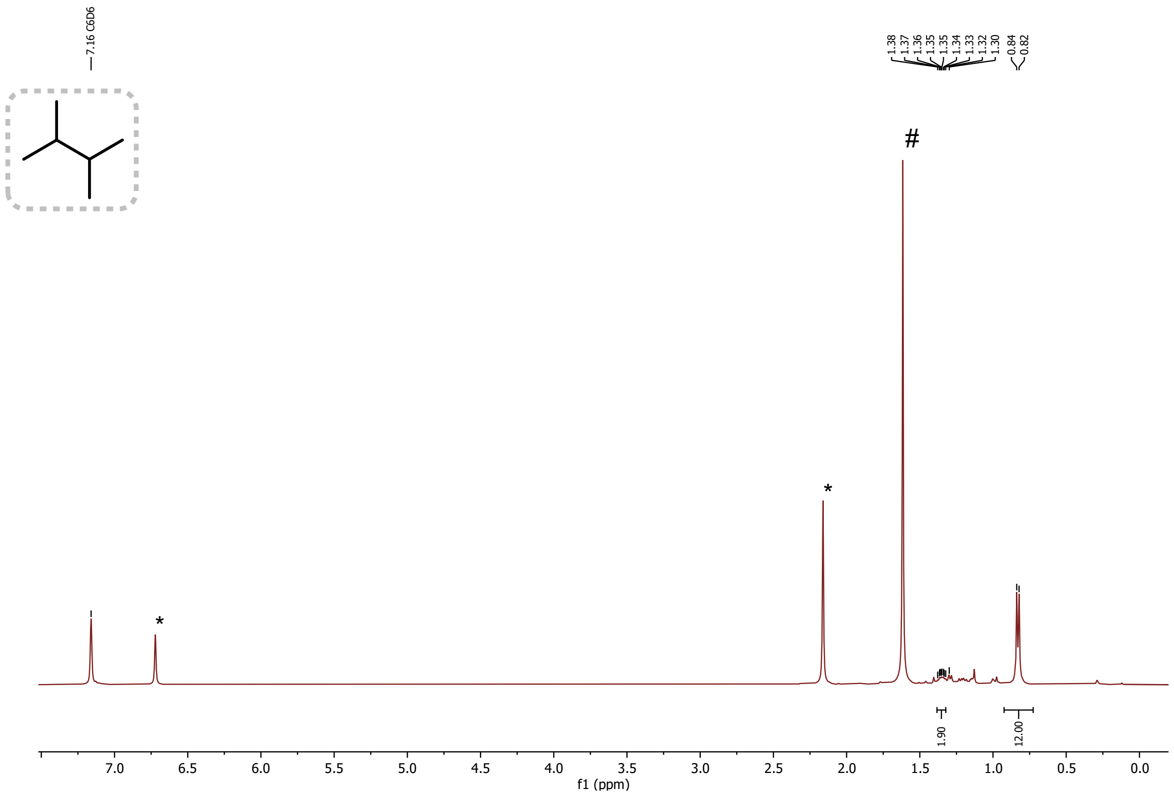

Figure S121. ^{1}H NMR spectrum (C_6D_6, 400 MHz, 298 K) of 2,3-dimethyl-but-2-ene, after 48 hours at 100°C with 1.5 mol% of **2** as catalyst forming 2,3-dimethyl-butane. *denotes the internal standard mesitylene. #denotes 2,3-dimethyl-but-2-ene.

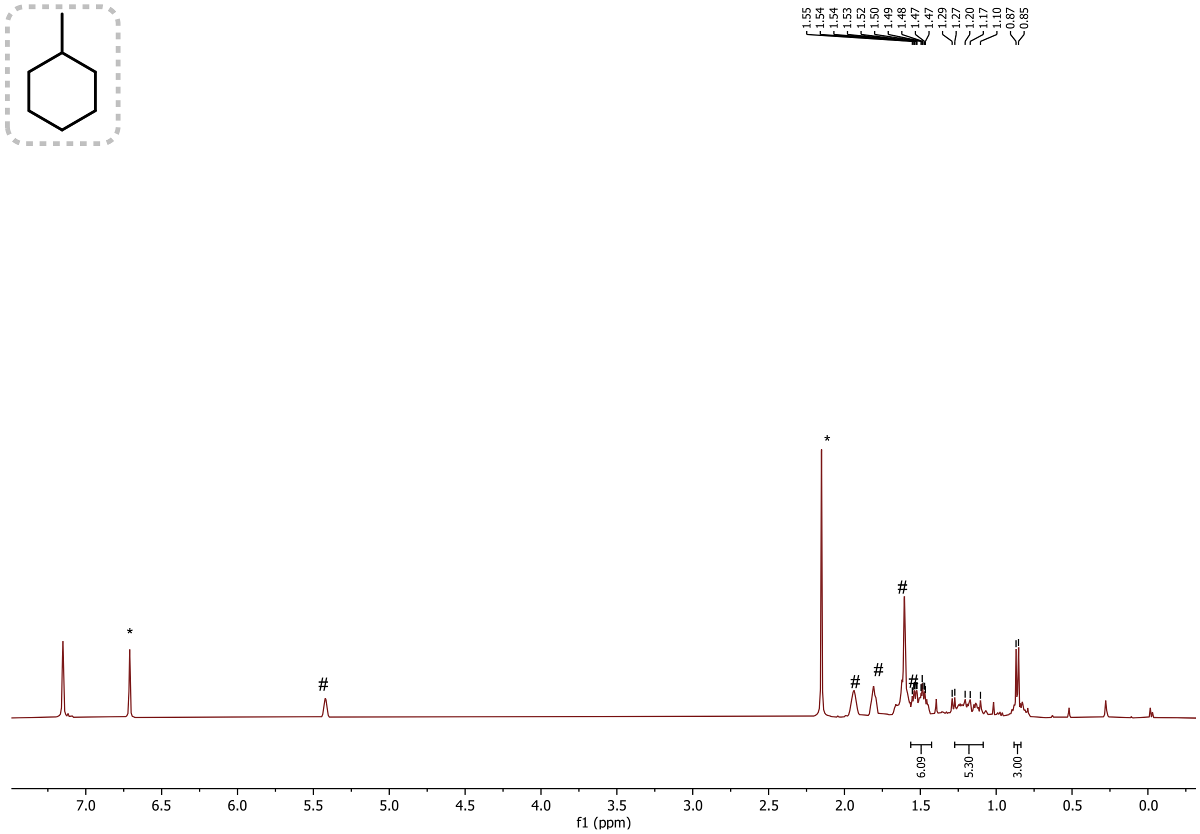

Figure S122. ^{1}H NMR spectrum (C_6D_6, 400 MHz, 298 K) of 1-methyl-cyclohex-1-ene, after 20 hours at 60°C with 1 mol% of **2** as catalyst forming methylcyclohexane. *denotes the internal standard mesitylene. #denotes 1-methyl-cyclohex-1-ene.

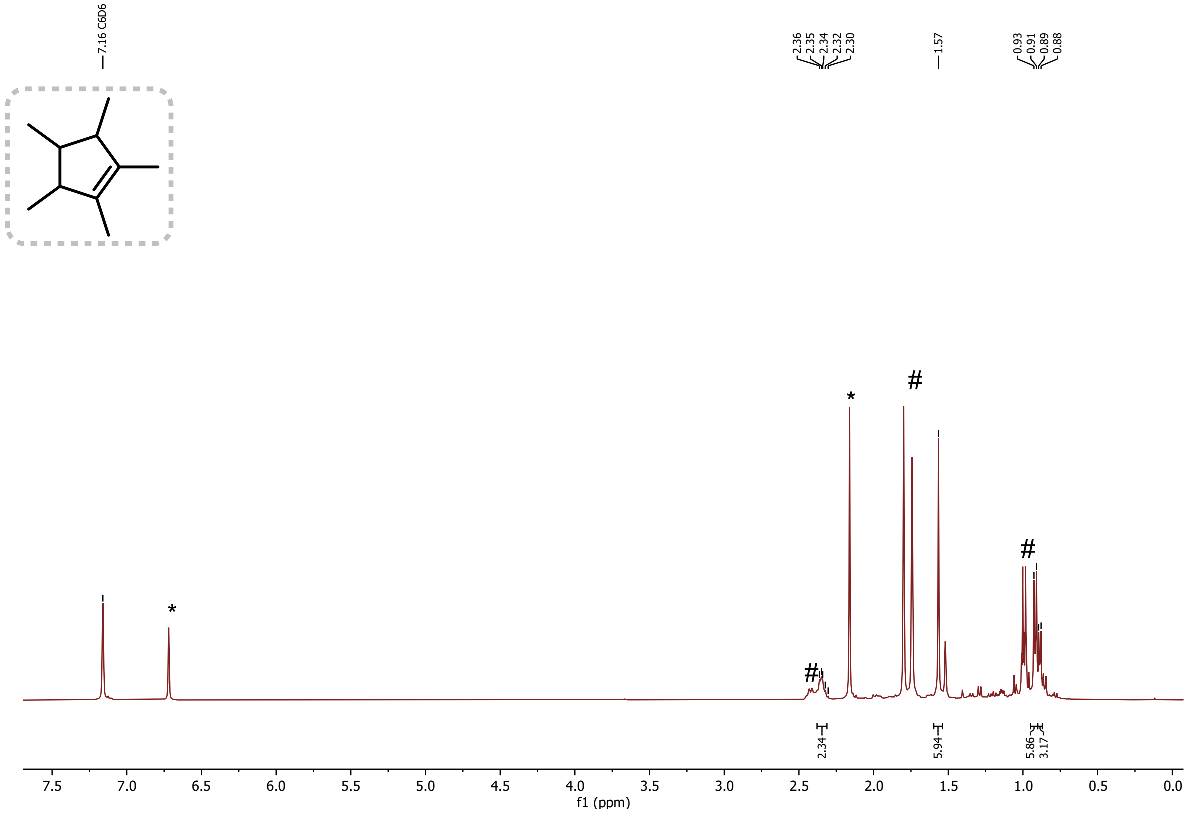

Figure S123. ^{1}H NMR spectrum (C_6D_6, 400 MHz, 298 K) of Cp*H, after 48 hours at 100°C with 1.5 mol% of **2** as catalyst forming 1,2,3,4,5-pentamethyl-cyclopent-1-ene. *denotes the internal standard mesitylene. #denotes Cp*H.

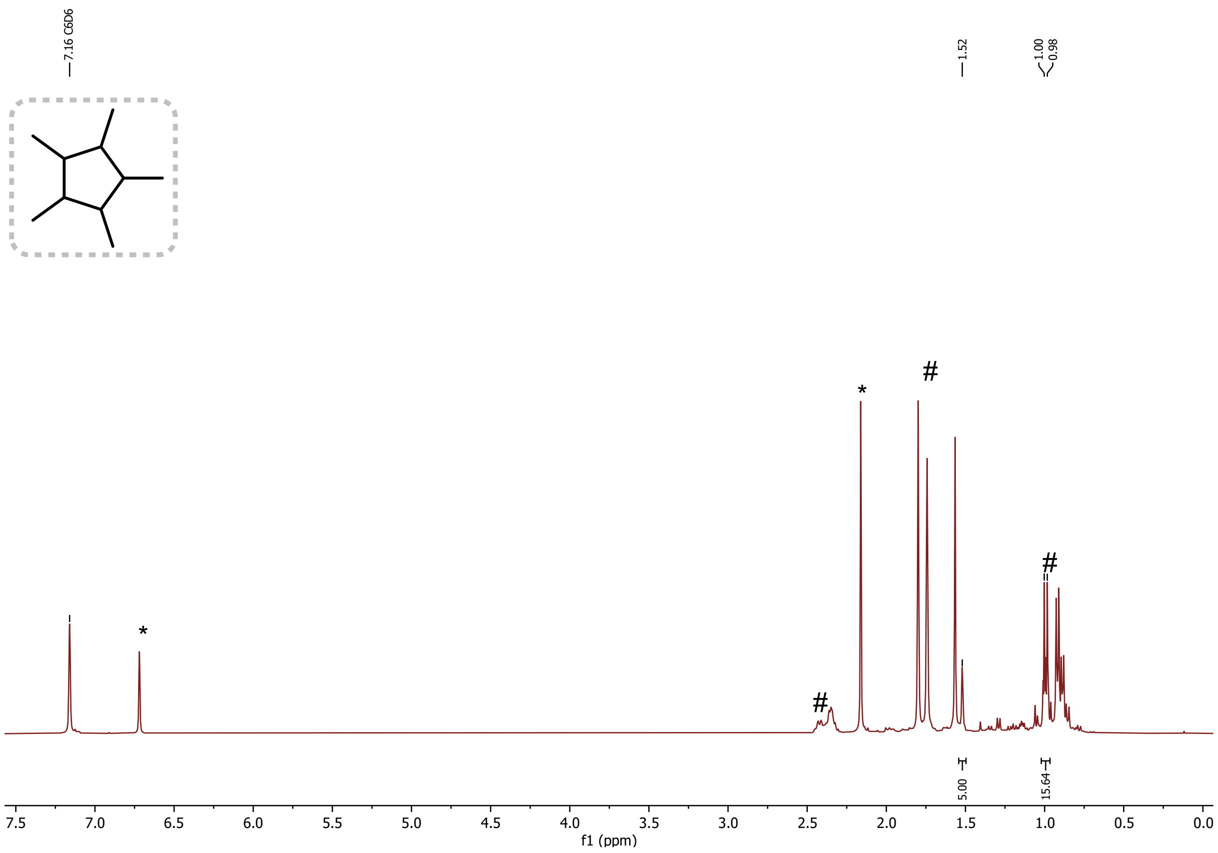

Figure S124. ^{1}H NMR spectrum (C_6D_6, 400 MHz, 298 K) of Cp*H, after 48 hours at 100°C with 1.5 mol% of **2** as catalyst forming 1,2,3,4,5-pentamethyl-cyclopentanne. *denotes the internal standard mesitylene. #denotes Cp*H.

17. X-ray crystallographic details

Single crystals of **2**, **3**, and **4** suitable for X-ray structural analysis were mounted in perfluoroalkyl ether oil on a nylon loop and positioned in a 150 K cold N_2 gas stream. Data collection was performed with a STOE StadiVari diffractometer (MoKα radiation) equipped with a DECTRIS PILATUS 300K detector. Structures were solved by Direct Methods (SHELXS-97)[23] and refined by full-matrix least-squares calculations against F^2 (SHELXL-2018).[24] The positions of the hydrogen atoms were calculated and refined using a riding model, aside from hydride ligands in **4** which were located and freely refined. All non-hydrogen atoms were treated with anisotropic displacement parameters. Crystal data, details of data collections, and refinements for all structures can be found in their CIF files, which are available free of charge via www.ccdc.cam.ac.uk/data_request/cif, and are summarized in Table S5.

Table S9. Summary of X-ray crystallographic data for **2**, **3**, and **4**.

	**2**	**3**	**4**
empirical form.	$C_{62}H_{110}Ga_2N_2NiP_2Si_2$	$C_{64}H_{114}Ga_2N_2NiP_2Si_2 \cdot C_6H_6$	$C_{64}H_{116}Ga_2N_2NiP_2Si_2 \cdot C_6H_6$
formula wt	1199.78	1305.94	1307.96
crystal syst.	triclinic	monoclinic	monoclinic
space group	*P-1*	*C2/c*	*C2/c*
a (Å)	13.165(3)	29.429(5)	29.459(6)
b (Å)	15.369(3)	12.5318(14)	12.510(3)
c (Å)	19.145(4)	20.676(3)	20.714(4)
α (deg.)	111.20(3)	90	90
β (δεγ)	96.49(3)	108.694(11)	108.97(3)
γ (deg.)	109.72(3)	90	90
vol (Å^3)	3275.4(14)	7223.1(17)	7219(3)
Z	2	4	4
ρ(calc) (g.cm^{-3})	1.217	1.201	1.203
μ (mm^{-1})	1.223	1.115	1.115
F(000)	1288	2808	2816
T (K)	150(2)	150(2)	150(2)
reflns collect.	35248	46045	31421
unique reflns	12850	7104	7070
R_{int}	0.0965	0.0392	0.0274
R1 [$I>2\sigma(I)$]	0.0725	0.0588	0.0507
*w*R2 (all data)	0.1317	0.0745	0.0592
CCDC No.	2473277	2473278	2473279

18. Computational Details.

All the geometry optimizations and frequency calculations reported in this paper were obtained with the ORCA 6.0.1 program.[25] Electron correlation was partially taken into account using the BP86[26,27] functional in conjunction with the D3(BJ) dispersion correction suggested by Grimme et al.,[28,29] the resolution-of-identity approach,[30] and the double-ζ quality plus polarization functions def2-SVP[31] basis set for all atoms. All species were characterized by frequency calculations: reactants and adducts exhibited positive definite Hessian matrices, while transition states showed a single negative eigenvalue in their diagonalized force constant matrices. Transition states were located by using the Nudged Elastic Band (NEB)[32] method implemented in ORCA. This level is denoted (RI)-BP86-D3(BJ)/def2-SVP.

The bonding situation in **2** was analyzed with the Energy Decomposition Analysis (EDA) method.[33,34] Within this approach, the interaction between the selected fragments ([NiGa···P] and [Ga····P] in this case), ΔE_{int} can be decomposed into the following physically meaningful terms:

$$\Delta E_{int} = \Delta E_{elstat} + \Delta E_{Pauli} + \Delta E_{orb} + \Delta E_{disp}$$

The term ΔE_{elstat} corresponds to the classical electrostatic interaction between the unperturbed charge distributions of the deformed reactants and is usually attractive. The Pauli repulsion ΔE_{Pauli} comprises the destabilizing interactions between occupied orbitals and is responsible for any steric repulsion. The orbital interaction ΔE_{orb} accounts for electron-pair bonding, charge transfer (interaction between occupied orbitals on one moiety with unoccupied orbitals on the other, including HOMO–LUMO interactions), and polarization (empty-occupied orbital mixing on one fragment due to the presence of another fragment). Finally, the ΔE_{disp} term takes into account the interactions which are due to dispersion forces. Moreover, the NOCV (Natural Orbital for Chemical Valence)[35] extension of the EDA method has been also used to further partition the ΔE_{orb} term. The EDA-NOCV approach provides pairwise energy contributions for each pair of interacting orbitals to the total bond energy.

The program package AMS 2020.101[36] was used for the EDA-NOCV calculations at the same BP86-D3BJ level, in conjunction with a double-ζ-quality basis set using

uncontracted Slater-type orbitals (STOs) augmented by one set of polarization functions with a frozen-core approximation for the core electrons.[37] Auxiliary sets of s, p, d, f, and g STOs were used to fit the molecular densities and to represent the Coulomb and exchange potentials accurately in each SCF cycle.[38] Scalar relativistic effects were incorporated by applying the zeroth-order regular approximation (ZORA).[39,40,41] This level of theory is denoted ZORA-BP86-D3BJ/DZP//RI-BP86-D3BJ/def2-SVP.

The below Figure outlines key intermediates in the mechanism leading to C-H bond forming reductive elimination. This regenerates compound **2** and forms ethane. Whilst reasonable transition states (*i.e.* with barriers <35 kcal·mol^{-1}) could not be located for the formation of INT1/INT2 from **4’** (*N.B.* **4’** is a computationally accessible isomer of **4**, see Fig. 6), the final barrier for alkane elimination is in keeping with the experimentally observed value, obtained through an Eyring analysis.

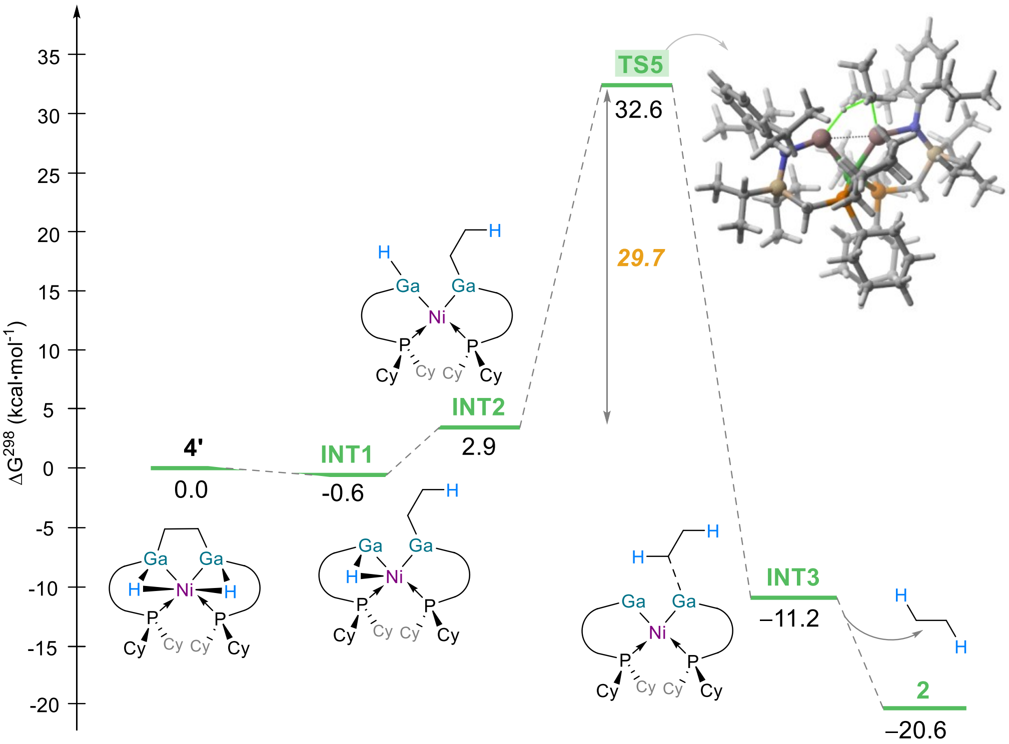

Figure S125. Calculated mechanism for the transformation of **4’** to **2**, in ethane reductive elimination. We note that energetically reasonable transition states for the transformation of **4’** to INT1/INT2 could not be found, and are as such not included.

Cartesian coordinates (in Å) and free energies (in a.u., at 298 K) of all the stationary points discussed in the text. All calculations have been performed at the (RI)-BP86-D3(BJ)/def2-SVP level:

2: G = -9155.40053663

Ga	1.260566000	0.757685000	-0.750050000
Ga	-1.261104000	0.756608000	0.750127000
Ni	0.000197000	-0.940273000	-0.000276000
P	-1.532411000	-1.506066000	-1.433164000
P	1.533230000	-1.505617000	1.432320000
Si	3.444280000	1.163184000	1.611957000
Si	-3.444662000	1.161716000	-1.612038000
N	2.942802000	1.430728000	-0.060203000
N	-2.943693000	1.428867000	0.060265000
C	-2.261438000	-0.115759000	-2.413714000
H	-2.772723000	-0.520059000	-3.314393000
H	-1.385810000	0.458619000	-2.778077000
C	-3.753342000	2.152243000	0.984495000
C	3.621131000	3.571069000	-1.116143000
C	4.731034000	-0.054401000	-1.854582000
H	4.212832000	-0.402630000	-0.939177000
C	-4.731611000	-0.057149000	1.853783000
H	-4.212845000	-0.405018000	0.938567000
C	-0.399368000	-3.971824000	-2.258585000
H	-1.263756000	-4.513526000	-1.824563000
H	0.313252000	-3.806044000	-1.423124000
C	3.061455000	-2.401655000	0.820802000
H	3.693572000	-1.559183000	0.460065000
C	-3.226184000	2.760027000	-2.656052000
H	-3.692333000	3.553268000	-2.029056000
C	3.751903000	2.154481000	-0.984666000
C	0.336776000	-1.887411000	-3.489727000
H	0.014921000	-0.907947000	-3.899065000
H	1.097328000	-1.654048000	-2.712795000
C	-0.838962000	-2.599627000	-2.797619000
H	-1.657733000	-2.744454000	-3.539475000
C	5.265958000	0.552110000	1.675843000
H	5.310103000	-0.240078000	0.894928000
C	-0.335913000	-1.888004000	3.488838000
H	-0.014435000	-0.908523000	3.898433000
H	-1.096477000	-1.654691000	2.711907000
C	5.505561000	2.202573000	-2.693983000
H	6.225227000	1.667892000	-3.332857000
C	3.225087000	2.760749000	2.657096000
H	3.691124000	3.554776000	2.031029000
C	2.261982000	-0.115406000	2.413316000
H	2.773757000	-0.519992000	3.313582000
H	1.386240000	0.458418000	2.778258000

C	-3.060170000	-2.402731000	-0.821629000
H	-3.692462000	-1.560615000	-0.460409000
C	3.919723000	-0.583593000	-3.052571000
H	2.874400000	-0.211469000	-3.012298000
H	3.885953000	-1.692979000	-3.062031000
H	4.356866000	-0.241203000	-4.013684000
C	-3.858872000	-3.162105000	-1.892258000
H	-4.078892000	-2.504843000	-2.757124000
H	-3.248486000	-4.004617000	-2.282728000
C	4.677699000	1.465832000	-1.827889000
C	3.860329000	-3.160939000	1.891402000
H	3.250353000	-4.003859000	2.281622000
H	4.079846000	-2.503738000	2.756443000
C	2.770514000	-3.286744000	-0.400322000
H	2.065026000	-4.098072000	-0.117853000
H	2.244954000	-2.675893000	-1.162511000
C	0.967882000	-2.748498000	-4.590535000
H	0.228127000	-2.905706000	-5.408431000
H	1.825848000	-2.208825000	-5.044245000
C	-3.623149000	3.568843000	1.116226000
C	-4.679289000	1.463118000	1.827087000
C	-2.768611000	-3.288006000	0.399257000
H	-2.062794000	-4.098960000	0.116537000
H	-2.243209000	-2.677053000	1.161467000
C	0.401170000	-3.971917000	2.257402000
H	-0.311442000	-3.806253000	1.421905000
H	1.265814000	-4.513201000	1.823381000
C	5.433316000	3.599123000	-2.759803000
H	6.101323000	4.160591000	-3.430641000
C	6.161353000	-0.613316000	-1.845275000
H	6.707083000	-0.374193000	-2.781417000
H	6.152003000	-1.718754000	-1.751780000
H	6.749989000	-0.203424000	-1.000532000
C	-1.734314000	3.096427000	-2.823863000
H	-1.268398000	2.469418000	-3.613255000
H	-1.151091000	2.923003000	-1.896522000
H	-1.579637000	4.156551000	-3.117696000
C	0.840167000	-2.599623000	2.796701000
H	1.658975000	-2.744187000	3.538553000
C	-4.480425000	4.266096000	1.986235000
H	-4.384211000	5.359579000	2.071508000
C	-0.966799000	-2.749568000	4.589393000
H	-1.824997000	-2.210311000	5.043154000
H	-0.227053000	-2.906693000	5.407311000
C	4.477718000	4.268762000	-1.986468000
H	4.381285000	5.362262000	-2.071286000
C	-2.480645000	4.304455000	0.434644000
H	-2.114412000	3.646512000	-0.372938000
C	0.254075000	-4.835545000	-3.350041000
H	0.598743000	-5.796957000	-2.913093000
H	-0.514716000	-5.095888000	-4.112627000

C	-5.159820000	-3.727436000	-1.302788000
H	-5.726507000	-4.277998000	-2.083118000
H	-5.809747000	-2.883812000	-0.977395000
C	1.416316000	-4.108700000	-4.040191000
H	1.841930000	-4.738714000	-4.849630000
H	2.235185000	-3.948243000	-3.304656000
C	-5.265879000	0.549480000	-1.676755000
H	-5.309612000	-0.243412000	-0.896529000
C	-5.508001000	2.199413000	2.692753000
H	-6.227642000	1.664393000	3.331353000
C	-3.920267000	-0.585727000	3.052063000
H	-2.875170000	-0.212942000	3.012070000
H	-3.885825000	-1.695089000	3.061575000
H	-4.357947000	-0.243595000	4.013016000
C	6.256792000	1.663254000	1.274287000
H	6.251070000	2.494429000	2.010283000
H	6.028749000	2.096889000	0.282598000
H	7.297415000	1.271868000	1.239602000
C	2.478928000	4.306081000	-0.433488000
H	2.114019000	3.648073000	0.374651000
C	-5.435949000	3.595958000	2.759239000
H	-6.104771000	4.157087000	3.429548000
C	-6.161472000	-0.617193000	1.843974000
H	-6.707814000	-0.378271000	2.779801000
H	-6.151216000	-1.722641000	1.750780000
H	-6.750083000	-0.207990000	0.998868000
C	-4.053914000	-3.899968000	0.970556000
H	-3.812156000	-4.577636000	1.816466000
H	-4.678850000	-3.083726000	1.392369000
C	-4.865515000	-4.640187000	-0.102667000
H	-5.810023000	-5.033159000	0.329233000
H	-4.288191000	-5.525485000	-0.454326000
C	-1.414672000	-4.109822000	4.038734000
H	-1.840159000	-4.740129000	4.848013000
H	-2.233502000	-3.949564000	3.303111000
C	3.934608000	2.759574000	4.021734000
H	3.719352000	3.695780000	4.582467000
H	5.035643000	2.682919000	3.926640000
H	3.595300000	1.916970000	4.661939000
C	4.056169000	-3.898006000	-0.971584000
H	3.814820000	-4.575653000	-1.817629000
H	4.680780000	-3.081402000	-1.393212000
C	-6.257708000	1.659477000	-1.274526000
H	-6.252626000	2.491206000	-2.009894000
H	-6.030189000	2.092607000	-0.282505000
H	-7.298001000	1.267194000	-1.240263000
C	-1.322963000	4.460764000	1.438508000
H	-1.627692000	5.070500000	2.314342000
H	-0.438823000	4.940370000	0.969761000
H	-1.002114000	3.469170000	1.821605000
C	1.319908000	4.461266000	-1.435916000

H	1.622881000	5.071023000	-2.312353000
H	0.435871000	4.940080000	-0.966159000
H	0.999508000	3.469269000	-1.818368000
C	2.871580000	5.652950000	0.187759000
H	3.162232000	6.399235000	-0.580981000
H	3.724316000	5.545339000	0.888810000
H	2.017729000	6.082505000	0.751401000
C	-5.690457000	-0.095414000	-3.007836000
H	-6.699730000	-0.553376000	-2.918224000
H	-4.999812000	-0.895278000	-3.340630000
H	-5.744832000	0.648696000	-3.826778000
C	-3.936054000	2.760021000	-4.020490000
H	-3.721325000	3.696889000	-4.580322000
H	-5.037031000	2.682814000	-3.925230000
H	-3.596578000	1.918206000	-4.661645000
C	1.733061000	3.096541000	2.824894000
H	1.267116000	2.468504000	3.613453000
H	1.150049000	2.924090000	1.897233000
H	1.578087000	4.156273000	3.119985000
C	-2.873356000	5.651002000	-0.187272000
H	-3.725162000	5.542903000	-0.889375000
H	-2.019058000	6.081014000	-0.749886000
H	-3.165346000	6.397134000	0.581107000
C	5.161670000	-3.725431000	1.301972000
H	5.728569000	-4.275859000	2.082242000
H	5.811165000	-2.881366000	0.976860000
C	-0.252048000	-4.836090000	3.348628000
H	-0.596234000	-5.797586000	2.911483000
H	0.516747000	-5.096236000	4.111278000
C	5.691360000	-0.093560000	3.006266000
H	6.700759000	-0.551094000	2.915866000
H	5.001161000	-0.893917000	3.338791000
H	5.745890000	0.649972000	3.825733000
C	4.868005000	-4.638049000	0.101588000
H	5.812767000	-5.030419000	-0.330306000
H	4.291111000	-5.523734000	0.452978000

3 (from X-ray): G = -9233.91523248

Ga	8.605566000	2.265444000	14.335633000
Ni	9.993933000	4.106240000	14.660745000
P	8.586459000	5.304881000	13.494519000
Si	5.778345000	3.966462000	14.507246000
N	6.689731000	2.449532000	14.455177000
C	5.967856000	1.212255000	14.460232000
C	6.911509000	5.478638000	14.234025000
C	9.083767000	7.110348000	13.266954000
H	9.188846000	7.405092000	14.333467000
C	8.192945000	4.572499000	11.807998000

H	7.542821000	3.719681000	12.119222000
C	4.985379000	4.295984000	16.224282000
H	4.454813000	3.352778000	16.477402000
C	4.409469000	3.946820000	13.155113000
H	4.909197000	3.469434000	12.283945000
C	6.060726000	4.555384000	17.292468000
H	6.437605000	5.598717000	17.231639000
H	6.940326000	3.894865000	17.176641000
H	5.665888000	4.417549000	18.321508000
C	5.617513000	0.567209000	13.231752000
C	3.956342000	5.439650000	16.227026000
H	3.560536000	5.616810000	17.251335000
H	3.087847000	5.236341000	15.571026000
H	4.410819000	6.396392000	15.889460000
C	7.392969000	5.424479000	10.814825000
H	8.031294000	6.259870000	10.454740000
H	6.508978000	5.883718000	11.301305000
C	5.607447000	0.579970000	15.692254000
C	10.480211000	7.223838000	12.639000000
H	11.156592000	6.507652000	13.144536000
H	10.434315000	6.910947000	11.571773000
C	3.216367000	3.056692000	13.559495000
H	2.653601000	3.497772000	14.408197000
H	3.528491000	2.038574000	13.861083000
H	2.496017000	2.950639000	12.718552000
C	6.096819000	1.069626000	11.874788000
H	6.530766000	2.077748000	12.035961000
C	4.436572000	-1.187834000	14.468151000
H	3.826809000	-2.104071000	14.469809000
C	3.920476000	5.328458000	12.685586000
H	3.171211000	5.222685000	11.870627000
H	4.741506000	5.957763000	12.285600000
H	3.433839000	5.902220000	13.499864000
C	9.426818000	3.963575000	11.126230000
H	10.129381000	4.775889000	10.841206000
H	9.965191000	3.324427000	11.856276000
C	6.964393000	4.581388000	9.603542000
H	6.284651000	3.770612000	9.948495000
H	6.377241000	5.201807000	8.893832000
C	9.227493000	0.318306000	14.442112000
H	8.394749000	-0.362727000	14.715350000
H	9.567114000	0.027353000	13.425489000
C	4.851860000	-0.612690000	13.262696000
H	4.577285000	-1.095705000	12.312101000
C	8.105828000	8.117230000	12.637748000
H	7.106960000	8.052692000	13.116849000
H	7.958820000	7.888507000	11.563591000
C	11.029750000	8.653482000	12.731202000
H	11.204589000	8.902337000	13.803133000
H	12.020224000	8.715796000	12.233284000
C	6.124045000	1.081076000	17.033555000

H	6.577637000	2.071215000	16.848485000
C	9.041610000	3.152445000	9.882443000
H	8.470817000	2.257536000	10.208023000
H	9.955531000	2.771187000	9.379550000
C	10.051623000	9.672507000	12.129337000
H	9.968685000	9.492405000	11.033268000
H	10.442501000	10.705331000	12.246971000
C	8.657294000	9.549061000	12.762366000
H	7.953058000	10.269867000	12.295557000
H	8.717905000	9.826516000	13.839383000
C	4.835862000	-0.596243000	15.670770000
H	4.555593000	-1.067607000	16.625715000
C	8.185053000	3.966982000	8.902399000
H	8.802250000	4.787291000	8.469982000
H	7.865035000	3.333167000	8.048297000
C	7.215749000	0.155657000	11.340200000
H	8.094445000	0.161249000	12.012356000
H	7.546964000	0.474533000	10.330366000
H	6.862738000	-0.893253000	11.261361000
C	5.024167000	1.235642000	18.097271000
H	4.174724000	1.845068000	17.729484000
H	5.428711000	1.723882000	19.007875000
H	4.616078000	0.251461000	18.408463000
C	4.966857000	1.196493000	10.838027000
H	4.571729000	0.203491000	10.539652000
H	5.339811000	1.687579000	9.915094000
H	4.117173000	1.790520000	11.225978000
C	7.241763000	0.156276000	17.551865000
H	6.859878000	-0.871139000	17.726729000
H	7.657540000	0.532668000	18.508689000
H	8.070629000	0.082606000	16.824085000
C	10.407101000	0.327612000	15.418689000
H	11.177080000	-0.450104000	15.233437000
H	10.064493000	0.211884000	16.469316000
Ga	11.191757000	2.192711000	15.241188000
P	11.150323000	5.160318000	16.194865000
Si	13.871793000	3.903860000	15.009789000
N	13.100771000	2.336285000	15.195114000
C	13.826256000	1.124989000	15.353438000
C	13.005526000	5.196679000	16.110638000
C	10.731322000	6.979375000	16.485764000
H	10.794715000	7.364633000	15.442495000
C	10.895826000	4.272961000	17.838501000
H	11.511224000	3.354394000	17.677587000
C	13.748192000	4.566435000	13.211375000
H	14.539062000	4.027947000	12.640482000
C	15.697633000	3.772989000	15.571154000
H	15.642007000	3.137225000	16.484296000
C	12.395520000	4.221940000	12.579765000
H	11.531791000	4.451754000	13.266739000
H	12.310085000	3.149120000	12.334714000

H	12.218840000	4.796867000	11.647257000
C	14.164657000	0.656719000	16.656046000
C	14.051599000	6.072270000	13.110487000
H	14.048083000	6.411283000	12.051581000
H	15.039081000	6.336588000	13.539550000
H	13.289535000	6.681043000	13.639757000
C	11.415039000	4.956774000	19.111295000
H	10.821440000	5.877190000	19.301234000
H	12.470703000	5.279428000	18.990920000
C	14.199266000	0.355372000	14.209202000
C	9.272532000	7.144009000	16.942767000
H	8.611453000	6.495084000	16.330387000
H	9.173106000	6.783309000	17.989284000
C	16.576692000	3.034836000	14.546184000
H	16.645697000	3.596296000	13.590107000
H	16.186917000	2.023125000	14.318905000
H	17.613969000	2.911424000	14.926701000
C	13.736379000	1.420515000	17.900960000
H	13.396490000	2.421248000	17.560659000
C	15.195523000	-1.332069000	15.673053000
H	15.724559000	-2.289206000	15.797401000
C	16.322427000	5.121366000	15.970535000
H	17.348325000	4.980567000	16.375629000
H	15.732371000	5.654538000	16.743669000
H	16.414180000	5.801721000	15.097791000
C	9.439175000	3.821023000	18.021658000
H	8.794861000	4.712876000	18.154817000
H	9.093471000	3.328449000	17.085182000
C	11.281978000	4.024258000	20.326912000
H	11.948363000	3.146782000	20.178857000
H	11.640637000	4.538674000	21.243450000
C	14.846366000	-0.567087000	16.791141000
H	15.104272000	-0.932108000	17.797836000
C	11.668578000	7.864369000	17.325773000
H	12.718181000	7.763751000	16.982468000
H	11.652768000	7.543263000	18.387429000
C	8.821776000	8.610079000	16.878872000
H	8.810372000	8.939893000	15.815644000
H	7.778060000	8.707612000	17.245524000
C	13.875358000	0.866688000	12.811697000
H	13.920515000	1.971660000	12.876043000
C	9.285429000	2.892901000	19.231320000
H	9.829547000	1.945043000	19.028231000
H	8.217575000	2.620117000	19.363396000
C	9.766803000	9.517370000	17.680302000
H	9.682927000	9.263558000	18.761590000
H	9.459614000	10.580206000	17.584118000
C	11.226384000	9.336287000	17.236644000
H	11.900069000	9.975734000	17.845411000
H	11.334346000	9.681027000	16.182793000
C	14.870646000	-0.865705000	14.391769000

H	15.154626000	-1.462974000	13.512859000
C	9.834947000	3.541399000	20.511191000
H	9.195449000	4.413180000	20.779415000
H	9.769701000	2.833534000	21.364462000
C	12.532637000	0.715790000	18.553881000
H	11.683629000	0.642373000	17.845881000
H	12.186044000	1.259545000	19.455924000
H	12.798201000	-0.317251000	18.859529000
C	14.887444000	0.430157000	11.743024000
H	15.927422000	0.660240000	12.050934000
H	14.687339000	0.957177000	10.787605000
H	14.829904000	-0.657942000	11.531064000
C	14.874602000	1.630257000	18.911726000
H	15.233997000	0.669743000	19.335802000
H	14.530243000	2.255435000	19.761769000
H	15.744576000	2.135842000	18.445133000
C	12.440214000	0.504065000	12.386520000
H	12.277865000	-0.592750000	12.416894000
H	12.226438000	0.859855000	11.357298000
H	11.688478000	0.966576000	13.057302000
H	6.320264000	6.215234000	13.652471000
H	7.074201000	5.936840000	15.230305000
H	13.303548000	6.190907000	15.720704000
H	13.414330000	5.137268000	17.141632000

INT1’: G = -9233.86087037

Ga	1.271648000	-0.194427000	-1.216631000
Ga	-1.683583000	-0.184714000	1.207881000
Ni	-0.283931000	-1.742510000	0.437490000
P	-1.576803000	-1.623947000	-1.490071000
P	1.504274000	-1.473821000	1.752331000
Si	3.392615000	1.132059000	1.003850000
Si	-3.298001000	1.193334000	-1.066121000
N	2.953670000	0.723154000	-0.647572000
N	-3.307012000	0.662388000	0.624600000
C	-2.068638000	0.083951000	-2.037006000
H	-2.413217000	0.073140000	-3.089915000
H	-1.094426000	0.625060000	-2.061610000
C	-4.258650000	1.127232000	1.578725000
C	3.531803000	2.431110000	-2.380692000
C	4.960265000	-1.082091000	-1.745928000
H	4.482752000	-1.123069000	-0.748343000
C	-5.699053000	-0.917372000	1.053877000
H	-5.077618000	-0.899285000	0.137042000
C	0.391333000	-3.313515000	-2.683335000
H	0.101193000	-4.047172000	-1.905214000
H	1.172035000	-2.673038000	-2.217151000
C	3.075546000	-2.430915000	1.344917000
H	3.540183000	-1.747945000	0.599381000
C	-2.562668000	2.962251000	-1.183480000

H	-3.137999000	3.552174000	-0.434287000
C	3.763683000	1.177904000	-1.731239000
C	-0.454928000	-1.454682000	-4.193171000
H	-1.346706000	-0.871789000	-4.499152000
H	0.296762000	-0.720492000	-3.824892000
C	-0.807534000	-2.423061000	-3.050637000
H	-1.612660000	-3.092129000	-3.428628000
C	5.203249000	0.643237000	1.443538000
H	5.300671000	-0.401007000	1.072400000
C	0.090259000	-1.109971000	4.230335000
H	0.253761000	-0.034875000	4.016596000
H	-0.896603000	-1.360186000	3.784376000
C	5.705811000	0.844289000	-3.203318000
H	6.534635000	0.208923000	-3.550399000
C	3.142189000	3.010701000	1.345845000
H	3.589688000	3.491756000	0.446856000
C	2.142529000	0.232016000	2.123643000
H	2.558271000	0.248816000	3.154083000
H	1.233548000	0.867193000	2.148334000
C	-3.247631000	-2.517335000	-1.490885000
H	-3.753207000	-2.122128000	-0.582089000
C	4.120957000	-1.966288000	-2.686108000
H	3.063652000	-1.622687000	-2.689696000
H	4.132846000	-3.033740000	-2.384999000
H	4.492878000	-1.895318000	-3.729056000
C	-4.117793000	-2.171551000	-2.712083000
H	-4.228091000	-1.079872000	-2.811001000
H	-3.607813000	-2.521855000	-3.637871000
C	4.833308000	0.356832000	-2.216908000
C	4.067025000	-2.611245000	2.506309000
H	3.625376000	-3.280958000	3.274638000
H	4.264131000	-1.645836000	3.008511000
C	2.830752000	-3.765059000	0.618446000
H	2.303327000	-4.473437000	1.293838000
H	2.156888000	-3.600040000	-0.247261000
C	0.105471000	-2.207059000	-5.410311000
H	-0.689061000	-2.863307000	-5.835512000
H	0.370776000	-1.481062000	-6.207789000
C	-4.000427000	2.272245000	2.392767000
C	-5.487147000	0.417817000	1.750619000
C	-3.143194000	-4.050065000	-1.358634000
H	-2.603815000	-4.457685000	-2.242968000
H	-2.551861000	-4.340332000	-0.473052000
C	0.960442000	-3.457119000	3.880824000
H	-0.008029000	-3.799430000	3.466063000
H	1.743563000	-4.082650000	3.407524000
C	5.538829000	2.121385000	-3.754109000
H	6.242563000	2.501163000	-4.510422000
C	6.399865000	-1.590648000	-1.607188000
H	6.916233000	-1.663153000	-2.586815000
H	6.410603000	-2.606898000	-1.162056000

H	7.000782000	-0.924987000	-0.955787000
C	-1.089552000	2.946711000	-0.765358000
H	-0.455042000	2.510972000	-1.560054000
H	-0.902133000	2.335753000	0.142403000
H	-0.701645000	3.965788000	-0.558418000
C	1.181792000	-1.967296000	3.565093000
H	2.145578000	-1.682025000	4.045300000
C	-4.996934000	2.731836000	3.274289000
H	-4.795756000	3.621892000	3.890544000
C	0.043701000	-1.345519000	5.747941000
H	-0.775023000	-0.745414000	6.198472000
H	0.990725000	-0.973673000	6.201030000
C	4.438563000	2.886479000	-3.358030000
H	4.262720000	3.864711000	-3.832748000
C	-2.634673000	2.939981000	2.398429000
H	-2.107225000	2.579987000	1.498056000
C	0.972567000	-4.040303000	-3.903704000
H	1.867227000	-4.624076000	-3.599780000
H	0.228080000	-4.780479000	-4.278090000
C	-5.508847000	-2.816299000	-2.631524000
H	-6.101871000	-2.549217000	-3.531740000
H	-6.051659000	-2.385378000	-1.761609000
C	1.321152000	-3.063289000	-5.034073000
H	1.701057000	-3.613753000	-5.920613000
H	2.144731000	-2.395926000	-4.700141000
C	-5.072448000	1.187461000	-1.808498000
H	-5.507563000	0.209307000	-1.507454000
C	-6.454357000	0.914412000	2.640803000
H	-7.406300000	0.375299000	2.757605000
C	-5.145205000	-2.040929000	1.949853000
H	-4.085231000	-1.846178000	2.209986000
H	-5.200965000	-3.028619000	1.445472000
H	-5.716057000	-2.104530000	2.899137000
C	6.238832000	1.500142000	0.687766000
H	6.195084000	2.561658000	1.009753000
H	6.087675000	1.484086000	-0.407610000
H	7.272788000	1.144529000	0.892629000
C	2.262342000	3.235536000	-2.147066000
H	1.764777000	2.794687000	-1.262971000
C	-6.228255000	2.078298000	3.387779000
H	-7.001627000	2.459399000	4.071865000
C	-7.149018000	-1.191898000	0.638173000
H	-7.823644000	-1.287860000	1.513926000
H	-7.218283000	-2.145765000	0.075897000
H	-7.545348000	-0.382302000	-0.007656000
C	-4.533002000	-4.703381000	-1.271127000
H	-4.426004000	-5.804977000	-1.181648000
H	-5.027471000	-4.359971000	-0.334307000
C	-5.417023000	-4.338224000	-2.469494000
H	-6.428648000	-4.782987000	-2.358802000
H	-4.979657000	-4.779073000	-3.394186000

C	-0.128125000	-2.833495000	6.085794000
H	-0.109012000	-2.987666000	7.185443000
H	-1.131463000	-3.172484000	5.740498000
C	3.849358000	3.581933000	2.585982000
H	3.607152000	4.659932000	2.716439000
H	4.951882000	3.502685000	2.522995000
H	3.529355000	3.066273000	3.516839000
C	4.149953000	-4.410167000	0.166941000
H	3.945273000	-5.387250000	-0.319697000
H	4.616215000	-3.766648000	-0.607687000
C	-5.937420000	2.296617000	-1.174298000
H	-5.561830000	3.305026000	-1.447244000
H	-5.961487000	2.238816000	-0.069559000
H	-6.986588000	2.233492000	-1.537070000
C	-1.817821000	2.461566000	3.614720000
H	-2.284524000	2.785522000	4.568376000
H	-0.780920000	2.857199000	3.581263000
H	-1.755571000	1.352310000	3.639777000
C	1.324230000	3.047242000	-3.357504000
H	1.820127000	3.369490000	-4.296624000
H	0.392680000	3.638221000	-3.244736000
H	1.049534000	1.977579000	-3.472397000
C	2.501761000	4.727091000	-1.867488000
H	2.914905000	5.254176000	-2.752661000
H	3.209758000	4.877317000	-1.028026000
H	1.547342000	5.228100000	-1.603625000
C	-5.137865000	1.273643000	-3.345617000
H	-6.178317000	1.109688000	-3.700833000
H	-4.499891000	0.523547000	-3.855940000
H	-4.822780000	2.269035000	-3.714906000
C	-2.714074000	3.644755000	-2.553673000
H	-2.187789000	4.624359000	-2.568818000
H	-3.772232000	3.839637000	-2.815810000
H	-2.273732000	3.031356000	-3.368376000
C	1.648631000	3.363646000	1.384251000
H	1.199901000	3.081875000	2.360513000
H	1.074622000	2.837250000	0.599010000
H	1.475361000	4.452865000	1.249858000
C	-2.682070000	4.472253000	2.320240000
H	-3.283181000	4.813263000	1.453143000
H	-1.658061000	4.885035000	2.211109000
H	-3.123616000	4.924354000	3.232842000
C	5.387776000	-3.233490000	2.027703000
H	6.079091000	-3.359570000	2.887667000
H	5.889717000	-2.533228000	1.323302000
C	0.949174000	-3.688010000	5.401892000
H	0.790301000	-4.765049000	5.621237000
H	1.948386000	-3.425609000	5.818053000
C	5.528571000	0.633621000	2.950585000
H	6.479933000	0.092789000	3.147449000
H	4.744678000	0.151227000	3.569284000

H	5.654372000	1.660821000	3.344076000
C	5.141424000	-4.575604000	1.326138000
H	6.097127000	-5.006790000	0.959784000
H	4.731915000	-5.302625000	2.064410000
C	-0.523067000	-3.740322000	0.871773000
H	-0.686777000	-4.278253000	-0.074050000
H	0.317960000	-4.129916000	1.458619000
C	-1.584152000	-3.084252000	1.515968000
H	-2.598784000	-3.095463000	1.094682000
H	-1.553290000	-2.925383000	2.604238000

TS1: G = -9233.85491991 (i = -34 cm-1)

Ga	0.919308000	1.388209000	0.105334000
Ga	-1.505893000	1.095578000	0.362402000
Ni	0.094441000	-0.665483000	0.462984000
P	-1.060621000	-1.917024000	-0.935544000
P	1.810782000	-1.119890000	1.714821000
Si	3.435292000	1.739110000	1.919891000
Si	-2.905948000	0.405161000	-2.416658000
N	2.642897000	2.123285000	0.379412000
N	-2.985138000	1.226791000	-0.857967000
C	-1.543779000	-0.935707000	-2.427469000
H	-1.824800000	-1.627876000	-3.251234000
H	-0.615027000	-0.428847000	-2.757100000
C	-4.044291000	2.144904000	-0.571287000
C	3.437499000	4.219559000	-0.687514000
C	3.615049000	0.555275000	-1.888465000
H	3.445950000	0.159680000	-0.865141000
C	-5.252481000	0.364377000	0.876821000
H	-4.322947000	-0.180532000	0.613660000
C	-0.088123000	-4.601805000	-0.803963000
H	-0.950656000	-4.846316000	-0.150565000
H	0.705919000	-4.218061000	-0.130675000
C	3.430071000	-1.925322000	1.148682000
H	4.055293000	-1.044168000	0.876941000
C	-2.356775000	1.577451000	-3.836000000
H	-3.012604000	2.464921000	-3.724055000
C	3.317193000	2.797682000	-0.685207000
C	0.710305000	-3.197377000	-2.751386000
H	0.412694000	-2.440866000	-3.504546000
H	1.534447000	-2.746428000	-2.161232000
C	-0.459696000	-3.493340000	-1.798403000
H	-1.319253000	-3.842925000	-2.414234000
C	5.290687000	1.413438000	1.565543000
H	5.286802000	0.857625000	0.599240000
C	0.064812000	-1.609560000	3.891670000
H	0.377592000	-0.635231000	4.319501000
H	-0.756198000	-1.386918000	3.177212000
C	4.602482000	2.719838000	-2.763853000
H	5.045422000	2.141206000	-3.587468000

C	3.215250000	3.119881000	3.242815000
H	3.733180000	4.017901000	2.839968000
C	2.528192000	0.262116000	2.724983000
H	3.211681000	-0.149841000	3.500271000
H	1.673040000	0.699995000	3.279914000
C	-2.723130000	-2.443699000	-0.225291000
H	-3.303322000	-1.495982000	-0.286764000
C	2.309617000	0.345445000	-2.676417000
H	1.466169000	0.916283000	-2.227700000
H	2.005635000	-0.719333000	-2.691822000
H	2.414099000	0.708501000	-3.719784000
C	-3.482670000	-3.530319000	-1.000242000
H	-3.606553000	-3.239643000	-2.062149000
H	-2.892029000	-4.472296000	-0.997518000
C	3.881390000	2.047100000	-1.759808000
C	4.178078000	-2.708812000	2.242867000
H	3.559635000	-3.572293000	2.567455000
H	4.328201000	-2.079971000	3.145388000
C	3.263150000	-2.765954000	-0.119688000
H	2.559266000	-3.602996000	0.074564000
H	2.775297000	-2.144799000	-0.897330000
C	1.219519000	-4.465299000	-3.454034000
H	0.426451000	-4.847016000	-4.136570000
H	2.090540000	-4.217159000	-4.097464000
C	-4.015322000	3.503091000	-1.026039000
C	-5.185551000	1.715959000	0.180696000
C	-2.601564000	-2.799331000	1.263387000
H	-1.935332000	-3.681523000	1.379417000
H	-2.100151000	-1.956587000	1.784059000
C	0.794920000	-3.619142000	2.555649000
H	0.012259000	-3.435122000	1.793273000
H	1.634207000	-4.103769000	2.016870000
C	4.760579000	4.109453000	-2.740849000
H	5.334456000	4.616792000	-3.531169000
C	4.761082000	-0.237479000	-2.527242000
H	4.954146000	0.079583000	-3.572380000
H	4.508155000	-1.314781000	-2.563353000
H	5.705507000	-0.119994000	-1.957926000
C	-0.895839000	2.026008000	-3.649267000
H	-0.197677000	1.253852000	-4.033642000
H	-0.628964000	2.198127000	-2.583722000
H	-0.673103000	2.962719000	-4.203211000
C	1.220713000	-2.251285000	3.105851000
H	2.083623000	-2.381642000	3.796350000
C	-5.143371000	4.325360000	-0.845070000
H	-5.111586000	5.359710000	-1.220836000
C	-0.447530000	-2.532336000	5.006208000
H	-1.295237000	-2.047698000	5.535210000
H	0.356457000	-2.672183000	5.764571000
C	4.163986000	4.849455000	-1.713086000
H	4.262578000	5.944697000	-1.707451000

C	-2.771653000	4.145391000	-1.620212000
H	-2.050356000	3.335474000	-1.825840000
C	0.410366000	-5.870975000	-1.512085000
H	0.696909000	-6.637816000	-0.761061000
H	-0.423224000	-6.310052000	-2.105907000
C	-4.854026000	-3.813970000	-0.369064000
H	-5.370270000	-4.621085000	-0.930961000
H	-5.492360000	-2.909770000	-0.463652000
C	1.587958000	-5.564372000	-2.447444000
H	1.916792000	-6.483711000	-2.976662000
H	2.456831000	-5.225723000	-1.839782000
C	-4.612490000	-0.374240000	-2.824113000
H	-4.899275000	-0.856904000	-1.862932000
C	-6.286723000	2.577632000	0.341097000
H	-7.165818000	2.221468000	0.900608000
C	-5.286095000	0.562126000	2.405676000
H	-4.475406000	1.233970000	2.746965000
H	-5.186152000	-0.405964000	2.939873000
H	-6.245317000	1.020043000	2.724806000
C	6.051611000	2.733716000	1.332344000
H	6.089329000	3.348362000	2.256722000
H	5.587331000	3.348372000	0.537196000
H	7.102781000	2.534933000	1.030808000
C	2.722779000	5.042854000	0.368274000
H	2.709428000	4.432362000	1.284997000
C	-6.290619000	3.869743000	-0.190167000
H	-7.167620000	4.524009000	-0.071063000
C	-6.461779000	-0.475451000	0.429814000
H	-7.415932000	0.036443000	0.671152000
H	-6.477405000	-1.456282000	0.947531000
H	-6.452849000	-0.661682000	-0.661563000
C	-3.961740000	-3.111734000	1.897543000
H	-3.826419000	-3.420238000	2.956467000
H	-4.570319000	-2.183721000	1.922590000
C	-4.718193000	-4.192987000	1.113055000
H	-5.718161000	-4.370802000	1.562689000
H	-4.163283000	-5.155901000	1.191077000
C	-0.870132000	-3.901225000	4.454609000
H	-1.197767000	-4.569466000	5.278692000
H	-1.752322000	-3.765367000	3.792224000
C	3.878087000	2.769847000	4.586870000
H	3.709270000	3.573166000	5.336848000
H	4.974063000	2.636896000	4.490944000
H	3.465979000	1.833644000	5.019907000
C	4.602003000	-3.329593000	-0.613915000
H	4.440976000	-3.961562000	-1.513567000
H	5.250870000	-2.487258000	-0.937058000
C	-5.675823000	0.692716000	-3.152004000
H	-5.441398000	1.213651000	-4.104512000
H	-5.760881000	1.466072000	-2.365733000
H	-6.679427000	0.229328000	-3.276619000

C	-2.123784000	5.083866000	-0.585444000
H	-2.838501000	5.867285000	-0.257131000
H	-1.240228000	5.591703000	-1.019927000
H	-1.795742000	4.517168000	0.305000000
C	1.254360000	5.245532000	-0.039084000
H	1.181861000	5.807366000	-0.993457000
H	0.693758000	5.806750000	0.736435000
H	0.741462000	4.271567000	-0.181261000
C	3.406848000	6.371781000	0.710139000
H	3.357928000	7.097870000	-0.128206000
H	4.475954000	6.225631000	0.967613000
H	2.906798000	6.848363000	1.578108000
C	-4.603874000	-1.466999000	-3.907417000
H	-5.584422000	-1.989736000	-3.948094000
H	-3.828768000	-2.242058000	-3.739560000
H	-4.421791000	-1.043133000	-4.914885000
C	-2.567462000	1.023775000	-5.255269000
H	-2.179597000	1.732042000	-6.020482000
H	-3.636991000	0.850277000	-5.485069000
H	-2.033741000	0.061043000	-5.408946000
C	1.726688000	3.463707000	3.431054000
H	1.184018000	2.643932000	3.947893000
H	1.206145000	3.635670000	2.466281000
H	1.592459000	4.375868000	4.050495000
C	-3.044140000	4.915514000	-2.924989000
H	-3.619874000	4.314452000	-3.656923000
H	-2.089937000	5.219323000	-3.402585000
H	-3.622806000	5.843563000	-2.736071000
C	5.524460000	-3.246022000	1.732927000
H	6.031744000	-3.819645000	2.537447000
H	6.193122000	-2.390850000	1.486788000
C	0.266174000	-4.552392000	3.653321000
H	-0.073731000	-5.508950000	3.202206000
H	1.100371000	-4.811078000	4.344591000
C	6.005319000	0.544722000	2.613760000
H	7.053573000	0.336369000	2.307660000
H	5.508191000	-0.431456000	2.769630000
H	6.052480000	1.048051000	3.601085000
C	5.328835000	-4.116586000	0.484119000
H	6.305582000	-4.491422000	0.112268000
H	4.729080000	-5.015371000	0.754829000
C	-1.833966000	2.479787000	1.910733000
H	-2.850904000	2.888382000	1.777008000
H	-1.070095000	3.228595000	2.171424000
C	-1.696540000	1.119521000	2.453851000
H	-2.588103000	0.547933000	2.755111000
H	-0.797176000	0.869765000	3.031471000

INT1: G = -9233.86558014

Ga	1.095995000	1.110900000	-0.175556000

Ga	-1.515958000	1.340201000	0.066330000
Ni	-0.158546000	-0.516501000	0.726010000
P	-1.173624000	-1.791658000	-0.837436000
P	1.652768000	-1.161187000	1.857910000
Si	3.439850000	1.582087000	2.028607000
Si	-3.002804000	0.433755000	-2.548506000
N	2.725928000	1.912864000	0.441568000
N	-3.026686000	1.404387000	-1.079753000
C	-1.651472000	-0.894508000	-2.387757000
H	-1.891933000	-1.643837000	-3.173146000
H	-0.712993000	-0.403282000	-2.718008000
C	-4.077929000	2.301274000	-0.708652000
C	3.137936000	4.157724000	-0.532167000
C	4.282986000	0.657956000	-1.735650000
H	4.025840000	0.224149000	-0.748221000
C	-5.035163000	0.547791000	0.939614000
H	-4.174568000	-0.009269000	0.519548000
C	-0.197891000	-4.503050000	-0.741426000
H	-1.058137000	-4.737460000	-0.082176000
H	0.611059000	-4.156613000	-0.069658000
C	3.235014000	-1.901906000	1.133417000
H	3.820717000	-0.988847000	0.887173000
C	-2.427018000	1.432362000	-4.088320000
H	-3.102358000	2.312320000	-4.129139000
C	3.331370000	2.743307000	-0.554039000
C	0.573478000	-3.092922000	-2.713783000
H	0.246709000	-2.347040000	-3.464533000
H	1.430238000	-2.637015000	-2.181921000
C	-0.560106000	-3.371105000	-1.709863000
H	-1.443324000	-3.701577000	-2.302586000
C	5.291862000	1.132450000	1.764370000
H	5.282105000	0.467804000	0.871636000
C	0.104906000	-1.867527000	4.158177000
H	0.435410000	-0.920226000	4.630127000
H	-0.802185000	-1.620809000	3.569280000
C	4.634064000	3.000277000	-2.609547000
H	5.210667000	2.551704000	-3.432789000
C	3.308776000	3.048844000	3.270008000
H	3.700513000	3.931517000	2.718581000
C	2.431410000	0.186156000	2.863693000
H	3.082785000	-0.266766000	3.642487000
H	1.605828000	0.686007000	3.408447000
C	-2.819453000	-2.381271000	-0.132318000
H	-3.416875000	-1.443998000	-0.160816000
C	3.304333000	0.054749000	-2.760674000
H	2.249483000	0.222509000	-2.445849000
H	3.451850000	-1.039383000	-2.870018000
H	3.428011000	0.524223000	-3.758441000
C	-3.584430000	-3.454468000	-0.920285000
H	-3.728059000	-3.141626000	-1.972947000
H	-2.990389000	-4.393660000	-0.947602000

C	4.084097000	2.163363000	-1.620258000
C	4.083633000	-2.751389000	2.093532000
H	3.526702000	-3.673105000	2.366628000
H	4.274938000	-2.208814000	3.042060000
C	2.996923000	-2.634503000	-0.183777000
H	2.352972000	-3.521095000	-0.008443000
H	2.418476000	-1.968111000	-0.852868000
C	1.036003000	-4.372533000	-3.429276000
H	0.214655000	-4.739488000	-4.086205000
H	1.890251000	-4.139077000	-4.100105000
C	-4.164035000	3.617838000	-1.258129000
C	-5.073345000	1.894629000	0.234036000
C	-2.671502000	-2.776841000	1.346007000
H	-2.008415000	-3.665407000	1.424408000
H	-2.152880000	-1.956028000	1.884392000
C	0.746246000	-3.736343000	2.587514000
H	-0.114368000	-3.527723000	1.922169000
H	1.544716000	-4.150811000	1.939962000
C	4.468753000	4.388708000	-2.569734000
H	4.914520000	5.024838000	-3.349520000
C	5.741046000	0.288960000	-2.054605000
H	6.046255000	0.633540000	-3.063561000
H	5.884849000	-0.810154000	-2.034859000
H	6.438361000	0.740378000	-1.320604000
C	-0.983359000	1.943726000	-3.914478000
H	-0.245471000	1.132960000	-4.093063000
H	-0.778391000	2.336404000	-2.895598000
H	-0.749100000	2.755342000	-4.635984000
C	1.187141000	-2.402208000	3.205281000
H	2.115994000	-2.561115000	3.797627000
C	-5.253253000	4.444812000	-0.928254000
H	-5.310107000	5.451827000	-1.370058000
C	-0.258776000	-2.901226000	5.234310000
H	-1.044483000	-2.488456000	5.901734000
H	0.629752000	-3.096945000	5.876912000
C	3.718448000	4.955425000	-1.533167000
H	3.565037000	6.045217000	-1.505874000
C	-3.067191000	4.200684000	-2.130693000
H	-2.361009000	3.382400000	-2.352171000
C	0.255163000	-5.773559000	-1.476536000
H	0.541757000	-6.555397000	-0.741212000
H	-0.602712000	-6.186122000	-2.054864000
C	-4.944979000	-3.757945000	-0.273095000
H	-5.467561000	-4.552999000	-0.846196000
H	-5.588859000	-2.853853000	-0.336002000
C	1.414736000	-5.481443000	-2.437754000
H	1.714993000	-6.402928000	-2.980009000
H	2.303996000	-5.160657000	-1.850278000
C	-4.727270000	-0.354143000	-2.814568000
H	-4.953957000	-0.786982000	-1.813126000
C	-6.136866000	2.762400000	0.544496000

H	-6.900228000	2.431866000	1.265983000
C	-4.786151000	0.723707000	2.448909000
H	-3.860692000	1.300203000	2.636108000
H	-4.687658000	-0.258541000	2.955690000
H	-5.624236000	1.267359000	2.932492000
C	6.112499000	2.384775000	1.393469000
H	6.162121000	3.100841000	2.240382000
H	5.686529000	2.923820000	0.525202000
H	7.159778000	2.111439000	1.138247000
C	2.238427000	4.795101000	0.509342000
H	2.112098000	4.049260000	1.309120000
C	-6.249061000	4.026245000	-0.041285000
H	-7.095578000	4.685392000	0.204301000
C	-6.317161000	-0.263289000	0.683283000
H	-7.210253000	0.259637000	1.082548000
H	-6.271914000	-1.253645000	1.179624000
H	-6.486599000	-0.426359000	-0.399502000
C	-4.018760000	-3.108975000	1.996204000
H	-3.863188000	-3.442382000	3.044575000
H	-4.629814000	-2.183714000	2.052526000
C	-4.784970000	-4.172768000	1.197279000
H	-5.777320000	-4.364955000	1.657630000
H	-4.226388000	-5.135625000	1.242551000
C	-0.724538000	-4.218734000	4.600029000
H	-0.974479000	-4.966342000	5.382106000
H	-1.662026000	-4.033610000	4.029074000
C	4.179481000	2.866077000	4.526604000
H	4.019194000	3.702555000	5.241657000
H	5.261232000	2.840599000	4.292071000
H	3.933099000	1.928596000	5.070191000
C	4.306101000	-3.084767000	-0.839457000
H	4.092925000	-3.651468000	-1.771169000
H	4.882053000	-2.186799000	-1.146767000
C	-5.802142000	0.705275000	-3.127119000
H	-5.599611000	1.206734000	-4.097794000
H	-5.856692000	1.493436000	-2.352214000
H	-6.809668000	0.240906000	-3.206415000
C	-2.290306000	5.269806000	-1.343186000
H	-2.936242000	6.139714000	-1.101245000
H	-1.421256000	5.639896000	-1.924261000
H	-1.916909000	4.854454000	-0.387603000
C	0.840022000	5.039042000	-0.076801000
H	0.875444000	5.755346000	-0.923598000
H	0.151464000	5.446486000	0.691831000
H	0.397801000	4.094135000	-0.455666000
C	2.823540000	6.068740000	1.135528000
H	2.884634000	6.903959000	0.406833000
H	3.846281000	5.894082000	1.528006000
H	2.187123000	6.415043000	1.975785000
C	-4.784919000	-1.499850000	-3.841083000
H	-5.734192000	-2.069645000	-3.743212000

H	-3.953803000	-2.226663000	-3.734086000
H	-4.743520000	-1.117363000	-4.880382000
C	-2.573795000	0.675993000	-5.420006000
H	-2.171014000	1.275730000	-6.265642000
H	-3.630773000	0.445885000	-5.657182000
H	-2.017326000	-0.285986000	-5.414455000
C	1.852904000	3.336628000	3.677085000
H	1.480243000	2.577331000	4.396233000
H	1.151800000	3.328847000	2.818423000
H	1.759330000	4.325193000	4.175027000
C	-3.592829000	4.765199000	-3.460994000
H	-4.218238000	4.026536000	-4.002233000
H	-2.753046000	5.058700000	-4.124359000
H	-4.217354000	5.668856000	-3.301567000
C	5.410776000	-3.159335000	1.434358000
H	6.010816000	-3.776009000	2.136554000
H	6.010204000	-2.244399000	1.227974000
C	0.343111000	-4.772208000	3.646916000
H	-0.017653000	-5.696892000	3.147990000
H	1.242904000	-5.065567000	4.234398000
C	5.959084000	0.350298000	2.907900000
H	6.981898000	0.022836000	2.619612000
H	5.391484000	-0.558176000	3.188815000
H	6.063931000	0.964992000	3.823505000
C	5.164223000	-3.918099000	0.122018000
H	6.127834000	-4.193702000	-0.356008000
H	4.641024000	-4.875369000	0.347541000
C	-1.356679000	2.121780000	1.859551000
H	-2.194242000	2.595643000	2.411311000
H	-0.477548000	2.794647000	1.903634000
C	-0.978039000	0.661287000	2.250891000
H	-1.893128000	0.110203000	2.558084000
H	-0.283150000	0.629575000	3.115247000

TS2: G = -9233.85226585 (i = -98 cm-1)

Ga	0.663260000	1.634216000	0.695398000
Ga	-1.636599000	1.286392000	-0.063637000
Ni	0.117580000	-0.695057000	0.620640000
P	-1.083435000	-1.706150000	-0.895805000
P	1.933037000	-1.250090000	1.814914000
Si	3.471803000	1.683488000	2.103110000
Si	-2.972706000	0.293286000	-2.767332000
N	2.439380000	2.283283000	0.804164000
N	-3.098919000	1.240026000	-1.292475000
C	-1.538694000	-0.928912000	-2.520800000
H	-1.716115000	-1.737647000	-3.261380000
H	-0.616088000	-0.412474000	-2.851049000
C	-4.118031000	2.228241000	-1.118548000
C	2.986772000	4.452635000	-0.237586000

C	3.146744000	0.861346000	-1.684819000
H	2.976885000	0.433102000	-0.674623000
C	-5.526344000	0.620626000	0.334689000
H	-4.631263000	-0.002564000	0.136673000
C	-0.080575000	-4.352087000	-0.521218000
H	-0.915212000	-4.508830000	0.191748000
H	0.733862000	-3.878462000	0.058221000
C	3.521187000	-1.991669000	1.106941000
H	4.116632000	-1.083849000	0.864757000
C	-2.485085000	1.350845000	-4.300344000
H	-3.128999000	2.255286000	-4.217798000
C	2.911550000	3.028630000	-0.314917000
C	0.629464000	-3.205501000	-2.659368000
H	0.299144000	-2.543306000	-3.483255000
H	1.498259000	-2.704878000	-2.185817000
C	-0.488410000	-3.365373000	-1.615379000
H	-1.385718000	-3.759915000	-2.144050000
C	5.226413000	1.392054000	1.390879000
H	5.030839000	0.889748000	0.416540000
C	0.404850000	-1.823633000	4.167011000
H	0.767053000	-0.868662000	4.597195000
H	-0.506454000	-1.572961000	3.583344000
C	3.709452000	3.142101000	-2.619331000
H	3.986121000	2.636380000	-3.557481000
C	3.543543000	2.883446000	3.607152000
H	4.017510000	3.812030000	3.215576000
C	2.688124000	0.105328000	2.841131000
H	3.448998000	-0.355517000	3.508389000
H	1.885811000	0.468691000	3.513688000
C	-2.725060000	-2.187552000	-0.105485000
H	-3.318411000	-1.254670000	-0.239850000
C	1.899951000	0.527997000	-2.520167000
H	1.000198000	1.000677000	-2.078963000
H	1.724801000	-0.565912000	-2.553553000
H	2.003552000	0.899295000	-3.560689000
C	-3.502722000	-3.348086000	-0.740678000
H	-3.635920000	-3.181495000	-1.827712000
H	-2.922065000	-4.289639000	-0.629595000
C	3.277761000	2.369433000	-1.523961000
C	4.342218000	-2.848447000	2.085789000
H	3.747606000	-3.738426000	2.383880000
H	4.554253000	-2.288269000	3.021072000
C	3.282503000	-2.733073000	-0.209708000
H	2.614866000	-3.601332000	-0.029854000
H	2.727981000	-2.064878000	-0.898951000
C	1.066090000	-4.564711000	-3.230300000
H	0.215201000	-5.010550000	-3.794125000
H	1.886481000	-4.422091000	-3.965472000
C	-3.936939000	3.555172000	-1.625109000
C	-5.337690000	1.915422000	-0.443394000
C	-2.558808000	-2.400408000	1.409115000

H	-1.905903000	-3.281978000	1.590979000
H	-2.023005000	-1.527754000	1.842228000
C	0.938897000	-3.767941000	2.664870000
H	0.072901000	-3.529942000	2.015235000
H	1.695788000	-4.244532000	2.008775000
C	3.787907000	4.536351000	-2.545019000
H	4.128727000	5.120899000	-3.413161000
C	4.401609000	0.212107000	-2.285519000
H	4.620184000	0.602057000	-3.300437000
H	4.264380000	-0.882893000	-2.382403000
H	5.297459000	0.395052000	-1.657362000
C	-1.011876000	1.795005000	-4.245583000
H	-0.340237000	0.964282000	-4.546755000
H	-0.684130000	2.116789000	-3.236058000
H	-0.815328000	2.638049000	-4.941515000
C	1.456443000	-2.435393000	3.220845000
H	2.382019000	-2.611408000	3.812902000
C	-5.004096000	4.469324000	-1.578860000
H	-4.861168000	5.480415000	-1.989911000
C	0.013364000	-2.797548000	5.287609000
H	-0.742484000	-2.323443000	5.948712000
H	0.902770000	-3.006891000	5.924300000
C	3.424855000	5.182061000	-1.355356000
H	3.482648000	6.278975000	-1.294813000
C	-2.570792000	4.031254000	-2.091163000
H	-1.974189000	3.128875000	-2.313476000
C	0.385112000	-5.704961000	-1.078492000
H	0.726995000	-6.356773000	-0.246139000
H	-0.481521000	-6.225537000	-1.545926000
C	-4.867306000	-3.531050000	-0.058728000
H	-5.414890000	-4.379110000	-0.521712000
H	-5.484072000	-2.624163000	-0.237683000
C	1.496337000	-5.537147000	-2.123326000
H	1.776521000	-6.520952000	-2.555460000
H	2.410675000	-5.142968000	-1.627628000
C	-4.614753000	-0.643851000	-3.087448000
H	-4.884427000	-1.028271000	-2.077770000
C	-6.377191000	2.864594000	-0.418018000
H	-7.322626000	2.607970000	0.085446000
C	-5.566688000	0.933189000	1.843469000
H	-4.670814000	1.500265000	2.159950000
H	-5.616698000	0.000281000	2.443616000
H	-6.459449000	1.541221000	2.098628000
C	5.947423000	2.721604000	1.095941000
H	6.156786000	3.280399000	2.032815000
H	5.358292000	3.384069000	0.432491000
H	6.926058000	2.539396000	0.600874000
C	2.556969000	5.160995000	1.037164000
H	2.771005000	4.460316000	1.862333000
C	-6.233935000	4.125047000	-1.006465000
H	-7.065033000	4.846583000	-0.991046000

C	-6.777209000	-0.172319000	-0.078856000
H	-7.704001000	0.413682000	0.091451000
H	-6.866072000	-1.101358000	0.521224000
H	-6.753867000	-0.454908000	-1.148253000
C	-3.907965000	-2.615743000	2.105834000
H	-3.749283000	-2.819910000	3.186308000
H	-4.492892000	-1.673175000	2.048403000
C	-4.709829000	-3.751771000	1.453145000
H	-5.704932000	-3.849189000	1.936687000
H	-4.182277000	-4.717709000	1.626255000
C	-0.520584000	-4.114906000	4.710012000
H	-0.793767000	-4.820813000	5.522343000
H	-1.456727000	-3.907166000	4.143154000
C	4.418689000	2.370099000	4.764091000
H	4.410460000	3.082625000	5.617910000
H	5.476251000	2.232097000	4.464517000
H	4.053937000	1.396284000	5.155173000
C	4.589320000	-3.231630000	-0.838505000
H	4.371251000	-3.793495000	-1.771974000
H	5.207605000	-2.357348000	-1.137673000
C	-5.737100000	0.307945000	-3.547916000
H	-5.520754000	0.729704000	-4.551305000
H	-5.879181000	1.160340000	-2.856442000
H	-6.709096000	-0.227127000	-3.627455000
C	-1.860842000	4.751742000	-0.929855000
H	-2.406128000	5.672957000	-0.635437000
H	-0.824499000	5.027574000	-1.209588000
H	-1.801335000	4.097873000	-0.034485000
C	1.036800000	5.391240000	1.044135000
H	0.735896000	6.083063000	0.231065000
H	0.698457000	5.822680000	2.009123000
H	0.491036000	4.437371000	0.886808000
C	3.322998000	6.458557000	1.325598000
H	3.078900000	7.261900000	0.599239000
H	4.419960000	6.297643000	1.295177000
H	3.059602000	6.843024000	2.332359000
C	-4.506562000	-1.855873000	-4.030192000
H	-5.451372000	-2.442248000	-4.031223000
H	-3.691593000	-2.552175000	-3.744172000
H	-4.314901000	-1.548237000	-5.077247000
C	-2.772267000	0.692432000	-5.661761000
H	-2.436814000	1.347154000	-6.496028000
H	-3.849074000	0.489004000	-5.820229000
H	-2.229777000	-0.271023000	-5.773119000
C	2.132921000	3.243453000	4.105417000
H	1.655898000	2.379180000	4.614869000
H	1.456887000	3.546956000	3.280998000
H	2.163115000	4.073063000	4.844280000
C	-2.595874000	4.910267000	-3.349030000
H	-3.132641000	4.416271000	-4.184207000
H	-1.562117000	5.128787000	-3.686231000

H	-3.090795000	5.886291000	-3.163319000
C	5.655465000	-3.330136000	1.447626000
H	6.216661000	-3.961836000	2.168397000
H	6.299924000	-2.449013000	1.232273000
C	0.508700000	-4.748382000	3.764857000
H	0.101671000	-5.673323000	3.303411000
H	1.403270000	-5.059298000	4.351077000
C	6.120999000	0.464209000	2.229975000
H	7.056241000	0.213772000	1.683384000
H	5.626361000	-0.490282000	2.492951000
H	6.424983000	0.944027000	3.182062000
C	5.391037000	-4.095742000	0.143909000
H	6.347122000	-4.423994000	-0.315459000
H	4.817311000	-5.022963000	0.372329000
C	-1.891730000	1.480622000	1.959396000
H	-2.686932000	0.790665000	2.311036000
H	-2.158934000	2.511467000	2.269359000
C	-0.488447000	1.043889000	2.360037000
H	-0.452263000	-0.082639000	2.412156000
H	-0.100278000	1.400801000	3.336763000

3: G = -9233.89671546

Ga	0.661798000	0.935647000	1.109350000
Ga	-1.690850000	0.095241000	0.260455000
Ni	0.271216000	-1.164978000	0.153803000
P	-0.990215000	-2.574290000	-1.033432000
P	1.866214000	-1.705339000	1.614117000
Si	3.566448000	1.141823000	1.995229000
Si	-2.827844000	-0.368773000	-2.711473000
N	2.404797000	1.761425000	0.853656000
N	-2.944709000	0.443531000	-1.145786000
C	-1.525569000	-1.764682000	-2.620028000
H	-1.862596000	-2.548919000	-3.330917000
H	-0.599355000	-1.342649000	-3.055685000
C	-3.878230000	1.500409000	-0.915420000
C	2.457293000	3.884505000	-0.402087000
C	2.969604000	0.263360000	-1.621249000
H	3.098362000	-0.034798000	-0.562390000
C	-5.534959000	-0.107694000	0.251801000
H	-4.741146000	-0.819738000	-0.052941000
C	-0.112518000	-5.278296000	-0.695523000
H	-0.954951000	-5.405405000	0.014510000
H	0.733740000	-4.878556000	-0.103467000
C	3.417719000	-2.565701000	0.970063000
H	3.973914000	-1.704073000	0.533513000
C	-2.217578000	0.781155000	-4.132267000
H	-2.794824000	1.723010000	-3.998713000
C	2.518189000	2.457441000	-0.370861000
C	0.633148000	-4.126447000	-2.831714000

H	0.325806000	-3.426856000	-3.634321000
H	1.540250000	-3.688531000	-2.368099000
C	-0.476611000	-4.249599000	-1.772671000
H	-1.390498000	-4.604488000	-2.299713000
C	5.246428000	0.811333000	1.143412000
H	4.992808000	0.139757000	0.293874000
C	0.072565000	-2.050339000	3.750464000
H	0.406724000	-1.066638000	4.137592000
H	-0.719871000	-1.828238000	3.003488000
C	2.463974000	2.436286000	-2.818603000
H	2.504049000	1.877577000	-3.767352000
C	3.773939000	2.280920000	3.534730000
H	4.303838000	3.188113000	3.161299000
C	2.721619000	-0.426717000	2.694126000
H	3.444792000	-0.965550000	3.342128000
H	1.946189000	-0.053193000	3.398051000
C	-2.662471000	-3.001340000	-0.261526000
H	-3.196757000	-2.026973000	-0.349911000
C	1.861045000	-0.619185000	-2.191369000
H	0.925411000	-0.526200000	-1.568936000
H	2.167878000	-1.680341000	-2.220886000
H	1.582308000	-0.321980000	-3.221741000
C	-3.511105000	-4.047532000	-0.997774000
H	-3.584795000	-3.804675000	-2.076190000
H	-3.024341000	-5.044895000	-0.926626000
C	2.613517000	1.742087000	-1.604479000
C	4.336031000	-3.233411000	2.005476000
H	3.813442000	-4.104983000	2.458279000
H	4.574485000	-2.542432000	2.839850000
C	3.115342000	-3.535188000	-0.174042000
H	2.577965000	-4.421437000	0.225781000
H	2.410430000	-3.049820000	-0.874695000
C	0.992337000	-5.494952000	-3.431692000
H	0.114607000	-5.890867000	-3.991363000
H	1.810918000	-5.380801000	-4.173814000
C	-3.528036000	2.842875000	-1.268892000
C	-5.145689000	1.255715000	-0.305933000
C	-2.572503000	-3.326898000	1.234480000
H	-2.012497000	-4.276679000	1.376937000
H	-1.980781000	-2.540081000	1.745922000
C	0.789949000	-4.170634000	2.568558000
H	0.029453000	-4.051284000	1.771874000
H	1.643191000	-4.707369000	2.105901000
C	2.287097000	3.825194000	-2.843608000
H	2.156937000	4.352829000	-3.800638000
C	4.299961000	0.021105000	-2.355114000
H	4.209034000	0.232169000	-3.440818000
H	4.617322000	-1.037634000	-2.246325000
H	5.106245000	0.667673000	-1.955669000
C	-0.714506000	1.101053000	-4.012050000
H	-0.110246000	0.278238000	-4.448855000

H	-0.361306000	1.232757000	-2.969059000
H	-0.445359000	2.024687000	-4.566250000
C	1.225668000	-2.774831000	3.028291000
H	2.077132000	-2.880658000	3.738677000
C	-4.466825000	3.874380000	-1.099795000
H	-4.193221000	4.901070000	-1.386896000
C	-0.502123000	-2.886494000	4.902855000
H	-1.355435000	-2.348524000	5.367336000
H	0.271817000	-2.990018000	5.697334000
C	2.326410000	4.541150000	-1.638443000
H	2.245994000	5.638894000	-1.659250000
C	-2.114632000	3.164516000	-1.723461000
H	-1.705159000	2.236618000	-2.149183000
C	0.272501000	-6.639455000	-1.295044000
H	0.579061000	-7.336611000	-0.486272000
H	-0.623520000	-7.093494000	-1.776029000
C	-4.915404000	-4.137864000	-0.379960000
H	-5.520415000	-4.899108000	-0.916304000
H	-5.436304000	-3.165523000	-0.522975000
C	1.387156000	-6.500205000	-2.340471000
H	1.629186000	-7.487630000	-2.787187000
H	2.316181000	-6.146166000	-1.839436000
C	-4.550814000	-1.097278000	-3.157303000
H	-4.902767000	-1.520811000	-2.189293000
C	-6.051368000	2.322999000	-0.150238000
H	-7.032246000	2.126576000	0.310663000
C	-5.561897000	-0.056399000	1.791614000
H	-4.572178000	0.217405000	2.202597000
H	-5.860709000	-1.035788000	2.219477000
H	-6.293634000	0.697750000	2.148122000
C	5.803512000	2.120508000	0.552642000
H	6.036503000	2.855160000	1.352911000
H	5.088267000	2.603187000	-0.143056000
H	6.747060000	1.940523000	-0.007792000
C	2.539441000	4.675410000	0.895227000
H	2.994918000	3.990192000	1.635851000
C	-5.734325000	3.621940000	-0.560175000
H	-6.463015000	4.438035000	-0.439778000
C	-6.876497000	-0.631777000	-0.287525000
H	-7.721559000	0.019127000	0.018787000
H	-7.084010000	-1.645926000	0.113162000
H	-6.881720000	-0.690309000	-1.391963000
C	-3.963289000	-3.452358000	1.868301000
H	-3.870798000	-3.733321000	2.939274000
H	-4.449163000	-2.455268000	1.851120000
C	-4.844808000	-4.461801000	1.119666000
H	-5.864076000	-4.485664000	1.560212000
H	-4.423425000	-5.484700000	1.251297000
C	-0.935074000	-4.281948000	4.433811000
H	-1.302114000	-4.883864000	5.291669000
H	-1.791752000	-4.178374000	3.734303000

C	4.619639000	1.669513000	4.665956000
H	4.685722000	2.362562000	5.533348000
H	5.655566000	1.438367000	4.352051000
H	4.171542000	0.726126000	5.045838000
C	4.400304000	-4.002620000	-0.865079000
H	4.161770000	-4.713633000	-1.684671000
H	4.896036000	-3.127682000	-1.343467000
C	-5.538232000	0.011161000	-3.574349000
H	-5.257192000	0.452907000	-4.552407000
H	-5.584090000	0.838506000	-2.840285000
H	-6.567840000	-0.393117000	-3.691817000
C	-1.227056000	3.494848000	-0.515224000
H	-1.578521000	4.404332000	0.013953000
H	-0.174049000	3.649458000	-0.823394000
H	-1.251437000	2.673413000	0.230758000
C	1.149473000	5.054100000	1.433791000
H	0.614534000	5.719208000	0.724713000
H	1.229097000	5.581255000	2.407061000
H	0.525617000	4.152521000	1.583830000
C	3.450291000	5.907625000	0.781321000
H	3.018624000	6.686690000	0.118555000
H	4.446773000	5.637030000	0.377075000
H	3.596780000	6.373735000	1.777713000
C	-4.558156000	-2.242520000	-4.185270000
H	-5.576697000	-2.678654000	-4.280476000
H	-3.876044000	-3.073178000	-3.911251000
H	-4.258745000	-1.897289000	-5.194844000
C	-2.512737000	0.248384000	-5.546165000
H	-2.096798000	0.930846000	-6.319693000
H	-3.596573000	0.148507000	-5.747585000
H	-2.048566000	-0.747493000	-5.713907000
C	2.400730000	2.722159000	4.069792000
H	1.850657000	1.868222000	4.519780000
H	1.754941000	3.138302000	3.274116000
H	2.502699000	3.490899000	4.865989000
C	-2.009813000	4.261583000	-2.789140000
H	-2.656694000	4.046788000	-3.664325000
H	-0.961402000	4.344493000	-3.141337000
H	-2.300067000	5.257940000	-2.394262000
C	5.636783000	-3.710678000	1.333989000
H	6.291264000	-4.210052000	2.079438000
H	6.195532000	-2.819425000	0.971285000
C	0.214973000	-5.007199000	3.722150000
H	-0.122793000	-5.993573000	3.338721000
H	1.024116000	-5.218490000	4.457911000
C	6.298046000	0.090127000	2.001996000
H	7.153897000	-0.252923000	1.380586000
H	5.889235000	-0.799114000	2.523039000
H	6.717454000	0.761097000	2.778830000
C	5.358303000	-4.647650000	0.147966000
H	6.309882000	-4.940683000	-0.343610000

H	4.899912000	-5.589071000	0.527648000
C	-2.043188000	0.951041000	2.091789000
H	-2.380845000	0.144570000	2.774310000
H	-2.858147000	1.698948000	2.019379000
C	-0.695541000	1.551046000	2.519000000
H	-0.713556000	2.656998000	2.585154000
H	-0.338059000	1.174645000	3.500332000

3-H2: G = -9235.08207073

Ga	8.409916000	9.991782000	14.849381000
Ni	9.948998000	8.371259000	14.232954000
P	8.386499000	7.255112000	13.184484000
Si	5.568804000	8.409702000	14.496206000
N	6.494414000	9.893474000	14.676078000
C	5.884437000	11.183777000	14.706098000
C	6.709159000	6.986848000	13.911430000
H	6.901094000	6.364913000	14.810391000
H	6.111053000	6.345956000	13.231625000
C	8.037426000	8.036859000	11.509464000
H	7.870774000	9.094373000	11.809546000
C	4.870723000	7.781169000	16.175369000
H	4.265188000	8.627676000	16.560962000
C	4.110117000	8.702318000	13.276880000
H	4.562795000	9.291957000	12.448131000
C	8.884519000	5.447268000	12.892218000
H	8.773384000	5.051042000	13.928126000
C	10.359660000	5.240211000	12.509759000
H	11.002824000	5.858496000	13.169199000
H	10.537073000	5.610272000	11.477725000
C	5.796666000	11.947876000	13.500135000
C	5.420777000	11.763951000	15.925413000
C	3.017133000	9.579507000	13.921177000
H	2.229643000	9.838153000	13.180064000
H	3.421911000	10.529269000	14.321332000
H	2.512414000	9.048972000	14.756482000
C	5.998429000	7.499430000	17.182354000
H	5.623817000	7.485868000	18.228323000
H	6.817780000	8.244893000	17.129128000
H	6.455427000	6.507414000	16.993537000
C	9.018312000	11.857002000	15.454026000
H	8.230889000	12.611265000	15.251838000
H	9.140433000	11.794118000	16.558230000
C	9.270170000	8.015585000	10.588618000
H	10.157985000	8.390514000	11.136062000
H	9.492939000	6.966146000	10.294383000
C	3.492762000	7.435080000	12.657951000
H	2.993686000	6.802176000	13.418418000
H	4.238375000	6.796327000	12.141495000
H	2.721179000	7.704247000	11.903979000

C	6.343053000	11.406738000	12.183900000
H	6.307076000	10.299989000	12.254389000
C	9.839668000	2.872582000	11.746827000
H	10.109613000	1.802007000	11.866778000
H	9.996082000	3.116905000	10.671593000
C	10.758158000	3.759625000	12.599561000
H	11.818620000	3.631889000	12.295434000
H	10.698397000	3.434339000	13.663444000
C	4.875252000	13.060573000	15.914416000
H	4.521571000	13.500186000	16.860486000
C	3.942548000	6.560087000	16.058857000
H	4.444828000	5.705403000	15.555679000
H	3.018722000	6.783335000	15.490205000
H	3.627487000	6.200796000	17.063434000
C	5.523186000	11.029837000	17.252617000
H	5.938309000	10.032182000	17.027075000
C	8.362700000	3.100674000	12.099406000
H	8.175023000	2.757556000	13.142333000
H	7.706420000	2.488441000	11.445154000
C	6.779662000	7.566799000	10.761147000
H	6.870077000	6.493289000	10.496252000
H	5.893390000	7.659655000	11.415479000
C	7.983542000	4.587746000	11.988984000
H	8.106073000	4.912201000	10.932839000
H	6.912495000	4.725332000	12.242736000
C	5.248111000	13.241879000	13.539306000
H	5.185059000	13.830327000	12.612076000
C	4.782798000	13.803902000	14.734119000
H	4.355637000	14.818211000	14.743198000
C	9.033745000	8.844295000	9.317952000
H	8.905712000	9.912504000	9.603804000
H	9.929332000	8.800598000	8.662708000
C	7.821919000	11.795648000	12.002547000
H	7.962323000	12.891909000	12.094034000
H	8.208483000	11.475324000	11.013010000
H	8.466771000	11.318383000	12.770071000
C	6.552966000	8.381959000	9.478237000
H	5.658846000	7.999910000	8.941785000
H	6.322108000	9.431212000	9.760153000
C	5.511474000	11.832283000	10.965316000
H	4.442611000	11.564600000	11.089496000
H	5.887261000	11.337735000	10.046821000
H	5.567620000	12.924793000	10.781710000
C	7.784948000	8.366974000	8.563963000
H	7.608677000	8.993207000	7.663843000
H	7.956496000	7.329631000	8.195575000
C	4.145326000	10.845432000	17.913461000
H	3.725022000	11.815453000	18.252320000
H	4.218996000	10.185981000	18.803276000
H	3.412275000	10.398533000	17.212097000
C	6.501840000	11.724158000	18.216008000

H	7.510642000	11.807799000	17.769563000
H	6.588839000	11.155403000	19.165262000
H	6.162334000	12.750127000	18.469474000
H	11.024121000	7.789899000	13.281388000
C	10.356256000	12.133372000	14.771409000
H	11.036423000	12.823684000	15.310201000
H	10.211777000	12.552572000	13.753652000
Ga	11.188589000	10.307877000	14.538587000
P	11.001468000	7.420066000	15.905886000
Si	13.932090000	8.575010000	14.942654000
N	13.065472000	10.111346000	14.862339000
C	13.745241000	11.368097000	14.885622000
C	12.798659000	7.160882000	15.563546000
H	12.809528000	6.385837000	14.767025000
H	13.293741000	6.697229000	16.440653000
C	11.008169000	8.381375000	17.520926000
H	11.472915000	9.338861000	17.184175000
C	14.629787000	7.961672000	13.251794000
H	15.281759000	8.788280000	12.890734000
C	15.399865000	8.795358000	16.163536000
H	14.972859000	9.462182000	16.941074000
C	10.464039000	5.646814000	16.256765000
H	10.514474000	5.236187000	15.222396000
C	8.995894000	5.572883000	16.694690000
H	8.392429000	6.266583000	16.076398000
H	8.902624000	5.935903000	17.740199000
C	13.959221000	12.063387000	16.113786000
C	14.160299000	11.976987000	13.660716000
C	16.574562000	9.549049000	15.510447000
H	17.341990000	9.821985000	16.267601000
H	16.247354000	10.486821000	15.018720000
H	17.083852000	8.927039000	14.744598000
C	13.562456000	7.693319000	12.180190000
H	14.029714000	7.401669000	11.214280000
H	12.916955000	8.572051000	11.986592000
H	12.891016000	6.860334000	12.477400000
C	9.593426000	8.717926000	18.020539000
H	8.986874000	9.122973000	17.179970000
H	9.081175000	7.789409000	18.348276000
C	15.876932000	7.526896000	16.891982000
H	16.273226000	6.757421000	16.198317000
H	15.067947000	7.049506000	17.482825000
H	16.693753000	7.771155000	17.605917000
C	13.485628000	11.507123000	17.450818000
H	13.206476000	10.447985000	17.272826000
C	9.332238000	3.189573000	17.468053000
H	8.971665000	2.143176000	17.377434000
H	9.232012000	3.462928000	18.543131000
C	8.458688000	4.137084000	16.631568000
H	7.403775000	4.105958000	16.978685000
H	8.447364000	3.792901000	15.572140000

C	14.783792000	13.236342000	13.685805000
H	15.097143000	13.700411000	12.737646000
C	15.511591000	6.711305000	13.429442000
H	14.940095000	5.866155000	13.871025000
H	16.386828000	6.895072000	14.082028000
H	15.899852000	6.356350000	12.449622000
C	13.893499000	11.294257000	12.328392000
H	13.632644000	10.250359000	12.571581000
C	10.812445000	3.283978000	17.065476000
H	10.932506000	2.908557000	16.023552000
H	11.431702000	2.622972000	17.708168000
C	11.873879000	7.835867000	18.667891000
H	11.453745000	6.871844000	19.026919000
H	12.903909000	7.620336000	18.315973000
C	11.334785000	4.731212000	17.132725000
H	11.309523000	5.080421000	18.186953000
H	12.395852000	4.766456000	16.812701000
C	14.589665000	13.321448000	16.089958000
H	14.753763000	13.857358000	17.037808000
C	15.006708000	13.911326000	14.891640000
H	15.497433000	14.896508000	14.897574000
C	9.627793000	9.708569000	19.189227000
H	10.021798000	10.681898000	18.826273000
H	8.597033000	9.904334000	19.549014000
C	12.225081000	12.243453000	17.940463000
H	12.405810000	13.336178000	18.006477000
H	11.923667000	11.891447000	18.948282000
H	11.370457000	12.089724000	17.254510000
C	11.918574000	8.828305000	19.841182000
H	12.528177000	8.410562000	20.670128000
H	12.437146000	9.751562000	19.506456000
C	14.584764000	11.545113000	18.527218000
H	15.523021000	11.074263000	18.171722000
H	14.255252000	11.010127000	19.441953000
H	14.827279000	12.585141000	18.827564000
C	10.510423000	9.190644000	20.334902000
H	10.568444000	9.943193000	21.149651000
H	10.036141000	8.285719000	20.779148000
C	15.119662000	11.269431000	11.403978000
H	15.386351000	12.282235000	11.035829000
H	14.922906000	10.638125000	10.512705000
H	16.008921000	10.860315000	11.925419000
C	12.665538000	11.900166000	11.624479000
H	11.761501000	11.828188000	12.263854000
H	12.449515000	11.371201000	10.672454000
H	12.819675000	12.975475000	11.396242000
H	10.932957000	8.724763000	13.062398000

TS3: G = -9235.06984880 (i = -148 cm-1)

Ga	-1.334077000	1.160304000	0.291421000
Ni	0.032851000	-0.742436000	-0.040278000

P	-1.341162000	-1.375380000	-1.549312000
Si	-4.177418000	-0.299388000	-0.286189000
N	-3.235483000	1.170245000	-0.098602000
C	-3.849636000	2.448282000	0.047032000
C	-3.086631000	-1.676669000	-1.056305000
H	-3.012346000	-2.482367000	-0.296099000
H	-3.624392000	-2.119287000	-1.919552000
C	-1.422359000	-0.243617000	-3.048816000
H	-1.576453000	0.756964000	-2.583451000
C	-4.771030000	-1.015672000	1.395869000
H	-5.302278000	-0.171940000	1.888518000
C	-5.685753000	0.032077000	-1.422921000
H	-5.269824000	0.690325000	-2.214344000
C	-0.870394000	-3.117941000	-2.121241000
H	-1.245766000	-3.692881000	-1.240621000
C	0.644649000	-3.356155000	-2.172824000
H	1.098352000	-2.907281000	-1.266610000
H	1.082109000	-2.808237000	-3.034810000
C	-4.109079000	3.273441000	-1.090717000
C	-4.157834000	2.957598000	1.347574000
C	-6.780672000	0.830130000	-0.688295000
H	-7.583997000	1.146092000	-1.389646000
H	-6.380734000	1.745111000	-0.207857000
H	-7.265523000	0.220550000	0.103166000
C	-3.580128000	-1.424989000	2.276722000
H	-3.873411000	-1.546305000	3.341778000
H	-2.741021000	-0.699708000	2.234650000
H	-3.164963000	-2.397897000	1.944511000
C	-0.640670000	3.059991000	0.038710000
H	-1.419551000	3.735415000	-0.370162000
H	-0.342190000	3.485701000	1.018461000
C	-0.077770000	-0.213433000	-3.796433000
H	0.739250000	0.024914000	-3.085741000
H	0.145280000	-1.226448000	-4.197738000
C	-6.267623000	-1.203653000	-2.130326000
H	-6.683509000	-1.943789000	-1.416971000
H	-5.513194000	-1.732291000	-2.749474000
H	-7.094958000	-0.911016000	-2.813305000
C	-3.757734000	2.839691000	-2.507855000
H	-3.471974000	1.770356000	-2.450338000
C	0.266183000	-5.474243000	-3.512868000
H	0.462599000	-6.566222000	-3.562370000
H	0.702963000	-5.036591000	-4.439245000
C	0.961380000	-4.851702000	-2.292400000
H	2.059775000	-5.007643000	-2.345997000
H	0.614613000	-5.366446000	-1.366577000
C	-4.722336000	4.238425000	1.481285000
H	-4.950262000	4.620884000	2.488665000
C	-5.763407000	-2.183449000	1.263018000
H	-5.333775000	-3.022794000	0.673681000
H	-6.708611000	-1.882810000	0.769854000

H	-6.031310000	-2.597272000	2.260271000
C	-3.840061000	2.161915000	2.604823000
H	-3.573013000	1.142297000	2.275431000
C	-1.245999000	-5.203717000	-3.495151000
H	-1.704532000	-5.739222000	-2.633110000
H	-1.725679000	-5.613644000	-4.409114000
C	-2.604564000	-0.502579000	-4.002112000
H	-2.564421000	-1.543220000	-4.381004000
H	-3.561892000	-0.402191000	-3.452588000
C	-1.556275000	-3.700601000	-3.366431000
H	-1.185546000	-3.177200000	-4.274411000
H	-2.654415000	-3.543962000	-3.330301000
C	-4.677142000	4.547527000	-0.907422000
H	-4.870925000	5.176641000	-1.790170000
C	-4.991362000	5.037148000	0.364694000
H	-5.434388000	6.037421000	0.485152000
C	-0.101216000	0.786751000	-4.958110000
H	-0.228771000	1.814023000	-4.550520000
H	0.876254000	0.777354000	-5.483992000
C	-2.541281000	3.619887000	-3.041388000
H	-2.729127000	4.713215000	-3.014808000
H	-2.319471000	3.346093000	-4.093744000
H	-1.634999000	3.424817000	-2.438117000
C	-2.594489000	0.465501000	-5.196558000
H	-3.421749000	0.206733000	-5.890556000
H	-2.805002000	1.490274000	-4.828453000
C	-4.950278000	2.978993000	-3.472301000
H	-5.859102000	2.485395000	-3.075328000
H	-4.714644000	2.527005000	-4.457762000
H	-5.204168000	4.043851000	-3.653024000
C	-1.247249000	0.474433000	-5.930003000
H	-1.267436000	1.210411000	-6.761197000
H	-1.070012000	-0.521624000	-6.396674000
C	-5.043057000	2.044312000	3.554623000
H	-5.310679000	3.021378000	4.008464000
H	-4.818685000	1.344935000	4.386859000
H	-5.941384000	1.669592000	3.022896000
C	-2.608701000	2.735233000	3.329386000
H	-1.710300000	2.698423000	2.679968000
H	-2.388514000	2.162871000	4.253486000
H	-2.764861000	3.796820000	3.613286000
H	-0.607166000	-2.047978000	0.212612000
C	0.580491000	2.879594000	-0.878582000
H	1.350805000	3.675945000	-0.801687000
H	0.268331000	2.826522000	-1.945344000
Ga	1.393811000	1.081267000	-0.443720000
P	1.277606000	-1.467176000	1.577849000
Si	4.170259000	-0.357645000	0.378540000
N	3.236266000	1.097169000	0.049233000
C	3.837507000	2.385808000	-0.068805000
C	3.043104000	-1.727873000	1.100898000

H	2.977422000	-2.503884000	0.308967000
H	3.582015000	-2.202685000	1.946864000
C	1.326345000	-0.400909000	3.115532000
H	1.452048000	0.611933000	2.671605000
C	4.928076000	-1.147813000	-1.205057000
H	5.517654000	-0.332668000	-1.677722000
C	5.584690000	0.078570000	1.604404000
H	5.110012000	0.794788000	2.308286000
C	0.860451000	-3.248720000	2.071487000
H	1.172488000	-3.765353000	1.132096000
C	-0.630835000	-3.560745000	2.255739000
H	-1.207889000	-3.095955000	1.428822000
H	-1.004889000	-3.095329000	3.192308000
C	3.937777000	3.236622000	1.071830000
C	4.282023000	2.866683000	-1.338105000
C	6.727696000	0.832397000	0.895630000
H	7.477946000	1.198592000	1.630133000
H	6.357668000	1.713294000	0.334590000
H	7.268090000	0.178193000	0.179602000
C	3.862170000	-1.613463000	-2.208765000
H	4.320874000	-1.911595000	-3.176626000
H	3.096751000	-0.839993000	-2.419985000
H	3.313502000	-2.498004000	-1.824829000
C	-0.035521000	-0.428113000	3.828787000
H	-0.835519000	-0.188317000	3.096238000
H	-0.239440000	-1.457811000	4.195616000
C	6.115511000	-1.094197000	2.447001000
H	6.570956000	-1.889781000	1.823734000
H	5.319141000	-1.570292000	3.056037000
H	6.899070000	-0.747490000	3.155734000
C	3.434132000	2.793804000	2.439012000
H	3.336172000	1.690435000	2.400726000
C	-0.049378000	-5.742104000	3.409908000
H	-0.191836000	-6.843327000	3.396008000
H	-0.425335000	-5.390015000	4.397267000
C	-0.873849000	-5.075291000	2.298550000
H	-1.955812000	-5.284702000	2.435697000
H	-0.590234000	-5.511601000	1.313320000
C	4.809792000	4.166259000	-1.440080000
H	5.146301000	4.533014000	-2.422726000
C	5.893547000	-2.304337000	-0.888567000
H	5.392765000	-3.113931000	-0.314313000
H	6.770059000	-1.973430000	-0.298423000
H	6.281760000	-2.767183000	-1.822483000
C	4.173397000	2.011282000	-2.591425000
H	3.831344000	1.014269000	-2.260102000
C	1.440788000	-5.391993000	3.287168000
H	1.852788000	-5.852363000	2.360699000
H	2.013418000	-5.826537000	4.133757000
C	2.494075000	-0.639180000	4.087689000
H	2.473867000	-1.683804000	4.459573000

H	3.459412000	-0.510295000	3.559313000
C	1.664927000	-3.870197000	3.226902000
H	1.332230000	-3.417868000	4.186562000
H	2.747740000	-3.651543000	3.129123000
C	4.469695000	4.530209000	0.921471000
H	4.537600000	5.188589000	1.800914000
C	4.907111000	5.001590000	-0.321860000
H	5.318696000	6.017813000	-0.418335000
C	-0.072296000	0.537460000	5.019112000
H	0.011767000	1.580555000	4.642712000
H	-1.054993000	0.467789000	5.531332000
C	2.025628000	3.351803000	2.703255000
H	2.017034000	4.460528000	2.659580000
H	1.646939000	3.044318000	3.700165000
H	1.308726000	2.986659000	1.942871000
C	2.428969000	0.319583000	5.287286000
H	3.258644000	0.097157000	5.990993000
H	2.595428000	1.356660000	4.926411000
C	4.410153000	3.153393000	3.570518000
H	5.432376000	2.782292000	3.353562000
H	4.080690000	2.710444000	4.532108000
H	4.478779000	4.249758000	3.725407000
C	1.072775000	0.255457000	6.001347000
H	1.046650000	0.974025000	6.847551000
H	0.935448000	-0.757154000	6.445351000
C	5.530624000	1.834534000	-3.293295000
H	5.893923000	2.788286000	-3.729968000
H	5.452053000	1.098589000	-4.120376000
H	6.307522000	1.477594000	-2.586978000
C	3.108469000	2.550861000	-3.563257000
H	2.117509000	2.618832000	-3.073804000
H	3.010823000	1.887880000	-4.448197000
H	3.367912000	3.566852000	-3.927555000
H	1.236346000	-0.657135000	-1.076036000

TS3‘: G = -9235.06971714 (i = -144 cm-1)

Ga	-1.335198404	1.146847384	0.291147159
Ni	0.028713559	-0.751826961	-0.046189747
P	-1.342239703	-1.376404843	-1.558231176
Si	-4.179081262	-0.307482731	-0.293833633
N	-3.236653777	1.161126328	-0.098812518
C	-3.850279325	2.438533765	0.054681798
C	-3.086206616	-1.683604512	-1.064000499
H	-3.008453151	-2.488361725	-0.303138890
H	-3.623972008	-2.128106326	-1.926374196
C	-1.426623112	-0.235850201	-3.050723951
H	-1.582112745	0.761598326	-2.579013722
C	-4.779177758	-1.025246447	1.384992049
H	-5.307675339	-0.179804647	1.877667409
C	-5.683726707	0.027285109	-1.434682548

H	-5.264563379	0.685495435	-2.224491077
C	-0.865312715	-3.112985496	-2.142254376
H	-1.236579263	-3.695737454	-1.265122885
C	0.650876121	-3.343333497	-2.198627201
H	1.104353404	-2.901795757	-1.288731265
H	1.083867087	-2.784003169	-3.055398323
C	-4.111677267	3.269826227	-1.078307973
C	-4.155908568	2.941589049	1.358303383
C	-6.779898849	0.826346954	-0.702982047
H	-7.580413101	1.143999999	-1.406795925
H	-6.380476670	1.740299531	-0.220207748
H	-7.268375166	0.216753720	0.086219100
C	-3.591922758	-1.441479727	2.267357102
H	-3.886667285	-1.559743087	3.332339053
H	-2.747162437	-0.722657484	2.225048087
H	-3.184004399	-2.417800202	1.936174143
C	-0.641353736	3.047207190	0.046981033
H	-1.419844123	3.724951018	-0.358900149
H	-0.343241894	3.468599208	1.028809974
C	-0.082460433	-0.198386374	-3.799127448
H	0.734552740	0.037389157	-3.087599403
H	0.142485683	-1.208346833	-4.206884200
C	-6.265490367	-1.206879065	-2.144965335
H	-6.687720978	-1.945345195	-1.433622659
H	-5.509387369	-1.738219496	-2.759739600
H	-7.088185633	-0.911832050	-2.832496620
C	-3.761750458	2.844277723	-2.498256132
H	-3.473605745	1.775310508	-2.446748264
C	0.280185511	-5.451449909	-3.557731713
H	0.482464324	-6.541890958	-3.616620101
H	0.711466808	-5.003848354	-4.481929932
C	0.976075097	-4.835676058	-2.334312899
H	2.075260328	-4.983784207	-2.393932184
H	0.636742219	-5.361952348	-1.412370923
C	-4.721805291	4.221080436	1.499234493
H	-4.948158278	4.598371057	2.508916077
C	-5.776333012	-2.188477751	1.248270810
H	-5.349129842	-3.028513293	0.658179303
H	-6.719285540	-1.882831769	0.753927919
H	-6.047883458	-2.602926490	2.244278661
C	-3.834070378	2.141728154	2.611873085
H	-3.562839597	1.124500082	2.278295422
C	-1.233365944	-5.189249956	-3.532599298
H	-1.686116033	-5.733860750	-2.673210016
H	-1.714084654	-5.594397421	-4.448147898
C	-2.609072489	-0.490689635	-4.004815988
H	-2.568256718	-1.529075303	-4.389719697
H	-3.566069938	-0.394427376	-3.454000812
C	-1.550569709	-3.688820202	-3.391009691
H	-1.183512426	-3.156589793	-4.295322330
H	-2.649357360	-3.537349297	-3.351864234

C	-4.681049686	4.542239426	-0.887770352
H	-4.876724967	5.175576465	-1.767083019
C	-4.994106538	5.024992227	0.387243666
H	-5.438581612	6.023908111	0.513477708
C	-0.108337472	0.808541354	-4.954897135
H	-0.237257035	1.833216069	-4.541252798
H	0.868872735	0.803900523	-5.481310496
C	-2.547694284	3.629891744	-3.029449140
H	-2.738327937	4.722615971	-2.997867204
H	-2.326106993	3.361366298	-4.083249704
H	-1.640388872	3.434392726	-2.427781903
C	-2.601059936	0.484193582	-5.193773150
H	-3.428284722	0.228118566	-5.888810014
H	-2.812796378	1.506537520	-4.819775647
C	-4.956120273	2.986227075	-3.460058948
H	-5.863390980	2.489153159	-3.063970018
H	-4.721440582	2.539492741	-4.448118201
H	-5.212122194	4.051529774	-3.635110894
C	-1.254289560	0.499726903	-5.927935680
H	-1.276349750	1.240574159	-6.754754043
H	-1.075460285	-0.493226286	-6.400595387
C	-5.036300744	2.015338270	3.561688190
H	-5.307669009	2.989627526	4.019212592
H	-4.808718182	1.313722878	4.391155877
H	-5.933353289	1.638973479	3.028977204
C	-2.605733845	2.718277703	3.339118549
H	-1.706110257	2.687452935	2.691069184
H	-2.384393947	2.144366261	4.261958846
H	-2.767150233	3.778222441	3.626135044
H	-0.587477630	-2.068362287	0.208641470
C	0.580067125	2.870670117	-0.870768996
H	1.347773074	3.669607912	-0.794120201
H	0.267295567	2.817130768	-1.937380989
Ga	1.398029886	1.074275632	-0.438689278
P	1.273360021	-1.469661301	1.579393178
Si	4.170665247	-0.365932385	0.383226482
N	3.239673072	1.090862144	0.054207528
C	3.840604737	2.379289624	-0.068853822
C	3.037696848	-1.734902988	1.099606508
H	2.969092089	-2.508311839	0.305345139
H	3.575357799	-2.213963678	1.944006122
C	1.327379002	-0.395311127	3.111705485
H	1.454030783	0.615212780	2.662826581
C	4.932239795	-1.153967311	-1.199525468
H	5.520064696	-0.336751738	-1.670798266
C	5.581626275	0.066422246	1.614360658
H	5.104628074	0.780327950	2.319128137
C	0.853928768	-3.246943724	2.085778258
H	1.157137432	-3.770168647	1.147070578
C	-0.637938245	-3.550206471	2.281101659
H	-1.216703064	-3.089128831	1.453556777

H	-1.004016875	-3.074449063	3.215437028
C	3.942458836	3.233800597	1.068805495
C	4.281840341	2.856845544	-1.340626625
C	6.725709914	0.823376166	0.910550872
H	7.474001123	1.187796600	1.647935196
H	6.356495657	1.705630556	0.351139937
H	7.268299437	0.171724358	0.193868465
C	3.868581280	-1.621710215	-2.204776683
H	4.328337812	-1.912595621	-3.174307140
H	3.097522258	-0.852622026	-2.411464280
H	3.326148224	-2.511633140	-1.824378089
C	-0.032232470	-0.416530700	3.829383001
H	-0.834512667	-0.178657241	3.098580422
H	-0.236813585	-1.443990543	4.201799059
C	6.111912091	-1.108554589	2.454210520
H	6.571722316	-1.900209579	1.829195720
H	5.314717200	-1.589500968	3.058342822
H	6.892024591	-0.762847789	3.167216652
C	3.440890317	2.796034219	2.438244862
H	3.338956656	1.692978767	2.402972255
C	-0.059653331	-5.725725593	3.449731803
H	-0.208876347	-6.826190062	3.446764669
H	-0.424543607	-5.363668055	4.437666220
C	-0.889348610	-5.063066247	2.339600306
H	-1.971438114	-5.264173375	2.487773987
H	-0.617106705	-5.510488648	1.356207918
C	4.809415660	4.156148613	-1.447352290
H	5.143503448	4.520175644	-2.431840274
C	5.900459500	-2.308260820	-0.883236228
H	5.401602967	-3.119127483	-0.309105560
H	6.776257985	-1.975394589	-0.293143721
H	6.289694706	-2.769974615	-1.817285214
C	4.169083715	1.999336236	-2.592159862
H	3.826452685	1.003233150	-2.258735881
C	1.431371101	-5.385321741	3.311070367
H	1.832053073	-5.853526471	2.383517304
H	2.009401073	-5.817913578	4.154979349
C	2.497475603	-0.632434697	4.081385245
H	2.476833954	-1.676038436	4.455814703
H	3.461294943	-0.506370449	3.549823335
C	1.662670504	-3.865116426	3.239926091
H	1.337474212	-3.406302314	4.199020564
H	2.745883033	-3.652038532	3.134649089
C	4.473801417	4.527051958	0.913587049
H	4.542882015	5.188024617	1.790987162
C	4.909088371	4.994754733	-0.331830559
H	5.320252958	6.010773315	-0.432150939
C	-0.062891534	0.553846804	5.015923715
H	0.024485485	1.595163878	4.635529489
H	-1.044914068	0.490084931	5.530176494
C	2.035345995	3.360228266	2.705248292

H	2.031752592	4.468990068	2.661863849
H	1.657591750	3.054433379	3.703020173
H	1.314781948	2.998565863	1.946568684
C	2.437879961	0.329159192	5.278952398
H	3.268686904	0.105708078	5.980989346
H	2.606642943	1.364844909	4.915465107
C	4.421873255	3.154878787	3.565755955
H	5.441678277	2.778363843	3.346895594
H	4.093488108	2.716799789	4.529925049
H	4.495949621	4.251432560	3.716659018
C	1.083307823	0.270954278	5.996501577
H	1.061442440	0.992022897	6.840679488
H	0.943879706	-0.739974735	6.443669668
C	5.524209212	1.819822044	-3.297375645
H	5.887278759	2.772428844	-3.736730702
H	5.442617401	1.082477467	-4.122915845
H	6.302730383	1.463369499	-2.592620419
C	3.102524092	2.539047016	-3.562207611
H	2.112982836	2.609665381	-3.070357437
H	3.001184015	1.874679205	-4.445668636
H	3.362790027	3.553912652	-3.929018243
H	1.236859161	-0.655365022	-1.079862985

4‘: G = -9235.08608688

Ga	8.076359000	10.224403000	14.993413000
Ni	9.549667000	8.505940000	15.135170000
P	8.487699000	7.529441000	13.512121000
Si	5.442006000	8.523398000	14.406956000
N	6.229335000	10.093158000	14.545930000
C	5.546775000	11.346094000	14.577534000
C	6.727934000	7.180462000	13.953975000
H	6.812400000	6.522883000	14.844806000
H	6.272034000	6.553768000	13.161097000
C	8.367248000	8.476142000	11.893564000
H	7.912348000	9.429611000	12.253327000
C	4.641743000	7.918500000	16.050574000
H	4.015512000	8.774580000	16.389013000
C	4.106746000	8.616891000	13.029456000
H	4.602130000	9.212631000	12.232669000
C	9.068242000	5.774539000	13.151120000
H	9.043943000	5.364528000	14.185219000
C	10.525991000	5.736559000	12.684815000
H	11.114485000	6.464222000	13.274594000
H	10.584498000	6.076626000	11.628938000
C	5.329407000	12.093287000	13.381416000
C	5.137669000	11.905628000	15.828543000
C	2.874381000	9.412300000	13.502265000
H	2.160288000	9.582052000	12.666397000
H	3.148066000	10.404943000	13.911827000
H	2.317837000	8.868659000	14.294596000

C	5.684068000	7.617416000	17.142001000
H	5.205825000	7.509162000	18.139430000
H	6.474295000	8.390402000	17.219352000
H	6.211891000	6.663238000	16.932364000
C	8.941287000	12.029433000	14.728266000
H	8.192978000	12.744078000	14.325826000
H	9.220222000	12.406512000	15.734858000
C	9.734295000	8.824347000	11.285663000
H	10.401713000	9.237933000	12.077078000
H	10.232862000	7.901232000	10.922210000
C	3.701721000	7.276670000	12.392587000
H	3.206237000	6.596933000	13.114297000
H	4.570257000	6.729511000	11.970018000
H	2.987596000	7.440132000	11.556012000
C	5.797330000	11.589999000	12.023455000
H	6.118808000	10.539366000	12.173157000
C	10.248020000	3.329289000	11.968767000
H	10.634370000	2.294469000	12.082304000
H	10.334498000	3.578762000	10.886794000
C	11.103477000	4.318301000	12.775402000
H	12.156181000	4.304983000	12.420838000
H	11.130335000	4.000773000	13.842703000
C	4.510283000	13.163380000	15.855641000
H	4.199974000	13.588206000	16.822990000
C	3.718324000	6.700878000	15.866757000
H	4.264431000	5.837877000	15.427458000
H	2.851843000	6.914687000	15.211104000
H	3.313680000	6.358291000	16.844534000
C	5.423943000	11.182292000	17.136085000
H	5.688045000	10.146434000	16.863719000
C	8.768574000	3.396885000	12.379584000
H	8.662420000	3.037261000	13.428420000
H	8.160677000	2.711632000	11.751797000
C	7.431341000	7.902762000	10.816932000
H	7.845316000	6.944646000	10.436024000
H	6.436232000	7.669944000	11.245570000
C	8.210781000	4.829986000	12.291912000
H	8.225748000	5.163601000	11.232506000
H	7.150468000	4.840531000	12.615129000
C	4.698310000	13.348948000	13.457245000
H	4.534174000	13.922999000	12.531859000
C	4.281923000	13.887325000	14.679639000
H	3.789405000	14.870975000	14.716308000
C	9.594305000	9.814780000	10.125334000
H	9.206057000	10.778471000	10.519772000
H	10.591210000	10.033474000	9.690638000
C	7.020572000	12.382955000	11.530477000
H	6.791746000	13.466575000	11.461513000
H	7.336123000	12.042583000	10.522400000
H	7.880867000	12.267250000	12.216938000
C	7.280698000	8.885628000	9.644130000

H	6.622905000	8.447723000	8.863978000
H	6.765997000	9.799194000	10.009694000
C	4.672969000	11.610848000	10.973251000
H	3.763014000	11.096724000	11.339748000
H	4.999046000	11.110511000	10.037702000
H	4.384833000	12.648248000	10.705129000
C	8.640901000	9.275202000	9.048032000
H	8.508305000	10.023156000	8.237835000
H	9.101037000	8.378730000	8.573038000
C	4.210076000	11.123245000	18.075300000
H	3.943648000	12.122869000	18.478058000
H	4.421612000	10.465509000	18.943652000
H	3.315888000	10.726045000	17.553266000
C	6.654281000	11.778138000	17.845738000
H	7.554303000	11.738336000	17.197268000
H	6.887306000	11.220640000	18.776984000
H	6.492500000	12.844117000	18.109489000
H	8.292534000	8.721751000	16.108861000
C	10.192706000	11.873782000	13.836422000
H	10.913506000	12.713249000	13.933961000
H	9.895735000	11.827827000	12.765485000
Ga	11.020181000	10.082479000	14.287591000
P	10.942764000	7.219283000	16.254248000
Si	13.762543000	8.402329000	14.883856000
N	12.901230000	9.918912000	14.589409000
C	13.576300000	11.178492000	14.547429000
C	12.616924000	6.980009000	15.495506000
H	12.425327000	6.344317000	14.607369000
H	13.243081000	6.356064000	16.168360000
C	11.331566000	8.028032000	17.900184000
H	11.689084000	9.024388000	17.549496000
C	14.538904000	7.774536000	13.240190000
H	15.147330000	8.644163000	12.907195000
C	15.177812000	8.682651000	16.160999000
H	14.698562000	9.256368000	16.984594000
C	10.388064000	5.450827000	16.540970000
H	10.479253000	5.053985000	15.503201000
C	8.896274000	5.389085000	16.909986000
H	8.331903000	6.098040000	16.269241000
H	8.756836000	5.756115000	17.949712000
C	13.720227000	11.941700000	15.748087000
C	14.062154000	11.718857000	13.319569000
C	16.310922000	9.552456000	15.578566000
H	17.061094000	9.798585000	16.361665000
H	15.943383000	10.507301000	15.157731000
H	16.853873000	9.016747000	14.771100000
C	13.479087000	7.489413000	12.163267000
H	13.915104000	7.518071000	11.141654000
H	12.632381000	8.206086000	12.185587000
H	13.049953000	6.477300000	12.298738000
C	10.063826000	8.275340000	18.737897000

H	9.280472000	8.728687000	18.096764000
H	9.658975000	7.299414000	19.088189000
C	15.766329000	7.404667000	16.788536000
H	16.298543000	6.780225000	16.044110000
H	15.001252000	6.759114000	17.265252000
H	16.503565000	7.665249000	17.578815000
C	13.136340000	11.451920000	17.066260000
H	12.999824000	10.356803000	16.964351000
C	9.191071000	2.978735000	17.639558000
H	8.825508000	1.938646000	17.505641000
H	9.049453000	3.223912000	18.716674000
C	8.359740000	3.956514000	16.793791000
H	7.290268000	3.919253000	17.091166000
H	8.397055000	3.642543000	15.725044000
C	14.703403000	12.971331000	13.319606000
H	15.077116000	13.381897000	12.368307000
C	15.483276000	6.569187000	13.381126000
H	14.969374000	5.695594000	13.837751000
H	16.370797000	6.794845000	14.003550000
H	15.857839000	6.237900000	12.387409000
C	13.854660000	10.994968000	11.999149000
H	13.450168000	9.999470000	12.251086000
C	10.688368000	3.068164000	17.302672000
H	10.852092000	2.719602000	16.257420000
H	11.273706000	2.385960000	17.955010000
C	12.449818000	7.417780000	18.757951000
H	12.144650000	6.414029000	19.119471000
H	13.367937000	7.273956000	18.156644000
C	11.210753000	4.510594000	17.433972000
H	11.121267000	4.830631000	18.494118000
H	12.289451000	4.558326000	17.176238000
C	14.368940000	13.187819000	15.699517000
H	14.482716000	13.772513000	16.624913000
C	14.869460000	13.705277000	14.498389000
H	15.377338000	14.681593000	14.481809000
C	10.367780000	9.158094000	19.956529000
H	10.661078000	10.171931000	19.602471000
H	9.449324000	9.296216000	20.565490000
C	11.739132000	12.051637000	17.294433000
H	11.783952000	13.158268000	17.362444000
H	11.283128000	11.663317000	18.229028000
H	11.056132000	11.801898000	16.460808000
C	12.757691000	8.310415000	19.970469000
H	13.554364000	7.847265000	20.590392000
H	13.168001000	9.278915000	19.609016000
C	14.052355000	11.709235000	18.270751000
H	15.065786000	11.289753000	18.108424000
H	13.629106000	11.247524000	19.186192000
H	14.166437000	12.792203000	18.482488000
C	11.500576000	8.572651000	20.812372000
H	11.735558000	9.246963000	21.663100000

H	11.156922000	7.612010000	21.259855000
C	15.164254000	10.795240000	11.218814000
H	15.577872000	11.758856000	10.854848000
H	14.997090000	10.151183000	10.330571000
H	15.942836000	10.318237000	11.848160000
C	12.796441000	11.709246000	11.139422000
H	11.831903000	11.786207000	11.677390000
H	12.620444000	11.161446000	10.189956000
H	13.115776000	12.740510000	10.881184000
H	10.302029000	9.598455000	15.885217000

TS4‘: G = -9235.07367118 (i = -548 cm-1)

Ga	-1.491530263	1.474825590	0.233937222
Ni	-0.095268868	-0.344158333	0.276140501
P	-1.275212440	-1.322896070	-1.256281828
Si	-4.201306238	-0.214244908	-0.204214067
N	-3.354668325	1.331326874	-0.149669709
C	-4.045382017	2.580077825	-0.167221856
C	-3.060055156	-1.602856709	-0.863442467
H	-3.038903278	-2.381188095	-0.070510578
H	-3.564939784	-2.075062296	-1.730492024
C	-1.337693879	-0.382731873	-2.888435215
H	-1.776230345	0.581772982	-2.537898020
C	-4.788906590	-0.830392295	1.522034826
H	-5.428013907	-0.013870544	1.925377721
C	-5.720092818	-0.025876864	-1.364178286
H	-5.339254509	0.631095215	-2.173943400
C	-0.743352552	-3.101894878	-1.597762150
H	-0.797714477	-3.506971594	-0.562315365
C	0.724504141	-3.184251331	-2.028968827
H	1.318673494	-2.488455611	-1.403884898
H	0.823808368	-2.820970959	-3.073932179
C	-4.303737181	3.258378605	-1.395864257
C	-4.442235512	3.195755971	1.061034624
C	-6.873904486	0.721972728	-0.668557934
H	-7.686858049	0.959333993	-1.389344173
H	-6.540521361	1.678469892	-0.218730135
H	-7.326610443	0.109917937	0.139815511
C	-3.623965263	-1.057672560	2.496757712
H	-3.991508618	-1.332265694	3.509813071
H	-2.971918319	-0.168732939	2.601198729
H	-2.964977780	-1.880588237	2.154081687
C	-0.706772198	3.315836506	-0.029226555
H	-1.470875906	4.001666406	-0.450476178
H	-0.451464678	3.708277722	0.976986974
C	0.057730242	-0.068381609	-3.449669500
H	0.703779584	0.325374242	-2.631483748
H	0.546400064	-1.004434498	-3.793761584
C	-6.209712120	-1.320298043	-2.036289118
H	-6.543046342	-2.080527193	-1.300714159

H	-5.425794222	-1.793012055	-2.663722053
H	-7.074552014	-1.113231170	-2.704019219
C	-3.844418564	2.698278913	-2.735350077
H	-3.543684262	1.646139388	-2.551846600
C	0.382203216	-5.567185510	-2.798447922
H	0.738295868	-6.614913555	-2.704976669
H	0.486869226	-5.295968247	-3.873646855
C	1.256576338	-4.621436471	-1.960672934
H	2.313012082	-4.657921643	-2.302608049
H	1.260662312	-4.964363282	-0.900624581
C	-5.088691997	4.443818293	1.037129359
H	-5.387937944	4.912454394	1.987765983
C	-5.656395812	-2.098710105	1.424710530
H	-5.107380244	-2.937015226	0.943141341
H	-6.584662249	-1.933849720	0.843652188
H	-5.958364130	-2.453522542	2.434706572
C	-4.142008713	2.532956709	2.396715542
H	-3.837337711	1.498753045	2.161533266
C	-1.098801159	-5.465720589	-2.400471307
H	-1.223464803	-5.838161174	-1.358041159
H	-1.718689936	-6.125859211	-3.043427379
C	-2.248963808	-0.929320280	-3.998851999
H	-1.849677404	-1.900404420	-4.362529993
H	-3.268001831	-1.132229634	-3.609559518
C	-1.615079692	-4.016457255	-2.473066825
H	-1.583270368	-3.671444597	-3.528502980
H	-2.677337007	-3.976074910	-2.157891202
C	-4.957573881	4.504489684	-1.371361947
H	-5.154779533	5.025652929	-2.321153927
C	-5.355106810	5.100836506	-0.169691228
H	-5.864696338	6.076430333	-0.174007767
C	-0.008870036	0.923675974	-4.614456094
H	-0.386719507	1.898303528	-4.236888706
H	1.010862018	1.114842446	-5.009406149
C	-2.603006389	3.454631754	-3.242116763
H	-2.811108486	4.540692048	-3.333283931
H	-2.289115881	3.086885803	-4.240558889
H	-1.747670875	3.337066273	-2.549902962
C	-2.325591388	0.052695872	-5.180288806
H	-2.964059330	-0.369869982	-5.984777524
H	-2.828837261	0.981645209	-4.838558147
C	-4.959226716	2.709196194	-3.795534049
H	-5.881266765	2.219386847	-3.423358627
H	-4.633282104	2.177616519	-4.713465180
H	-5.229003331	3.742437250	-4.096231187
C	-0.934130303	0.406120335	-5.726572400
H	-1.019000978	1.154562587	-6.542826324
H	-0.479025826	-0.502834769	-6.182341209
C	-5.367698333	2.460300735	3.320330218
H	-5.680108909	3.463465438	3.678938065
H	-5.144496908	1.845507089	4.216940218

H	-6.236884649	2.007773546	2.801124186
C	-2.945337582	3.199729785	3.099631336
H	-2.039320276	3.173250483	2.460787498
H	-2.704004866	2.681243537	4.051196278
H	-3.154220522	4.265501052	3.329241532
H	-1.020617073	0.261353082	1.447499006
C	0.560184799	3.204173181	-0.899022585
H	1.286693544	4.032641778	-0.763407984
H	0.293573204	3.173907267	-1.977823612
Ga	1.397808376	1.430886580	-0.497435279
P	1.196458921	-1.616237358	1.518283456
Si	4.058516669	-0.310786465	0.188174040
N	3.266010452	1.254615168	-0.093317638
C	3.962442042	2.497548703	-0.062047330
C	2.913476557	-1.758044696	0.810062056
H	2.758652232	-2.378085338	-0.098668808
H	3.549854956	-2.375291087	1.478637540
C	1.454425059	-0.760899257	3.166185697
H	1.600486807	0.289081411	2.823270179
C	4.749831612	-0.962615760	-1.484419323
H	5.414357550	-0.139298880	-1.826187099
C	5.528682836	-0.062665122	1.407471044
H	5.120149800	0.580515110	2.217437077
C	0.742742778	-3.414718243	1.804933328
H	0.905365165	-3.823756190	0.780715716
C	-0.755937573	-3.575996574	2.106772896
H	-1.338063852	-2.933976252	1.413249861
H	-0.974805177	-3.193633887	3.125650147
C	4.070452045	3.213568809	1.169520978
C	4.478911967	3.077360043	-1.258774646
C	6.679579815	0.714553062	0.735433755
H	7.472338243	0.960741509	1.474784605
H	6.339419313	1.666364205	0.283924644
H	7.159359104	0.113038817	-0.065338656
C	3.639400633	-1.144709038	-2.530080695
H	4.048480820	-1.185757923	-3.562336895
H	2.885235114	-0.331287514	-2.501269510
H	3.089476384	-2.090791613	-2.361843291
C	0.180713869	-0.784954732	4.028167791
H	-0.685744646	-0.467363641	3.412870956
H	-0.020446852	-1.827357226	4.361254213
C	6.062469493	-1.346874846	2.068712302
H	6.472787780	-2.059742350	1.325558290
H	5.289602691	-1.889216148	2.650441355
H	6.886535881	-1.105996762	2.775019919
C	3.435614580	2.671702800	2.446254002
H	3.407091972	1.566936732	2.350066793
C	-0.324533307	-5.929702356	2.939042018
H	-0.605735202	-6.998248261	2.827645128
H	-0.542873990	-5.658006209	3.996820164
C	-1.177547665	-5.048238365	2.012831221

H	-2.256122561	-5.156337943	2.253646373
H	-1.054983254	-5.391813335	0.960195934
C	5.112032810	4.332234212	-1.200056312
H	5.508728538	4.777226473	-2.126388460
C	5.599590028	-2.239705892	-1.376204908
H	5.022806914	-3.078955720	-0.930691257
H	6.505950995	-2.092991865	-0.756816716
H	5.939788010	-2.580630507	-2.378785945
C	4.327165545	2.377241298	-2.600433867
H	3.913391138	1.377638632	-2.382351616
C	1.178050777	-5.741282171	2.675415025
H	1.421641198	-6.117296407	1.655714903
H	1.776308493	-6.351188944	3.385113019
C	2.691053909	-1.166226694	3.982523818
H	2.617990805	-2.234181405	4.277173754
H	3.605093137	-1.066926373	3.366061430
C	1.588389725	-4.260323552	2.768764632
H	1.428119080	-3.903468879	3.809300363
H	2.671377073	-4.146137973	2.554018263
C	4.718981922	4.461172631	1.180255505
H	4.809915108	5.015648624	2.125950472
C	5.244552969	5.022850981	0.009175278
H	5.747553815	6.001313099	0.040781701
C	0.333484326	0.103980664	5.269430973
H	0.453199404	1.162432263	4.944949641
H	-0.591918025	0.066589386	5.882082690
C	1.970737022	3.129517927	2.563103460
H	1.888250417	4.235115292	2.530803646
H	1.508014938	2.771907377	3.506075276
H	1.361811439	2.727147840	1.729383531
C	2.829986729	-0.303308093	5.248045835
H	3.705036873	-0.641474895	5.842448581
H	3.048052953	0.742437386	4.942266400
C	4.230154545	3.021226530	3.711932006
H	5.289373608	2.703620135	3.627626348
H	3.790280475	2.520912977	4.598151986
H	4.219181090	4.109988415	3.923962683
C	1.554057843	-0.315974849	6.100750686
H	1.673774924	0.345303531	6.984939304
H	1.386744749	-1.343508236	6.497369933
C	5.673150447	2.184437688	-3.318003216
H	6.112279506	3.152088205	-3.639179534
H	5.546328872	1.559883586	-4.226695224
H	6.413948056	1.686874544	-2.659428918
C	3.306382987	3.095951861	-3.501691903
H	2.314704728	3.160223837	-3.012132752
H	3.178228095	2.556101502	-4.463418338
H	3.628885052	4.132398495	-3.734161876
H	1.329529447	0.041338675	0.421895228

4: G = -9235.10249218

Ga	8.209595000	10.223365000	14.840353000
Ni	9.683235000	8.451348000	14.686606000
P	8.370078000	7.344010000	13.311395000
Si	5.486108000	8.502717000	14.466068000
N	6.338957000	10.045756000	14.574472000
C	5.661900000	11.304650000	14.570311000
C	6.616312000	7.085693000	13.832992000
H	6.693611000	6.374521000	14.682761000
H	6.063415000	6.541967000	13.039948000
C	8.220737000	8.240879000	11.662902000
H	8.047606000	9.288083000	12.001044000
C	4.882458000	7.881029000	16.184207000
H	4.236989000	8.693794000	16.581558000
C	3.974822000	8.731840000	13.300202000
H	4.356422000	9.398535000	12.496662000
C	8.886358000	5.564807000	12.954332000
H	8.671642000	5.101516000	13.944422000
C	10.399601000	5.423303000	12.729719000
H	10.935365000	6.048468000	13.472062000
H	10.670221000	5.835925000	11.736162000
C	5.479901000	12.015624000	13.347040000
C	5.229115000	11.901285000	15.793781000
C	2.830615000	9.480593000	14.012336000
H	2.012464000	9.724966000	13.300291000
H	3.172038000	10.433088000	14.463978000
H	2.383588000	8.865351000	14.821409000
C	6.045980000	7.666074000	17.163754000
H	5.679330000	7.444373000	18.189567000
H	6.719998000	8.543493000	17.231552000
H	6.679572000	6.811775000	16.853857000
C	8.995614000	12.069796000	15.023967000
H	8.303917000	12.859202000	14.664199000
H	9.107709000	12.219837000	16.121008000
C	9.549401000	8.222967000	10.887505000
H	10.378893000	8.521609000	11.560475000
H	9.765328000	7.183163000	10.558384000
C	3.467326000	7.447979000	12.620972000
H	3.132326000	6.686782000	13.354652000
H	4.241328000	6.971936000	11.984150000
H	2.599097000	7.668767000	11.962332000
C	5.984766000	11.455072000	12.023703000
H	6.118952000	10.363071000	12.168494000
C	10.047235000	3.098004000	11.790913000
H	10.329903000	2.027974000	11.883650000
H	10.328253000	3.406181000	10.758162000
C	10.834056000	3.953388000	12.796360000
H	11.926387000	3.865529000	12.614634000
H	10.655539000	3.568395000	13.826229000
C	4.622504000	13.169756000	15.769510000
H	4.293328000	13.625192000	16.716879000

C	4.025040000	6.606223000	16.088442000
H	4.582654000	5.770368000	15.612814000
H	3.098092000	6.762719000	15.502759000
H	3.720248000	6.253459000	17.098242000
C	5.441722000	11.205697000	17.128982000
H	5.813279000	10.194695000	16.891316000
C	8.530085000	3.268956000	11.968553000
H	8.227324000	2.853862000	12.956681000
H	7.981663000	2.682037000	11.201416000
C	7.043971000	7.851038000	10.752100000
H	7.130862000	6.785515000	10.453913000
H	6.083811000	7.949105000	11.297072000
C	8.113678000	4.749730000	11.904883000
H	8.338803000	5.144318000	10.890238000
H	7.017357000	4.844719000	12.049189000
C	4.871111000	13.283665000	13.371758000
H	4.736353000	13.835156000	12.428986000
C	4.437802000	13.864407000	14.569385000
H	3.963359000	14.857542000	14.566110000
C	9.496568000	9.122464000	9.646870000
H	9.365595000	10.180192000	9.967121000
H	10.464350000	9.076890000	9.104182000
C	7.369610000	12.035776000	11.683286000
H	7.333661000	13.143109000	11.624245000
H	7.740832000	11.650772000	10.711237000
H	8.117799000	11.772535000	12.457793000
C	7.004824000	8.725868000	9.487671000
H	6.171448000	8.401243000	8.829297000
H	6.775777000	9.769742000	9.787173000
C	4.986247000	11.668061000	10.875008000
H	3.984283000	11.269859000	11.134692000
H	5.330109000	11.157099000	9.953292000
H	4.866053000	12.741506000	10.622659000
C	8.337483000	8.713584000	8.726835000
H	8.282307000	9.381904000	7.841557000
H	8.529940000	7.688453000	8.335453000
C	4.136680000	11.054331000	17.927717000
H	3.750776000	12.034728000	18.277258000
H	4.298357000	10.422420000	18.825661000
H	3.340868000	10.584445000	17.314675000
C	6.533596000	11.897847000	17.964066000
H	7.491217000	11.941407000	17.409168000
H	6.712722000	11.351481000	18.913837000
H	6.250772000	12.940880000	18.217959000
H	8.700097000	8.785081000	15.834585000
C	10.370047000	12.068945000	14.341082000
H	11.061338000	12.860063000	14.697843000
H	10.257563000	12.214785000	13.243477000
Ga	11.156696000	10.223328000	14.530352000
P	10.996115000	7.343820000	16.061803000
Si	13.880086000	8.503083000	14.907037000

N	13.027147000	10.046014000	14.797647000
C	13.704661000	11.304648000	14.802252000
C	12.749880000	7.085959000	15.539978000
H	12.672511000	6.375075000	14.689974000
H	13.302922000	6.541956000	16.332737000
C	11.145287000	8.240128000	17.710564000
H	11.318165000	9.287451000	17.372783000
C	14.484407000	7.880998000	13.189252000
H	15.130050000	8.693666000	12.792029000
C	15.391231000	8.732604000	16.073163000
H	15.009821000	9.399781000	16.876407000
C	10.480156000	5.564353000	16.418128000
H	10.694885000	5.101516000	15.427826000
C	8.966930000	5.422374000	16.642730000
H	8.430975000	6.047784000	15.900736000
H	8.696258000	5.834399000	17.636515000
C	13.886131000	12.015299000	16.025819000
C	14.138898000	11.901129000	13.579258000
C	16.535827000	9.480650000	15.360909000
H	17.354119000	9.724582000	16.072937000
H	16.194905000	10.433315000	14.909287000
H	16.982513000	8.865149000	14.551844000
C	13.321326000	7.665930000	12.209223000
H	13.688468000	7.443743000	11.183693000
H	12.647591000	8.543508000	12.140693000
H	12.687374000	6.811903000	12.519120000
C	9.816604000	8.221473000	18.485951000
H	8.987133000	8.520677000	17.813194000
H	9.600723000	7.181363000	18.814108000
C	15.898218000	7.448860000	16.753033000
H	16.233046000	6.687230000	16.019734000
H	15.123957000	6.973362000	17.389949000
H	16.766420000	7.669617000	17.411713000
C	13.378865000	11.454944000	17.348320000
H	13.249257000	10.362207000	17.204842000
C	9.319797000	3.096788000	17.580571000
H	9.037349000	2.026728000	17.487482000
H	9.038693000	3.404581000	18.613406000
C	8.532867000	3.952370000	16.575372000
H	7.440532000	3.864140000	16.756898000
H	8.711673000	3.567870000	15.545361000
C	14.746821000	13.168976000	13.604291000
H	15.077234000	13.624264000	12.657278000
C	15.341733000	6.606174000	13.285518000
H	14.783853000	5.770354000	13.760896000
H	16.268399000	6.762649000	13.871653000
H	15.647021000	6.253366000	12.275885000
C	13.926699000	11.206241000	12.243624000
H	13.552906000	10.195838000	12.480474000
C	10.836921000	3.268125000	17.403014000
H	11.139787000	2.853440000	16.414750000

H	11.385437000	2.681053000	18.169966000
C	12.322201000	7.850085000	18.621096000
H	12.235683000	6.784362000	18.918630000
H	13.282292000	7.948812000	18.076125000
C	11.253018000	4.748956000	17.467203000
H	11.027876000	5.143117000	18.482005000
H	12.349317000	4.844227000	17.322891000
C	14.496366000	13.282655000	16.001872000
H	14.631046000	13.833845000	16.944803000
C	14.931373000	13.863121000	14.804706000
H	15.407006000	14.855689000	14.808599000
C	9.869156000	9.119741000	19.727513000
H	9.999365000	10.177922000	19.408480000
H	8.901459000	9.072995000	20.270254000
C	11.990538000	12.030653000	17.682777000
H	12.021779000	13.138203000	17.740328000
H	11.617185000	11.645399000	18.653896000
H	11.246243000	11.763438000	16.905862000
C	12.361108000	8.724110000	19.886065000
H	13.194720000	8.399474000	20.544133000
H	12.589628000	9.768278000	19.587176000
C	14.372051000	11.673448000	18.500513000
H	15.376790000	11.279594000	18.245024000
H	14.026872000	11.161743000	19.421298000
H	14.486295000	12.747612000	18.752564000
C	11.028550000	8.710605000	20.647034000
H	11.083494000	9.378182000	21.532887000
H	10.836625000	7.685043000	21.037553000
C	15.232525000	11.052766000	11.446556000
H	15.620474000	12.032587000	11.097677000
H	15.070965000	10.421254000	10.548305000
H	16.026783000	10.581497000	12.060536000
C	12.837378000	11.900930000	11.407281000
H	11.879268000	11.946861000	11.961105000
H	12.658043000	11.354951000	10.457323000
H	13.123017000	12.943251000	11.153630000
H	10.666494000	8.783830000	13.538386000

Cartesian coordinates (in Å) and free energies (in a.u., at 298 K) of the species in Figure S125.

INT1: G= -9235.08702464

Ga	-1.517020000	1.094987000	0.846734000
Ni	-0.241799000	-0.558431000	0.127090000
P	-1.385476000	-1.087755000	-1.632045000
Si	-4.163199000	-0.197995000	-0.088765000
N	-3.368279000	1.305308000	0.396900000
C	-3.978529000	2.579333000	0.250417000

C	-3.243091000	-0.894131000	-1.620874000
H	-3.702111000	-1.853909000	-1.928292000
H	-3.486671000	-0.179960000	-2.432205000
C	-1.004126000	-0.310212000	-3.307307000
H	-1.375833000	0.725236000	-3.119939000
C	-4.368772000	-1.548999000	1.277970000
H	-5.463860000	-1.494955000	1.474610000
C	-5.961089000	0.212156000	-0.628693000
H	-5.880435000	1.069387000	-1.329277000
C	-1.116826000	-2.954852000	-1.755865000
H	-1.239127000	-3.251843000	-0.690334000
C	0.340485000	-3.265953000	-2.140416000
H	1.022628000	-2.624784000	-1.543223000
H	0.502779000	-2.991996000	-3.205176000
C	-4.057072000	3.234159000	-1.017260000
C	-4.522940000	3.224128000	1.406018000
C	-6.829839000	0.687387000	0.548312000
H	-7.856715000	0.938096000	0.203545000
H	-6.412563000	1.595742000	1.024401000
H	-6.935444000	-0.091289000	1.333768000
C	-3.674323000	-1.321194000	2.627326000
H	-3.972860000	-2.098598000	3.364200000
H	-3.930833000	-0.334438000	3.061599000
H	-2.573320000	-1.359522000	2.526083000
C	0.210581000	3.867447000	-0.555765000
H	-0.214546000	4.753284000	-1.076491000
H	1.028404000	4.232752000	0.094800000
C	0.484167000	-0.185421000	-3.656624000
H	1.058296000	0.188160000	-2.778860000
H	0.905771000	-1.186881000	-3.880125000
C	-6.615965000	-0.964536000	-1.372612000
H	-6.699018000	-1.862763000	-0.723406000
H	-6.047262000	-1.263312000	-2.277278000
H	-7.648023000	-0.710117000	-1.699305000
C	-3.371358000	2.684089000	-2.264918000
H	-2.721334000	1.851601000	-1.922505000
C	-0.316760000	-5.633699000	-2.754118000
H	-0.104972000	-6.709685000	-2.579098000
H	-0.165568000	-5.457640000	-3.843389000
C	0.664070000	-4.755050000	-1.964351000
H	1.710999000	-4.956428000	-2.275780000
H	0.603148000	-5.017481000	-0.884085000
C	-5.180363000	4.457262000	1.267260000
H	-5.607905000	4.945859000	2.155246000
C	-4.091309000	-2.954486000	0.717674000
H	-3.029969000	-3.072658000	0.424298000
H	-4.705901000	-3.180068000	-0.177743000
H	-4.312315000	-3.739280000	1.473529000
C	-4.359194000	2.570770000	2.770652000
H	-4.363749000	1.476297000	2.586784000
C	-1.773440000	-5.307592000	-2.390899000

H	-1.955228000	-5.579852000	-1.326483000
H	-2.472797000	-5.923495000	-2.994916000
C	-1.772163000	-0.867333000	-4.520163000
H	-1.380835000	-1.879834000	-4.761216000
H	-2.849712000	-0.989893000	-4.290492000
C	-2.090041000	-3.813460000	-2.579298000
H	-2.011824000	-3.551128000	-3.655841000
H	-3.139805000	-3.618802000	-2.279805000
C	-4.744796000	4.460290000	-1.109292000
H	-4.821953000	4.957406000	-2.088980000
C	-5.313526000	5.069523000	0.013506000
H	-5.846183000	6.027806000	-0.083582000
C	0.691505000	0.735663000	-4.864499000
H	0.363533000	1.763732000	-4.592509000
H	1.771505000	0.806840000	-5.104949000
C	-2.470084000	3.740505000	-2.930608000
H	-3.071118000	4.546943000	-3.399271000
H	-1.860065000	3.278309000	-3.734495000
H	-1.784289000	4.210323000	-2.203448000
C	-1.588595000	0.049282000	-5.741822000
H	-2.140807000	-0.358996000	-6.614662000
H	-2.045408000	1.039135000	-5.515047000
C	-4.355332000	2.140897000	-3.318991000
H	-4.979364000	1.311232000	-2.938872000
H	-3.808368000	1.771515000	-4.211714000
H	-5.044250000	2.941961000	-3.658558000
C	-0.104266000	0.244062000	-6.082835000
H	0.010835000	0.949752000	-6.932474000
H	0.318880000	-0.727891000	-6.424822000
C	-5.486579000	2.886378000	3.762784000
H	-5.483742000	3.951743000	4.075073000
H	-5.369684000	2.279033000	4.683645000
H	-6.482717000	2.663289000	3.330127000
C	-2.985743000	2.919299000	3.375245000
H	-2.152207000	2.642145000	2.688664000
H	-2.813684000	2.384687000	4.332992000
H	-2.897968000	4.009975000	3.560139000
H	-1.495637000	-0.922026000	0.965810000
C	0.706069000	2.792461000	-1.519536000
H	1.590071000	3.161233000	-2.073013000
H	-0.078356000	2.534322000	-2.261051000
Ga	1.189146000	1.057429000	-0.603669000
P	1.212872000	-1.507151000	1.429714000
Si	4.040880000	-0.337096000	0.171671000
N	3.083387000	1.095418000	-0.171918000
C	3.596254000	2.426289000	-0.204947000
C	2.940079000	-1.751667000	0.835562000
H	2.813737000	-2.461292000	-0.010030000
H	3.530195000	-2.299966000	1.598463000
C	1.371067000	-0.623388000	3.075734000
H	1.514008000	0.427100000	2.740541000

C	4.825539000	-1.106820000	-1.411511000
H	5.473286000	-0.315870000	-1.842990000
C	5.409908000	0.091717000	1.451712000
H	4.875010000	0.703079000	2.210996000
C	0.761811000	-3.316208000	1.747067000
H	0.944364000	-3.727337000	0.726388000
C	-0.722989000	-3.560082000	2.046615000
H	-1.334167000	-2.942926000	1.360530000
H	-0.966368000	-3.201988000	3.069025000
C	3.514032000	3.246470000	0.964281000
C	4.112325000	3.003293000	-1.409222000
C	6.504074000	0.982759000	0.833464000
H	7.233678000	1.312344000	1.605366000
H	6.086760000	1.893901000	0.361277000
H	7.081035000	0.433465000	0.058846000
C	3.751233000	-1.455101000	-2.452123000
H	4.203957000	-1.769067000	-3.417695000
H	3.075687000	-0.602875000	-2.661097000
H	3.107828000	-2.293489000	-2.111185000
C	0.054341000	-0.674377000	3.870682000
H	-0.787267000	-0.369485000	3.216186000
H	-0.147488000	-1.722471000	4.182003000
C	6.037425000	-1.104500000	2.189251000
H	6.605542000	-1.764460000	1.503857000
H	5.286967000	-1.738642000	2.704700000
H	6.750945000	-0.754174000	2.966795000
C	3.064665000	2.688092000	2.309209000
H	3.044710000	1.585659000	2.200959000
C	-0.183558000	-5.898754000	2.861061000
H	-0.404586000	-6.979635000	2.732769000
H	-0.428778000	-5.653092000	3.919361000
C	-1.069466000	-5.050071000	1.936668000
H	-2.143804000	-5.211294000	2.165466000
H	-0.923154000	-5.376309000	0.881738000
C	4.471577000	4.362787000	-1.430572000
H	4.857582000	4.798505000	-2.365655000
C	5.708724000	-2.332259000	-1.120597000
H	5.148970000	-3.125477000	-0.579299000
H	6.596516000	-2.077595000	-0.509297000
H	6.081232000	-2.789162000	-2.063925000
C	4.329397000	2.176989000	-2.668992000
H	3.874490000	1.187280000	-2.479039000
C	1.308545000	-5.623807000	2.617912000
H	1.587016000	-5.980776000	1.600306000
H	1.931706000	-6.201654000	3.333211000
C	2.569310000	-0.997786000	3.962712000
H	2.502494000	-2.065160000	4.260119000
H	3.516175000	-0.883320000	3.400182000
C	1.635912000	-4.123210000	2.722339000
H	1.435720000	-3.779551000	3.760605000
H	2.716045000	-3.957100000	2.532926000

C	3.876512000	4.604049000	0.890790000
H	3.790163000	5.230764000	1.791907000
C	4.343499000	5.173656000	-0.297955000
H	4.615682000	6.239255000	-0.338663000
C	0.112056000	0.207881000	5.123361000
H	0.208999000	1.272013000	4.813087000
H	-0.843867000	0.131031000	5.683281000
C	1.638273000	3.123236000	2.686974000
H	1.531134000	4.227431000	2.670884000
H	1.377228000	2.771019000	3.706145000
H	0.888329000	2.697921000	1.990390000
C	2.615686000	-0.126016000	5.229061000
H	3.469864000	-0.434015000	5.868558000
H	2.814212000	0.925887000	4.930435000
C	4.067289000	3.043376000	3.421754000
H	5.100893000	2.759007000	3.139607000
H	3.811999000	2.520752000	4.366101000
H	4.069200000	4.130411000	3.642715000
C	1.299252000	-0.178372000	6.016275000
H	1.354111000	0.483369000	6.906515000
H	1.142602000	-1.211808000	6.401777000
C	5.836702000	1.978477000	-2.926414000
H	6.322253000	2.942540000	-3.186354000
H	6.009760000	1.276209000	-3.768753000
H	6.355661000	1.582009000	-2.032473000
C	3.659037000	2.772907000	-3.918479000
H	2.568537000	2.899632000	-3.779563000
H	3.817886000	2.112301000	-4.796144000
H	4.083900000	3.765553000	-4.174773000
H	-0.599738000	3.486307000	0.115805000

INT2: G = -9235.08146746

Ga	8.171203000	2.189669000	14.850702000
Ni	9.414271000	4.149634000	14.978236000
P	8.252847000	5.153097000	13.398032000
Si	5.532956000	4.042838000	14.734784000
N	6.265762000	2.450704000	14.766089000
C	5.483226000	1.268880000	14.668128000
C	6.396016000	5.159807000	13.459915000
C	8.647197000	6.974327000	13.076867000
H	8.568396000	7.380520000	14.111951000
C	8.546010000	4.231433000	11.774779000
H	8.032300000	3.266203000	11.996368000
C	5.712710000	4.888314000	16.446621000
H	4.889604000	4.470381000	17.070162000
C	3.697345000	3.900315000	14.210010000
H	3.722880000	3.123326000	13.412244000
C	7.035419000	4.497911000	17.109853000
H	7.914712000	4.621996000	16.414464000
H	7.046065000	3.438811000	17.424615000

H	7.254734000	5.119431000	18.002721000
C	5.162688000	0.732495000	13.387132000
C	5.516267000	6.411873000	16.373417000
H	5.565114000	6.878758000	17.381238000
H	4.536660000	6.683124000	15.929619000
H	6.299688000	6.898028000	15.756600000
C	7.916555000	4.821350000	10.501708000
H	8.431305000	5.773742000	10.250410000
H	6.846563000	5.072079000	10.658632000
C	5.019545000	0.608872000	15.846217000
C	10.107179000	7.145732000	12.627526000
H	10.769122000	6.506887000	13.250214000
H	10.212439000	6.771831000	11.586801000
C	2.797271000	3.370284000	15.338291000
H	2.734159000	4.088147000	16.184088000
H	3.163134000	2.404438000	15.737992000
H	1.759586000	3.204602000	14.975556000
C	5.693266000	1.383661000	12.116985000
H	6.045765000	2.396328000	12.402196000
C	3.963811000	-1.108357000	14.460821000
H	3.375261000	-2.035081000	14.380667000
C	3.123400000	5.189497000	13.596150000
H	2.083424000	5.028875000	13.236950000
H	3.717388000	5.550221000	12.732321000
H	3.080165000	6.016231000	14.337043000
C	10.025039000	3.905536000	11.513406000
H	10.580506000	4.837150000	11.283803000
H	10.491224000	3.506465000	12.439928000
C	8.063142000	3.852755000	9.315319000
H	7.472426000	2.933578000	9.525403000
H	7.621908000	4.304084000	8.401429000
C	10.637760000	0.885871000	13.210547000
H	9.646579000	1.055306000	12.736235000
H	11.388532000	1.126181000	12.429433000
C	4.405908000	-0.450582000	13.307048000
H	4.161878000	-0.869794000	12.318216000
C	7.713960000	7.829934000	12.203251000
H	6.659062000	7.727919000	12.530296000
H	7.749498000	7.480620000	11.150532000
C	10.546861000	8.615396000	12.665476000
H	10.546924000	8.968849000	13.720781000
H	11.593013000	8.711957000	12.305350000
C	5.332814000	1.192409000	17.217468000
H	5.423108000	2.286683000	17.065732000
C	10.183670000	2.921506000	10.349600000
H	9.707870000	1.956299000	10.624763000
H	11.258730000	2.701440000	10.181483000
C	9.602389000	9.496278000	11.834556000
H	9.702544000	9.221876000	10.759739000
H	9.896898000	10.564136000	11.912736000
C	8.139148000	9.308220000	12.263235000

H	7.466639000	9.925703000	11.630947000
H	8.013101000	9.675958000	13.307251000
C	4.272906000	-0.575316000	15.719621000
H	3.916901000	-1.089378000	16.624929000
C	9.531926000	3.472355000	9.072537000
H	10.090065000	4.376810000	8.739347000
H	9.609356000	2.734675000	8.246200000
C	6.916878000	0.600113000	11.609012000
H	7.703228000	0.548135000	12.389027000
H	7.355788000	1.067487000	10.702998000
H	6.640348000	-0.444108000	11.355542000
C	4.226699000	0.962084000	18.257840000
H	3.235179000	1.281934000	17.878549000
H	4.442613000	1.537609000	19.181526000
H	4.147359000	-0.104986000	18.553139000
C	4.631274000	1.550222000	11.020096000
H	4.274975000	0.572822000	10.633409000
H	5.046996000	2.111912000	10.157646000
H	3.746911000	2.104137000	11.396031000
C	6.690112000	0.696228000	17.751657000
H	6.679584000	-0.404937000	17.888128000
H	6.929391000	1.164517000	18.729611000
H	7.517369000	0.922045000	17.048108000
C	10.760361000	-0.575498000	13.646042000
H	10.643792000	-1.270306000	12.784228000
H	9.975972000	-0.839427000	14.384258000
Ga	10.881095000	2.427862000	14.514101000
P	10.896261000	5.353341000	16.073530000
Si	13.720051000	4.084916000	14.919881000
N	12.814514000	2.605591000	14.657848000
C	13.425295000	1.315381000	14.718322000
C	12.573954000	5.547012000	15.356062000
C	10.419435000	7.175451000	16.276653000
H	10.298738000	7.450925000	15.206271000
C	11.272350000	4.640586000	17.771103000
H	11.962987000	3.811356000	17.493178000
C	14.572879000	4.757248000	13.329736000
H	15.268196000	3.964165000	12.991854000
C	14.999211000	3.778200000	16.320903000
H	14.435635000	3.149455000	17.047176000
C	13.542844000	4.994962000	12.212072000
H	12.892249000	5.866630000	12.437536000
H	12.873385000	4.123638000	12.066635000
H	14.036367000	5.208050000	11.239246000
C	13.304645000	0.542745000	15.919285000
C	15.404885000	6.026045000	13.584184000
H	15.845027000	6.412610000	12.638576000
H	16.244477000	5.845179000	14.284542000
H	14.788906000	6.847196000	14.009990000
C	12.005016000	5.532816000	18.781115000
H	11.328133000	6.352152000	19.106667000

H	12.890805000	6.017292000	18.323098000
C	14.109331000	0.731892000	13.604592000
C	9.039354000	7.332969000	16.927619000
H	8.335465000	6.631593000	16.441977000
H	9.093917000	7.032779000	17.998111000
C	16.194850000	2.945959000	15.819971000
H	16.797409000	3.511118000	15.076817000
H	15.875594000	1.997928000	15.344690000
H	16.878134000	2.684253000	16.657475000
C	12.530697000	1.064622000	17.120543000
H	12.426293000	2.158185000	16.973492000
C	14.527925000	-1.310639000	14.896477000
H	14.950602000	-2.324644000	14.960895000
C	15.480078000	5.032331000	17.069180000
H	16.170009000	4.759965000	17.897642000
H	14.644649000	5.604356000	17.519494000
H	16.032759000	5.726746000	16.403831000
C	10.033846000	4.002868000	18.417599000
H	9.297926000	4.801319000	18.650894000
H	9.551905000	3.330572000	17.677400000
C	12.412603000	4.720898000	20.020655000
H	13.115174000	3.914257000	19.711688000
H	12.966748000	5.364749000	20.736005000
C	13.854929000	-0.748714000	15.986032000
H	13.744601000	-1.330893000	16.913396000
C	11.430135000	8.177088000	16.865118000
H	12.414586000	8.088688000	16.362280000
H	11.600673000	7.962896000	17.938451000
C	8.519510000	8.773263000	16.824184000
H	8.331552000	9.010248000	15.751800000
H	7.539266000	8.863487000	17.337443000
C	14.298011000	1.457300000	12.280963000
H	13.751295000	2.414837000	12.362877000
C	10.378189000	3.243009000	19.705107000
H	10.981094000	2.347785000	19.443307000
H	9.448828000	2.861425000	20.178390000
C	9.528535000	9.780548000	17.392263000
H	9.630346000	9.614673000	18.489006000
H	9.156241000	10.819308000	17.266949000
C	10.905111000	9.617921000	16.731441000
H	11.635455000	10.329298000	17.171881000
H	10.826649000	9.881938000	15.652260000
C	14.646281000	-0.563761000	13.720760000
H	15.167186000	-1.002449000	12.855306000
C	11.177305000	4.103887000	20.693937000
H	10.529312000	4.925942000	21.074896000
H	11.472989000	3.502890000	21.579766000
C	11.109380000	0.472175000	17.148065000
H	10.546938000	0.697657000	16.215270000
H	10.512856000	0.875972000	17.990433000
H	11.137584000	-0.633342000	17.233802000

C	15.789248000	1.753277000	12.021534000
H	16.270305000	2.246666000	12.888251000
H	15.918315000	2.406040000	11.133030000
H	16.349897000	0.814303000	11.830309000
C	13.264363000	0.835963000	18.450757000
H	13.332308000	-0.240468000	18.710109000
H	12.731442000	1.335774000	19.284916000
H	14.296683000	1.238708000	18.413340000
C	13.711476000	0.686860000	11.082907000
H	14.244114000	-0.271906000	10.912808000
H	13.807115000	1.285856000	10.153074000
H	12.640379000	0.449076000	11.229435000
H	6.002615000	4.911661000	12.452028000
H	6.060389000	6.195999000	13.665520000
H	12.417783000	6.113358000	14.414979000
H	13.168962000	6.217284000	16.010145000
H	8.284276000	0.598361000	14.883070000
H	11.741995000	-0.793611000	14.109112000

TS5: G = -9235.03419974 (i = -1085 cm-1)

Ga	8.138703000	2.161229000	14.779957000
Ni	9.375385000	4.214673000	15.020798000
P	8.235021000	5.125790000	13.409128000
Si	5.505897000	4.018317000	14.717757000
N	6.218802000	2.417480000	14.692097000
C	5.430336000	1.243644000	14.565707000
C	6.375267000	5.157016000	13.467056000
C	8.652471000	6.946308000	13.100843000
H	8.590897000	7.338801000	14.142416000
C	8.504136000	4.217870000	11.768503000
H	7.958410000	3.266443000	11.973897000
C	5.728619000	4.816489000	16.449242000
H	4.919877000	4.384907000	17.082283000
C	3.659407000	3.917154000	14.222817000
H	3.655759000	3.174937000	13.391583000
C	7.066724000	4.407639000	17.068110000
H	7.930821000	4.590175000	16.357064000
H	7.096858000	3.334772000	17.332324000
H	7.302061000	4.990386000	17.982450000
C	5.089804000	0.747185000	13.273369000
C	5.545527000	6.343455000	16.425439000
H	5.633515000	6.780536000	17.443902000
H	4.555769000	6.640280000	16.022568000
H	6.315365000	6.837057000	15.797305000
C	7.886199000	4.853776000	10.512032000
H	8.432395000	5.791842000	10.273198000
H	6.827552000	5.140261000	10.683282000
C	4.982678000	0.545319000	15.728740000
C	10.111186000	7.095443000	12.640168000
H	10.763239000	6.444253000	13.259774000

H	10.204009000	6.719985000	11.598591000
C	2.780741000	3.350322000	15.350565000
H	2.742719000	4.036907000	16.223383000
H	3.148965000	2.367781000	15.705341000
H	1.733687000	3.205190000	15.006278000
C	5.632798000	1.421907000	12.021445000
H	5.947866000	2.443128000	12.320700000
C	3.876119000	-1.107839000	14.306360000
H	3.270743000	-2.021607000	14.205535000
C	3.091435000	5.239958000	13.678987000
H	2.038254000	5.114638000	13.344875000
H	3.668663000	5.624409000	12.813970000
H	3.088568000	6.035175000	14.454549000
C	9.967426000	3.839924000	11.490793000
H	10.566200000	4.753016000	11.297354000
H	10.406919000	3.378199000	12.403281000
C	7.985875000	3.906278000	9.304313000
H	7.366193000	3.003552000	9.501702000
H	7.551022000	4.391648000	8.404912000
C	10.549873000	0.653735000	13.391308000
H	9.963780000	0.795663000	12.458994000
H	11.588686000	0.952432000	13.116587000
C	4.315016000	-0.422237000	13.167375000
H	4.053968000	-0.808502000	12.169758000
C	7.724446000	7.827170000	12.247210000
H	6.672445000	7.742399000	12.588117000
H	7.738959000	7.488377000	11.190717000
C	10.578154000	8.556833000	12.677177000
H	10.598340000	8.908097000	13.733006000
H	11.621339000	8.635463000	12.304113000
C	5.353897000	1.067583000	17.109804000
H	5.445100000	2.167626000	17.004105000
C	10.084902000	2.885899000	10.297022000
H	9.582235000	1.926980000	10.551105000
H	11.151548000	2.639771000	10.111966000
C	9.639062000	9.458733000	11.862958000
H	9.717424000	9.186727000	10.785671000
H	9.954987000	10.520521000	11.940777000
C	8.179462000	9.296357000	12.313848000
H	7.509147000	9.932734000	11.698151000
H	8.078293000	9.656963000	13.363139000
C	4.215630000	-0.622680000	15.576716000
H	3.869199000	-1.162819000	16.470505000
C	9.438100000	3.482650000	9.038246000
H	10.022944000	4.374960000	8.718352000
H	9.483894000	2.760182000	8.196123000
C	6.896313000	0.675416000	11.555109000
H	7.664073000	0.673320000	12.357500000
H	7.338679000	1.140430000	10.650026000
H	6.664183000	-0.383642000	11.319356000
C	4.295925000	0.792668000	18.187682000

H	3.291886000	1.142067000	17.872389000
H	4.560846000	1.316148000	19.129358000
H	4.217520000	-0.287754000	18.429942000
C	4.603231000	1.560280000	10.891664000
H	4.295082000	0.575420000	10.482911000
H	5.027613000	2.144569000	10.048761000
H	3.687824000	2.079394000	11.241956000
C	6.732913000	0.539764000	17.548705000
H	6.720125000	-0.565562000	17.645714000
H	7.040475000	0.972675000	18.523968000
H	7.520841000	0.788803000	16.802564000
C	10.609845000	-0.813902000	13.821937000
H	11.005299000	-1.458928000	13.008163000
H	9.607055000	-1.195905000	14.108970000
Ga	10.924949000	2.613239000	14.594312000
P	10.881539000	5.416689000	16.110260000
Si	13.737354000	4.112413000	14.924386000
N	12.873289000	2.605596000	14.654301000
C	13.449743000	1.307997000	14.751909000
C	12.550007000	5.562376000	15.332569000
C	10.436440000	7.242900000	16.316029000
H	10.279119000	7.507795000	15.247296000
C	11.301223000	4.715016000	17.803551000
H	11.988178000	3.884131000	17.520604000
C	14.590649000	4.761369000	13.326030000
H	15.283292000	3.954773000	13.008738000
C	15.005078000	3.838220000	16.338209000
H	14.438025000	3.230992000	17.080082000
C	13.554969000	4.968484000	12.206013000
H	12.932279000	5.868684000	12.394356000
H	12.858124000	4.110661000	12.113347000
H	14.042528000	5.116637000	11.218511000
C	13.293145000	0.548833000	15.955262000
C	15.423992000	6.035490000	13.544036000
H	15.861515000	6.398260000	12.587841000
H	16.265236000	5.871818000	14.246665000
H	14.808215000	6.866233000	13.951052000
C	12.050246000	5.620325000	18.789448000
H	11.380273000	6.447343000	19.109762000
H	12.930229000	6.094549000	18.310004000
C	14.115481000	0.697824000	13.641395000
C	9.074544000	7.399928000	17.006891000
H	8.364467000	6.677360000	16.560903000
H	9.166501000	7.129874000	18.083081000
C	16.192460000	2.985990000	15.850347000
H	16.801486000	3.536054000	15.101324000
H	15.863300000	2.036309000	15.384535000
H	16.871604000	2.726413000	16.691720000
C	12.550560000	1.107493000	17.161564000
H	12.467459000	2.201580000	17.001566000
C	14.457899000	-1.350590000	14.940070000

H	14.845492000	-2.378044000	15.011360000
C	15.494135000	5.108693000	17.052035000
H	16.179802000	4.855328000	17.890021000
H	14.661256000	5.699116000	17.482866000
H	16.053236000	5.779431000	16.368148000
C	10.075053000	4.080445000	18.477564000
H	9.336876000	4.877945000	18.708808000
H	9.585591000	3.393908000	17.756311000
C	12.474263000	4.830777000	20.037745000
H	13.174295000	4.019526000	19.734923000
H	13.036308000	5.487677000	20.734912000
C	13.800761000	-0.761761000	16.024848000
H	13.669391000	-1.337359000	16.953903000
C	11.459673000	8.256378000	16.857834000
H	12.428657000	8.161836000	16.325744000
H	11.663784000	8.062040000	17.929172000
C	8.540788000	8.833194000	16.881120000
H	8.325038000	9.041300000	15.807887000
H	7.573308000	8.931751000	17.416919000
C	14.297623000	1.426758000	12.318911000
H	13.815677000	2.413715000	12.438178000
C	10.441280000	3.342801000	19.772490000
H	11.047515000	2.447508000	19.517459000
H	9.520972000	2.962414000	20.264324000
C	9.558496000	9.859217000	17.399893000
H	9.686725000	9.719889000	18.497616000
H	9.177031000	10.892736000	17.259283000
C	10.921555000	9.691684000	16.711407000
H	11.656008000	10.416886000	17.121548000
H	10.816496000	9.934263000	15.629462000
C	14.607313000	-0.613684000	13.759516000
H	15.114625000	-1.072661000	12.896121000
C	11.248435000	4.223817000	20.736624000
H	10.602675000	5.051239000	21.109749000
H	11.555690000	3.639828000	21.629878000
C	11.115339000	0.554297000	17.238090000
H	10.519586000	0.824642000	16.337901000
H	10.570964000	0.958920000	18.114747000
H	11.111958000	-0.552848000	17.307558000
C	15.787712000	1.646250000	11.997835000
H	16.322427000	2.119381000	12.845273000
H	15.910903000	2.294135000	11.104868000
H	16.298280000	0.683628000	11.784901000
C	13.312015000	0.885511000	18.478157000
H	13.357346000	-0.187284000	18.757076000
H	12.814904000	1.417009000	19.315188000
H	14.353952000	1.257642000	18.406478000
C	13.592974000	0.713282000	11.149839000
H	14.028777000	-0.290148000	10.961521000
H	13.695560000	1.301780000	10.214102000
H	12.512068000	0.575653000	11.350762000

H	5.978415000	4.937422000	12.454010000
H	6.053423000	6.192469000	13.697413000
H	12.363673000	6.081805000	14.369787000
H	13.161890000	6.270579000	15.931020000
H	9.336764000	1.004175000	14.190691000
H	11.284293000	-0.945744000	14.690181000

INT3: G = -9235.10399070

Ga	8.453036000	2.924187000	15.363426000
Ni	9.777840000	4.602080000	14.708780000
P	8.357190000	5.305690000	13.240139000
Si	5.596857000	3.990849000	14.409498000
N	6.662043000	2.608353000	14.653304000
C	6.166414000	1.278720000	14.743004000
C	6.570519000	5.479043000	13.679068000
C	8.814893000	7.097264000	12.865196000
H	8.461928000	7.591668000	13.800579000
C	8.353848000	4.339380000	11.633153000
H	8.294438000	3.294680000	12.010211000
C	4.922868000	4.676450000	16.079375000
H	4.429896000	3.812706000	16.577707000
C	4.142684000	3.473300000	13.268343000
H	4.618602000	2.808327000	12.515470000
C	6.059899000	5.173710000	16.985350000
H	6.519655000	6.100762000	16.583299000
H	6.886821000	4.438341000	17.089154000
H	5.696627000	5.409806000	18.008961000
C	6.051998000	0.479034000	13.563720000
C	3.866882000	5.780453000	15.893509000
H	3.532757000	6.187177000	16.873341000
H	2.964608000	5.414678000	15.365398000
H	4.267953000	6.637973000	15.310732000
C	7.160319000	4.571491000	10.689893000
H	7.133823000	5.632484000	10.363281000
H	6.205961000	4.380946000	11.220361000
C	5.828633000	0.700967000	16.007037000
C	10.339598000	7.300946000	12.811460000
H	10.790683000	6.763318000	13.672787000
H	10.754949000	6.814650000	11.904193000
C	3.107174000	2.628695000	14.036394000
H	2.565862000	3.235362000	14.792908000
H	3.573578000	1.772959000	14.564011000
H	2.341278000	2.214310000	13.345111000
C	6.529515000	1.001440000	12.214912000
H	6.495230000	2.108339000	12.265546000
C	5.220672000	-1.395605000	14.903192000
H	4.851019000	-2.430466000	14.962242000
C	3.473936000	4.627063000	12.501688000
H	2.677026000	4.244431000	11.827134000
H	4.193307000	5.185824000	11.868319000

H	2.996759000	5.361624000	13.181979000
C	9.691980000	4.482606000	10.886505000
H	9.813574000	5.532342000	10.540964000
H	10.526242000	4.272081000	11.588786000
C	7.245643000	3.662771000	9.452631000
H	7.125759000	2.608820000	9.780727000
H	6.395896000	3.875318000	8.769948000
C	9.117979000	-1.161161000	15.102587000
H	9.052336000	-2.265682000	15.189568000
H	8.174276000	-0.814093000	14.636684000
C	5.571116000	-0.837967000	13.667129000
H	5.481845000	-1.451859000	12.758539000
C	8.122161000	7.802190000	11.689651000
H	7.020935000	7.672336000	11.740965000
H	8.455328000	7.340680000	10.734674000
C	10.700044000	8.790771000	12.799457000
H	10.383744000	9.248919000	13.764078000
H	11.801490000	8.919646000	12.736646000
C	6.005185000	1.475122000	17.305134000
H	6.209301000	2.523909000	17.019916000
C	9.760924000	3.551738000	9.670479000
H	9.738664000	2.496405000	10.021699000
H	10.729917000	3.686032000	9.144529000
C	10.001207000	9.519424000	11.640429000
H	10.403503000	9.133080000	10.676315000
H	10.235574000	10.604785000	11.663285000
C	8.480240000	9.299491000	11.673359000
H	7.997093000	9.798749000	10.806716000
H	8.061162000	9.780448000	12.586264000
C	5.364336000	-0.625766000	16.061778000
H	5.111293000	-1.065170000	17.039445000
C	8.584817000	3.802955000	8.716829000
H	8.669870000	4.831148000	8.296287000
H	8.624861000	3.107276000	7.852011000
C	8.004853000	0.618220000	11.998379000
H	8.646602000	1.058719000	12.787343000
H	8.380037000	0.981378000	11.019275000
H	8.138476000	-0.482149000	12.030056000
C	4.740908000	1.471336000	18.178984000
H	3.855706000	1.814109000	17.605627000
H	4.867850000	2.143655000	19.052688000
H	4.510970000	0.458881000	18.571715000
C	5.646763000	0.543370000	11.045066000
H	5.713136000	-0.551461000	10.878503000
H	5.961122000	1.030488000	10.100102000
H	4.580225000	0.792042000	11.221078000
C	7.234476000	0.974800000	18.086416000
H	7.132469000	-0.094815000	18.364817000
H	7.384117000	1.563159000	19.015647000
H	8.157606000	1.071515000	17.474775000
C	10.346001000	-0.732877000	14.303066000

H	10.322825000	-1.118069000	13.263557000
H	11.296758000	-1.068400000	14.762223000
Ga	11.120053000	2.950432000	14.031945000
P	11.180502000	5.300312000	16.195692000
Si	13.946536000	3.952428000	15.061643000
N	12.873920000	2.579730000	14.797298000
C	13.352217000	1.243620000	14.717697000
C	12.972885000	5.451718000	15.769552000
C	10.746951000	7.100934000	16.558099000
H	11.110630000	7.581375000	15.619401000
C	11.161823000	4.340554000	17.806205000
H	11.215929000	3.293660000	17.434639000
C	14.659483000	4.631030000	13.404032000
H	15.152502000	3.761535000	12.915605000
C	15.371430000	3.423344000	16.233924000
H	14.872427000	2.769160000	16.981709000
C	13.548667000	5.142510000	12.472609000
H	13.088959000	6.073625000	12.865739000
H	12.716562000	4.416386000	12.344699000
H	13.940142000	5.377495000	11.459204000
C	13.430644000	0.444336000	15.899208000
C	15.724398000	5.722921000	13.610499000
H	16.081756000	6.125857000	12.637342000
H	16.612197000	5.347001000	14.155687000
H	15.322003000	6.584884000	14.185748000
C	12.349322000	4.570044000	18.757686000
H	12.382576000	5.633616000	19.074985000
H	13.305960000	4.366233000	18.236628000
C	13.702373000	0.659803000	13.460230000
C	9.225929000	7.329584000	16.606793000
H	8.766714000	6.789466000	15.751698000
H	8.802079000	6.862316000	17.520323000
C	16.410176000	2.561415000	15.489817000
H	16.970850000	3.156841000	14.738376000
H	15.943452000	1.707129000	14.960225000
H	17.159578000	2.143580000	16.196903000
C	12.924686000	0.973796000	17.234442000
H	12.982802000	2.079995000	17.187848000
C	14.260257000	-1.443696000	14.577172000
H	14.617477000	-2.483409000	14.526380000
C	16.041645000	4.573317000	17.005016000
H	16.816081000	4.184258000	17.701633000
H	15.318111000	5.151605000	17.615709000
H	16.546834000	5.292054000	16.328219000
C	9.817943000	4.498302000	18.539201000
H	9.697018000	5.552369000	18.871467000
H	8.988542000	4.284138000	17.832550000
C	12.246435000	3.673914000	20.002641000
H	12.359391000	2.615756000	19.685543000
H	13.092814000	3.885502000	20.689772000
C	13.894021000	-0.879608000	15.806168000

H	13.957898000	-1.493790000	16.716773000
C	11.447319000	7.805957000	17.729178000
H	12.546551000	7.658998000	17.682179000
H	11.104848000	7.357878000	18.687322000
C	8.889345000	8.824969000	16.601547000
H	9.216424000	9.267557000	15.633266000
H	7.789876000	8.972163000	16.658678000
C	13.561945000	1.436729000	12.159142000
H	13.370890000	2.488881000	12.441353000
C	9.733110000	3.581689000	19.765278000
H	9.751828000	2.522299000	19.426582000
H	8.760606000	3.727892000	20.281521000
C	9.595498000	9.553721000	17.755936000
H	9.183807000	9.182770000	18.722108000
H	9.378927000	10.642473000	17.722091000
C	11.112716000	9.308895000	17.730656000
H	11.600842000	9.808230000	18.594458000
H	11.542681000	9.774591000	16.814917000
C	14.148183000	-0.673640000	13.415108000
H	14.413343000	-1.117701000	12.442699000
C	10.902945000	3.834731000	20.725893000
H	10.823640000	4.868682000	21.133177000
H	10.849243000	3.149810000	21.598500000
C	11.436412000	0.617678000	17.408559000
H	10.821852000	1.067745000	16.602719000
H	11.042500000	0.988551000	18.377361000
H	11.285724000	-0.480480000	17.375580000
C	14.841710000	1.412660000	11.308572000
H	15.721239000	1.743648000	11.897380000
H	14.740692000	2.084763000	10.431298000
H	15.063750000	0.395984000	10.922262000
C	13.762877000	0.497160000	18.429195000
H	13.676302000	-0.597447000	18.587626000
H	13.423831000	0.983261000	19.366160000
H	14.837733000	0.732945000	18.289336000
C	12.338429000	0.959070000	11.354472000
H	12.430413000	-0.110234000	11.071217000
H	12.213855000	1.555392000	10.426563000
H	11.406195000	1.064020000	11.949800000
H	6.007758000	5.907986000	12.824475000
H	6.562952000	6.258419000	14.470226000
H	12.994395000	6.224076000	14.971753000
H	13.532152000	5.883549000	16.625046000
H	9.137846000	-0.753799000	16.133331000
H	10.405444000	0.377202000	14.226412000

19. References

1 J. Hicks, P. Vasko, J. M. Goicoechea and S. Aldridge, *Nature*, 2018, **557**, 92–95.
2 A. J. Sicard and R. T. Baker, *Org. Process Res. Dev.*, 2020, **24**, 2950–2952.
3 T. L. Kalkuhl, I. Fernández and T. J. Hadlington, *Chem*, 2025, **11**, 102349.
4 D. S. Raiford, C. L. Fisk and E. D. Becker, *Anal. Chem.*, 1979, **51**, 2050–2051.
5 M. Muhr, P. Heiß, M. Schütz, R. Bühler, C. Gemel, M. H. Linden, H. B. Linden and R. A. Fischer, *Dalton Trans.*, 2021, **50**, 9031–9036.
6 D. S. Raiford, C. L. Fisk and E. D. Becker, *Anal. Chem.*, 1979, **51**, 2050–2051.
7 J. Spielmann, F. Buch and S. Harder, *Angew Chem Int Ed*, 2008, **47**, 9434–9438.
8 H. E. Gottlieb, V. Kotlyar and A. Nudelman, *J. Org. Chem.*, 1997, **62**, 7512–7515.
9 H. Jia, S. Du, C. Xu and Z. Mo, *Eur J Inorg Chem*, DOI:10.1002/ejic.202300086.
10 M. Hruzd, S. L. Kleynemeyer, C. Michon, S. Bastin, E. Pollet, V. Ritleng and J.-B. Sortais, *Chem. Commun.*, 2025, **61**, 2969–2972.
11 M. Flinker, H. Yin, R. W. Juhl, E. Z. Eikeland, J. Overgaard, D. U. Nielsen and T. Skrydstrup, *Angew Chem Int Ed*, 2017, **56**, 15910–15915.
12 D. P. Zobernig, M. Luxner, B. Stöger, L. F. Veiros and K. Kirchner, *Chemistry A European J*, 2024, **30**, e202302455.
13 H. Bauer, K. Thum, M. Alonso, C. Fischer and S. Harder, *Angew Chem Int Ed*, 2019, **58**, 4248–4253.
14 A. J. MacNair, M.-M. Tran, J. E. Nelson, G. U. Sloan, A. Ironmonger and S. P. Thomas, *Org. Biomol. Chem.*, 2014, **12**, 5082–5088.
15 Y. Wang, W. Chen, Z. Lu, Z. H. Li and H. Wang, *Angew Chem Int Ed*, 2013, **52**, 7496–7499.
16 J. Martin, C. Knüpfer, J. Eyselein, C. Färber, S. Grams, J. Langer, K. Thum, M. Wiesinger and S. Harder, *Angew Chem Int Ed*, 2020, **59**, 9102–9112.
17 K. Nikki, H. Inakura, Wu-Le, N. Suzuki and T. Endo, *J. Chem. Soc., Perkin Trans. 2*, 2001, 2370–2373.
18 M. R. Jalilian and M. Zahedi-Tabrizi, *Spectrochimica Acta Part A: Molecular and Biomolecular Spectroscopy*, 2008, **69**, 278–281.
19 H. Bauer, M. Alonso, C. Fischer, B. Rösch, H. Elsen and S. Harder, *Angew Chem Int Ed*, 2018, **57**, 15177–15182.
20 Z. Mazloomi, R. Pretorius, O. Pàmies, M. Albrecht and M. Diéguez, *Inorg. Chem.*, 2017, **56**, 11282–11298.
21 J. Pinkas, J. Kubišta, R. Gyepes, K. Mach and M. Horáček, *Eur J Inorg Chem*, 2020, **2020**, 128–136.
22 M. Mehta, M. H. Holthausen, I. Mallov, M. Pérez, Z. Qu, S. Grimme and D. W. Stephan, *Angew Chem Int Ed*, 2015, **54**, 8250–8254.
23 G. M. Sheldrick, SHELXL-97, Program for Crystal Strucutre Refinement, Göttingen, 1997.
24 G. M. Sheldrick, *Acta Crystallogr C Struct Chem*, 2015, **71**, 3–8.
25 F. Neese, *WIREs Comput Mol Sci*, DOI:10.1002/wcms.70019.
26 A. D. Becke, *Phys. Rev. A*, 1988, **38**, 3098–3100.
27 J. P. Perdew, *Phys. Rev. B*, 1986, **33**, 8822–8824.
28 S. Grimme, J. Antony, S. Ehrlich and H. Krieg, *The Journal of Chemical Physics*, DOI:10.1063/1.3382344.
29 S. Grimme, S. Ehrlich and L. Goerigk, *J Comput Chem*, 2011, **32**, 1456–1465.
30 F. Neese, *J Comput Chem*, 2003, **24**, 1740–1747.
31 F. Weigend and R. Ahlrichs, *Phys. Chem. Chem. Phys.*, 2005, **7**, 3297.
32 V. Ásgeirsson, B. O. Birgisson, R. Bjornsson, U. Becker, F. Neese, C. Riplinger and H. Jónsson, *J. Chem. Theory Comput.*, 2021, **17**, 4929–4945.
33 In *Reviews in Computational Chemistry*, Wiley, 1st edn., 2000, pp. 1–86.
34 D. S. Levine and M. Head-Gordon, *Proc. Natl. Acad. Sci. U.S.A.*, 2017, **114**, 12649–12656.
35 M. P. Mitoraj, A. Michalak and T. Ziegler, *J. Chem. Theory Comput.*, 2009, **5**, 962–975.
36 ADF2020, SCM, Theoretical Chemistry, Vrije Universiteit, Amsterdam, The Netherlands, http://www.scm.com.
37 J. G. Snijders, P. Vernooijs and E. J. Baerends, *Atomic Data and Nuclear Data Tables*, 1981, **26**, 483–509.

38 J. Krijn, E. J. Baerends, Fit Functions in the HFS-Method, Internal Report (in Dutch), Vrije Universiteit Amsterdam, The Netherlands, 1984.
39 E. V. Lenthe, E. J. Baerends and J. G. Snijders, *The Journal of Chemical Physics*, 1993, **99**, 4597–4610.
40 E. Van Lenthe, E. J. Baerends and J. G. Snijders, *The Journal of Chemical Physics*, 1994, **101**, 9783–9792.
41 E. Van Lenthe, A. Ehlers and E.-J. Baerends, *The Journal of Chemical Physics*, 1999, **110**, 8943–8953.
